# Supplementary material for: Associations between alcohol consumption and gray and white matter volumes in the UK Biobank
Source: Nat Commun. 2022 Mar 4;13:1175. doi: 10.1038/s41467-022-28735-5 (PMC8897479; doi:10.1038/s41467-022-28735-5)
Supplement: Supplementary file 1 — Supplementary Information [file 41467_2022_28735_MOESM1_ESM.pdf]

Supplementary Information (SI) for

Associations Between Alcohol Consumption and Gray and White Matter Volumes in the UK Biobank

Table of Contents

Supplementary Tables

Supplementary Figures

Supplementary Table 1. Regression Results: Volume of Gray Matter (whole brain, normalized)
log intake is measured in standardized log(1 + daily units of alcohol).

	Standard Control Variables<br>N: 36,678 (df: 36,585), R^2: 0.514		Extended Control Variables<br>N: 36,678 (df: 36,578), R^2: 0.520	
**Variable**	**Regression Coefficient (SE), 95% CI**	**t-stat (p-value)**	**Regression Coefficient (SE), 95% CI**	**t-stat (p-value)**
log intake	-0.1095 (0.0058), CI: [-0.1209,-0.0982]	-19.0 (p < 1.0e-16)	-0.1125 (0.0057), CI: [-0.1238,-0.1013]	-19.6 (p < 1.0e-16)
log intake^2	-0.0651 (0.0037), CI: [-0.0723,-0.0579]	-17.7 (p < 1.0e-16)	-0.0596 (0.0037), CI: [-0.0668,-0.0524]	-16.2 (p < 1.0e-16)
log intake x male	0.0174 (0.0080), CI: [0.0018,0.0330]	2.2 (p = 2.9e-02)	0.0224 (0.0079), CI: [0.0068,0.0379]	2.8 (p = 4.8e-03)
log intake x std. age	0.0080 (0.0037), CI: [0.0008,0.0152]	2.2 (p = 3.0e-02)	0.0080 (0.0037), CI: [0.0008,0.0151]	2.2 (p = 3.0e-02)
standardized age	-0.5991 (0.0038), CI: [-0.6066,-0.5916]	-157.0 (p < 1.0e-16)	-0.5995 (0.0038), CI: [-0.6069,-0.5921]	-158.0 (p < 1.0e-16)
standardized age^2	-0.0378 (0.0034), CI: [-0.0445,-0.0311]	-11.0 (p < 1.0e-16)	-0.0403 (0.0034), CI: [-0.0469,-0.0336]	-11.8 (p < 1.0e-16)
	**Against model without log intake and interactions**<br>Delta R^2: 0.0099<br>F-test: p < 1.0e-16		**Against model without log intake and interactions**<br>Delta R^2: 0.0099<br>F-test: p < 1.0e-16	

	Excluding Abstainers<br>N: 33,773 (df: 33,676), R2: 0.517		Excluding Those that Consume a High Level of Alcohol<br>N: 34,383 (df: 34,286), R2: 0.510	
**Variable**	**Regression Coefficient (SE), 95% CI**	**t-stat (p-value)**	**Regression Coefficient (SE), 95% CI**	**t-stat (p-value)**
log intake	-0.1025 (0.0064), CI: [-0.1151,-0.0899]	-15.9 (p < 1.0e-16)	-0.0960 (0.0073), CI: [-0.1104,-0.0817]	-13.1 (p < 1.0e-16)
log intake^2	-0.0701 (0.0045), CI: [-0.0790,-0.0611]	-15.4 (p < 1.0e-16)	-0.0499 (0.0049), CI: [-0.0595,-0.0402]	-10.1 (p < 1.0e-16)
log intake x male	0.0114 (0.0095), CI: [-0.0072,0.0299]	1.2 (p = 2.3e-01)	0.0214 (0.0088), CI: [0.0041,0.0387]	2.4 (p = 1.5e-02)
log intake x std. age	0.0127 (0.0043), CI: [0.0042,0.0212]	2.9 (p = 3.4e-03)	0.0100 (0.0040), CI: [0.0020,0.0179]	2.5 (p = 1.4e-02)
standardized age	-0.6012 (0.0040), CI: [-0.6091,-0.5933]	-149.9 (p < 1.0e-16)	-0.5989 (0.0040), CI: [-0.6066,-0.5911]	-151.2 (p < 1.0e-16)
standardized age^2	-0.0389 (0.0036), CI: [-0.0459,-0.0319]	-11.0 (p < 1.0e-16)	-0.0388 (0.0035), CI: [-0.0457,-0.0319]	-11.0 (p < 1.0e-16)
	**Against model without log intake and interactions**<br>Delta R^2: 0.0108<br>F-test: p < 1.0e-16		**Against model without log intake and interactions**<br>Delta R^2: 0.0038<br>F-test: p < 1.0e-16	

Supplementary Table 2. Regression Results: Volume of White Matter (whole brain, normalized)
log intake is measured in standardized log(1 + daily units of alcohol).

	**Standard Control Variables**		**Extended Control Variables**	
	N: 36,678 (df: 36,585), R^2: 0.122		N: 36,678 (df: 36,578), R^2: 0.123	
**Variable**	**Regression Coefficient (SE), 95% CI**	**t-stat (p-value)**	**Regression Coefficient (SE), 95% CI**	**t-stat (p-value)**
log intake	-0.0650 (0.0078), CI: [-0.0802,-0.0498]	-8.4 (p < 1.0e-16)	-0.0632 (0.0078), CI: [-0.0784,-0.0479]	-8.1 (p = 4.3e-16)
log intake^2	-0.0370 (0.0050), CI: [-0.0468,-0.0273]	-7.5 (p = 7.8e-14)	-0.0378 (0.0050), CI: [-0.0475,-0.0280]	-7.6 (p = 2.9e-14)
log intake x male	0.0164 (0.0107), CI: [-0.0046,0.0374]	1.5 (p = 1.2e-01)	0.0148 (0.0107), CI: [-0.0062,0.0358]	1.4 (p = 1.7e-01)
log intake x std. age	0.0111 (0.0050), CI: [0.0014,0.0208]	2.2 (p = 2.5e-02)	0.0110 (0.0050), CI: [0.0013,0.0207]	2.2 (p = 2.6e-02)
standardized age	-0.3213 (0.0051), CI: [-0.3313,-0.3112]	-62.6 (p < 1.0e-16)	-0.3218 (0.0051), CI: [-0.3319,-0.3118]	-62.7 (p < 1.0e-16)
standardized age^2	-0.0127 (0.0046), CI: [-0.0217,-0.0037]	-2.8 (p = 5.7e-03)	-0.0126 (0.0046), CI: [-0.0216,-0.0036]	-2.7 (p = 6.2e-03)
	**Against model without log intake and interactions**		**Against model without log intake and interactions**	
	Delta R^2: 0.0033		Delta R^2: 0.0032	
	F-test: p < 1.0e-16		F-test: p < 1.0e-16	

	**Excluding Abstainers**		**Excluding Those that Consume a High Level of Alcohol**	
	N: 33,773 (df: 33,676), R2: 0.124		N: 34,383 (df: 34,286), R2: 0.124	
**Variable**	**Regression Coefficient (SE), 95% CI**	**t-stat (p-value)**	**Regression Coefficient (SE), 95% CI**	**t-stat (p-value)**
log intake	-0.0599 (0.0087), CI: [-0.0769,-0.0429]	-6.9 (p = 4.6e-12)	-0.0596 (0.0098), CI: [-0.0789,-0.0403]	-6.1 (p = 1.4e-09)
log intake^2	-0.0369 (0.0061), CI: [-0.0489,-0.0249]	-6.0 (p = 1.7e-09)	-0.0327 (0.0066), CI: [-0.0456,-0.0197]	-4.9 (p = 7.9e-07)
log intake x male	0.0073 (0.0127), CI: [-0.0176,0.0323]	0.6 (p = 5.7e-01)	0.0148 (0.0119), CI: [-0.0084,0.0381]	1.3 (p = 2.1e-01)
log intake x std. age	0.0187 (0.0059), CI: [0.0072,0.0301]	3.2 (p = 1.4e-03)	0.0088 (0.0054), CI: [-0.0019,0.0194]	1.6 (p = 1.1e-01)
standardized age	-0.3251 (0.0054), CI: [-0.3357,-0.3146]	-60.2 (p < 1.0e-16)	-0.3223 (0.0053), CI: [-0.3327,-0.3119]	-60.6 (p < 1.0e-16)
standardized age^2	-0.0133 (0.0048), CI: [-0.0227,-0.0040]	-2.8 (p = 5.3e-03)	-0.0135 (0.0047), CI: [-0.0228,-0.0043]	-2.9 (p = 4.2e-03)
	**Against model without log intake and interactions**		**Against model without log intake and interactions**	
	Delta R^2: 0.0036		Delta R^2: 0.0014	
	F-test: p < 1.0e-16		F-test: p = 5.0e-13	

Supplementary Figure 1. Bar plots representing the average residual volume of whole-brain gray and white matter volume for individuals grouped by the number of daily alcohol units after excluding heavy drinkers and controlling for standard control variables. The mean residuals are in terms of standard deviations of the dependent variable, where zero represents the average residual in the sample. The error bars represent the 95% confidence interval. **p*<0.01 and ***p*<.0001 for groups showing a significant difference in pairwise two-tailed t-test (uncorrected) against the group consuming up to one alcohol unit daily. F = females, M = males.

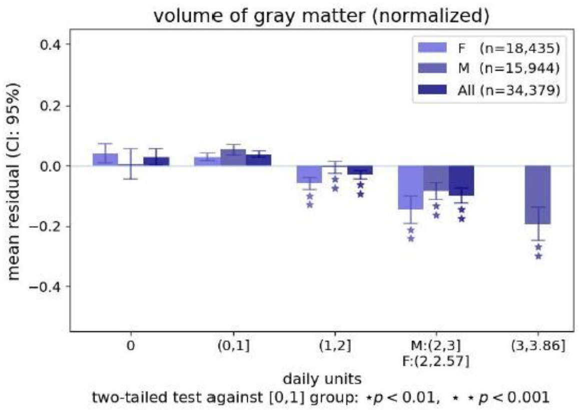

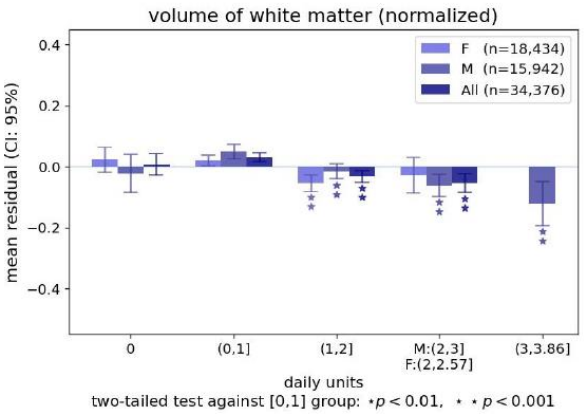

Supplementary Figure 2. Associations between alcohol intake and regional gray matter volume imaging-derived phenotypes predicted from the regression model with standard controls, arranged into brain lobes along the x-axis. For these variables, the significance of the correlation is plotted vertically in units of –log10(p-value). The dotted horizontal line indicates the threshold corresponding to multiple correction using the Holm method (1.64 x 10^{-4}).

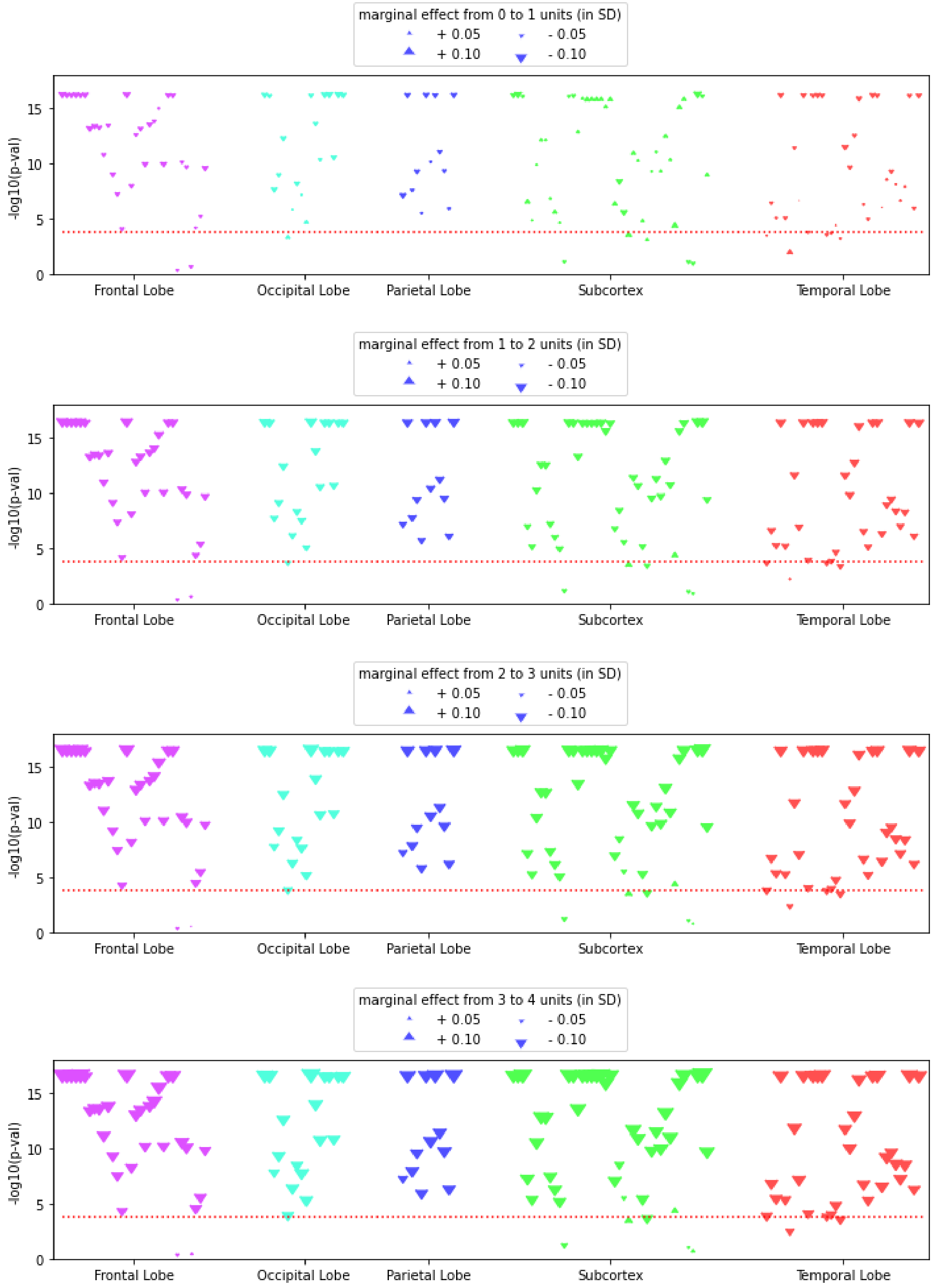

Supplementary Figure 3. Bar plots representing the average residual volume of localized gray matter volume imaging-derived phenotypes for individuals grouped by the number of daily alcohol units after excluding heavy drinkers and controlling for standard control variables. The mean residuals are in terms of standard deviations of the dependent variable, where zero represents the average residual in the sample. The error bars represent the 95% confidence interval. *$p<0.01$ and **$p<.0001$ for groups showing a significant difference in pairwise two-tailed t-test (uncorrected) against the group consuming up to one alcohol unit daily. F = females, M = males, l = left, r = right.

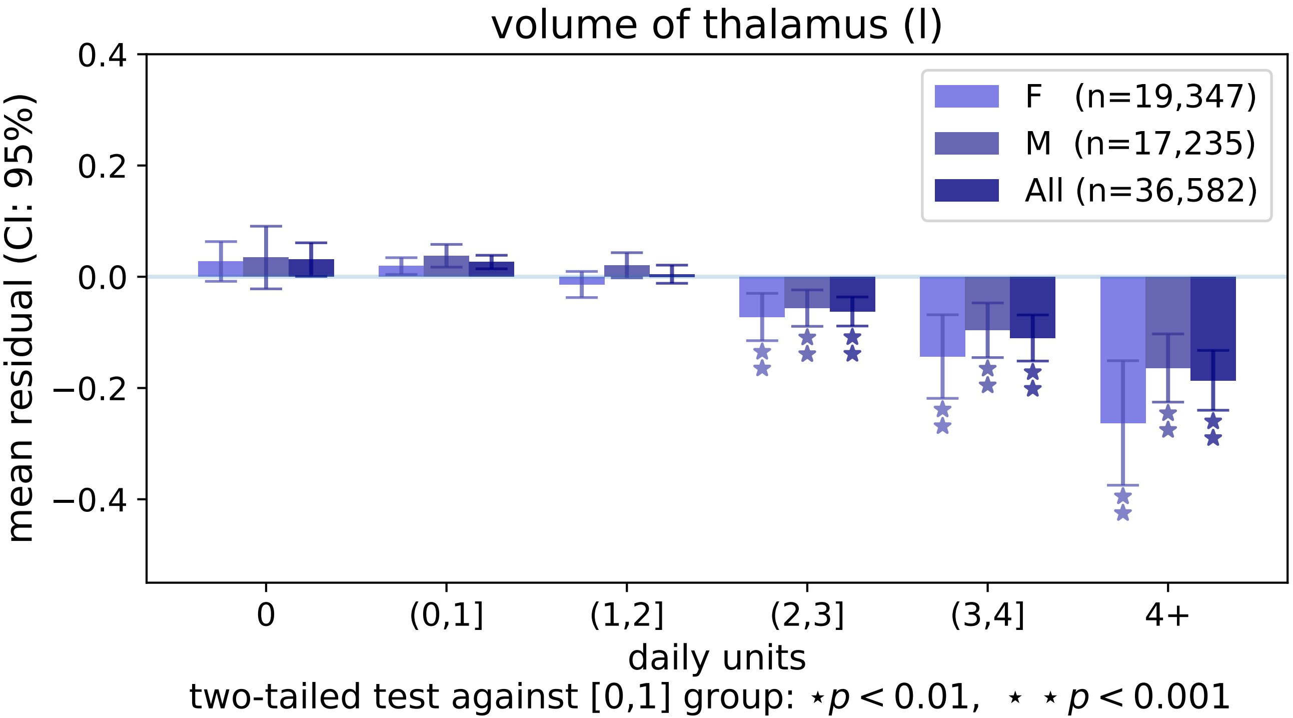

volume of thalamus (l)
mean residual (CI: 95%)
0.4
0.2
0.0
−0.2
−0.4
F (n=19,347)
M (n=17,235)
All (n=36,582)
0
(0,1]
(1,2]
(2,3]
(3,4]
4+
daily units
two-tailed test against [0,1] group: ⋆p < 0.01, ⋆ ⋆ p < 0.001

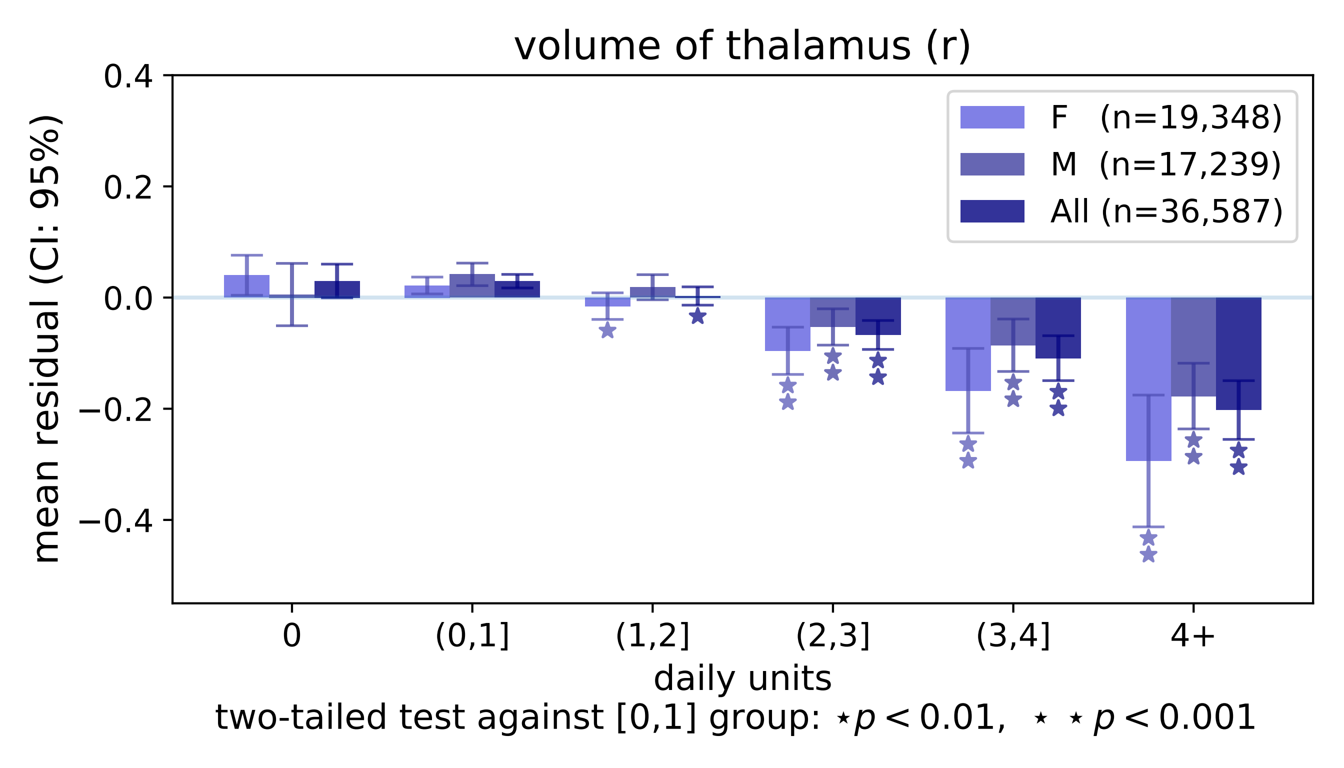
volume of thalamus (r)
mean residual (CI: 95%)
0.4
0.2
0.0
−0.2
−0.4
F (n=19,348)
M (n=17,239)
All (n=36,587)
0
(0,1]
(1,2]
(2,3]
(3,4]
4+
daily units
two-tailed test against [0,1] group: ⋆p < 0.01, ⋆ ⋆ p < 0.001

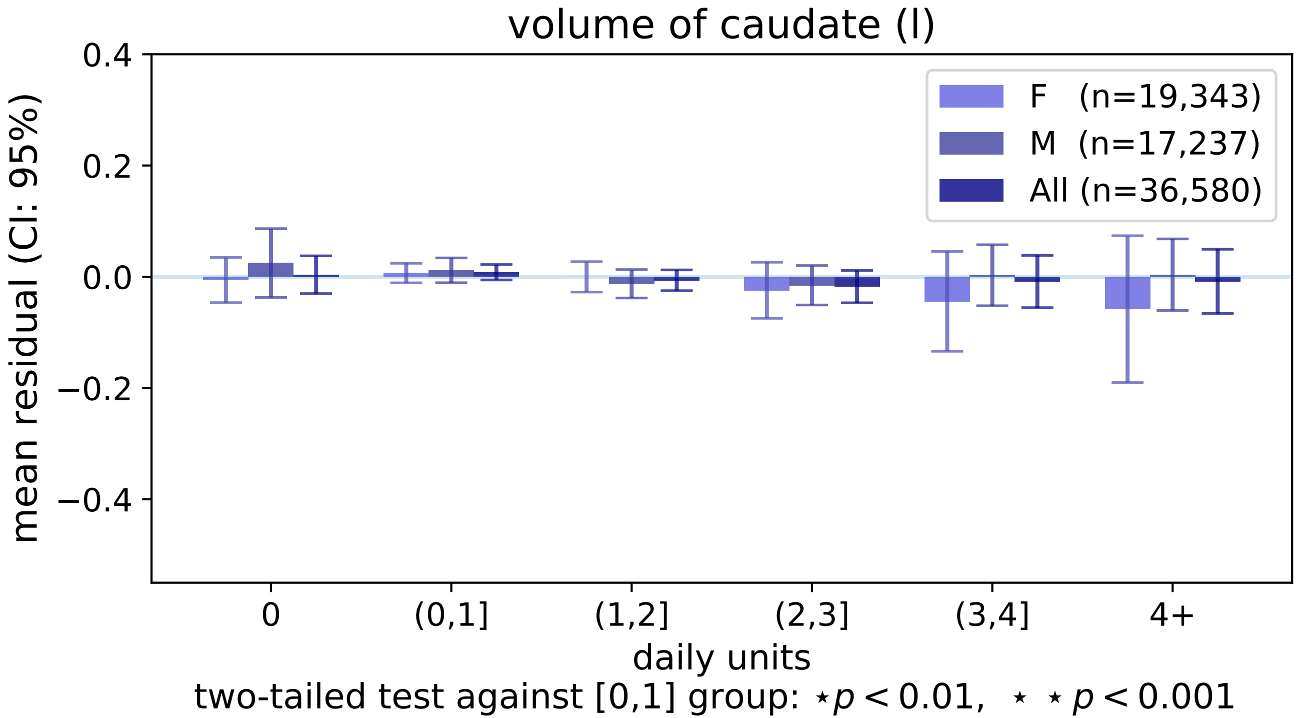
volume of caudate (l)
F (n=19,343)
M (n=17,237)
All (n=36,580)
mean residual (CI: 95%)
0.4
0.2
0.0
−0.2
−0.4
0
(0,1]
(1,2]
(2,3]
(3,4]
4+
daily units
two-tailed test against [0,1] group: ⋆p < 0.01, ⋆ ⋆ p < 0.001

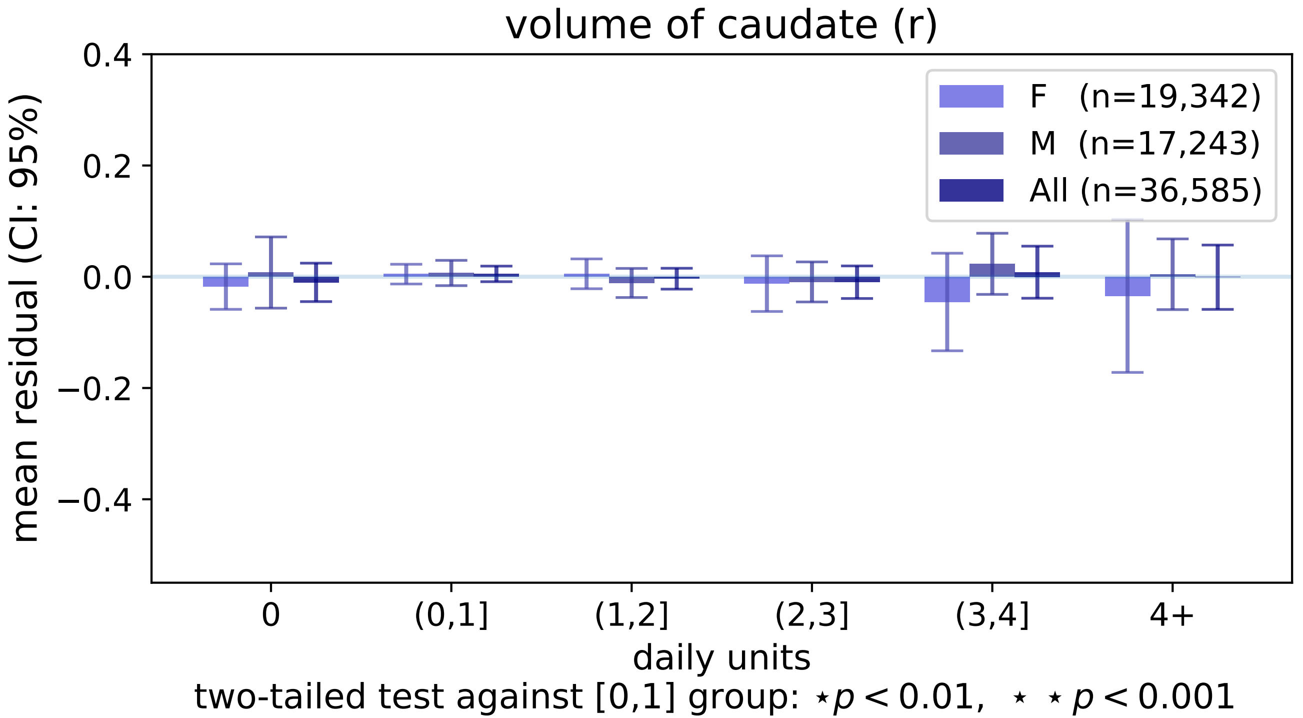
volume of caudate (r)
mean residual (CI: 95%)
0.4
0.2
0.0
−0.2
−0.4
F (n=19,342)
M (n=17,243)
All (n=36,585)
0
(0,1]
(1,2]
(2,3]
(3,4]
4+
daily units
two-tailed test against [0,1] group: ⋆p < 0.01, ⋆ ⋆ p < 0.001

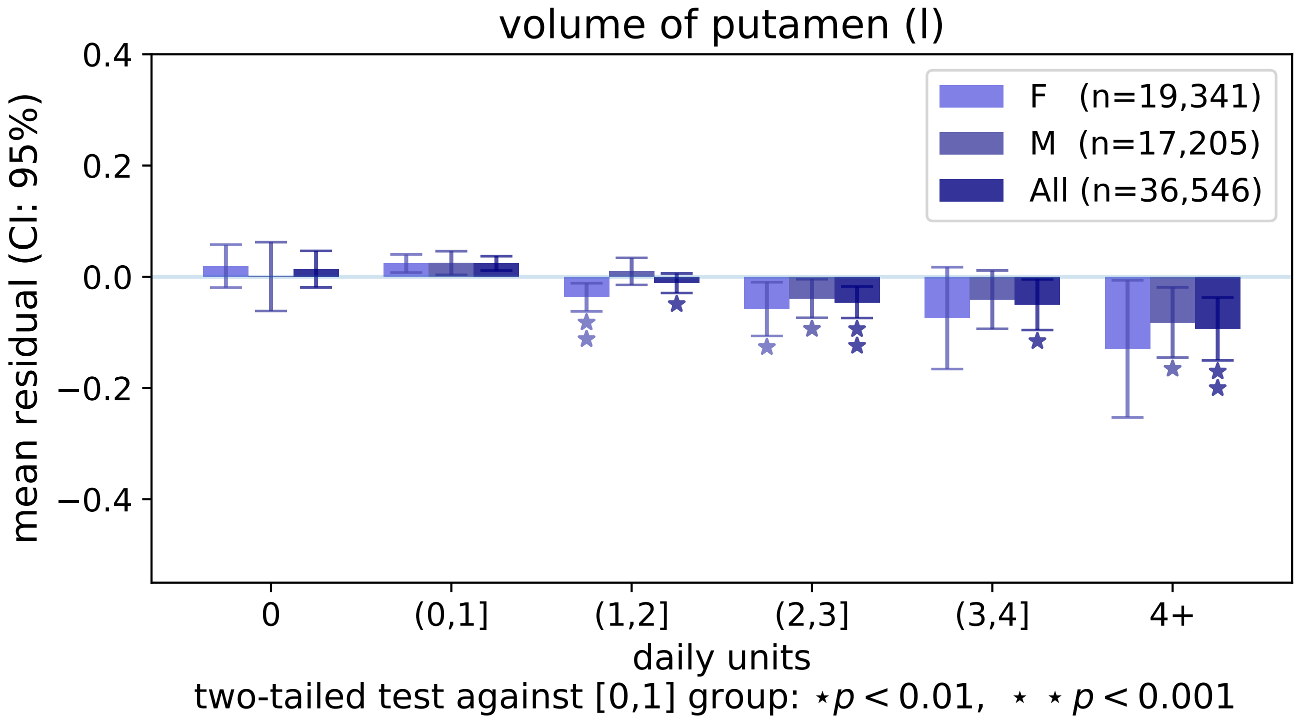
volume of putamen (l)
mean residual (CI: 95%)
0.4
0.2
0.0
−0.2
−0.4
F (n=19,341)
M (n=17,205)
All (n=36,546)
0
(0,1]
(1,2]
(2,3]
(3,4]
4+
daily units
two-tailed test against [0,1] group: ⋆ $p < 0.01$, ⋆ ⋆ $p < 0.001$

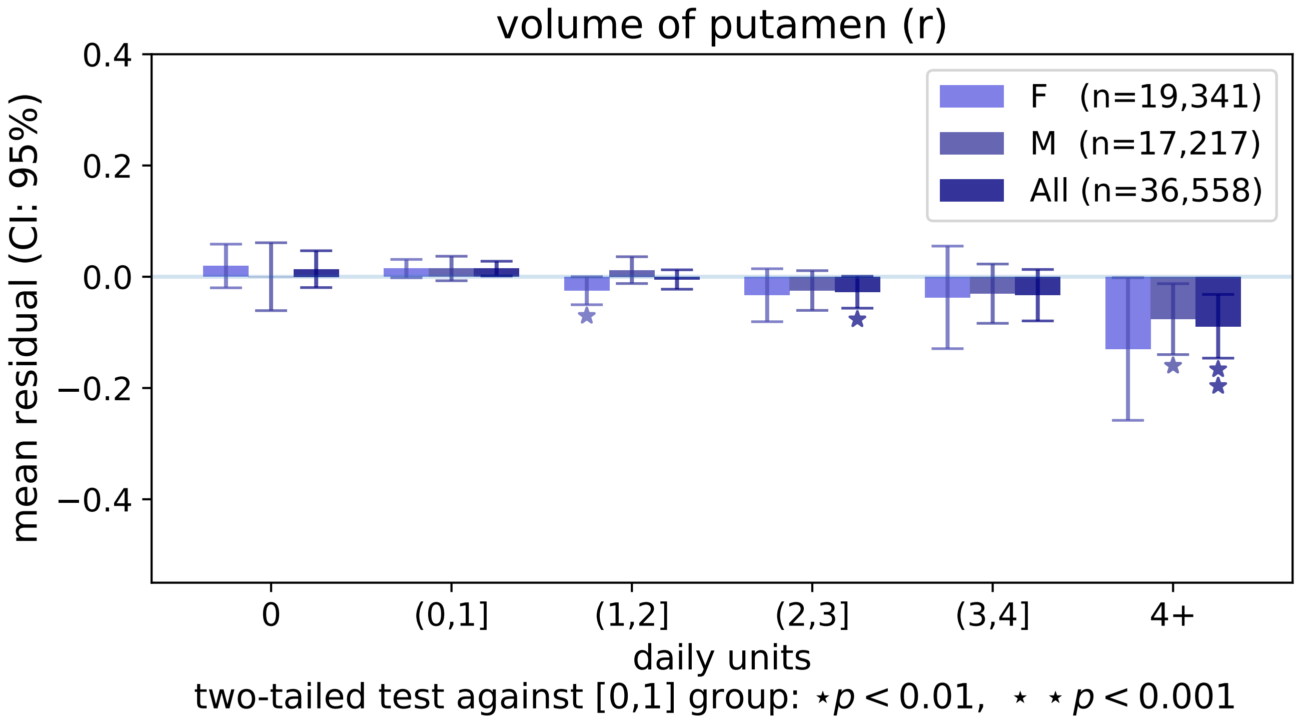

volume of putamen (r)
0.4
0.2
0.0
−0.2
−0.4
mean residual (CI: 95%)
F (n=19,341)
M (n=17,217)
All (n=36,558)
0
(0,1]
(1,2]
(2,3]
(3,4]
4+
daily units
two-tailed test against [0,1] group: ⋆ p < 0.01, ⋆ ⋆ p < 0.001

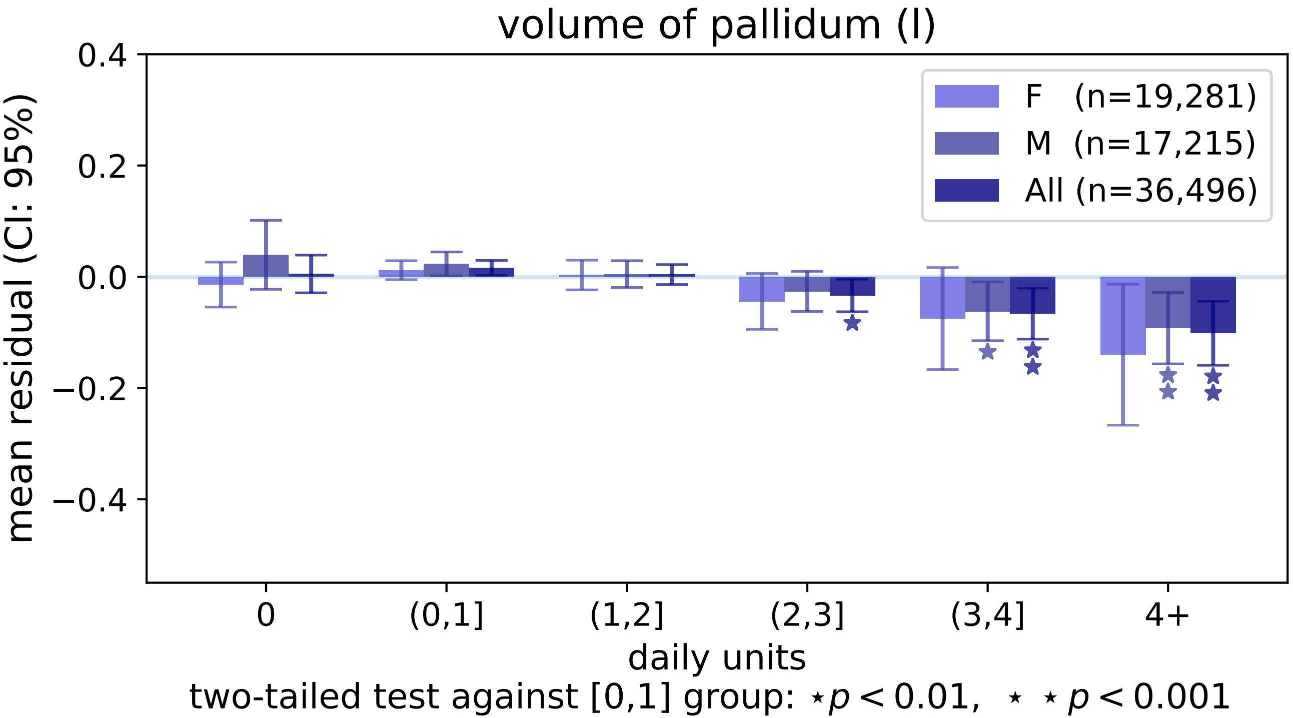

volume of pallidum (l)
0.4
0.2
0.0
−0.2
−0.4
mean residual (CI: 95%)
F (n=19,281)
M (n=17,215)
All (n=36,496)
0
(0,1]
(1,2]
(2,3]
(3,4]
4+
daily units
two-tailed test against [0,1] group: ⋆p < 0.01, ⋆ ⋆ p < 0.001

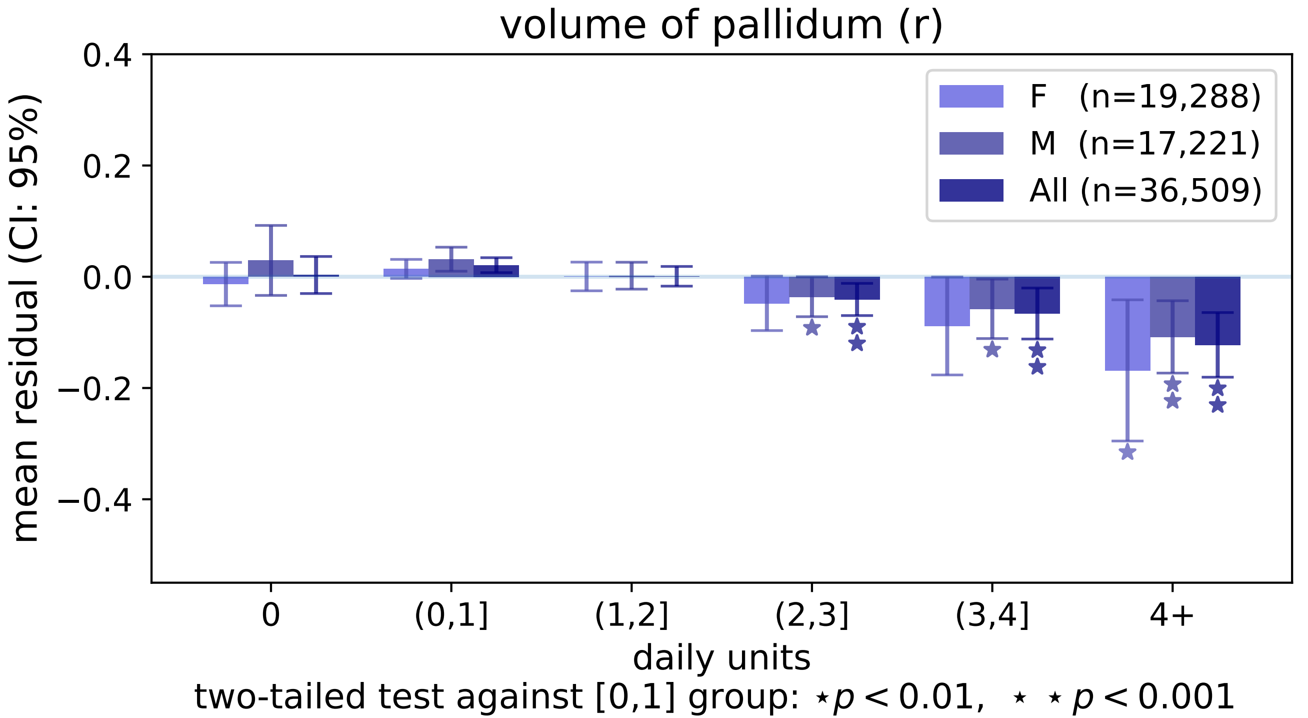
volume of pallidum (r)
mean residual (CI: 95%)
0.4
0.2
0.0
−0.2
−0.4
F (n=19,288)
M (n=17,221)
All (n=36,509)
0
(0,1]
(1,2]
(2,3]
(3,4]
4+
daily units
two-tailed test against [0,1] group: ⋆p < 0.01, ⋆ ⋆ p < 0.001

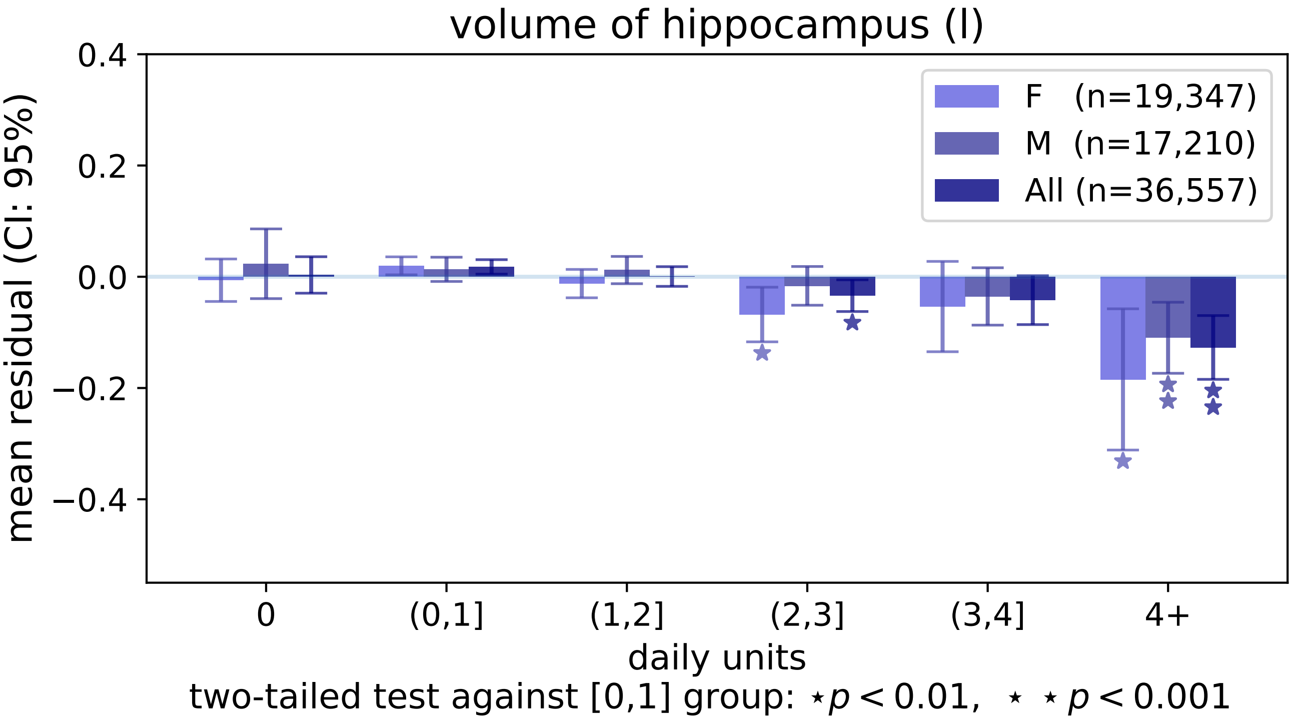

volume of hippocampus (l)
F (n=19,347)
M (n=17,210)
All (n=36,557)
mean residual (CI: 95%)
0.4
0.2
0.0
−0.2
−0.4
0
(0,1]
(1,2]
(2,3]
(3,4]
4+
daily units
two-tailed test against [0,1] group: ⋆p < 0.01, ⋆ ⋆ p < 0.001

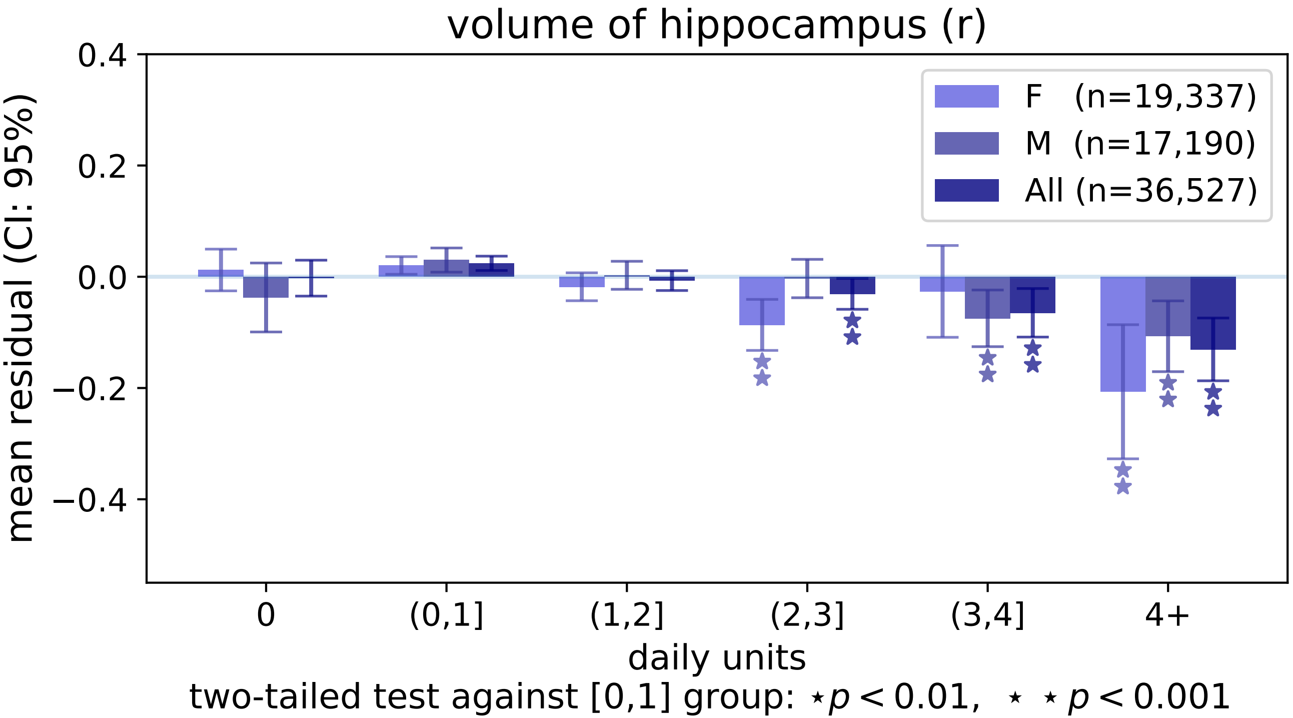
volume of hippocampus (r)
mean residual (CI: 95%)
0.4
0.2
0.0
−0.2
−0.4
F (n=19,337)
M (n=17,190)
All (n=36,527)
0
(0,1]
(1,2]
(2,3]
(3,4]
4+
daily units
two-tailed test against [0,1] group: ⋆ $p < 0.01$, ⋆ ⋆ $p < 0.001$

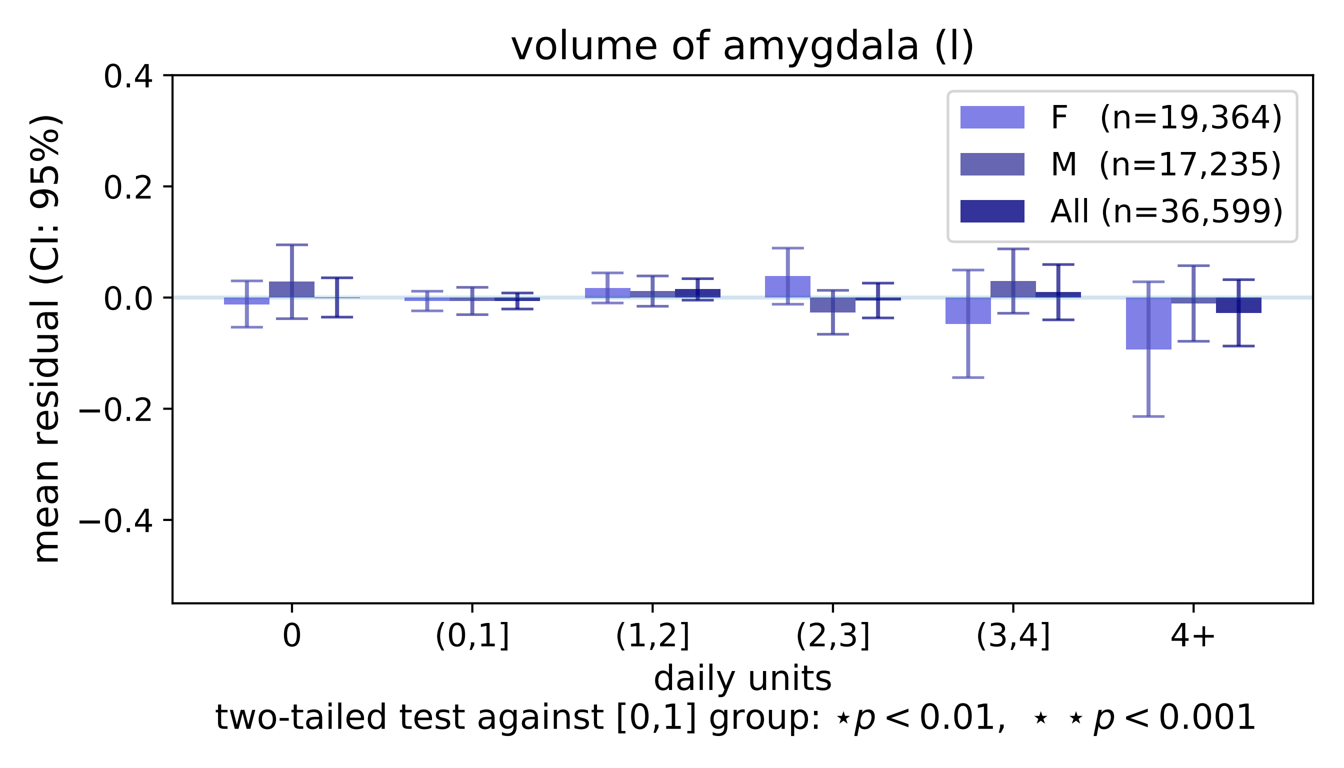

volume of amygdala (l)
mean residual (CI: 95%)
0.4
0.2
0.0
−0.2
−0.4
F (n=19,364)
M (n=17,235)
All (n=36,599)
0
(0,1]
(1,2]
(2,3]
(3,4]
4+
daily units
two-tailed test against [0,1] group: ⋆p < 0.01, ⋆ ⋆ p < 0.001

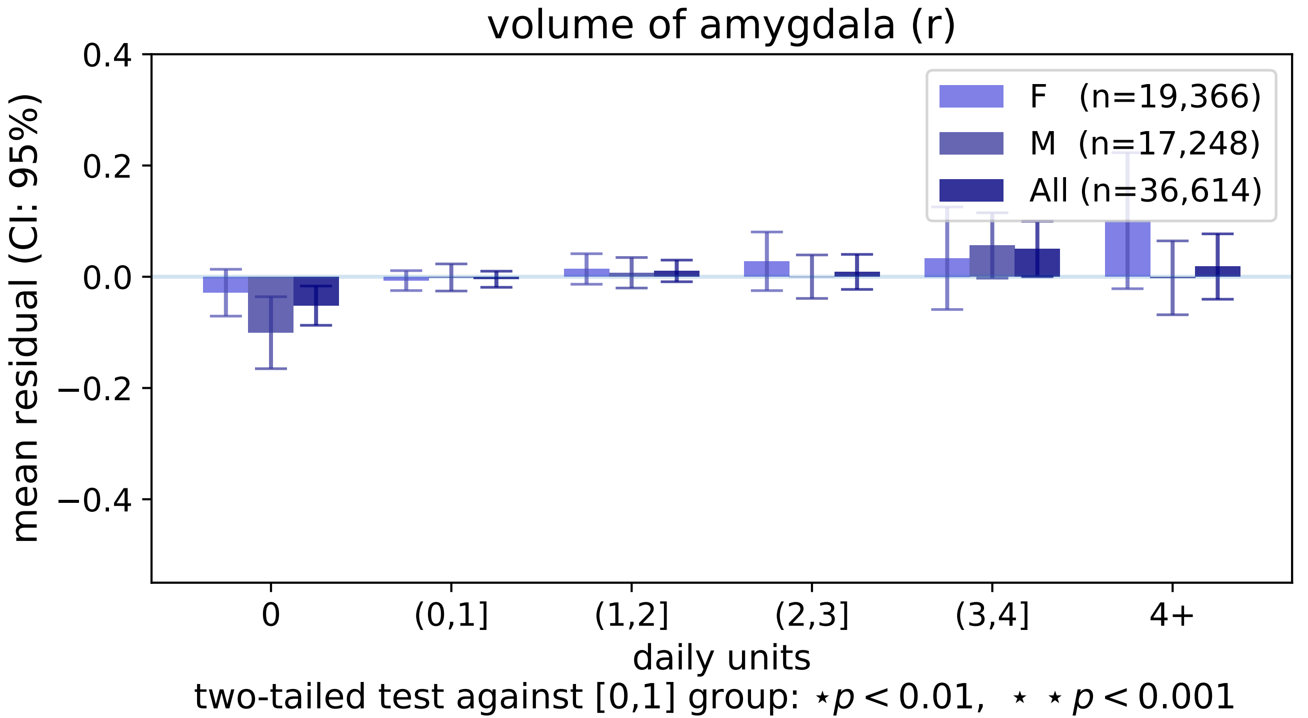
volume of amygdala (r)
0.4
0.2
0.0
−0.2
−0.4
mean residual (CI: 95%)
F (n=19,366)
M (n=17,248)
All (n=36,614)
0
(0,1]
(1,2]
(2,3]
(3,4]
4+
daily units
two-tailed test against [0,1] group: ⋆p < 0.01, ⋆ ⋆ p < 0.001

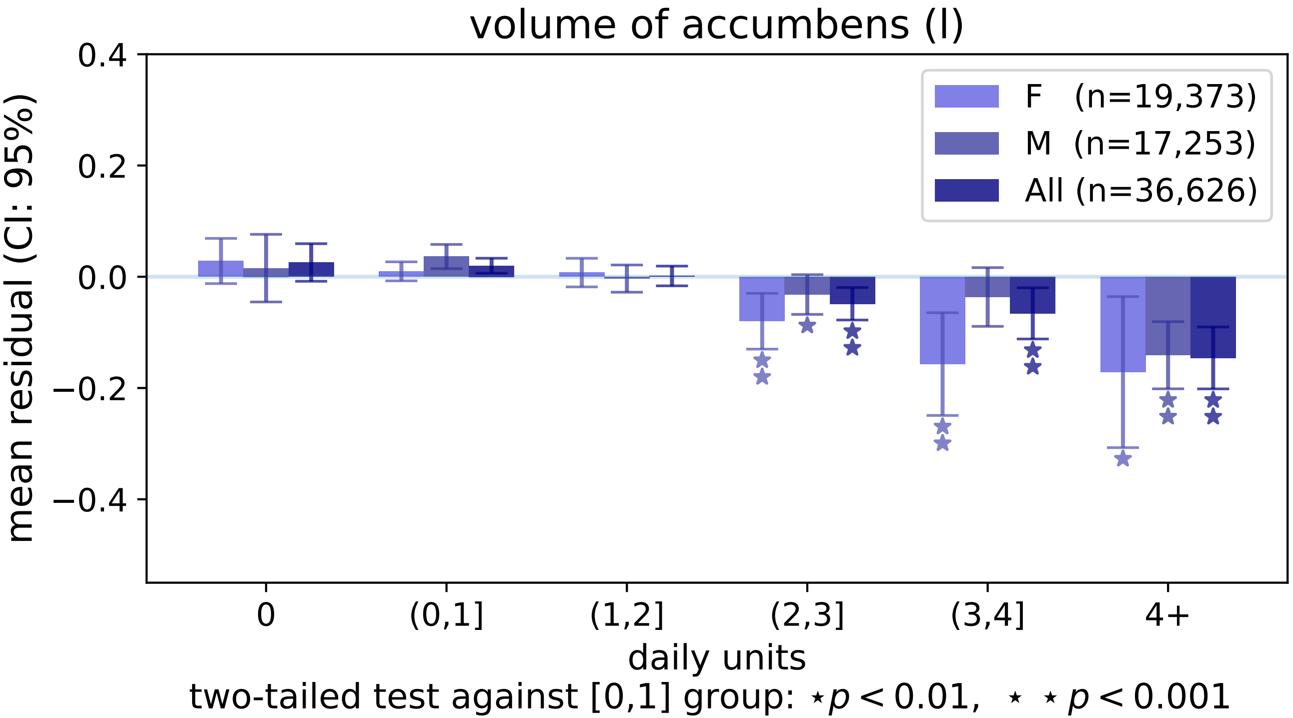
volume of accumbens (l)
F (n=19,373)
M (n=17,253)
All (n=36,626)
mean residual (CI: 95%)
0.4
0.2
0.0
−0.2
−0.4
0
(0,1]
(1,2]
(2,3]
(3,4]
4+
daily units
two-tailed test against [0,1] group: ⋆p < 0.01, ⋆ ⋆ p < 0.001

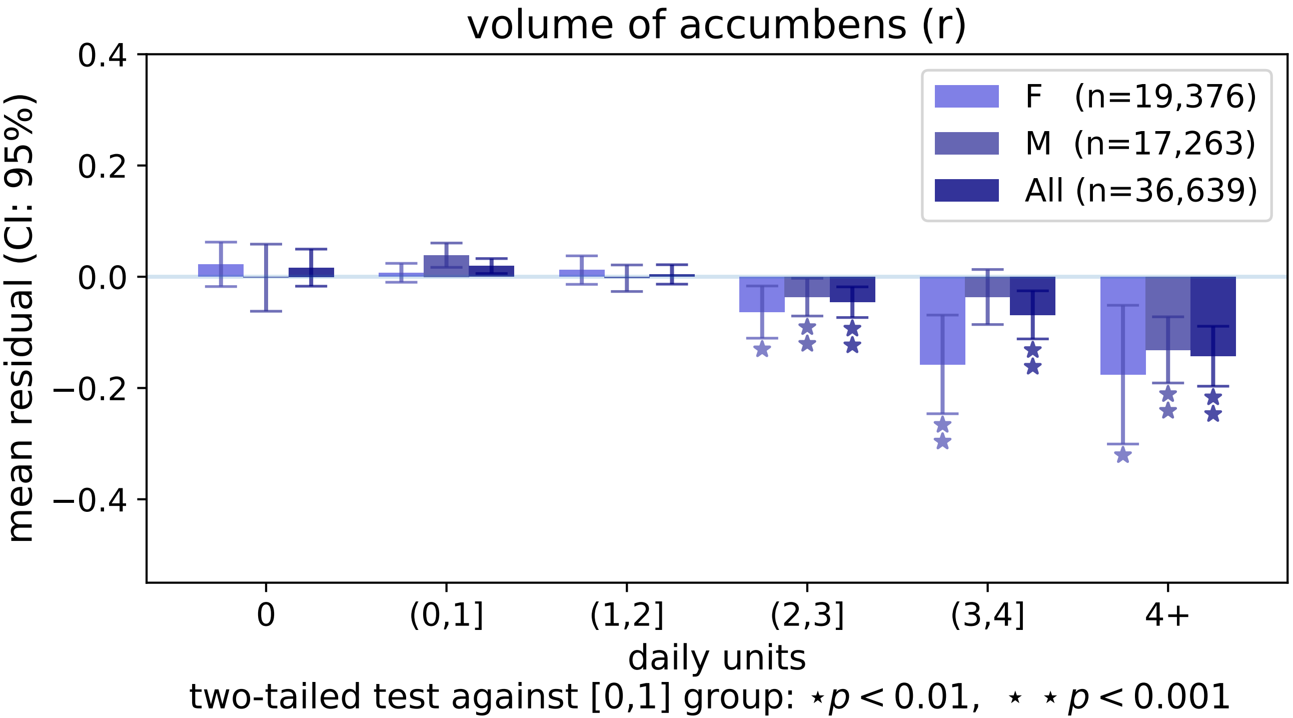
volume of accumbens (r)
mean residual (CI: 95%)
0.4
0.2
0.0
−0.2
−0.4
F (n=19,376)
M (n=17,263)
All (n=36,639)
0
(0,1]
(1,2]
(2,3]
(3,4]
4+
daily units
two-tailed test against [0,1] group: ⋆$p < 0.01$, ⋆ ⋆ $p < 0.001$

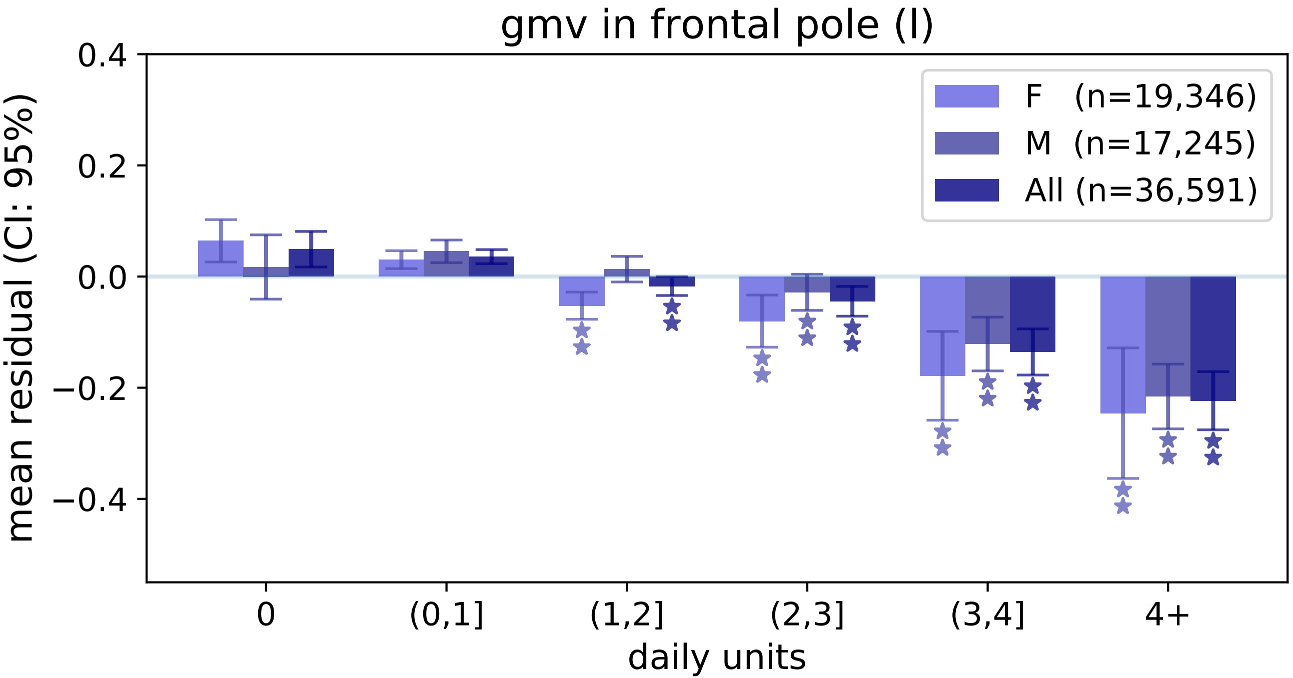

two-tailed test against [0,1] group: ⋆$p < 0.01$, ⋆ ⋆$p < 0.001$

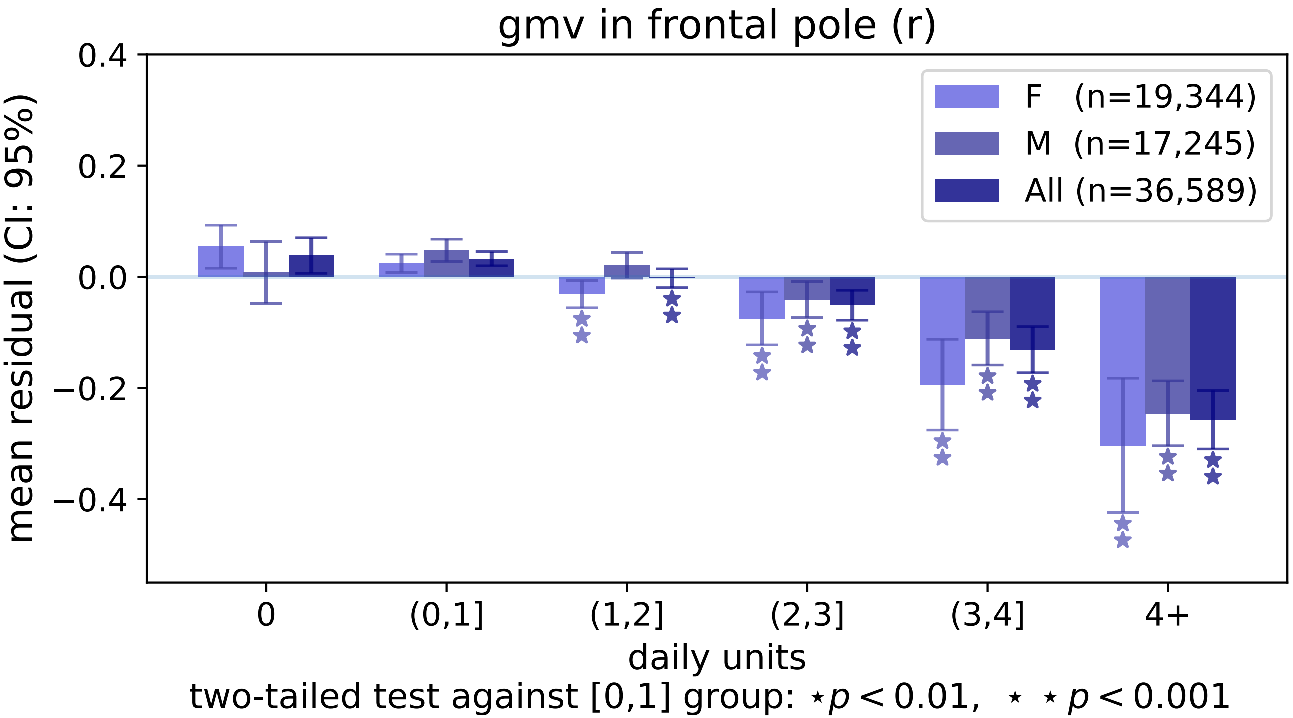
gmv in frontal pole (r)
mean residual (CI: 95%)
0.4
0.2
0.0
−0.2
−0.4
F (n=19,344)
M (n=17,245)
All (n=36,589)
0
(0,1]
(1,2]
(2,3]
(3,4]
4+
daily units
two-tailed test against [0,1] group: ⋆ p < 0.01, ⋆ ⋆ p < 0.001

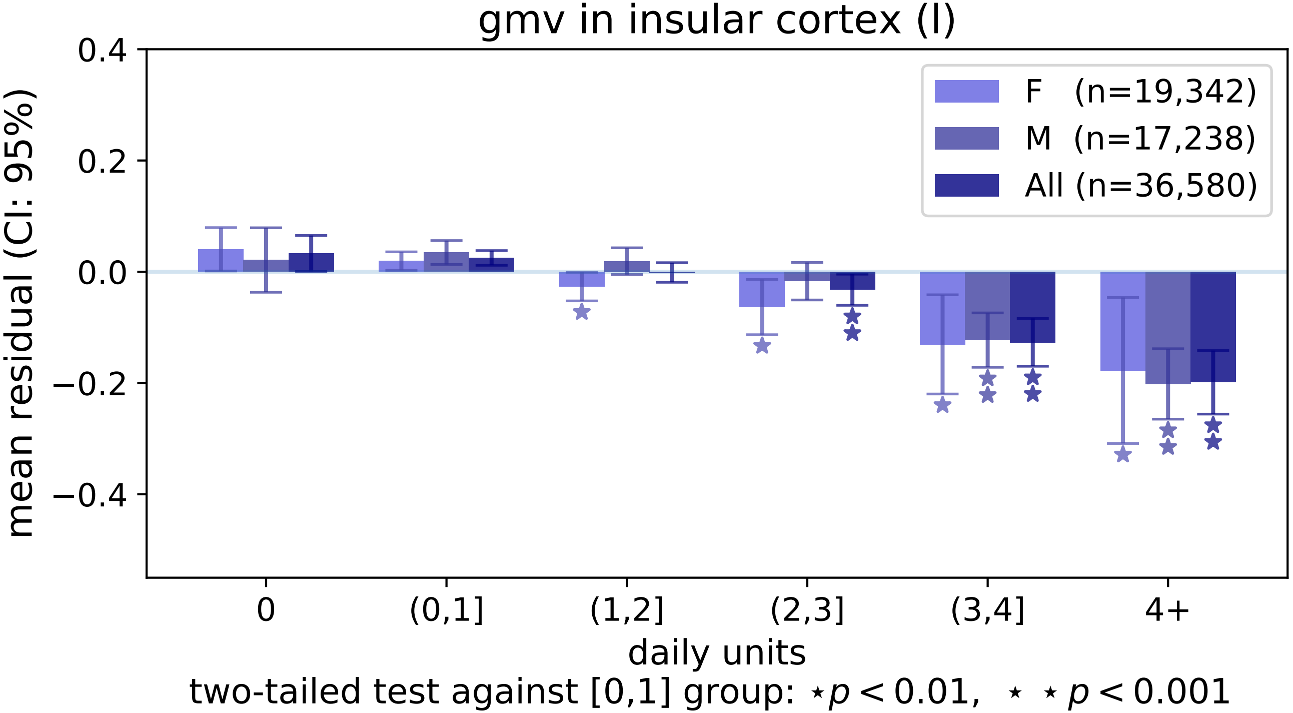
gmv in insular cortex (l)
mean residual (CI: 95%)
0.4
0.2
0.0
−0.2
−0.4
F (n=19,342)
M (n=17,238)
All (n=36,580)
0
(0,1]
(1,2]
(2,3]
(3,4]
4+
daily units
two-tailed test against [0,1] group: ⋆$p < 0.01$, ⋆ ⋆ $p < 0.001$

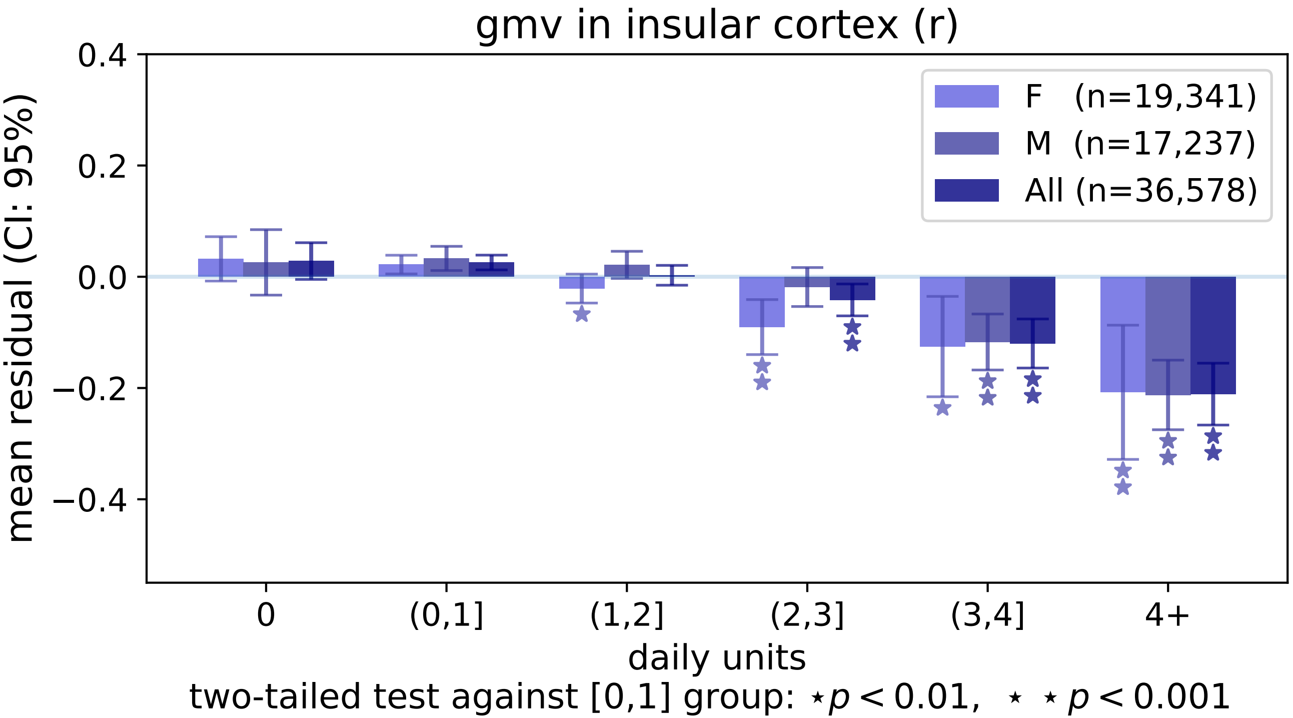
gmv in insular cortex (r)
mean residual (CI: 95%)
0.4
0.2
0.0
−0.2
−0.4
F (n=19,341)
M (n=17,237)
All (n=36,578)
0
(0,1]
(1,2]
(2,3]
(3,4]
4+
daily units
two-tailed test against [0,1] group: ⋆p < 0.01, ⋆ ⋆ p < 0.001

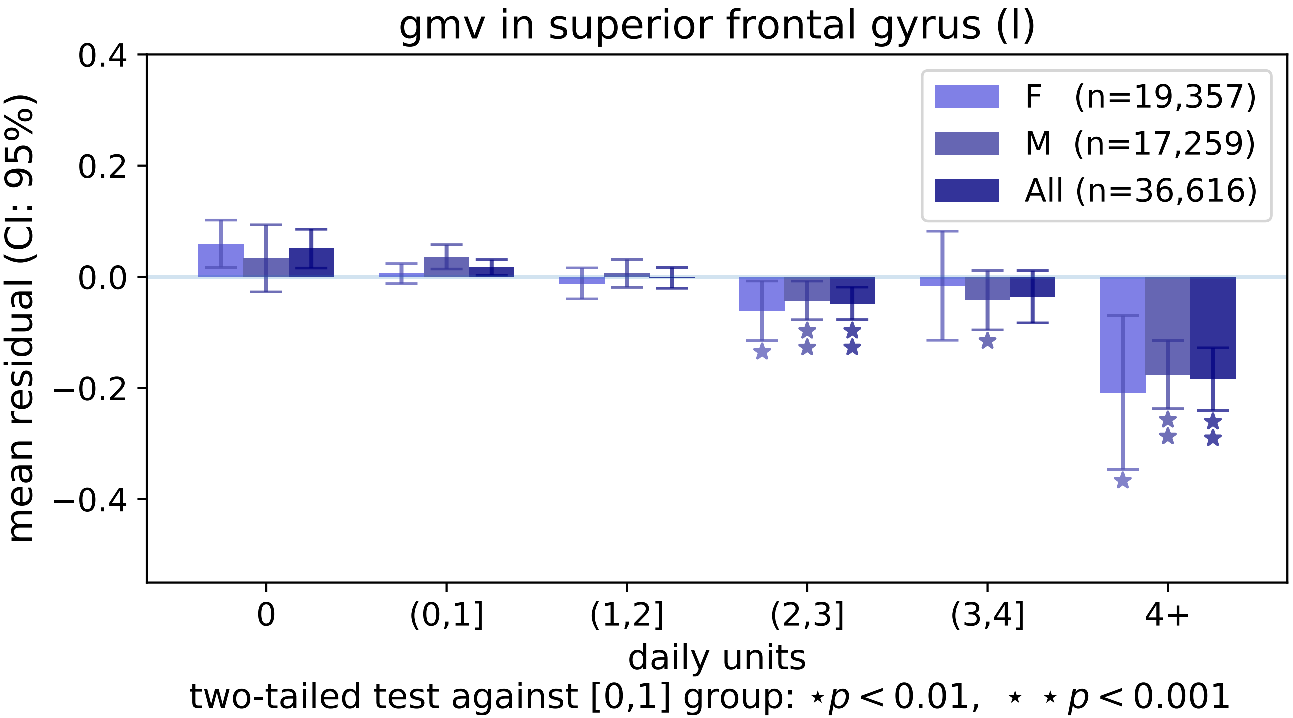
gmv in superior frontal gyrus (l)
mean residual (CI: 95%)
0.4
0.2
0.0
−0.2
−0.4
F (n=19,357)
M (n=17,259)
All (n=36,616)
0
(0,1]
(1,2]
(2,3]
(3,4]
4+
daily units
two-tailed test against [0,1] group: ⋆p < 0.01, ⋆ ⋆ p < 0.001

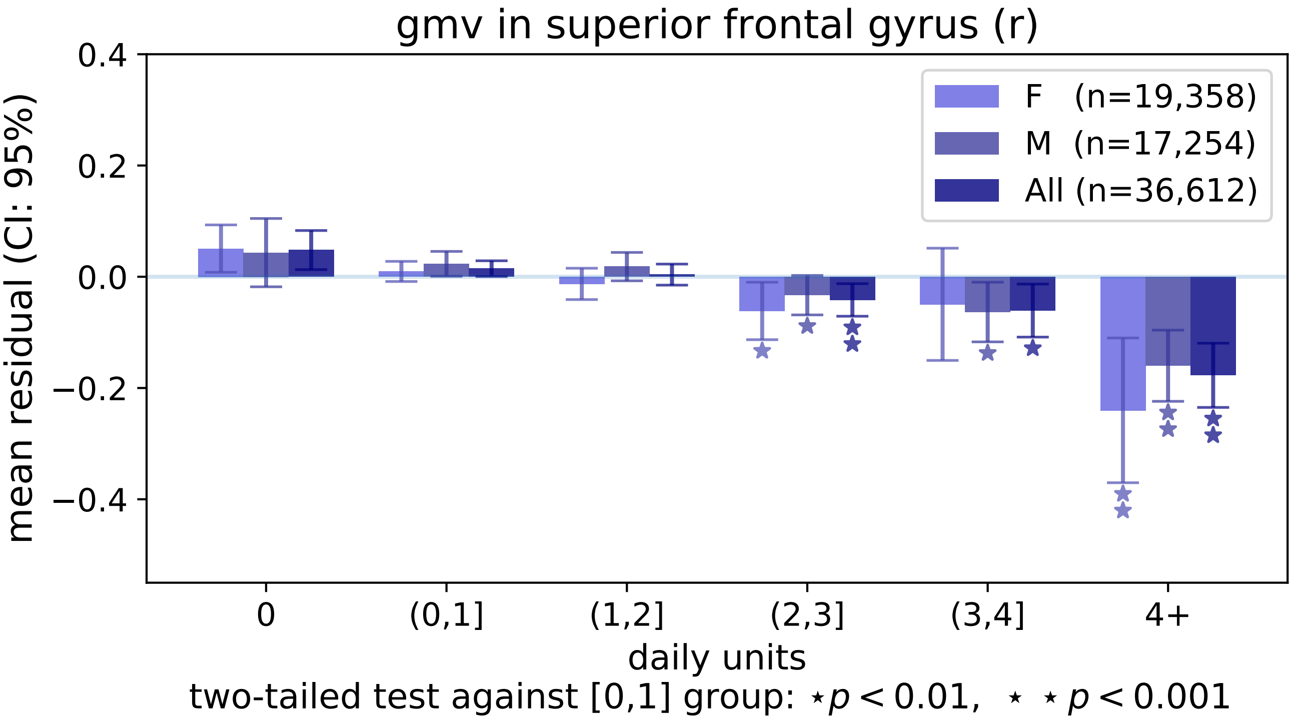
gmv in superior frontal gyrus (r)
mean residual (CI: 95%)
0.4
0.2
0.0
−0.2
−0.4
F (n=19,358)
M (n=17,254)
All (n=36,612)
0
(0,1]
(1,2]
(2,3]
(3,4]
4+
daily units
two-tailed test against [0,1] group: ⋆p < 0.01, ⋆ ⋆ p < 0.001

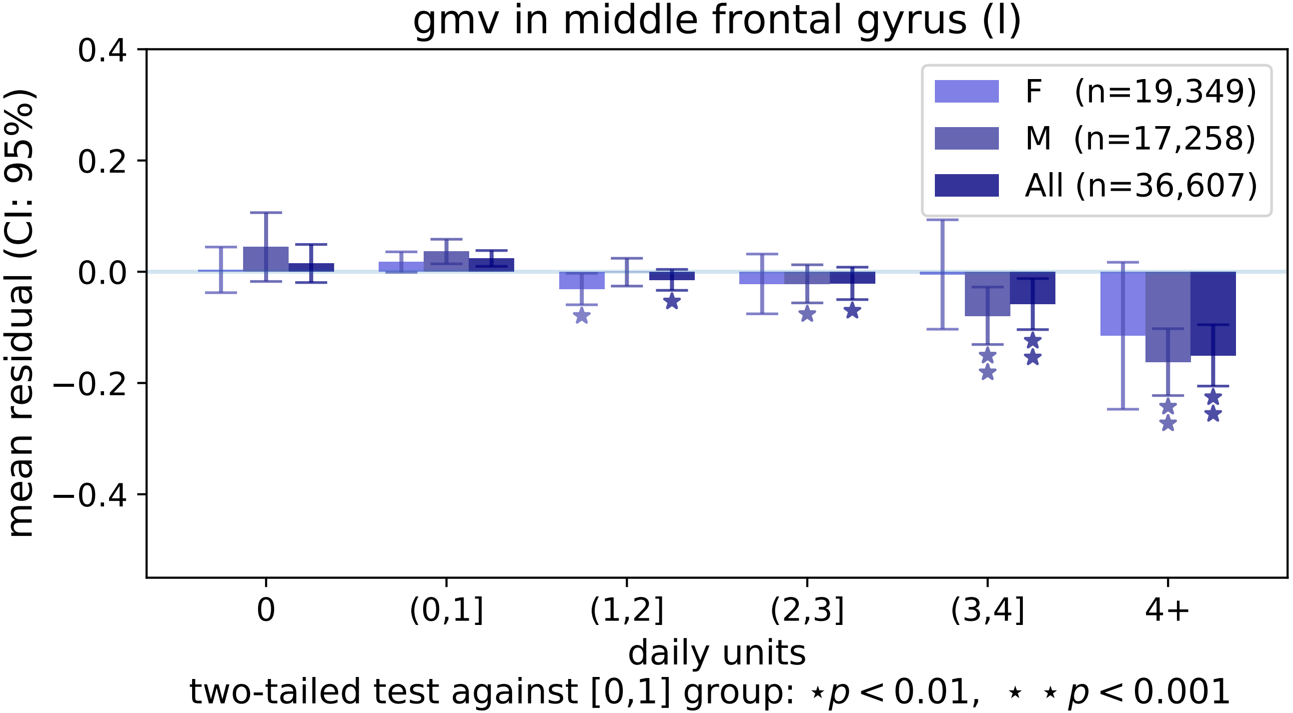
gmv in middle frontal gyrus (l)
F (n=19,349)
M (n=17,258)
All (n=36,607)
mean residual (CI: 95%)
0.4
0.2
0.0
−0.2
−0.4
0
(0,1]
(1,2]
(2,3]
(3,4]
4+
daily units
two-tailed test against [0,1] group: ⋆ p < 0.01, ⋆ ⋆ p < 0.001

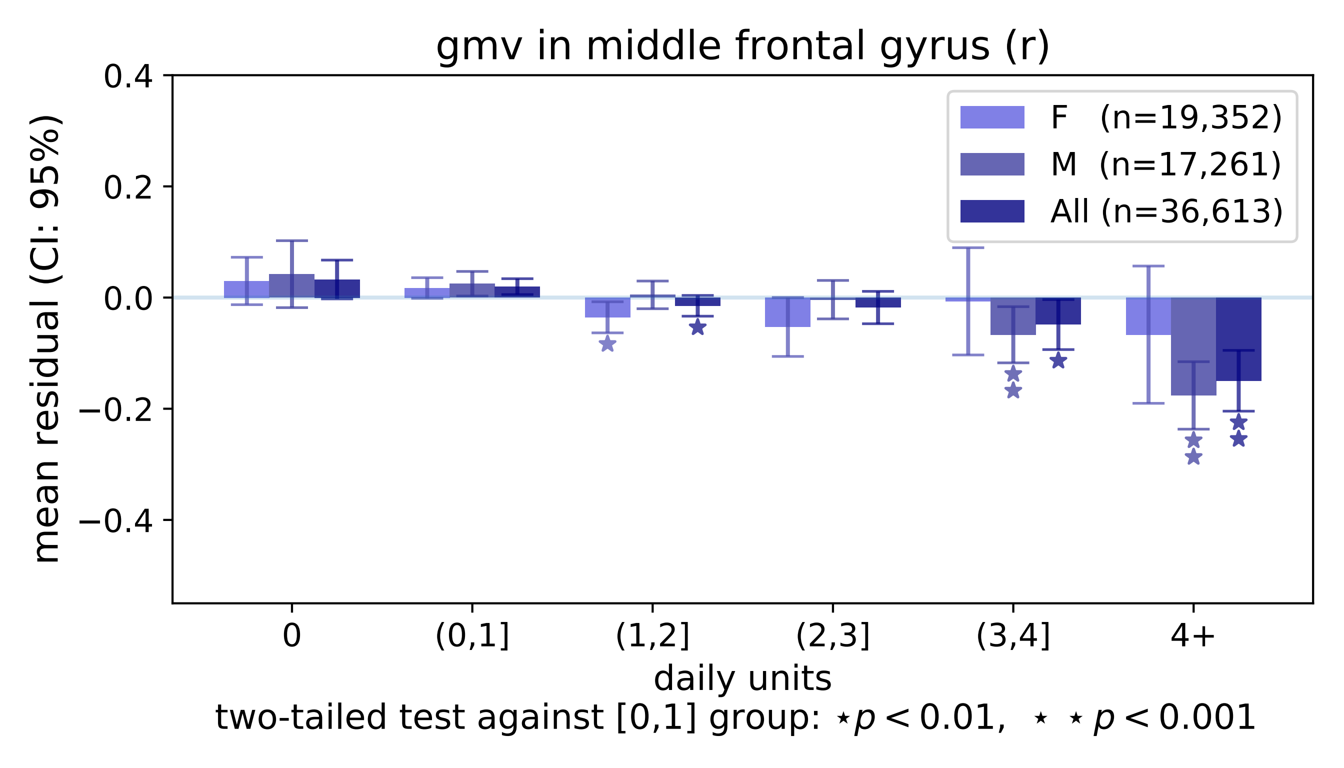

gmv in middle frontal gyrus (r)
0.4
0.2
0.0
−0.2
−0.4
mean residual (CI: 95%)
F (n=19,352)
M (n=17,261)
All (n=36,613)
0
(0,1]
(1,2]
(2,3]
(3,4]
4+
daily units
two-tailed test against [0,1] group: ⋆ p < 0.01, ⋆ ⋆ p < 0.001

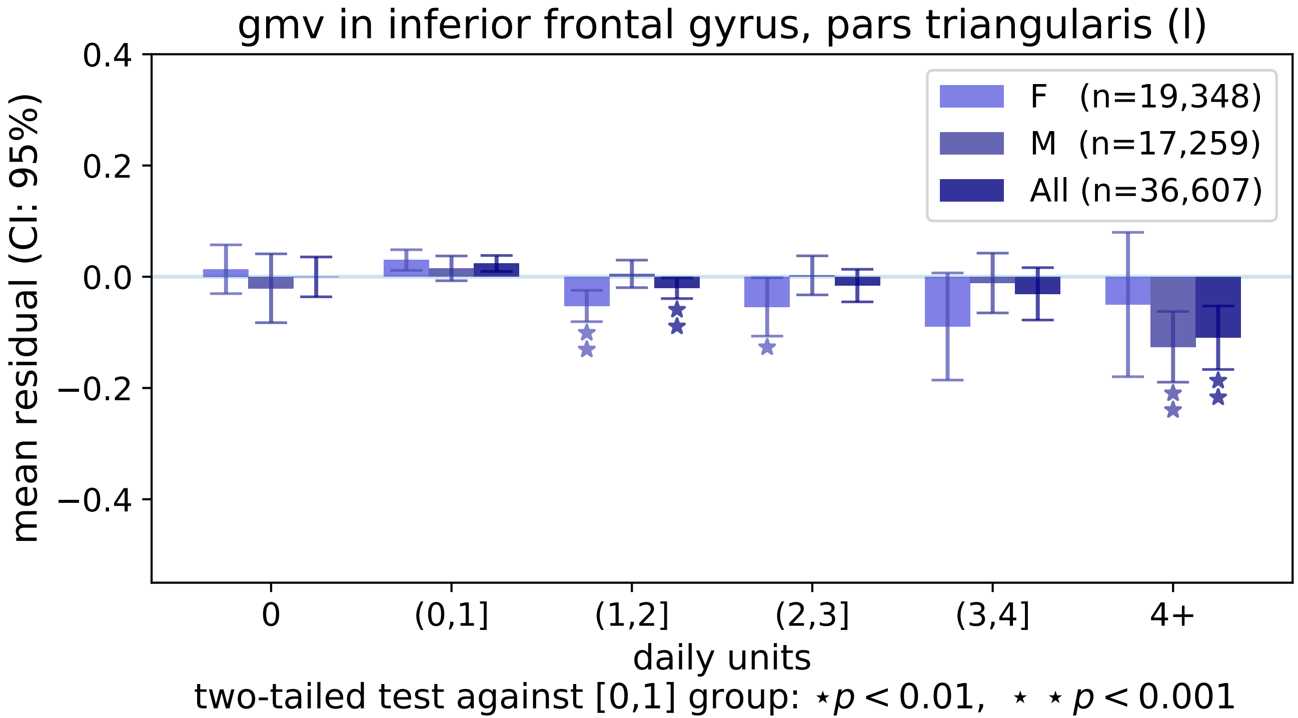

gmv in inferior frontal gyrus, pars triangularis (l)
mean residual (CI: 95%)
0.4
0.2
0.0
−0.2
−0.4
F (n=19,348)
M (n=17,259)
All (n=36,607)
0
(0,1]
(1,2]
(2,3]
(3,4]
4+
daily units
two-tailed test against [0,1] group: ⋆p < 0.01, ⋆ ⋆ p < 0.001

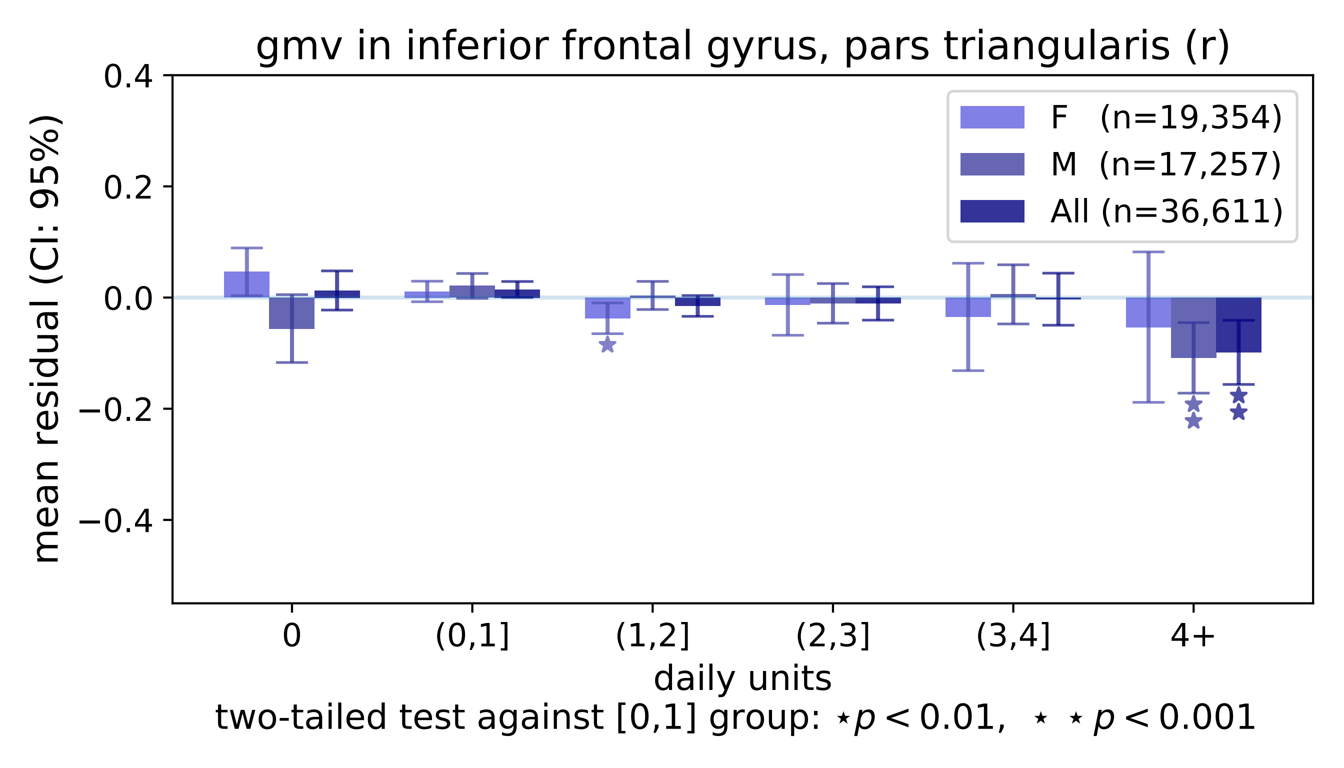
gmv in inferior frontal gyrus, pars triangularis (r)
mean residual (CI: 95%)
0.4
0.2
0.0
−0.2
−0.4
F (n=19,354)
M (n=17,257)
All (n=36,611)
0
(0,1]
(1,2]
(2,3]
(3,4]
4+
daily units
two-tailed test against [0,1] group: ⋆p < 0.01, ⋆ ⋆ p < 0.001

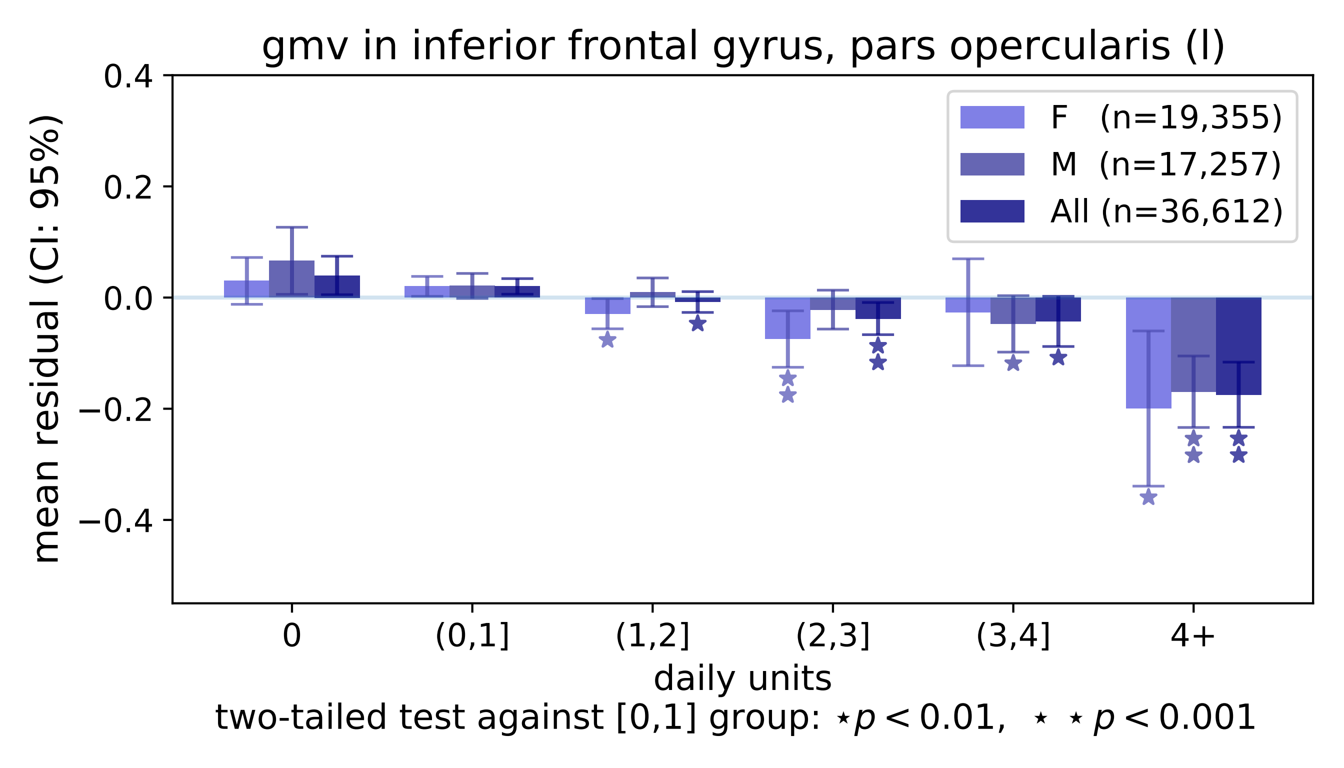
gmv in inferior frontal gyrus, pars opercularis (l)
mean residual (CI: 95%)
0.4
0.2
0.0
−0.2
−0.4
F (n=19,355)
M (n=17,257)
All (n=36,612)
0
(0,1]
(1,2]
(2,3]
(3,4]
4+
daily units
two-tailed test against [0,1] group: ⋆p < 0.01, ⋆ ⋆ p < 0.001

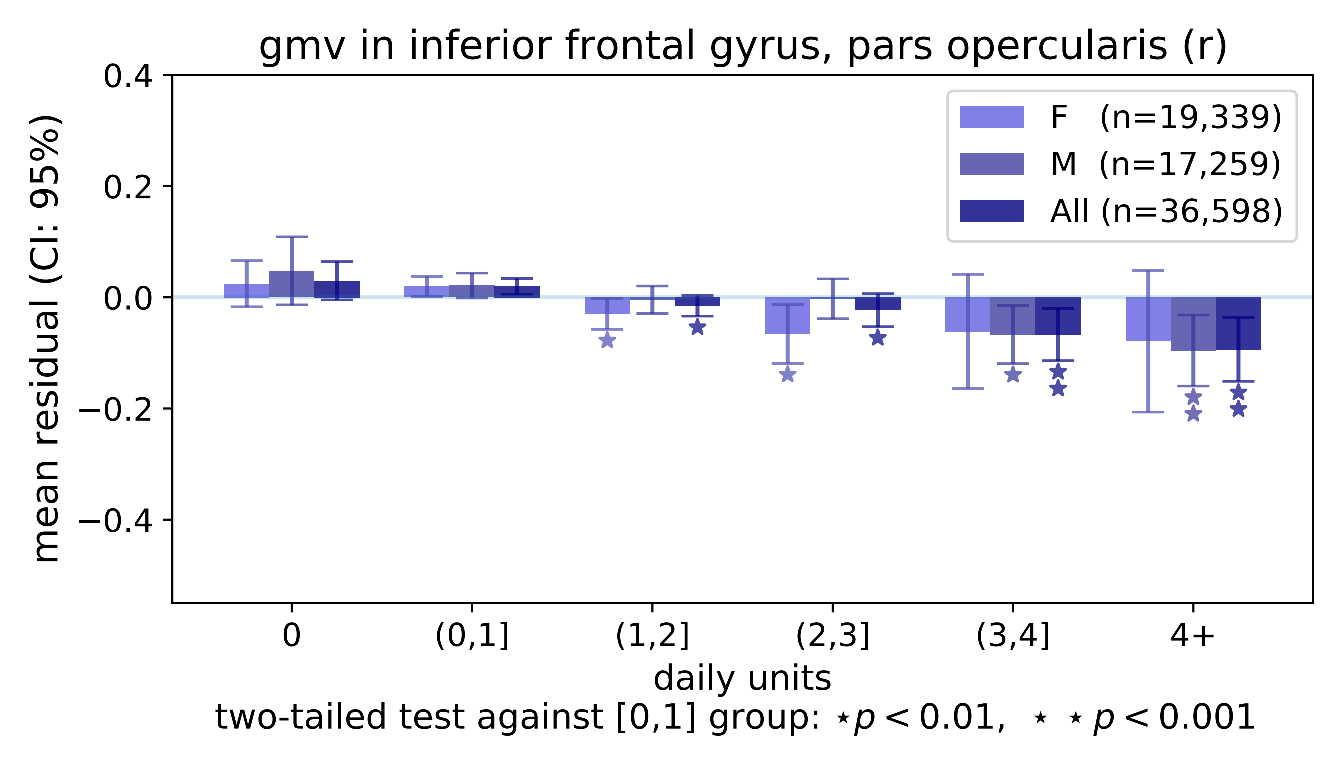
gmv in inferior frontal gyrus, pars opercularis (r)
mean residual (CI: 95%)
0.4
0.2
0.0
−0.2
−0.4
F (n=19,339)
M (n=17,259)
All (n=36,598)
0
(0,1]
(1,2]
(2,3]
(3,4]
4+
daily units
two-tailed test against [0,1] group: ⋆p < 0.01, ⋆ ⋆ p < 0.001

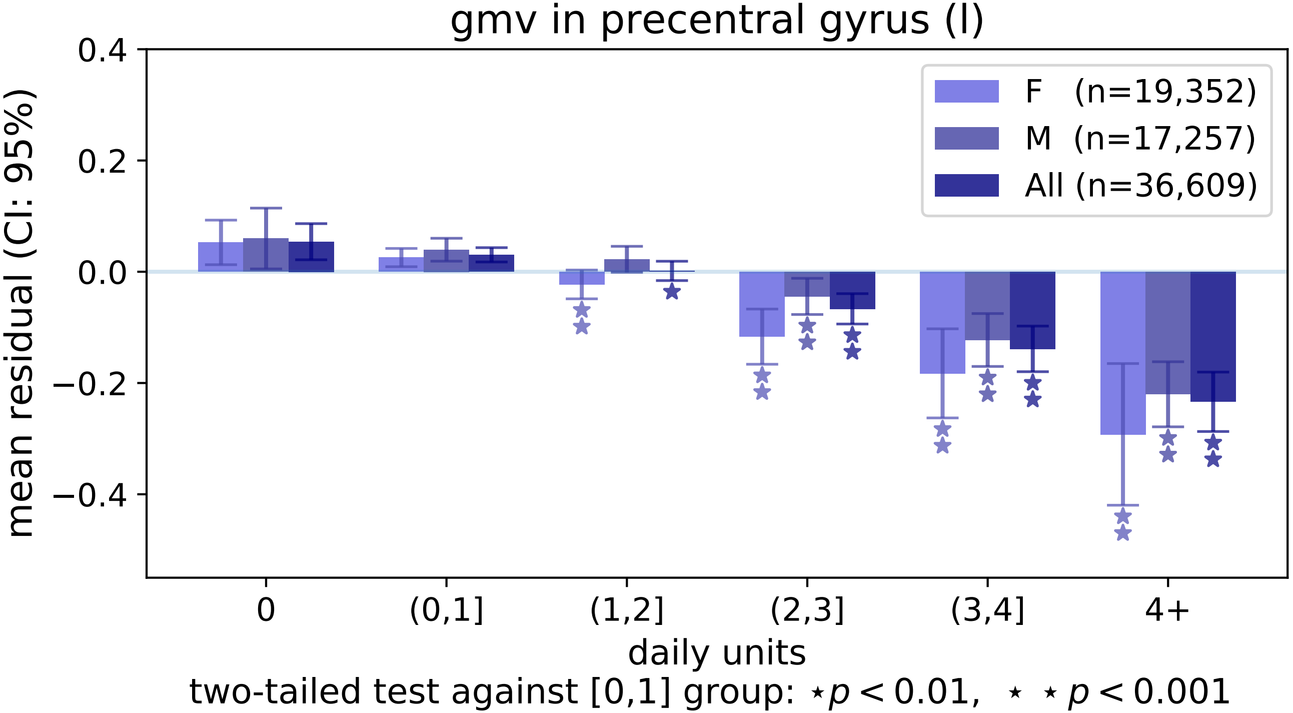

gmv in precentral gyrus (l)
0.4
0.2
0.0
−0.2
−0.4
mean residual (CI: 95%)
F (n=19,352)
M (n=17,257)
All (n=36,609)
0
(0,1]
(1,2]
(2,3]
(3,4]
4+
daily units
two-tailed test against [0,1] group: ⋆p < 0.01, ⋆ ⋆ p < 0.001

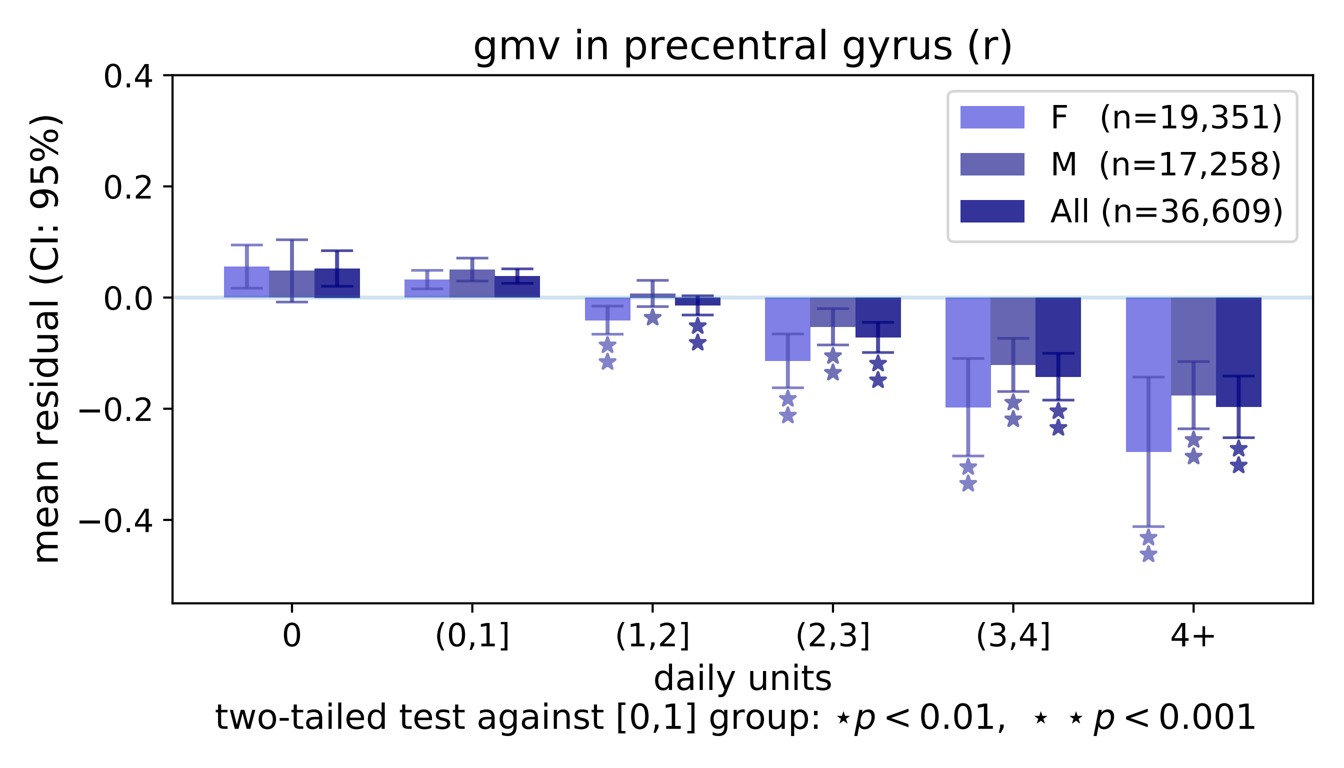
gmv in precentral gyrus (r)
mean residual (CI: 95%)
0.4
0.2
0.0
−0.2
−0.4
F (n=19,351)
M (n=17,258)
All (n=36,609)
0
(0,1]
(1,2]
(2,3]
(3,4]
4+
daily units
two-tailed test against [0,1] group: ⋆p < 0.01, ⋆ ⋆ p < 0.001

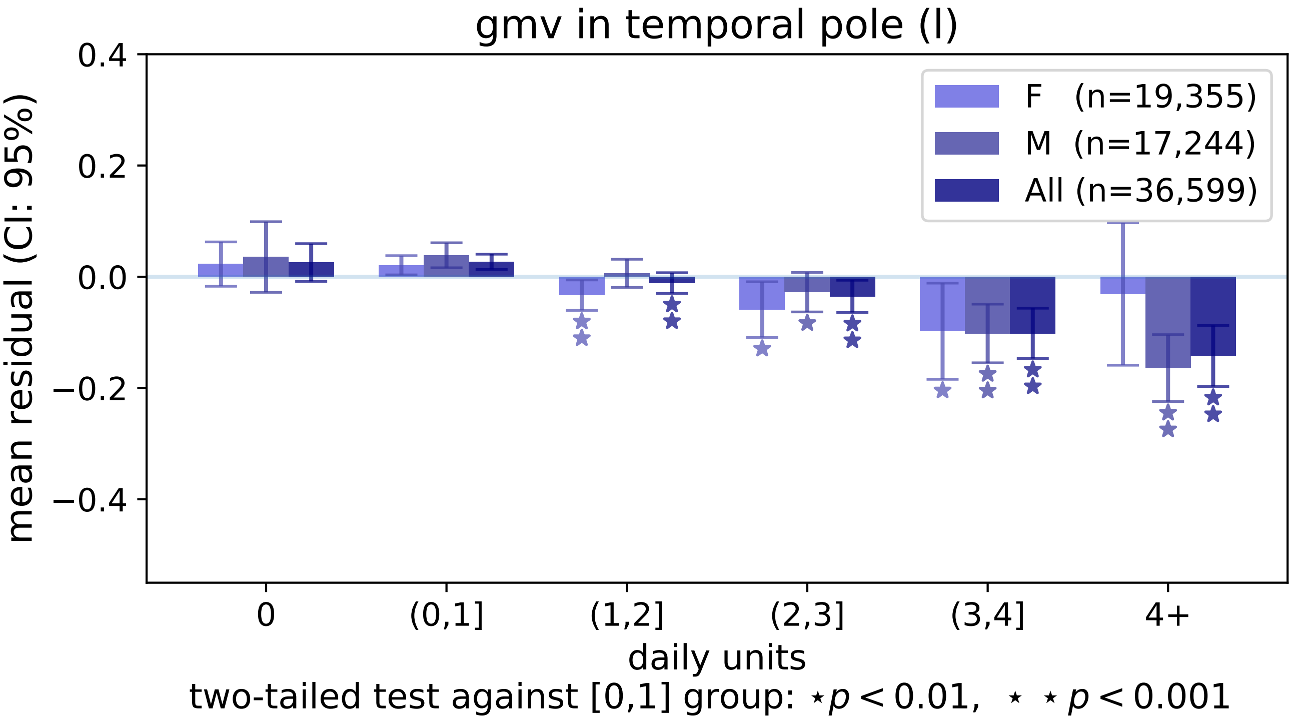
gmv in temporal pole (l)
mean residual (CI: 95%)
0.4
0.2
0.0
−0.2
−0.4
F (n=19,355)
M (n=17,244)
All (n=36,599)
0
(0,1]
(1,2]
(2,3]
(3,4]
4+
daily units
two-tailed test against [0,1] group: ⋆p < 0.01, ⋆ ⋆ p < 0.001

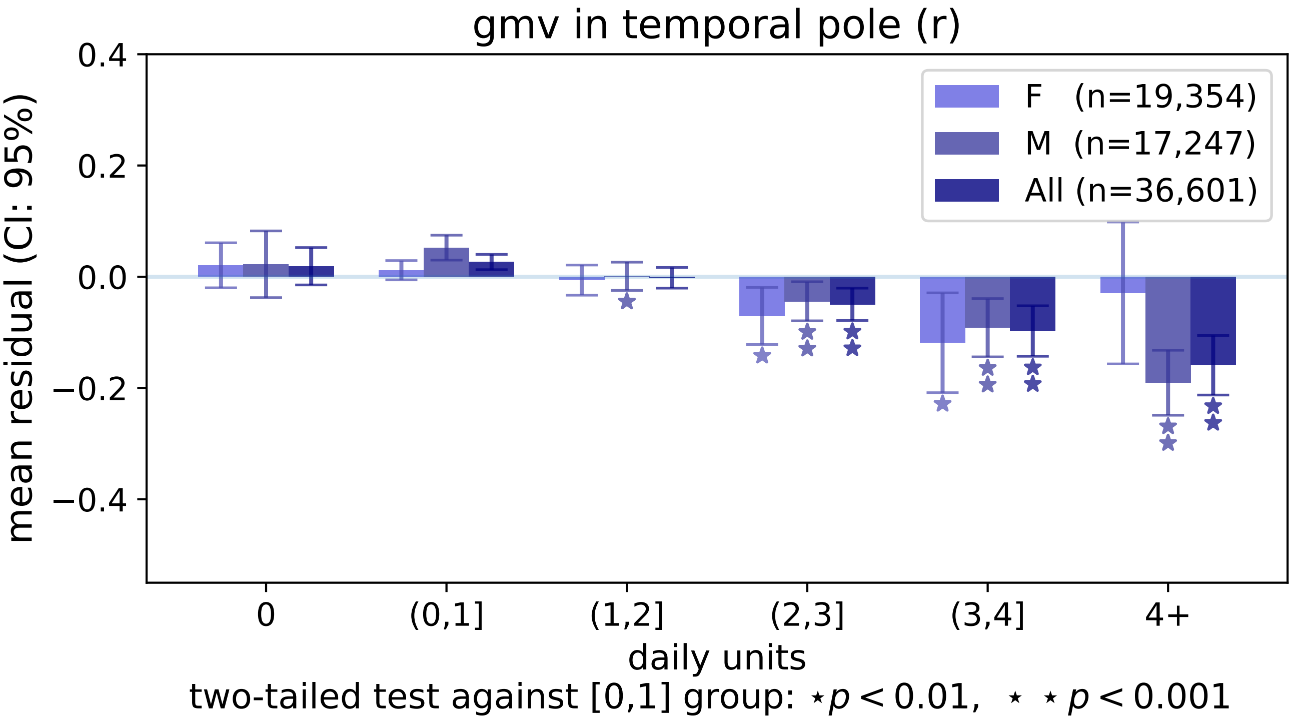

gmv in temporal pole (r)
0.4
0.2
0.0
−0.2
−0.4
mean residual (CI: 95%)
F (n=19,354)
M (n=17,247)
All (n=36,601)
0
(0,1]
(1,2]
(2,3]
(3,4]
4+
daily units
two-tailed test against [0,1] group: ⋆p < 0.01, ⋆ ⋆ p < 0.001

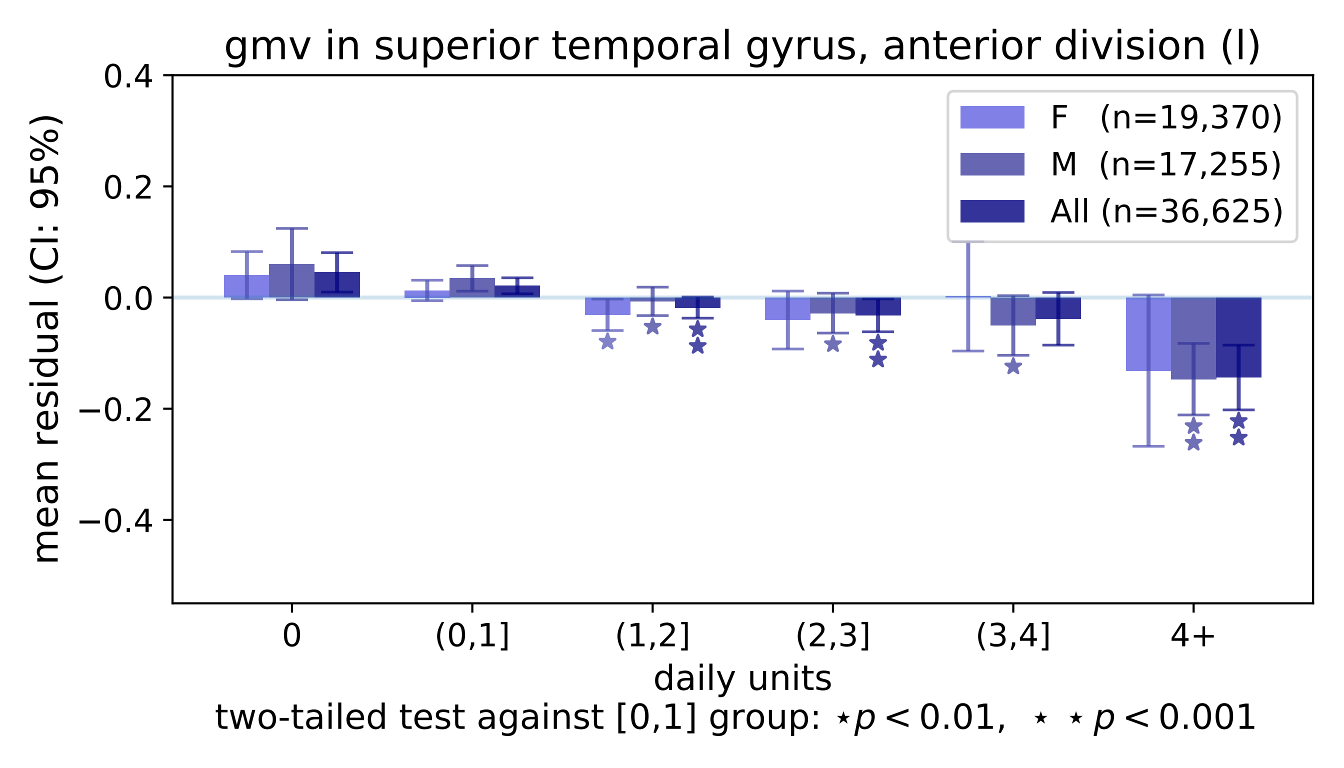
gmv in superior temporal gyrus, anterior division (l)
mean residual (CI: 95%)
0.4
0.2
0.0
−0.2
−0.4
F (n=19,370)
M (n=17,255)
All (n=36,625)
0
(0,1]
(1,2]
(2,3]
(3,4]
4+
daily units
two-tailed test against [0,1] group: ⋆ $p < 0.01$, ⋆ ⋆ $p < 0.001$

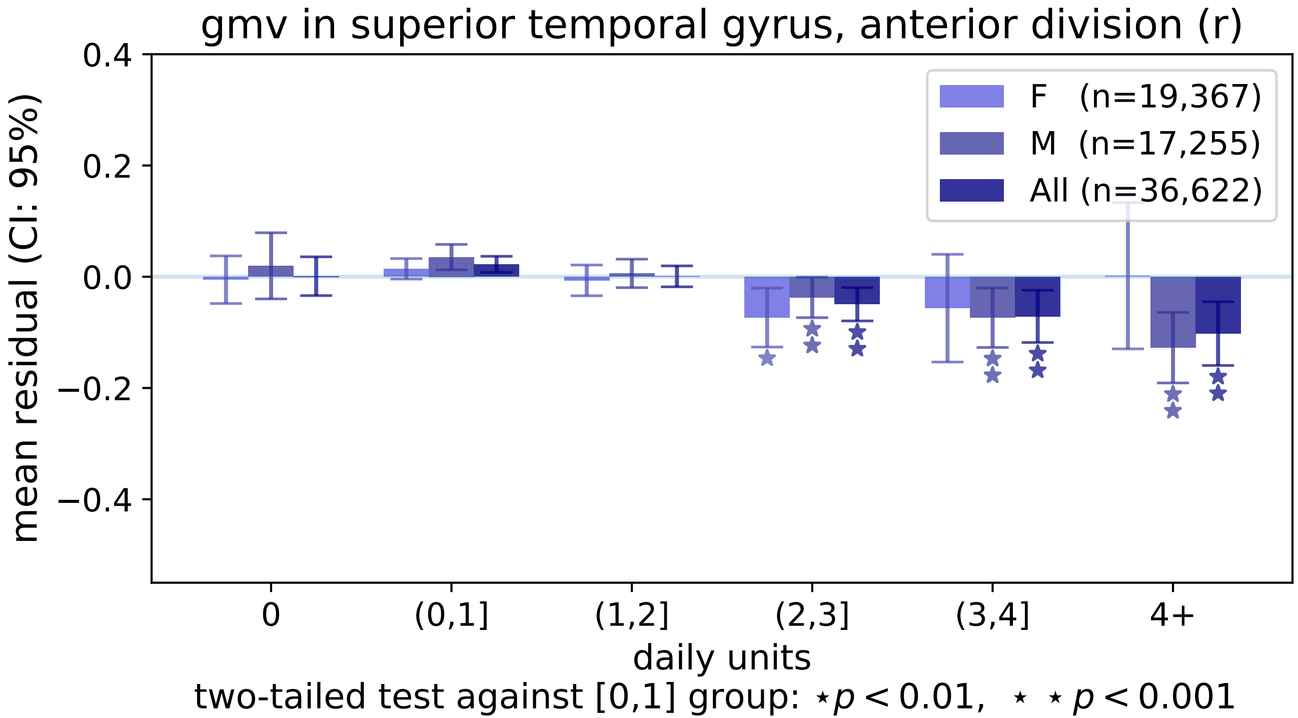

gmv in superior temporal gyrus, anterior division (r)
0.4
0.2
0.0
−0.2
−0.4
mean residual (CI: 95%)
F (n=19,367)
M (n=17,255)
All (n=36,622)
0
(0,1]
(1,2]
(2,3]
(3,4]
4+
daily units
two-tailed test against [0,1] group: ⋆p < 0.01, ⋆ ⋆ p < 0.001

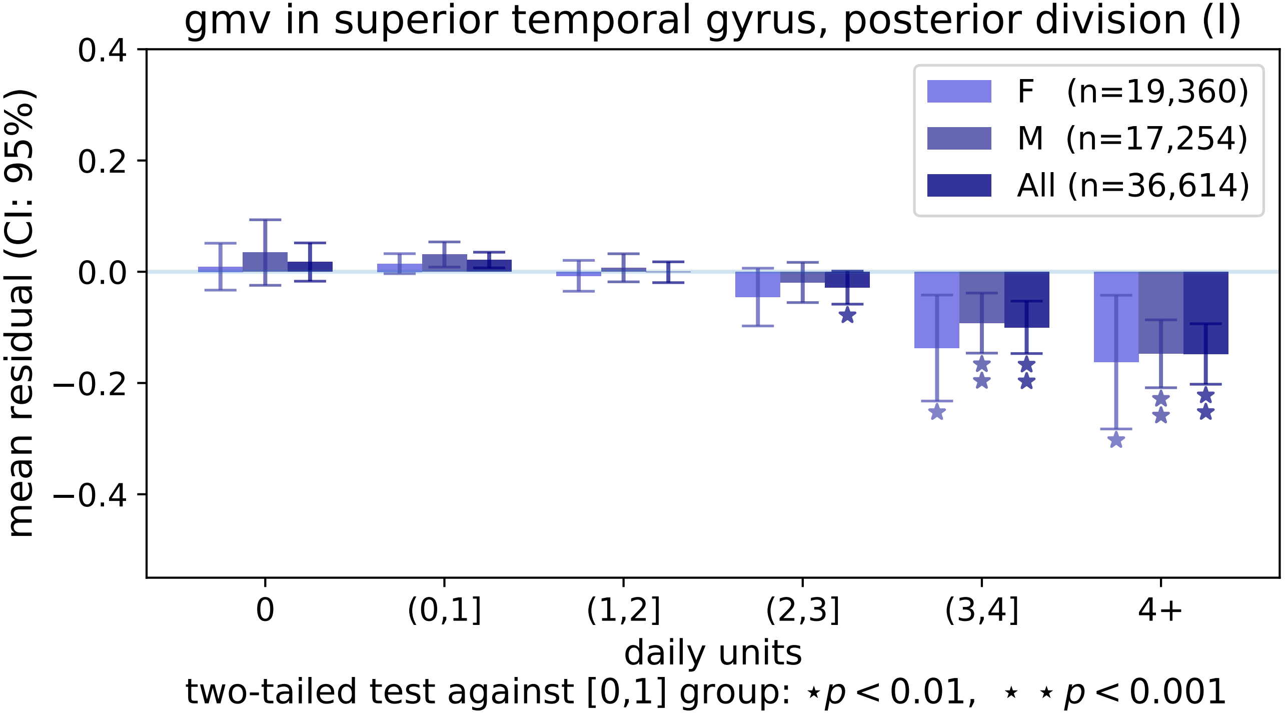
gmv in superior temporal gyrus, posterior division (l)
mean residual (CI: 95%)
0.4
0.2
0.0
−0.2
−0.4
F (n=19,360)
M (n=17,254)
All (n=36,614)
0
(0,1]
(1,2]
(2,3]
(3,4]
4+
daily units
two-tailed test against [0,1] group: ⋆p < 0.01, ⋆ ⋆ p < 0.001

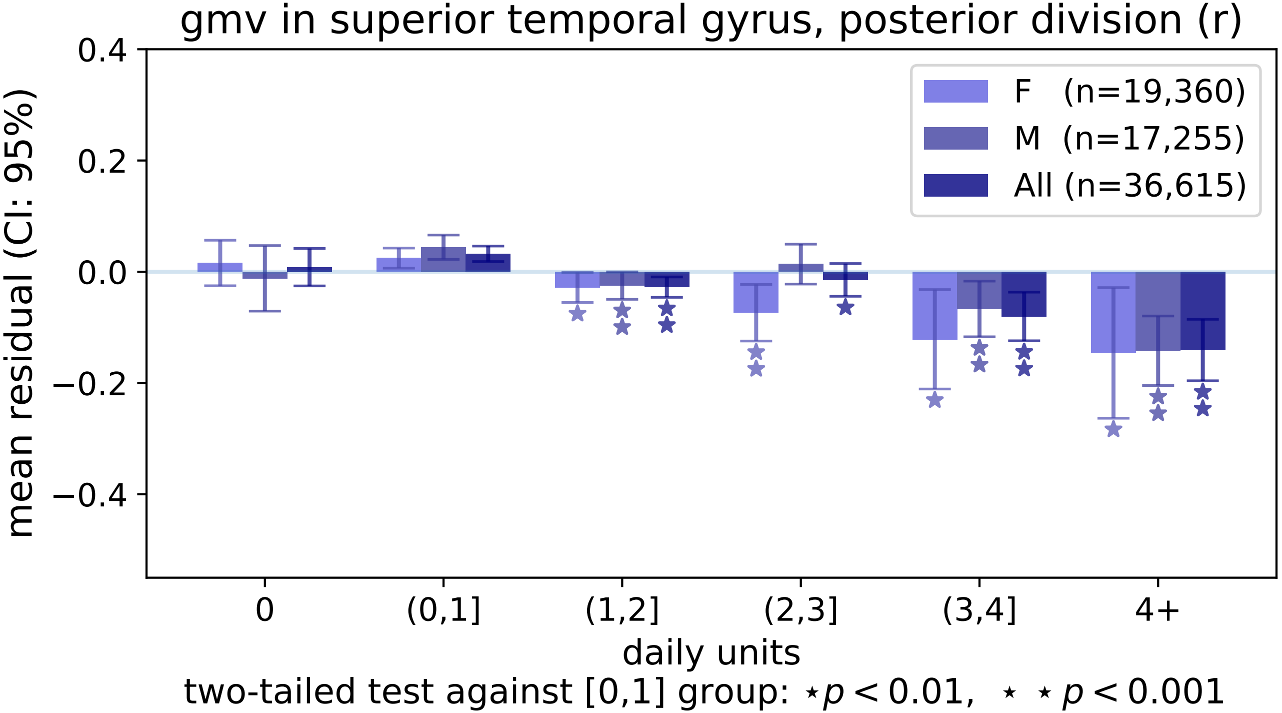
gmv in superior temporal gyrus, posterior division (r)
mean residual (CI: 95%)
0.4
0.2
0.0
−0.2
−0.4
F (n=19,360)
M (n=17,255)
All (n=36,615)
0
(0,1]
(1,2]
(2,3]
(3,4]
4+
daily units
two-tailed test against [0,1] group: ⋆p < 0.01, ⋆ ⋆ p < 0.001

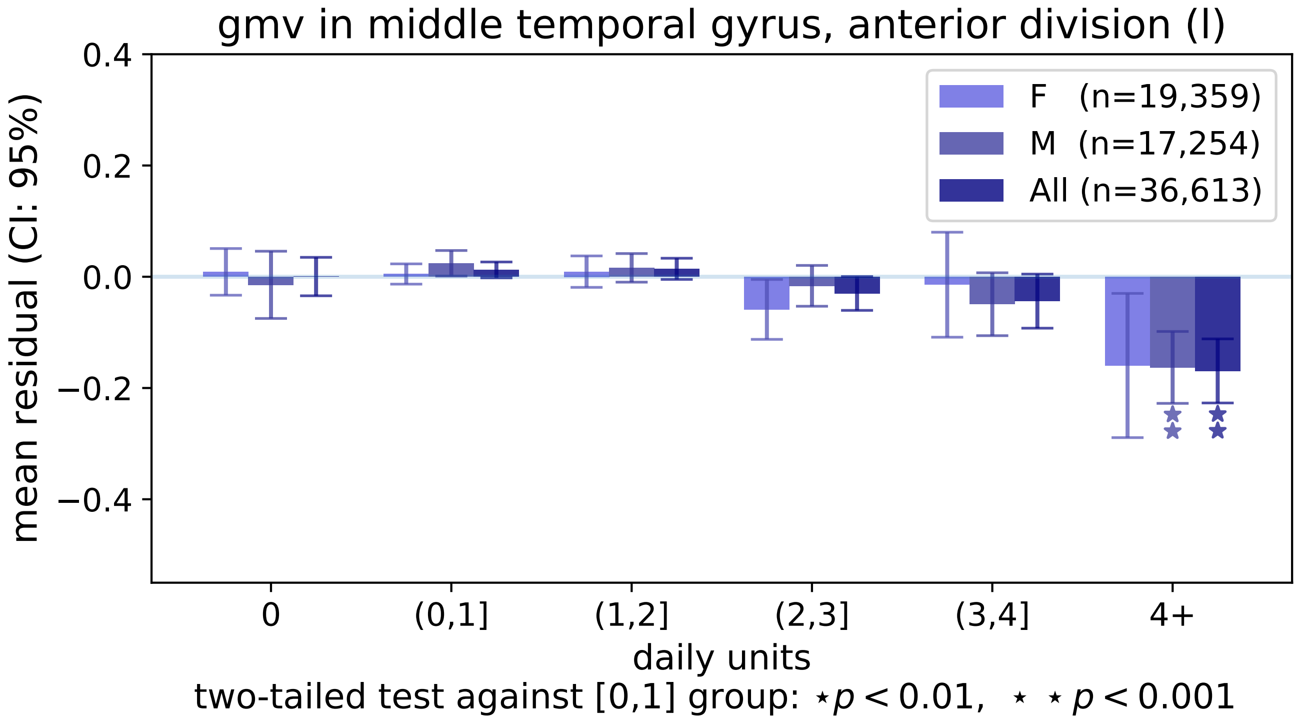
gmv in middle temporal gyrus, anterior division (l)
mean residual (CI: 95%)
0.4
0.2
0.0
−0.2
−0.4
F (n=19,359)
M (n=17,254)
All (n=36,613)
0
(0,1]
(1,2]
(2,3]
(3,4]
4+
daily units
two-tailed test against [0,1] group: ⋆p < 0.01, ⋆ ⋆ p < 0.001

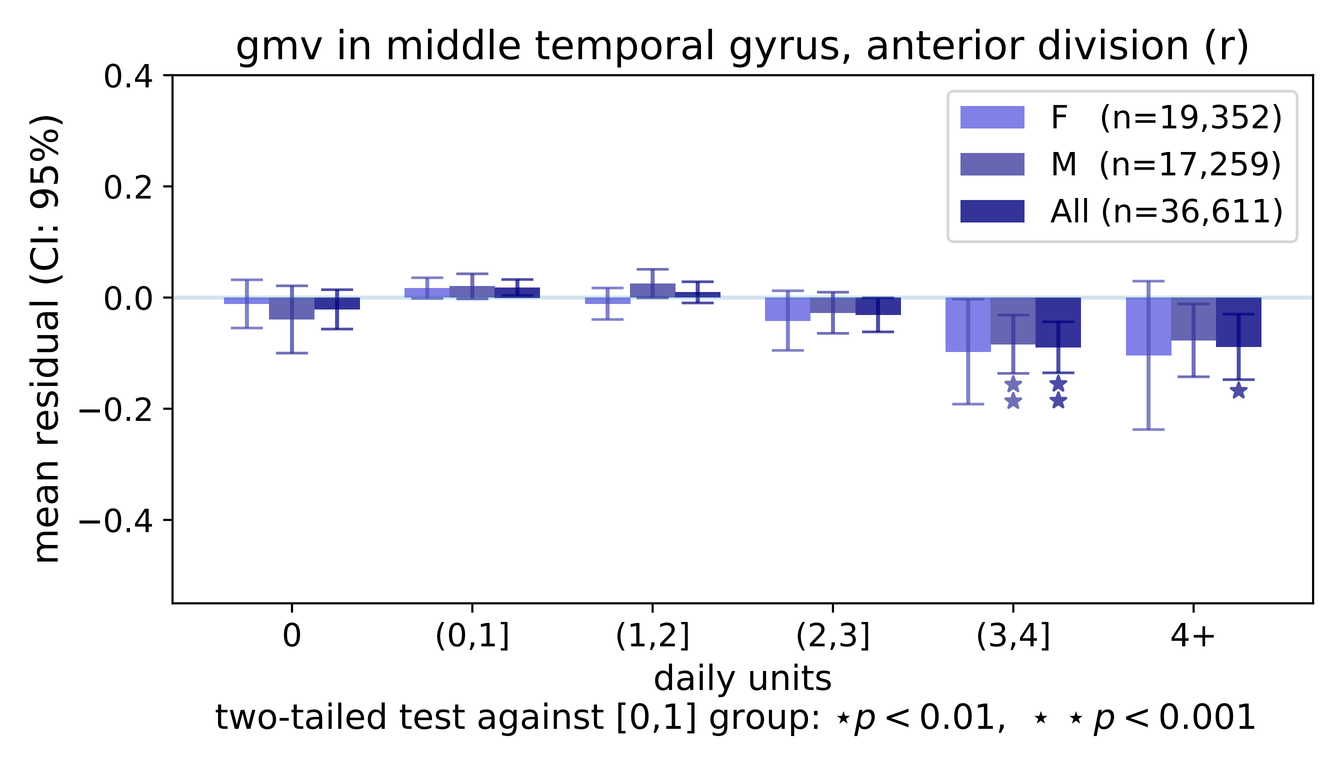
gmv in middle temporal gyrus, anterior division (r)
mean residual (CI: 95%)
0.4
0.2
0.0
−0.2
−0.4
F (n=19,352)
M (n=17,259)
All (n=36,611)
0
(0,1]
(1,2]
(2,3]
(3,4]
4+
daily units
two-tailed test against [0,1] group: ⋆p < 0.01, ⋆ ⋆ p < 0.001

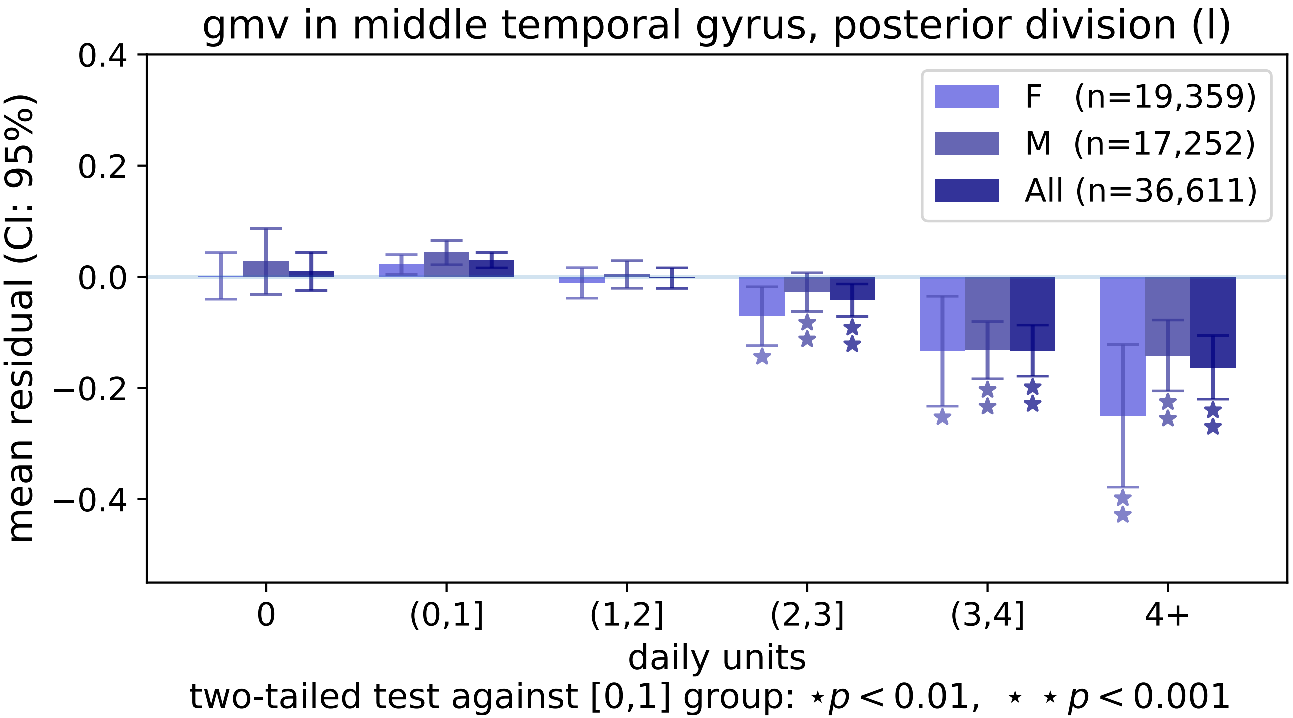

gmv in middle temporal gyrus, posterior division (l)
mean residual (CI: 95%)
0.4
0.2
0.0
−0.2
−0.4
F (n=19,359)
M (n=17,252)
All (n=36,611)
0
(0,1]
(1,2]
(2,3]
(3,4]
4+
daily units
two-tailed test against [0,1] group: ⋆p < 0.01, ⋆ ⋆ p < 0.001

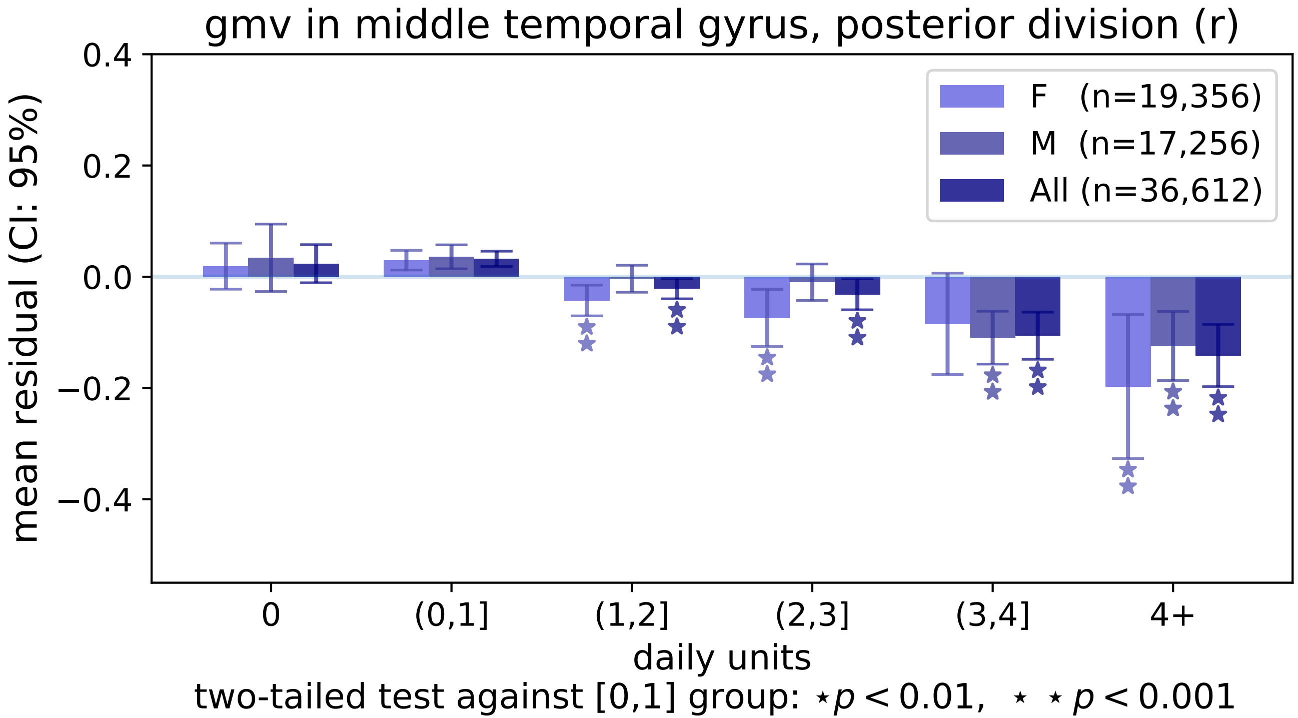
gmv in middle temporal gyrus, posterior division (r)
mean residual (CI: 95%)
0.4
0.2
0.0
−0.2
−0.4
F (n=19,356)
M (n=17,256)
All (n=36,612)
0
(0,1]
(1,2]
(2,3]
(3,4]
4+
daily units
two-tailed test against [0,1] group: ⋆p < 0.01, ⋆ ⋆ p < 0.001

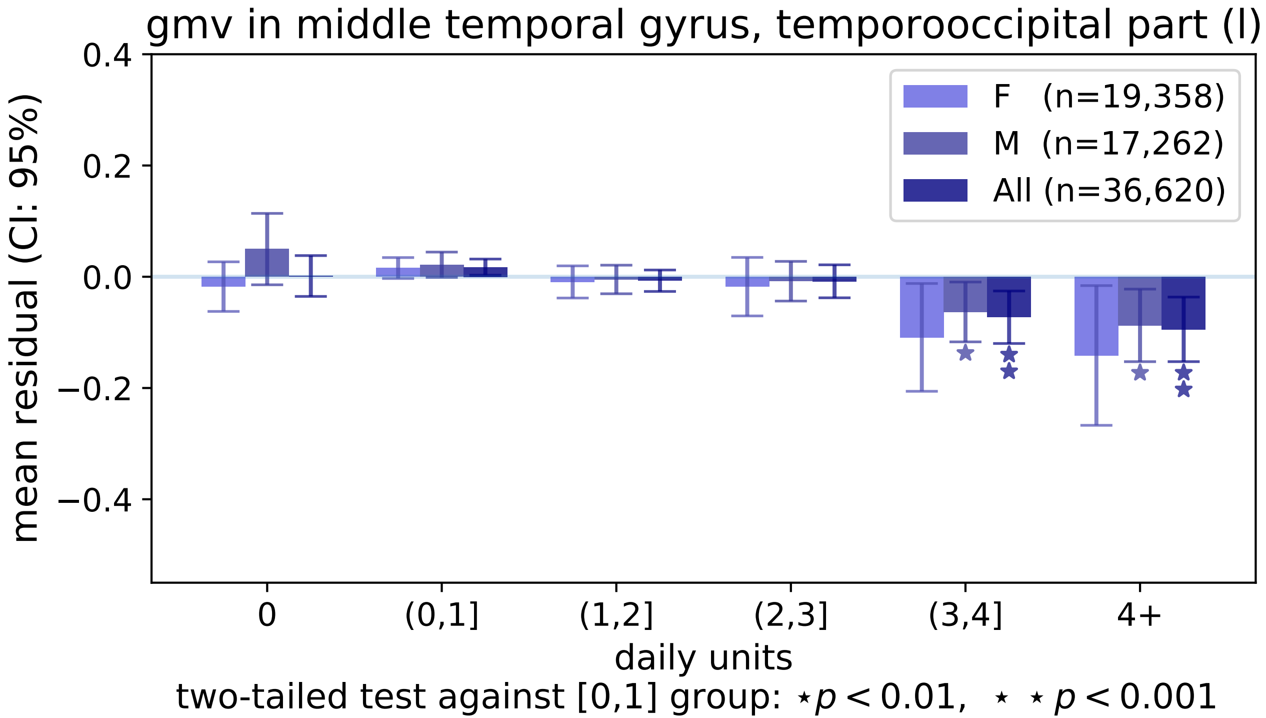

gmv in middle temporal gyrus, temporooccipital part (l)
0.4
0.2
0.0
−0.2
−0.4
mean residual (CI: 95%)
F (n=19,358)
M (n=17,262)
All (n=36,620)
0
(0,1]
(1,2]
(2,3]
(3,4]
4+
daily units
two-tailed test against [0,1] group: ⋆p < 0.01, ⋆ ⋆ p < 0.001

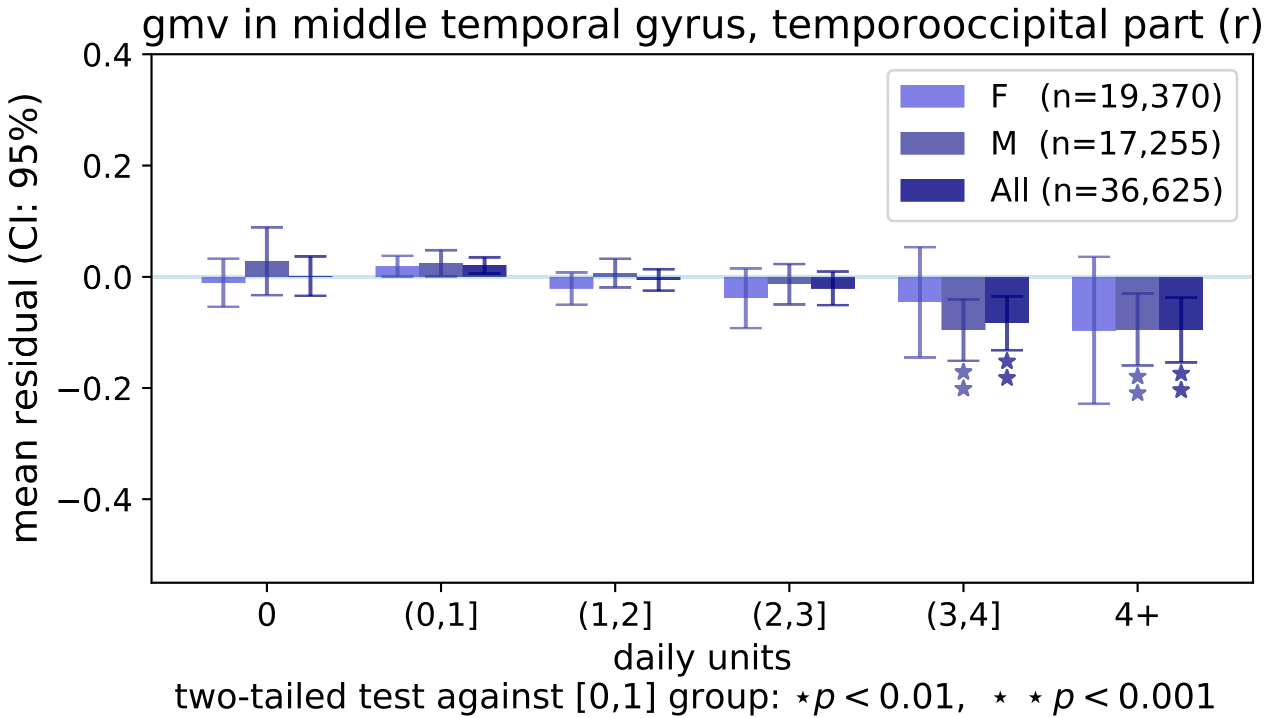

gmv in middle temporal gyrus, temporooccipital part (r)
mean residual (CI: 95%)
0.4
0.2
0.0
−0.2
−0.4
F (n=19,370)
M (n=17,255)
All (n=36,625)
0
(0,1]
(1,2]
(2,3]
(3,4]
4+
daily units
two-tailed test against [0,1] group: ⋆p < 0.01, ⋆ ⋆p < 0.001

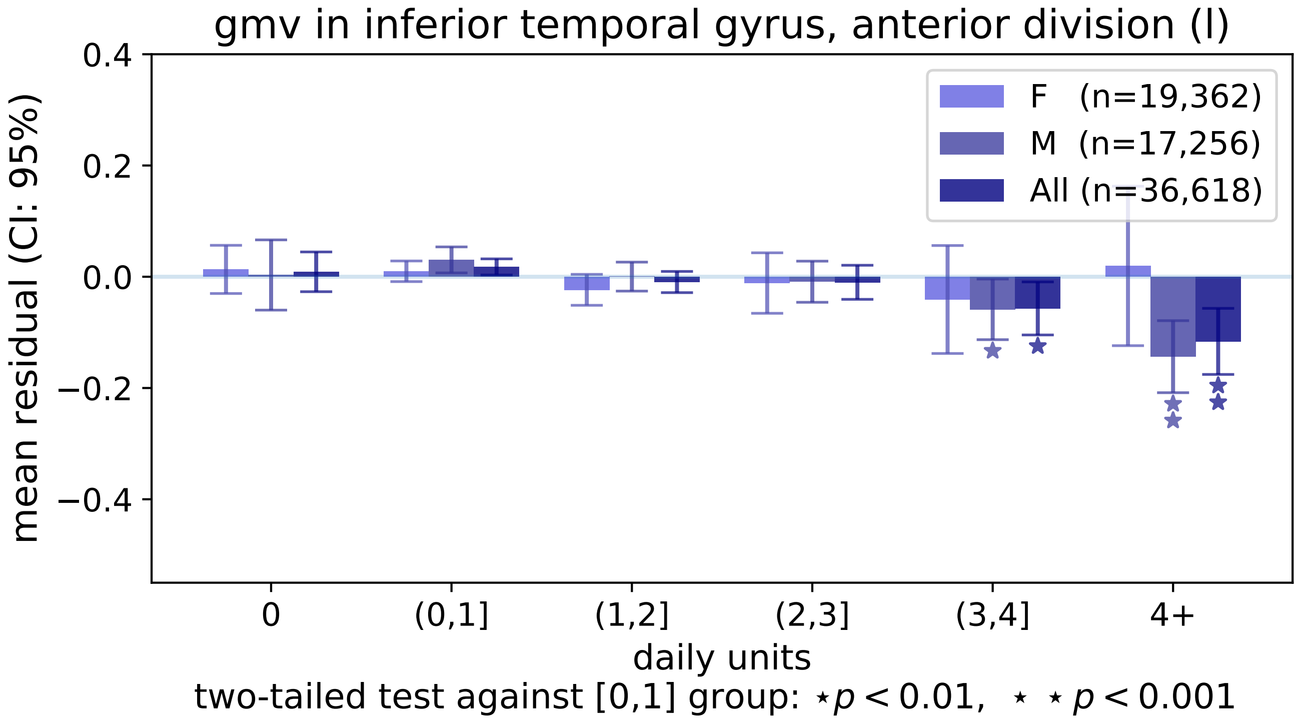

gmv in inferior temporal gyrus, anterior division (l)
0.4
0.2
0.0
−0.2
−0.4
mean residual (CI: 95%)
F (n=19,362)
M (n=17,256)
All (n=36,618)
0
(0,1]
(1,2]
(2,3]
(3,4]
4+
daily units
two-tailed test against [0,1] group: ⋆p < 0.01, ⋆ ⋆ p < 0.001

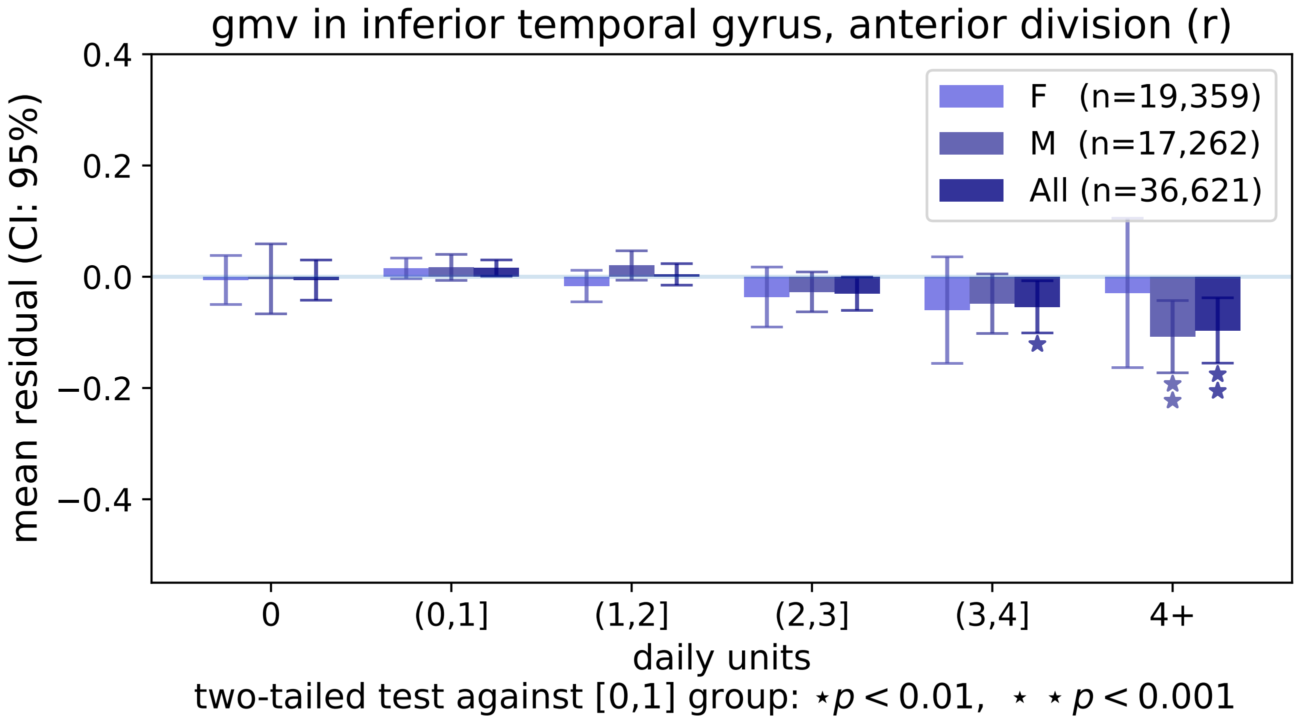

gmv in inferior temporal gyrus, anterior division (r)
mean residual (CI: 95%)
0.4
0.2
0.0
−0.2
−0.4
F (n=19,359)
M (n=17,262)
All (n=36,621)
0
(0,1]
(1,2]
(2,3]
(3,4]
4+
daily units
two-tailed test against [0,1] group: ⋆p < 0.01, ⋆ ⋆ p < 0.001

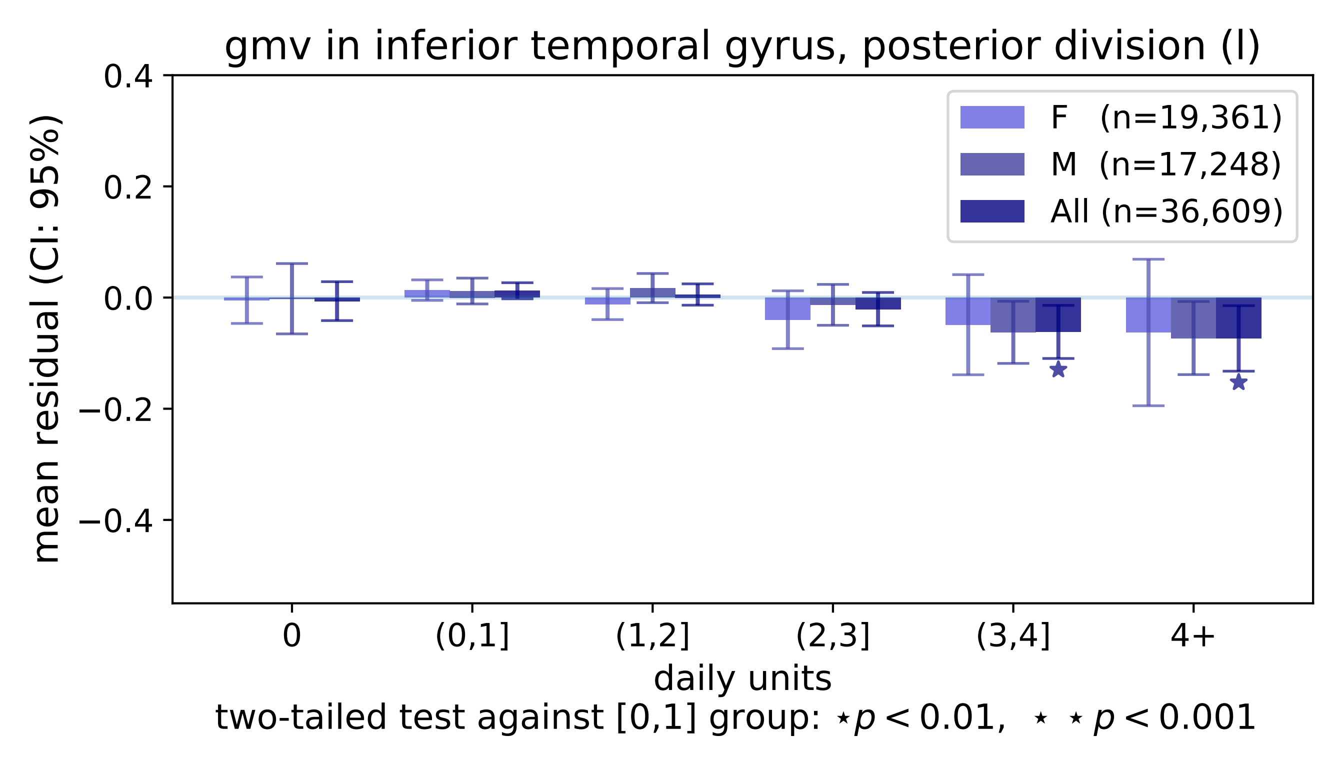
gmv in inferior temporal gyrus, posterior division (l)
mean residual (CI: 95%)
0.4
0.2
0.0
−0.2
−0.4
F (n=19,361)
M (n=17,248)
All (n=36,609)
0
(0,1]
(1,2]
(2,3]
(3,4]
4+
daily units
two-tailed test against [0,1] group: ⋆p < 0.01, ⋆ ⋆ p < 0.001

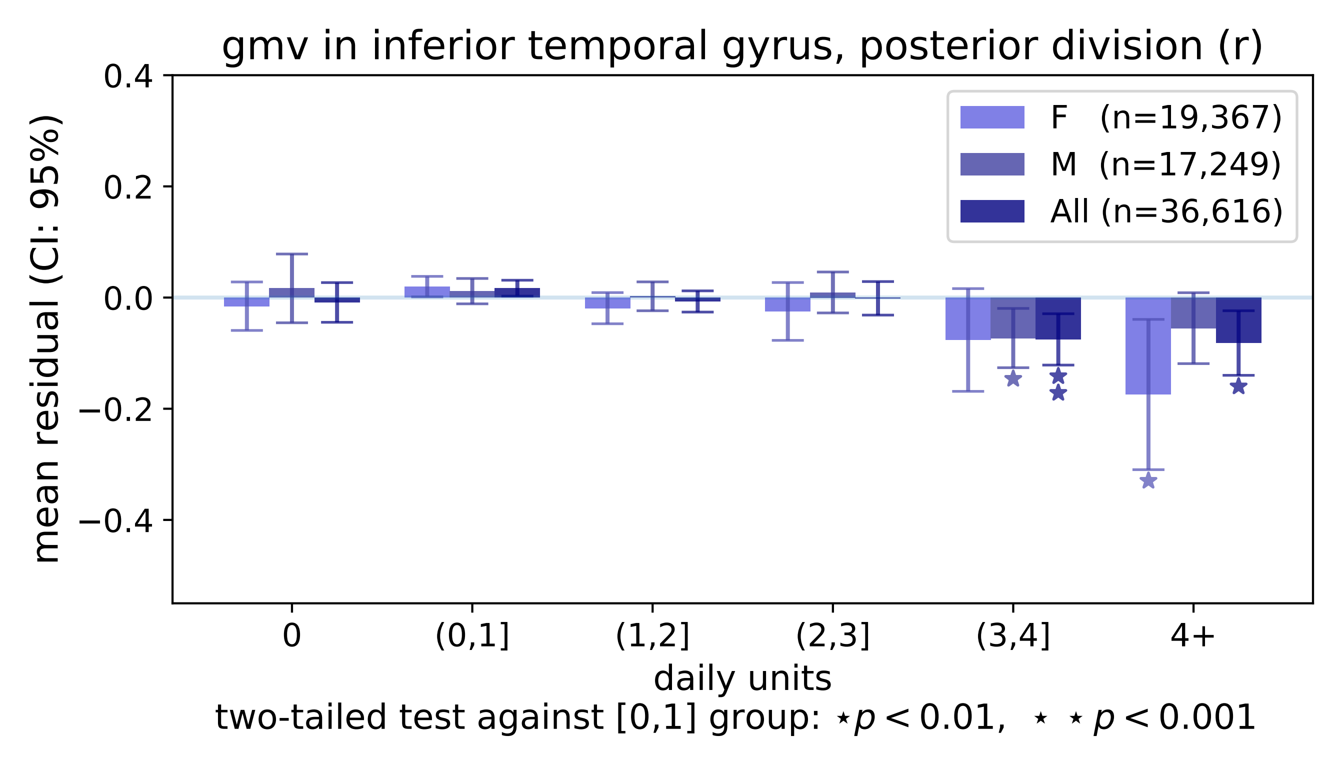
gmv in inferior temporal gyrus, posterior division (r)
mean residual (CI: 95%)
0.4
0.2
0.0
−0.2
−0.4
F (n=19,367)
M (n=17,249)
All (n=36,616)
0
(0,1]
(1,2]
(2,3]
(3,4]
4+
daily units
two-tailed test against [0,1] group: ⋆p < 0.01, ⋆ ⋆ p < 0.001

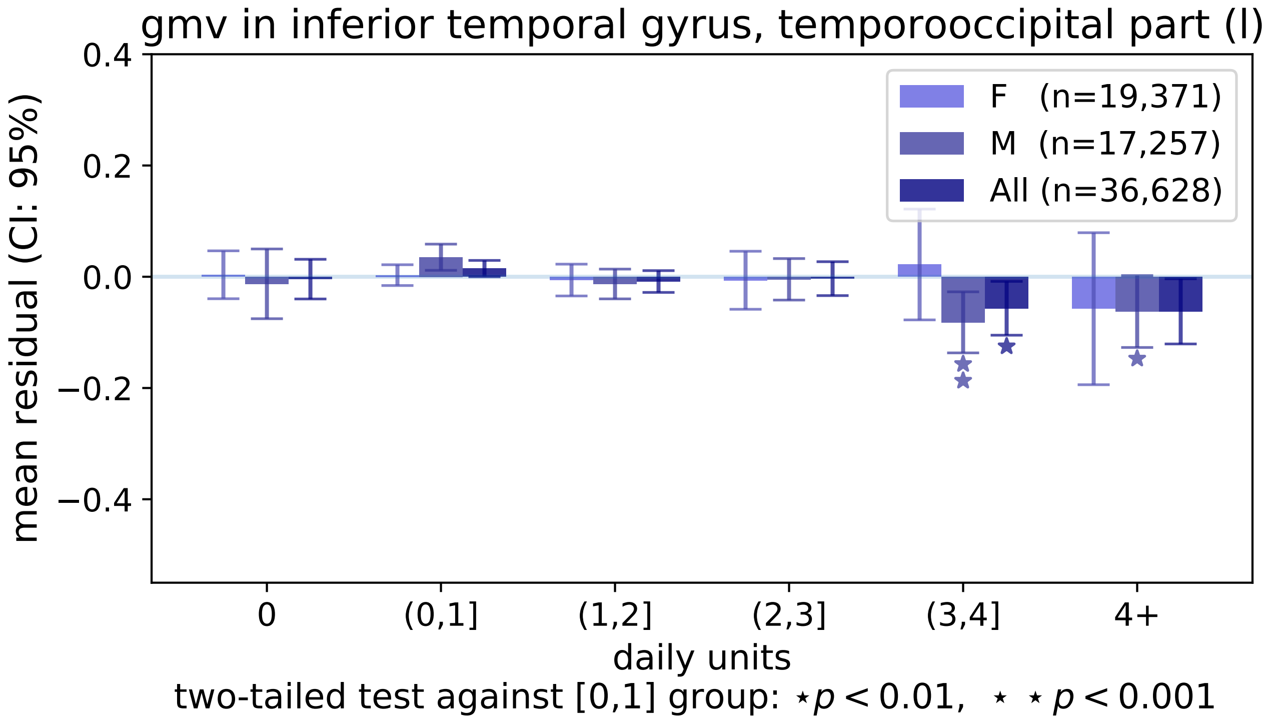

gmv in inferior temporal gyrus, temporooccipital part (l)
0.4
0.2
0.0
−0.2
−0.4
mean residual (CI: 95%)
F (n=19,371)
M (n=17,257)
All (n=36,628)
0
(0,1]
(1,2]
(2,3]
(3,4]
4+
daily units
two-tailed test against [0,1] group: ⋆p < 0.01, ⋆ ⋆ p < 0.001

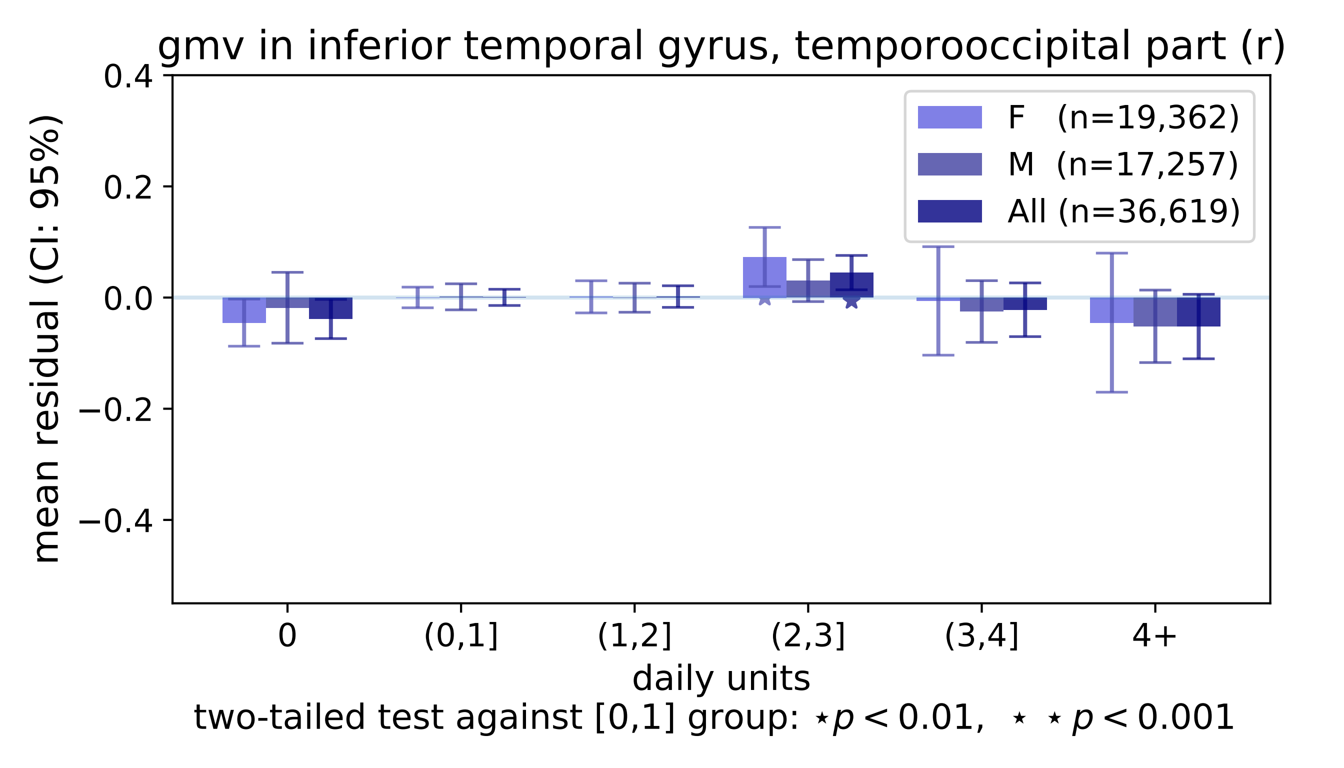

gmv in inferior temporal gyrus, temporooccipital part (r)
mean residual (CI: 95%)
0.4
0.2
0.0
−0.2
−0.4
F (n=19,362)
M (n=17,257)
All (n=36,619)
0
(0,1]
(1,2]
(2,3]
(3,4]
4+
daily units
two-tailed test against [0,1] group: ⋆p < 0.01, ⋆ ⋆p < 0.001

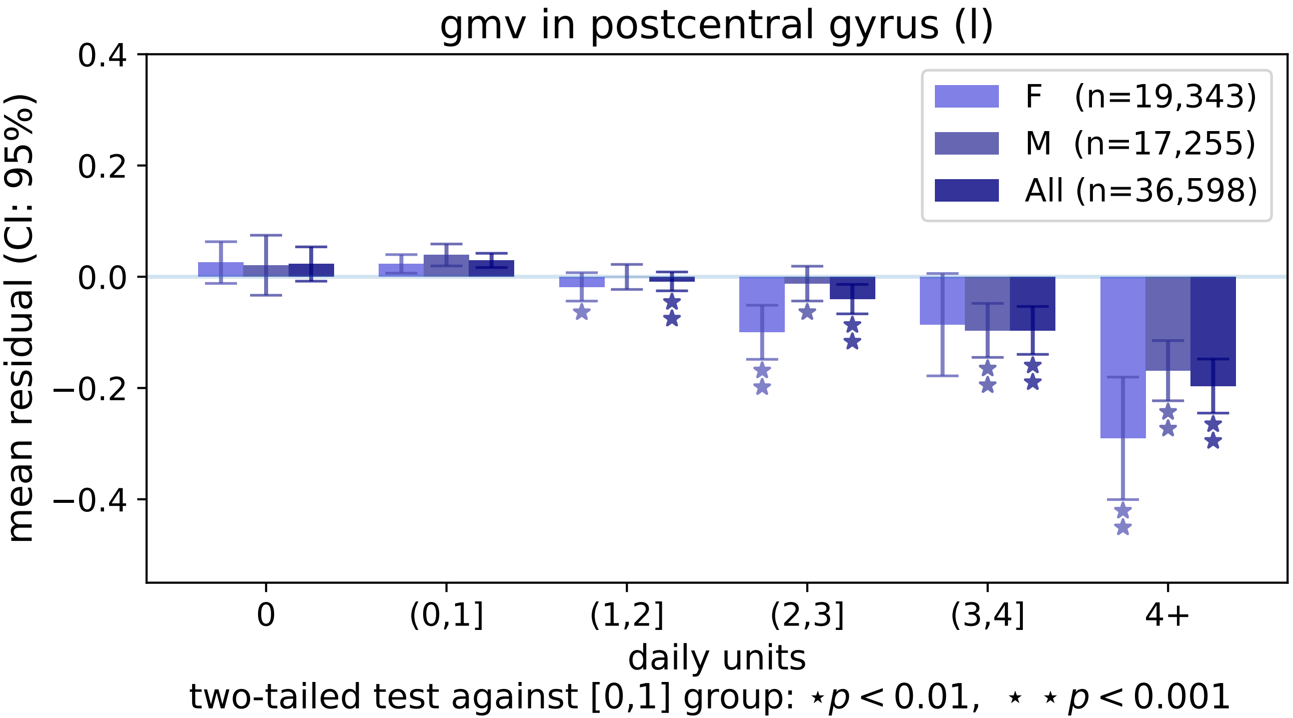
gmv in postcentral gyrus (l)
0.4
0.2
0.0
−0.2
−0.4
mean residual (CI: 95%)
F (n=19,343)
M (n=17,255)
All (n=36,598)
0
(0,1]
(1,2]
(2,3]
(3,4]
4+
daily units
two-tailed test against [0,1] group: ⋆ $p < 0.01$, ⋆ ⋆ $p < 0.001$

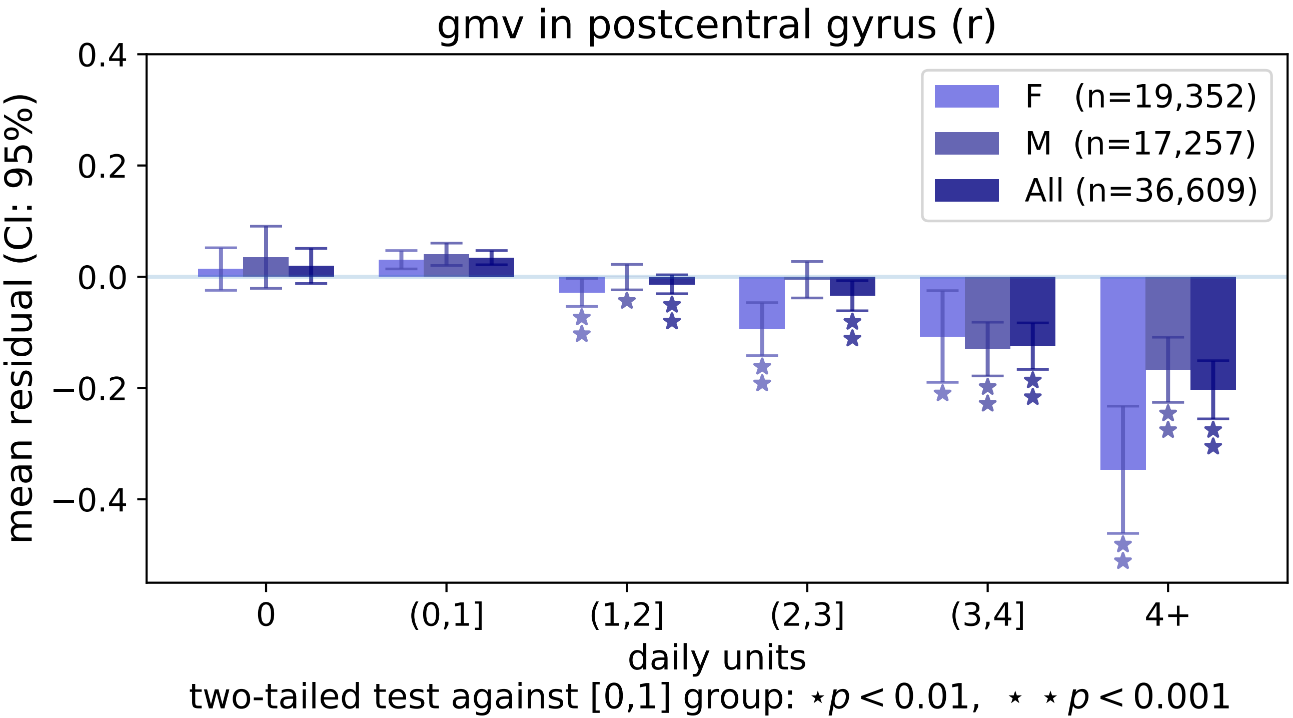
gmv in postcentral gyrus (r)
F (n=19,352)
M (n=17,257)
All (n=36,609)
mean residual (CI: 95%)
0.4
0.2
0.0
−0.2
−0.4
0
(0,1]
(1,2]
(2,3]
(3,4]
4+
daily units
two-tailed test against [0,1] group: ⋆p < 0.01, ⋆ ⋆ p < 0.001

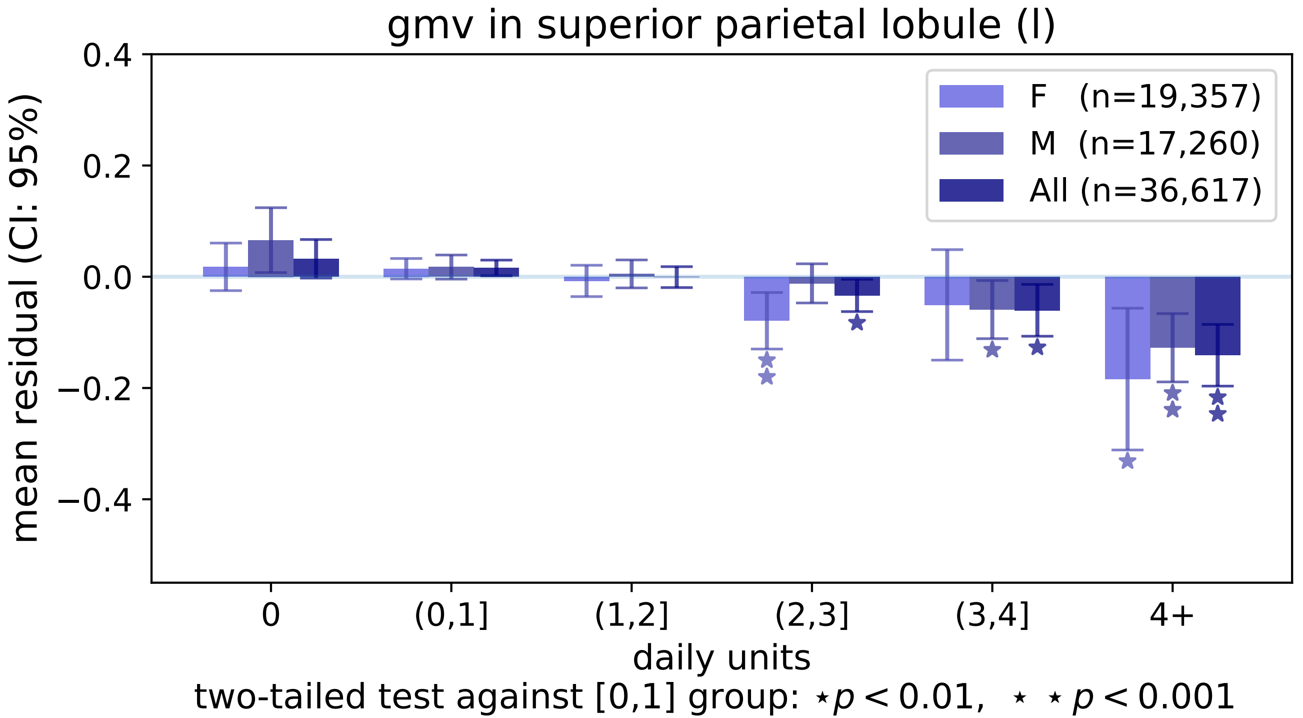
gmv in superior parietal lobule (l)
mean residual (CI: 95%)
0.4
0.2
0.0
−0.2
−0.4
F (n=19,357)
M (n=17,260)
All (n=36,617)
0
(0,1]
(1,2]
(2,3]
(3,4]
4+
daily units
two-tailed test against [0,1] group: ⋆p < 0.01, ⋆ ⋆ p < 0.001

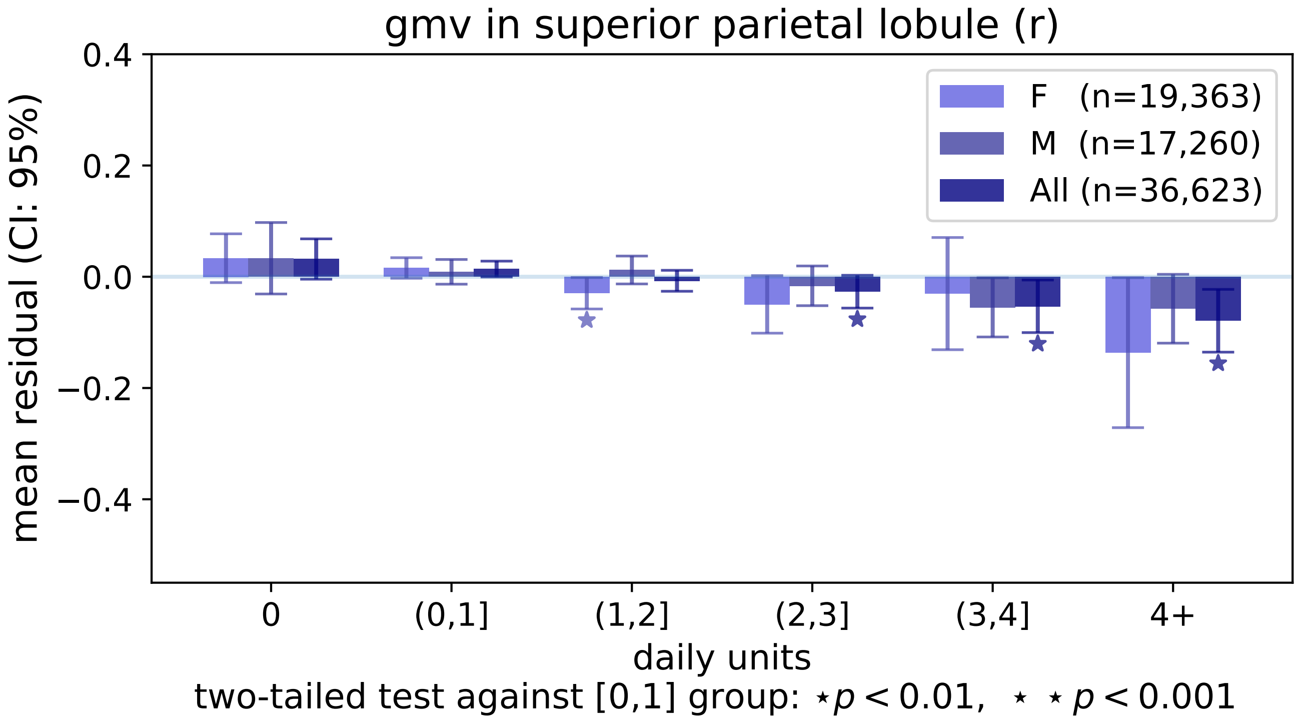

gmv in superior parietal lobule (r)
0.4
0.2
0.0
−0.2
−0.4
mean residual (CI: 95%)
F (n=19,363)
M (n=17,260)
All (n=36,623)
0
(0,1]
(1,2]
(2,3]
(3,4]
4+
daily units
two-tailed test against [0,1] group: ⋆p < 0.01, ⋆ ⋆ p < 0.001

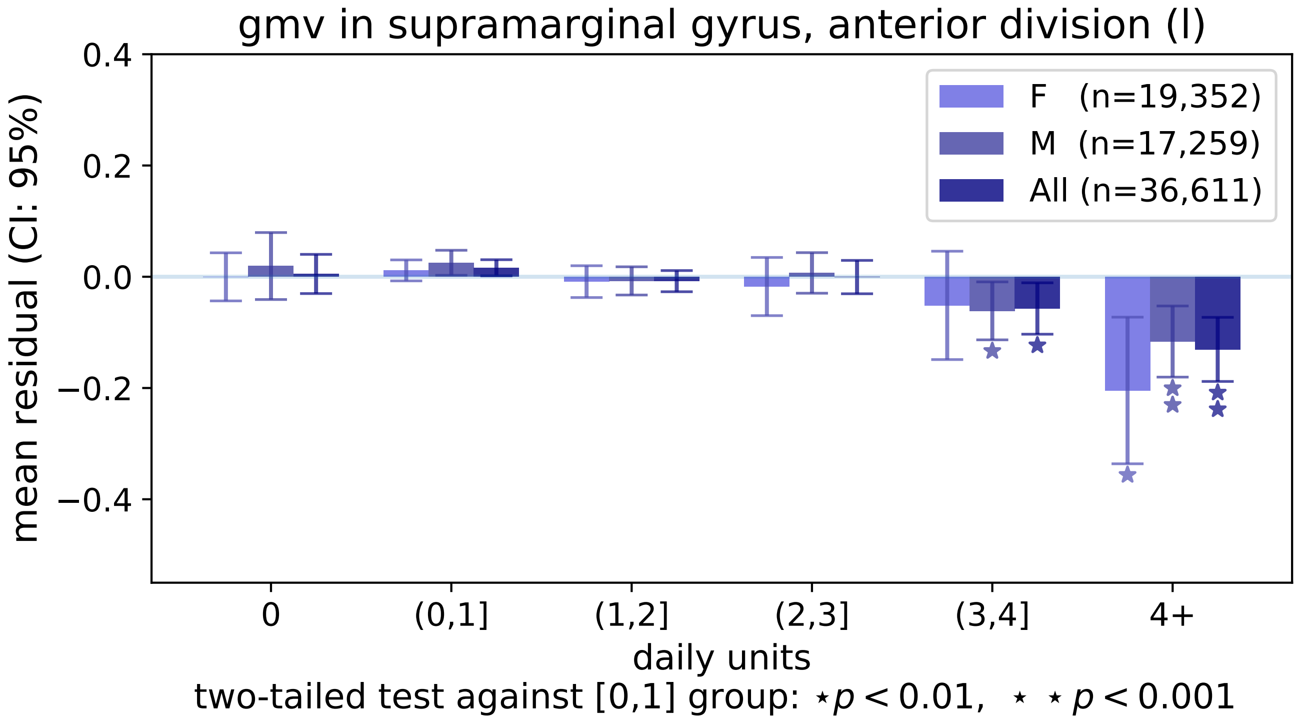
gmv in supramarginal gyrus, anterior division (l)
0.4
0.2
0.0
−0.2
−0.4
mean residual (CI: 95%)
F (n=19,352)
M (n=17,259)
All (n=36,611)
0
(0,1]
(1,2]
(2,3]
(3,4]
4+
daily units
two-tailed test against [0,1] group: ⋆p < 0.01, ⋆ ⋆ p < 0.001

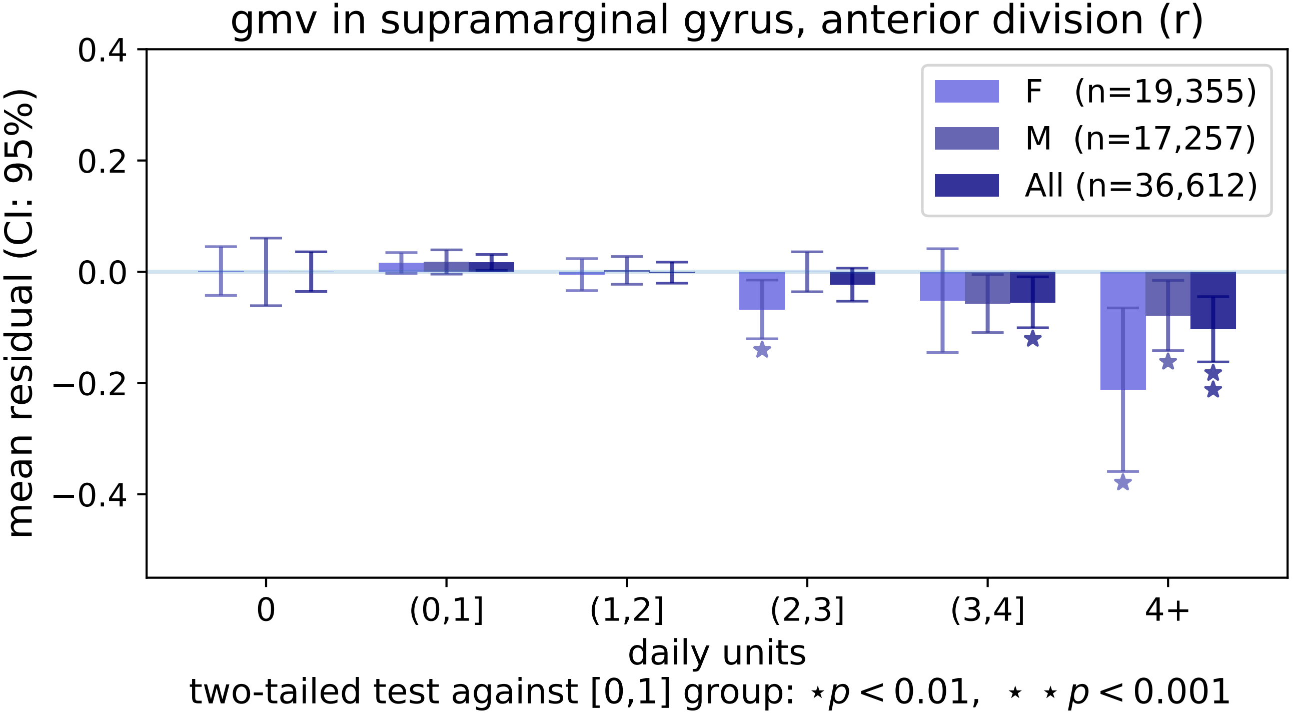

gmv in supramarginal gyrus, anterior division (r)
mean residual (CI: 95%)
0.4
0.2
0.0
−0.2
−0.4
F (n=19,355)
M (n=17,257)
All (n=36,612)
0
(0,1]
(1,2]
(2,3]
(3,4]
4+
daily units
two-tailed test against [0,1] group: ⋆ p < 0.01, ⋆ ⋆ p < 0.001

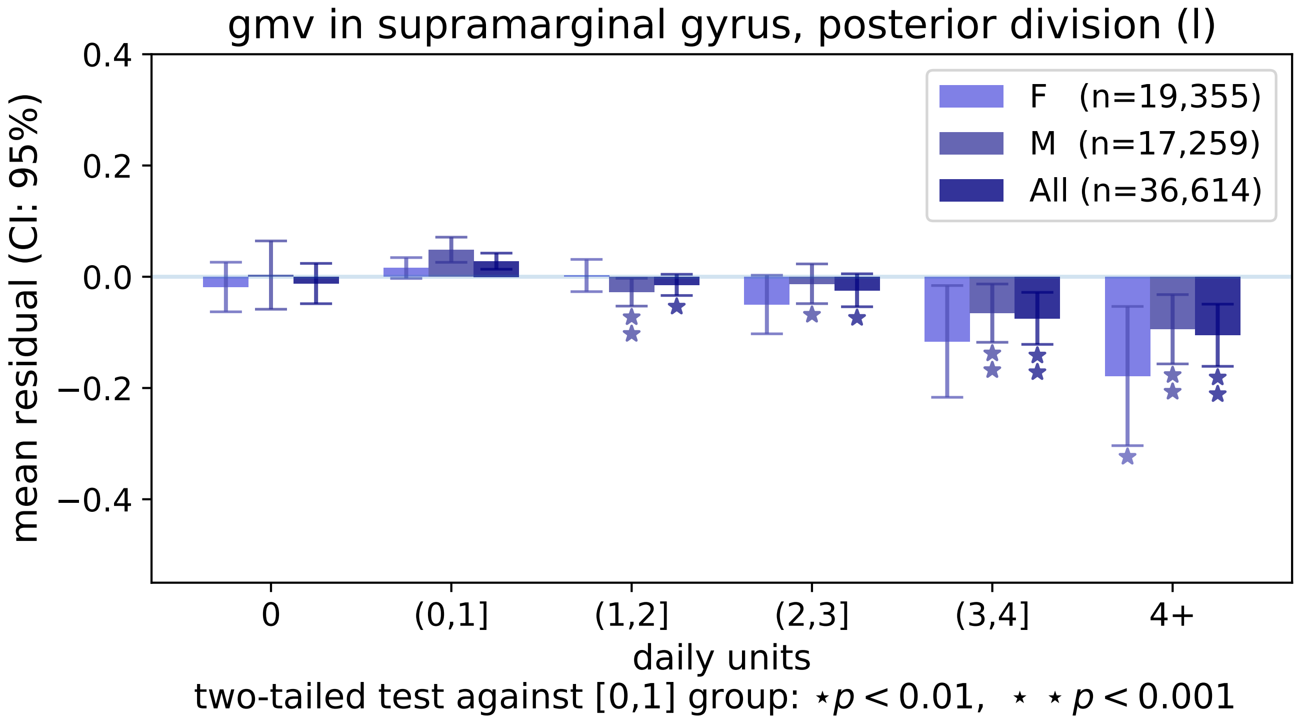

gmv in supramarginal gyrus, posterior division (l)
mean residual (CI: 95%)
0.4
0.2
0.0
−0.2
−0.4
F (n=19,355)
M (n=17,259)
All (n=36,614)
0
(0,1]
(1,2]
(2,3]
(3,4]
4+
daily units
two-tailed test against [0,1] group: ⋆ p < 0.01, ⋆ ⋆ p < 0.001

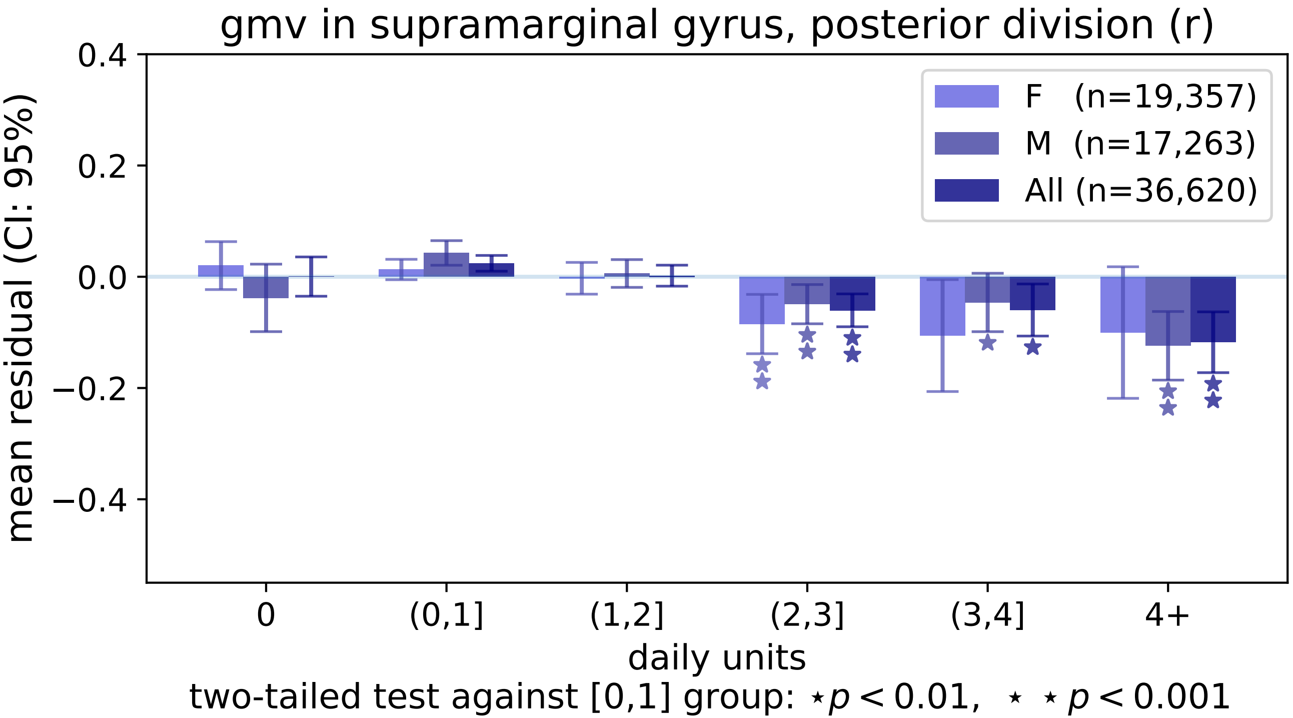

gmv in supramarginal gyrus, posterior division (r)
mean residual (CI: 95%)
0.4
0.2
0.0
−0.2
−0.4
F (n=19,357)
M (n=17,263)
All (n=36,620)
0
(0,1]
(1,2]
(2,3]
(3,4]
4+
daily units
two-tailed test against [0,1] group: ⋆p < 0.01, ⋆ ⋆ p < 0.001

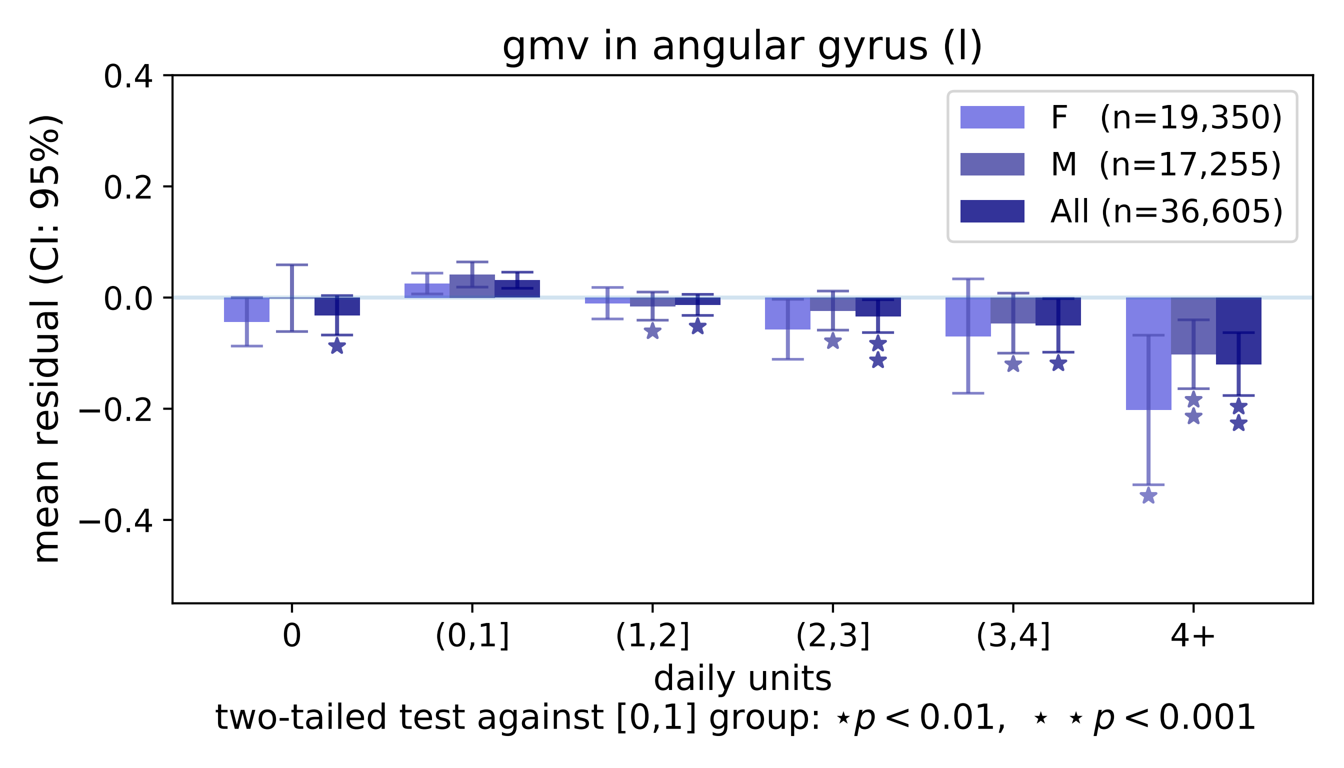

gmv in angular gyrus (l)
mean residual (CI: 95%)
0.4
0.2
0.0
−0.2
−0.4
F (n=19,350)
M (n=17,255)
All (n=36,605)
0
(0,1]
(1,2]
(2,3]
(3,4]
4+
daily units
two-tailed test against [0,1] group: ⋆p < 0.01, ⋆ ⋆p < 0.001

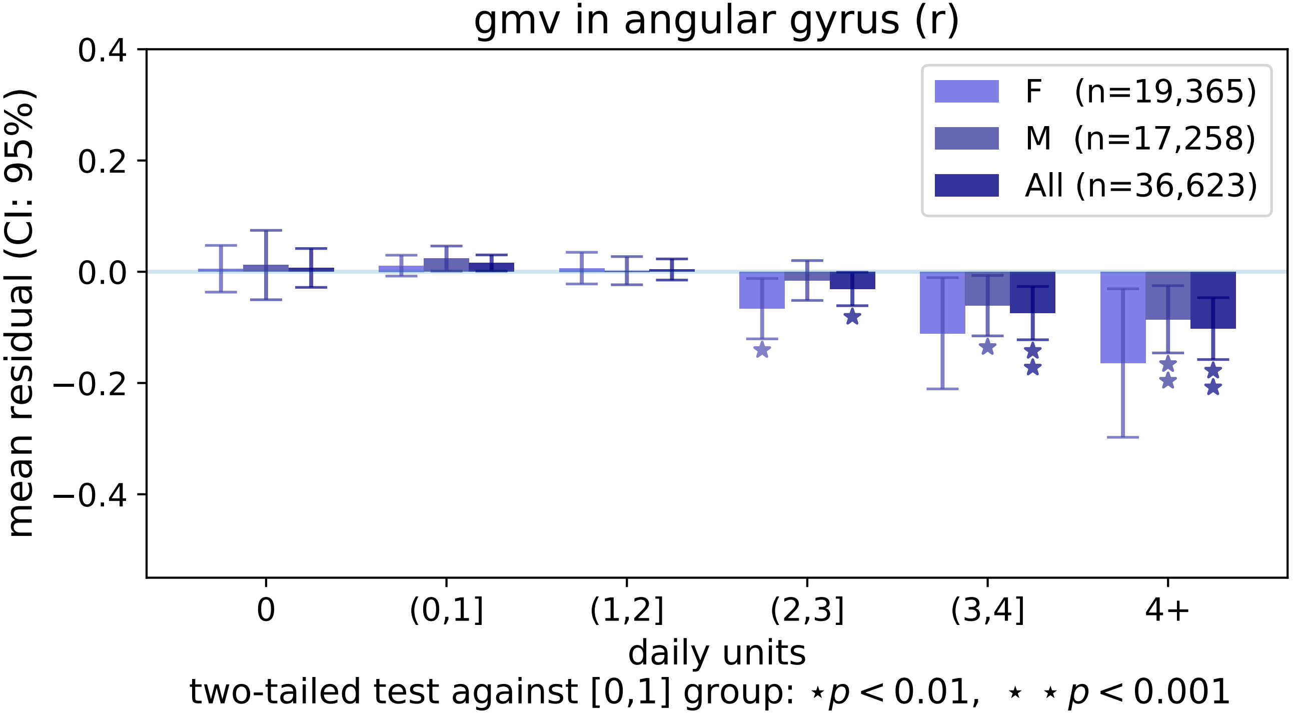
gmv in angular gyrus (r)
mean residual (CI: 95%)
0.4
0.2
0.0
−0.2
−0.4
F (n=19,365)
M (n=17,258)
All (n=36,623)
0
(0,1]
(1,2]
(2,3]
(3,4]
4+
daily units
two-tailed test against [0,1] group: ⋆ $p < 0.01$, ⋆ ⋆ $p < 0.001$

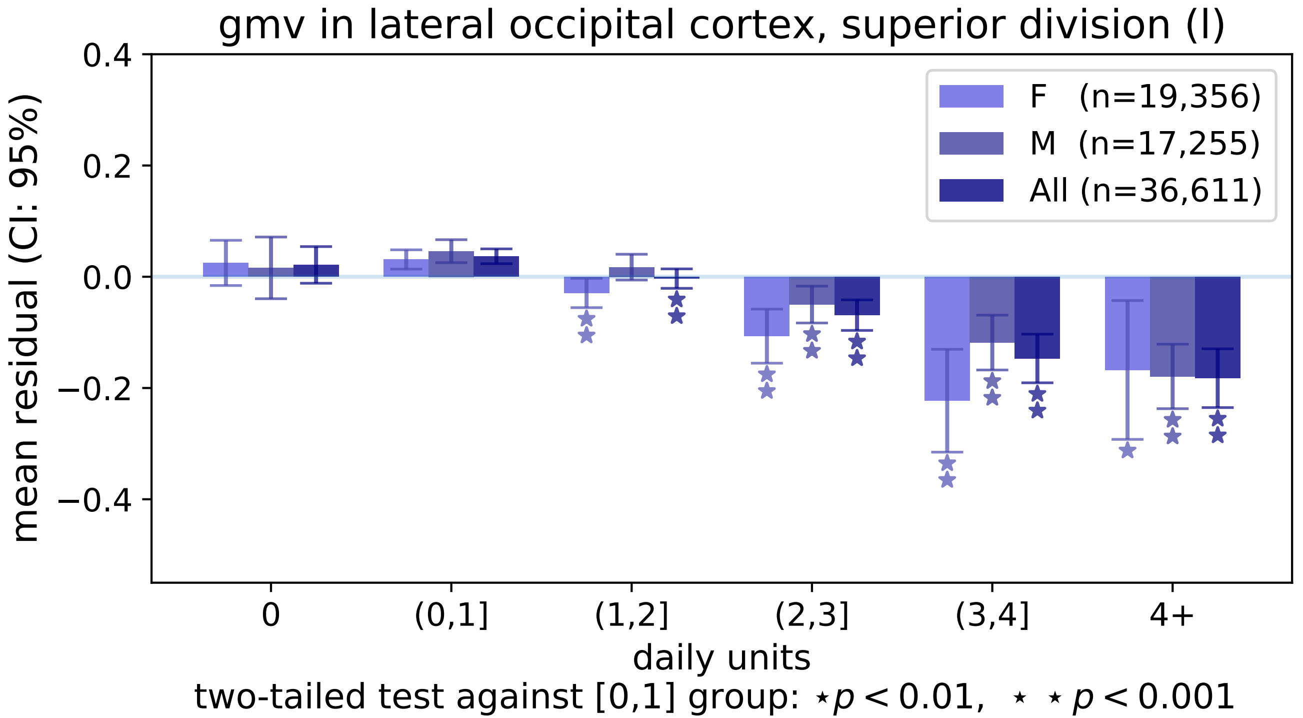
gmv in lateral occipital cortex, superior division (l)
mean residual (CI: 95%)
0.4
0.2
0.0
−0.2
−0.4
F (n=19,356)
M (n=17,255)
All (n=36,611)
0
(0,1]
(1,2]
(2,3]
(3,4]
4+
daily units
two-tailed test against [0,1] group: ⋆p < 0.01, ⋆ ⋆ p < 0.001

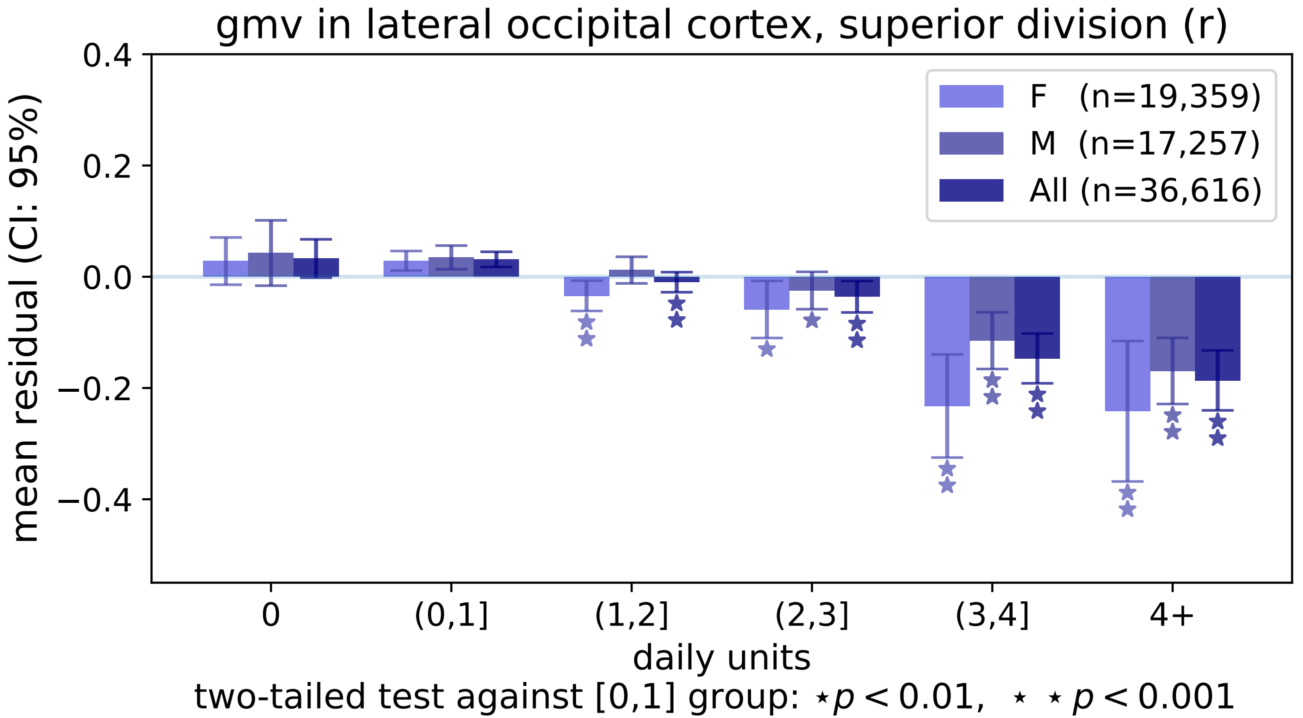
gmv in lateral occipital cortex, superior division (r)
0.4
0.2
0.0
−0.2
−0.4
mean residual (CI: 95%)
F (n=19,359)
M (n=17,257)
All (n=36,616)
0
(0,1]
(1,2]
(2,3]
(3,4]
4+
daily units
two-tailed test against [0,1] group: ⋆p < 0.01, ⋆ ⋆ p < 0.001

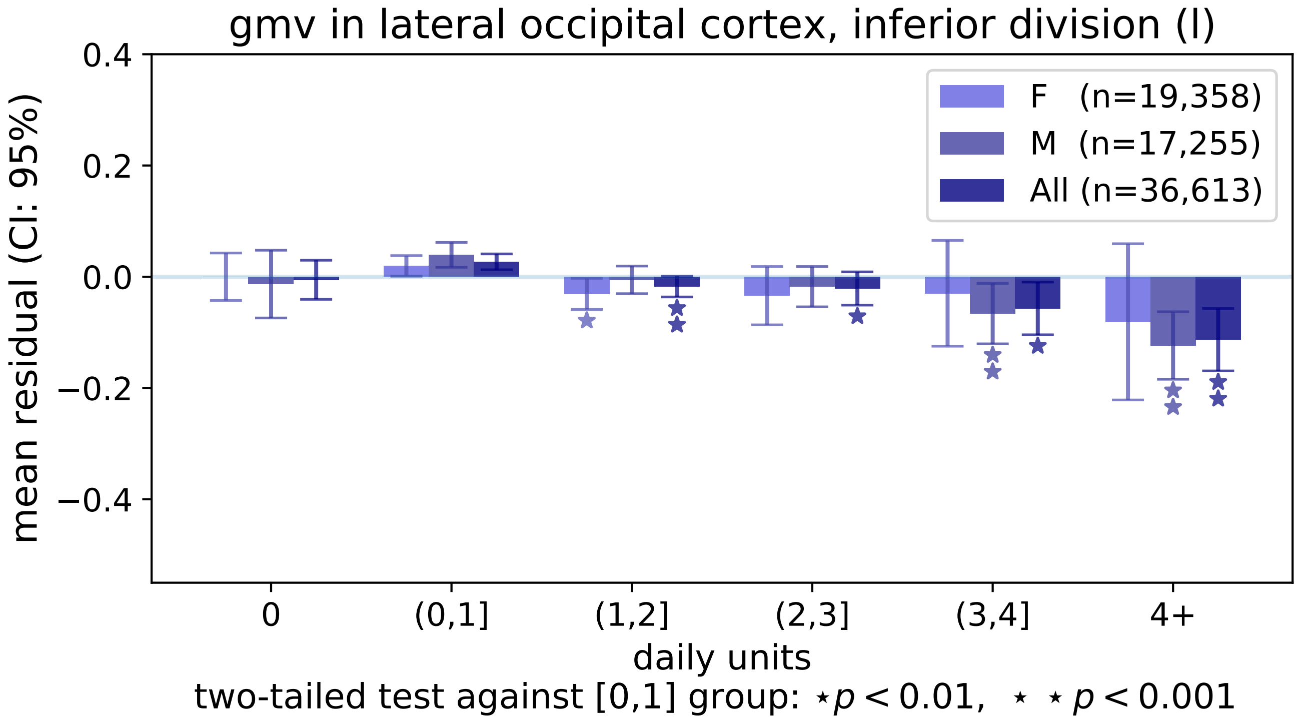

gmv in lateral occipital cortex, inferior division (l)
F (n=19,358)
M (n=17,255)
All (n=36,613)
mean residual (CI: 95%)
0.4
0.2
0.0
−0.2
−0.4
0
(0,1]
(1,2]
(2,3]
(3,4]
4+
daily units
two-tailed test against [0,1] group: ⋆ p < 0.01, ⋆ ⋆ p < 0.001

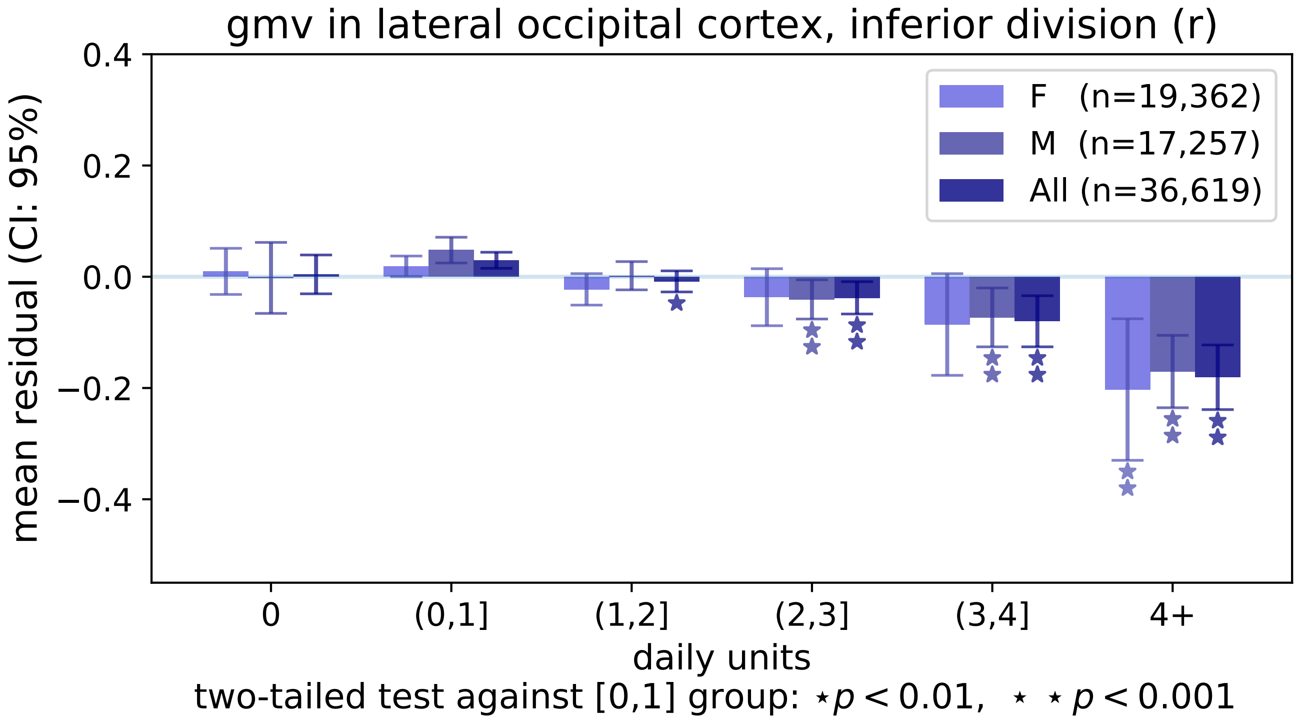
gmv in lateral occipital cortex, inferior division (r)
mean residual (CI: 95%)
0.4
0.2
0.0
−0.2
−0.4
F (n=19,362)
M (n=17,257)
All (n=36,619)
0
(0,1]
(1,2]
(2,3]
(3,4]
4+
daily units
two-tailed test against [0,1] group: ⋆ p < 0.01, ⋆ ⋆ p < 0.001

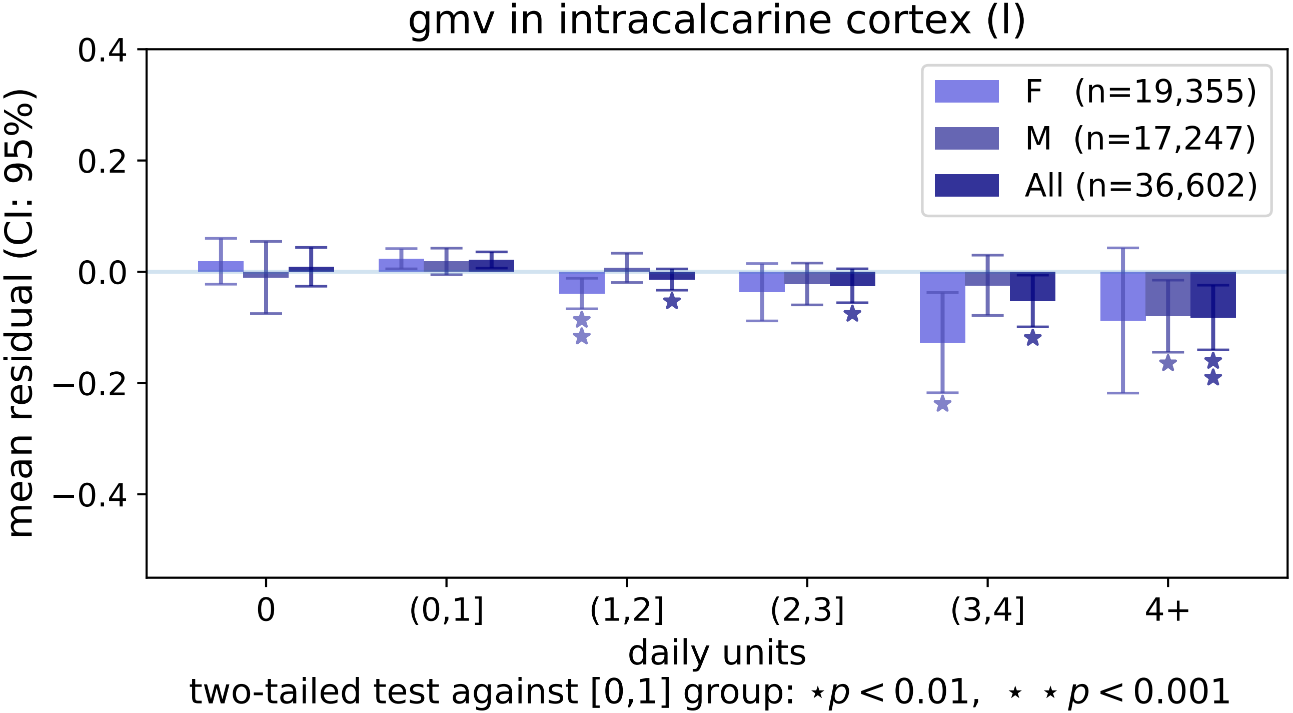
gmv in intracalcarine cortex (l)
mean residual (CI: 95%)
0.4
0.2
0.0
−0.2
−0.4
F (n=19,355)
M (n=17,247)
All (n=36,602)
0
(0,1]
(1,2]
(2,3]
(3,4]
4+
daily units
two-tailed test against [0,1] group: ⋆p < 0.01, ⋆ ⋆ p < 0.001

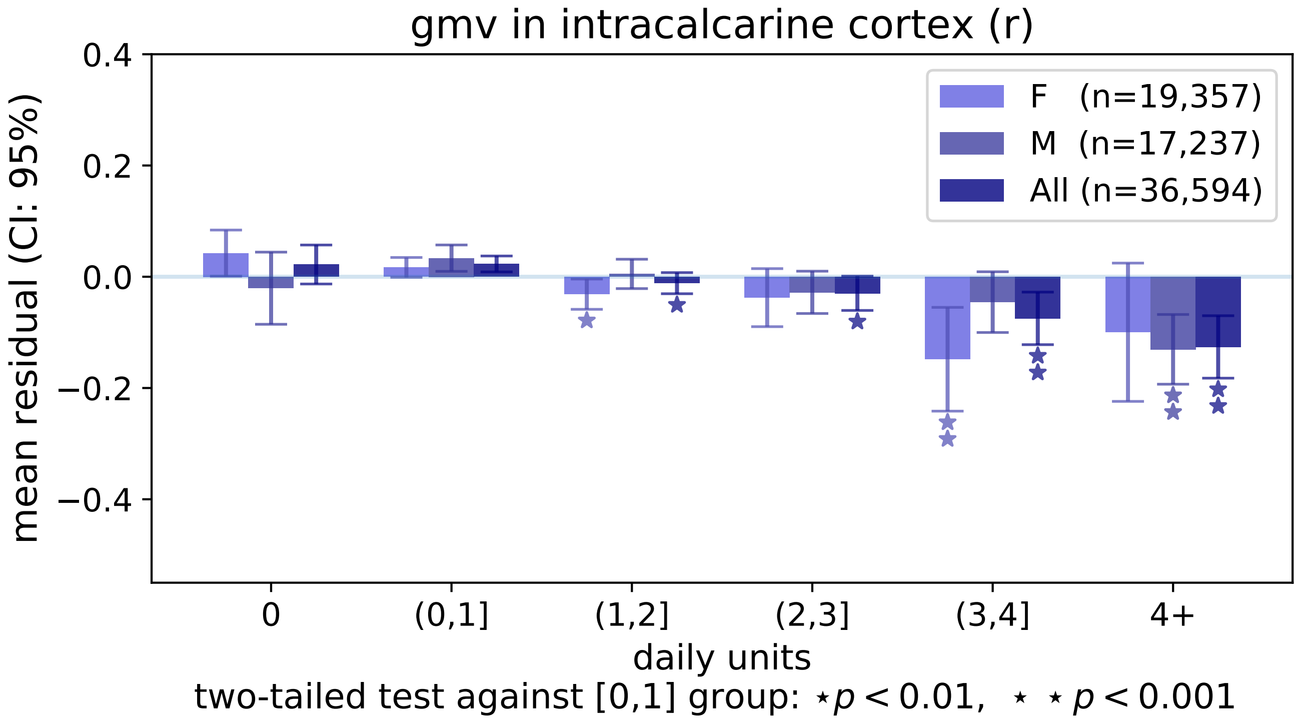
gmv in intracalcarine cortex (r)
mean residual (CI: 95%)
0.4
0.2
0.0
−0.2
−0.4
F (n=19,357)
M (n=17,237)
All (n=36,594)
0
(0,1]
(1,2]
(2,3]
(3,4]
4+
daily units
two-tailed test against [0,1] group: ⋆p < 0.01, ⋆ ⋆ p < 0.001

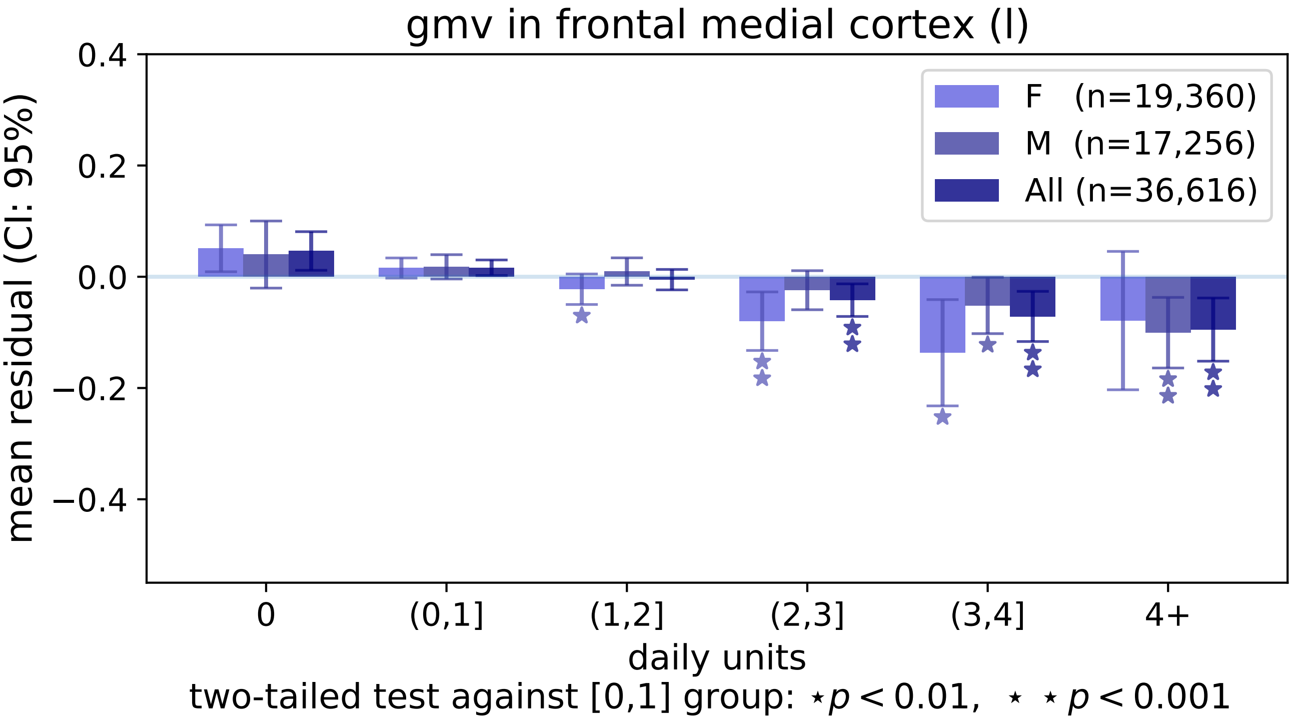
gmv in frontal medial cortex (l)
mean residual (CI: 95%)
0.4
0.2
0.0
−0.2
−0.4
F (n=19,360)
M (n=17,256)
All (n=36,616)
0
(0,1]
(1,2]
(2,3]
(3,4]
4+
daily units
two-tailed test against [0,1] group: ⋆p < 0.01, ⋆ ⋆ p < 0.001

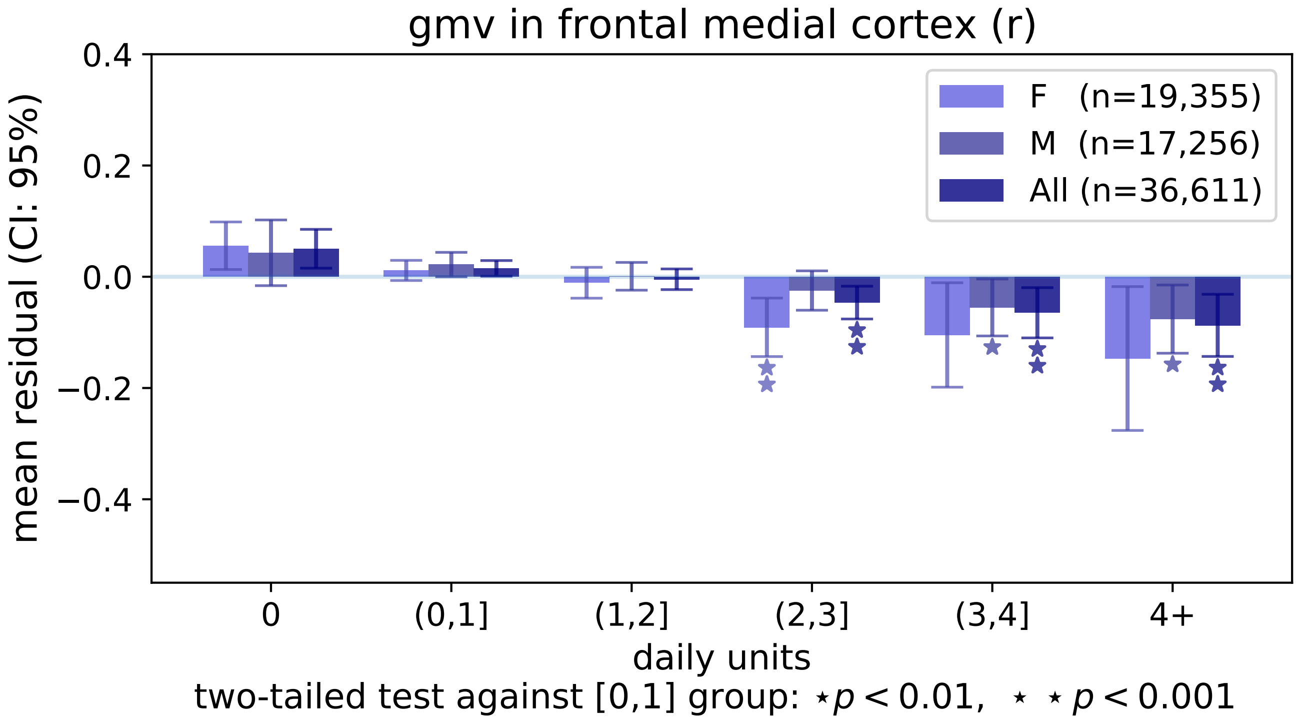
gmv in frontal medial cortex (r)
mean residual (CI: 95%)
0.4
0.2
0.0
−0.2
−0.4
F (n=19,355)
M (n=17,256)
All (n=36,611)
0
(0,1]
(1,2]
(2,3]
(3,4]
4+
daily units
two-tailed test against [0,1] group: ⋆p < 0.01, ⋆ ⋆ p < 0.001

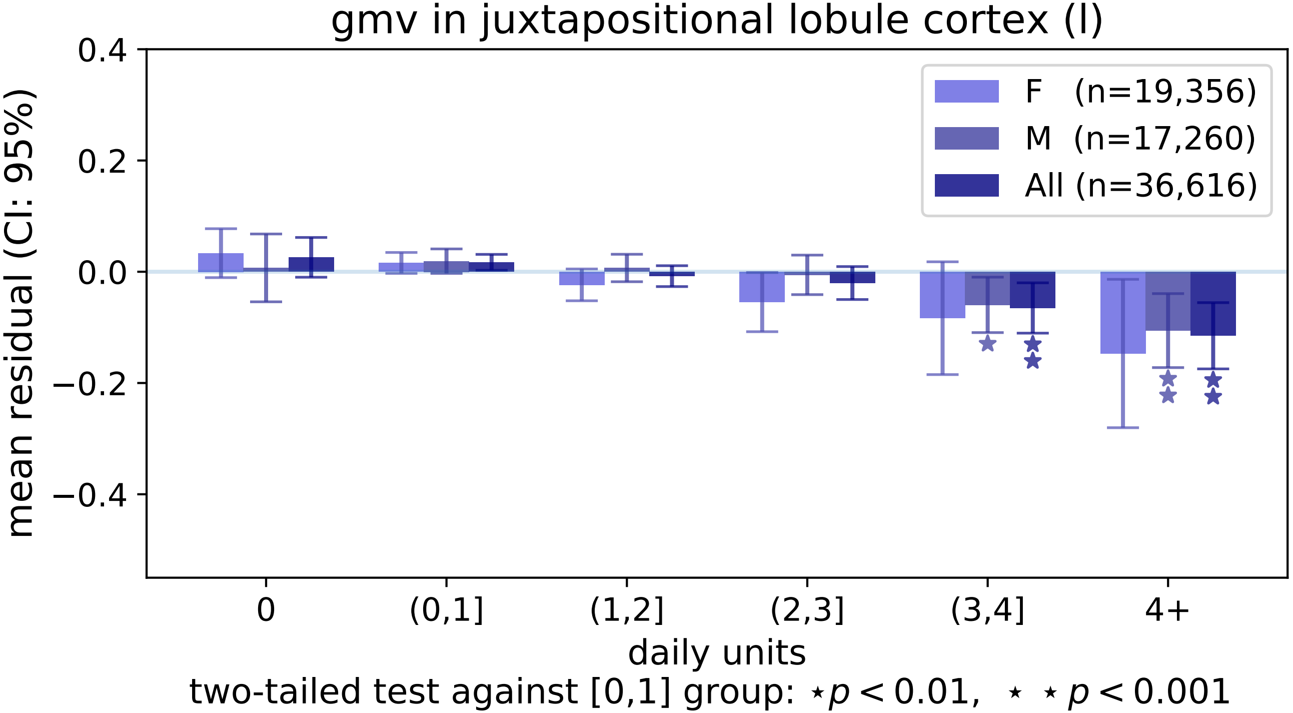
gmv in juxtapositional lobule cortex (l)
0.4
0.2
0.0
−0.2
−0.4
mean residual (CI: 95%)
F (n=19,356)
M (n=17,260)
All (n=36,616)
0
(0,1]
(1,2]
(2,3]
(3,4]
4+
daily units
two-tailed test against [0,1] group: ⋆ $p < 0.01$, ⋆ ⋆ $p < 0.001$

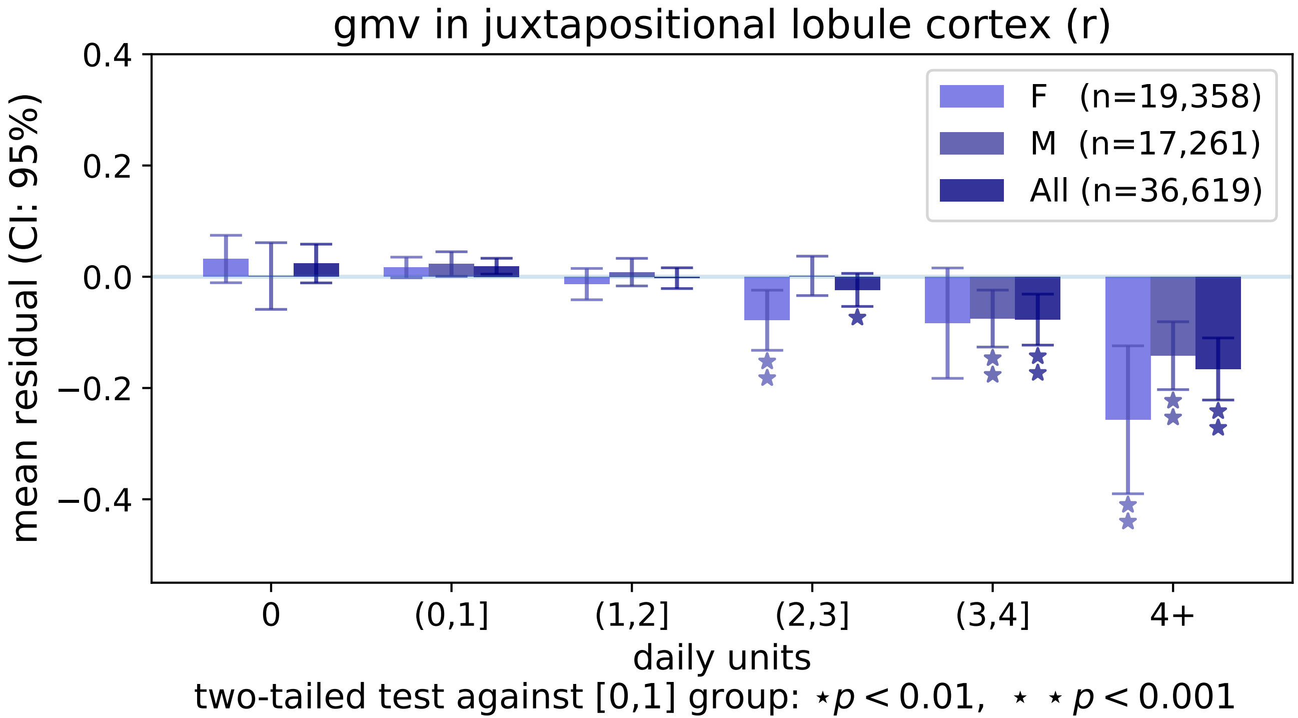
gmv in juxtapositional lobule cortex (r)
mean residual (CI: 95%)
0.4
0.2
0.0
−0.2
−0.4
F (n=19,358)
M (n=17,261)
All (n=36,619)
0
(0,1]
(1,2]
(2,3]
(3,4]
4+
daily units
two-tailed test against [0,1] group: ⋆ $p < 0.01$, ⋆ ⋆ $p < 0.001$

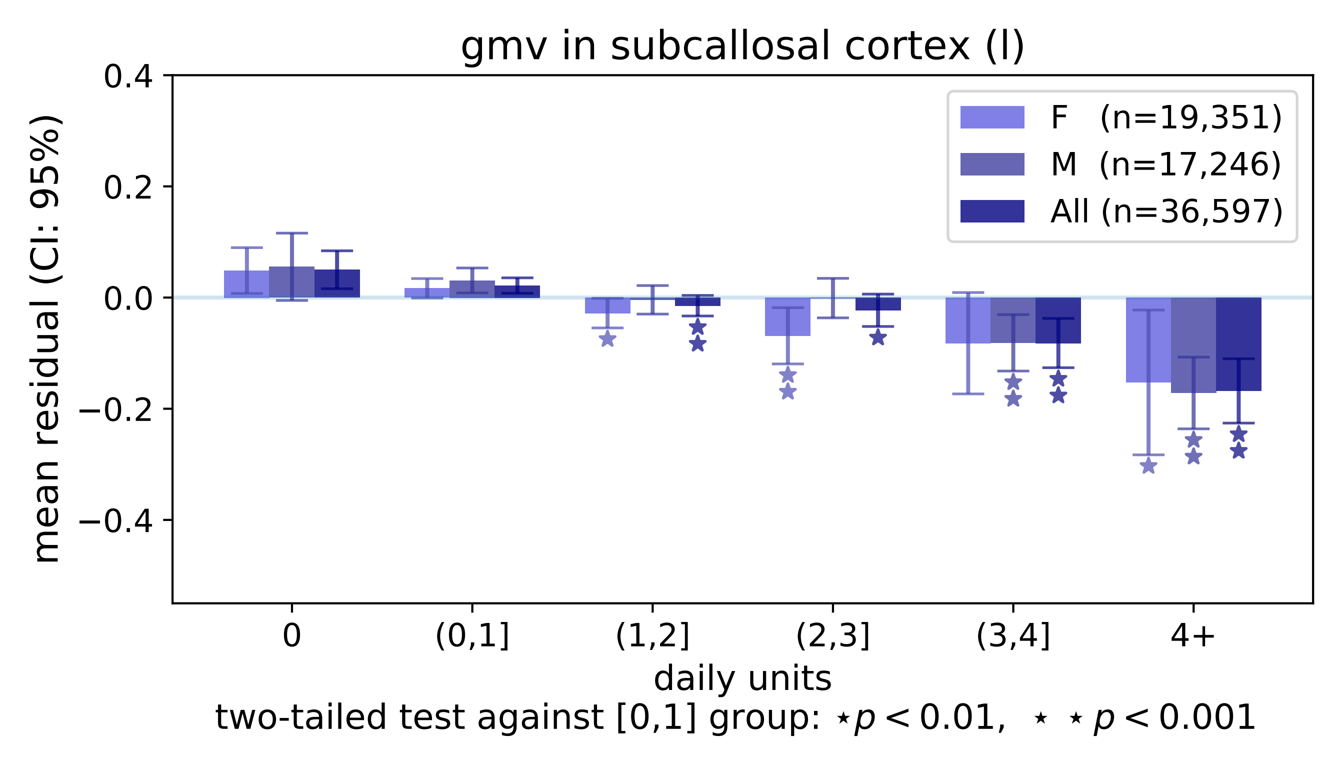
gmv in subcallosal cortex (l)
mean residual (CI: 95%)
0.4
0.2
0.0
−0.2
−0.4
F (n=19,351)
M (n=17,246)
All (n=36,597)
0
(0,1]
(1,2]
(2,3]
(3,4]
4+
daily units
two-tailed test against [0,1] group: ⋆p < 0.01, ⋆ ⋆ p < 0.001

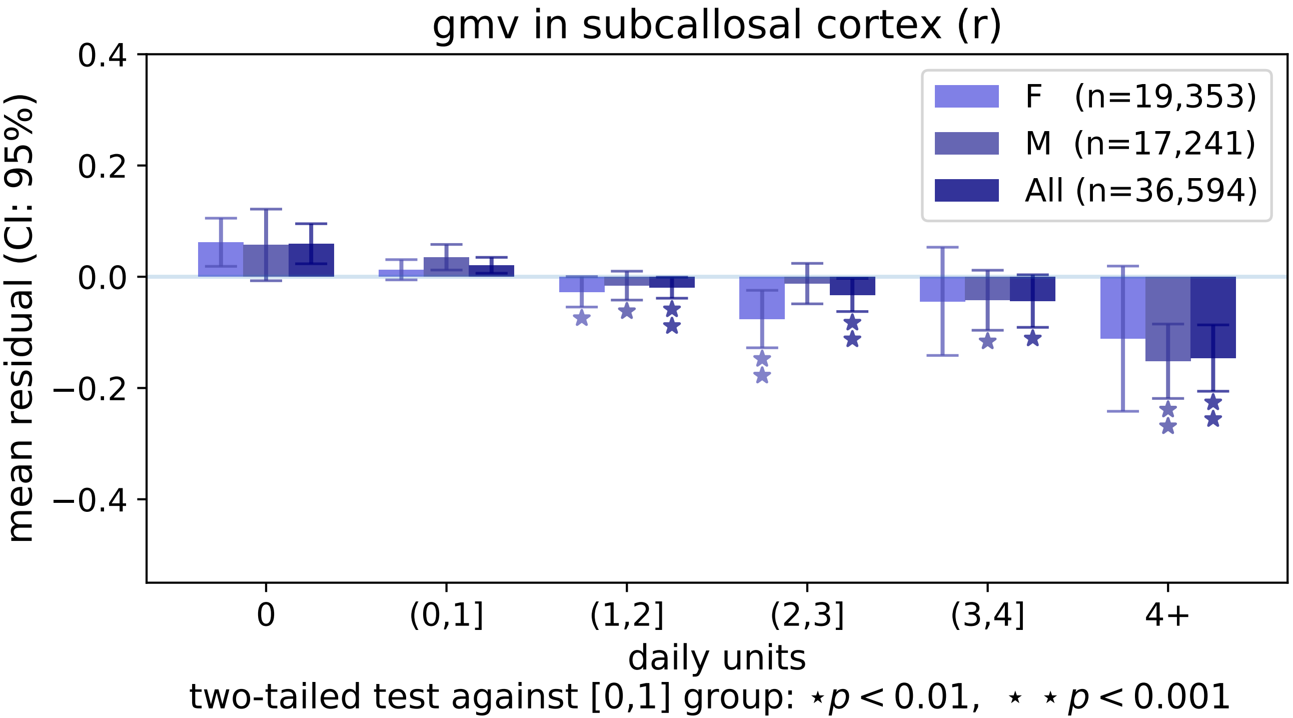

gmv in subcallosal cortex (r)
mean residual (CI: 95%)
0.4
0.2
0.0
−0.2
−0.4
F (n=19,353)
M (n=17,241)
All (n=36,594)
0
(0,1]
(1,2]
(2,3]
(3,4]
4+
daily units
two-tailed test against [0,1] group: ⋆p < 0.01, ⋆ ⋆ p < 0.001

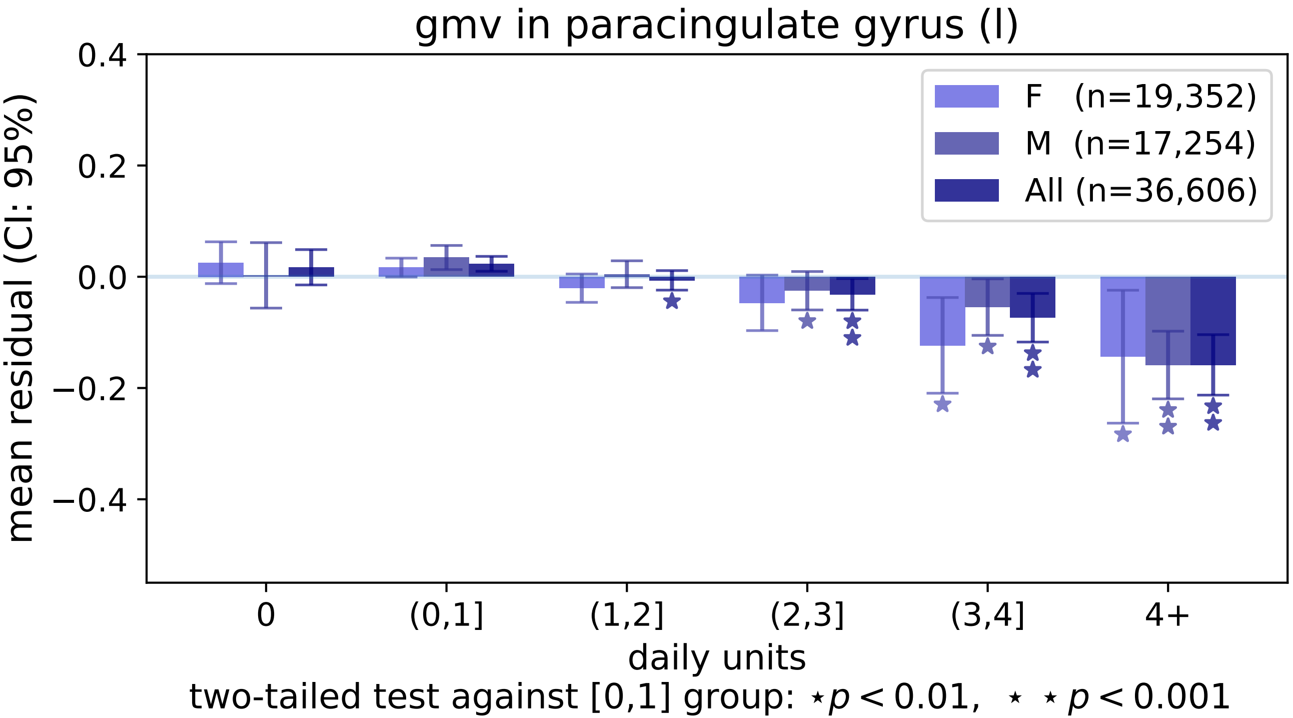
gmv in paracingulate gyrus (l)
mean residual (CI: 95%)
0.4
0.2
0.0
−0.2
−0.4
F (n=19,352)
M (n=17,254)
All (n=36,606)
0
(0,1]
(1,2]
(2,3]
(3,4]
4+
daily units
two-tailed test against [0,1] group: ⋆ $p < 0.01$, ⋆ ⋆ $p < 0.001$

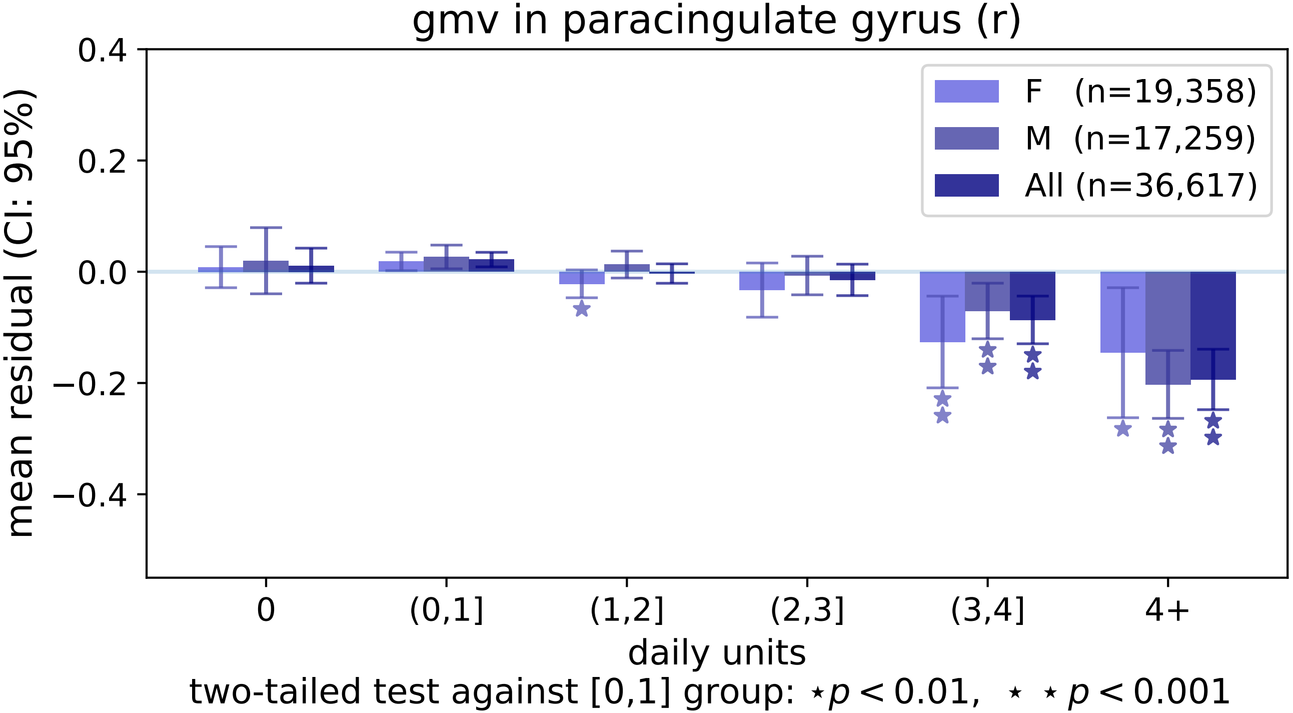
gmv in paracingulate gyrus (r)
mean residual (CI: 95%)
0.4
0.2
0.0
−0.2
−0.4
F (n=19,358)
M (n=17,259)
All (n=36,617)
0
(0,1]
(1,2]
(2,3]
(3,4]
4+
daily units
two-tailed test against [0,1] group: ⋆ p < 0.01, ⋆ ⋆ p < 0.001

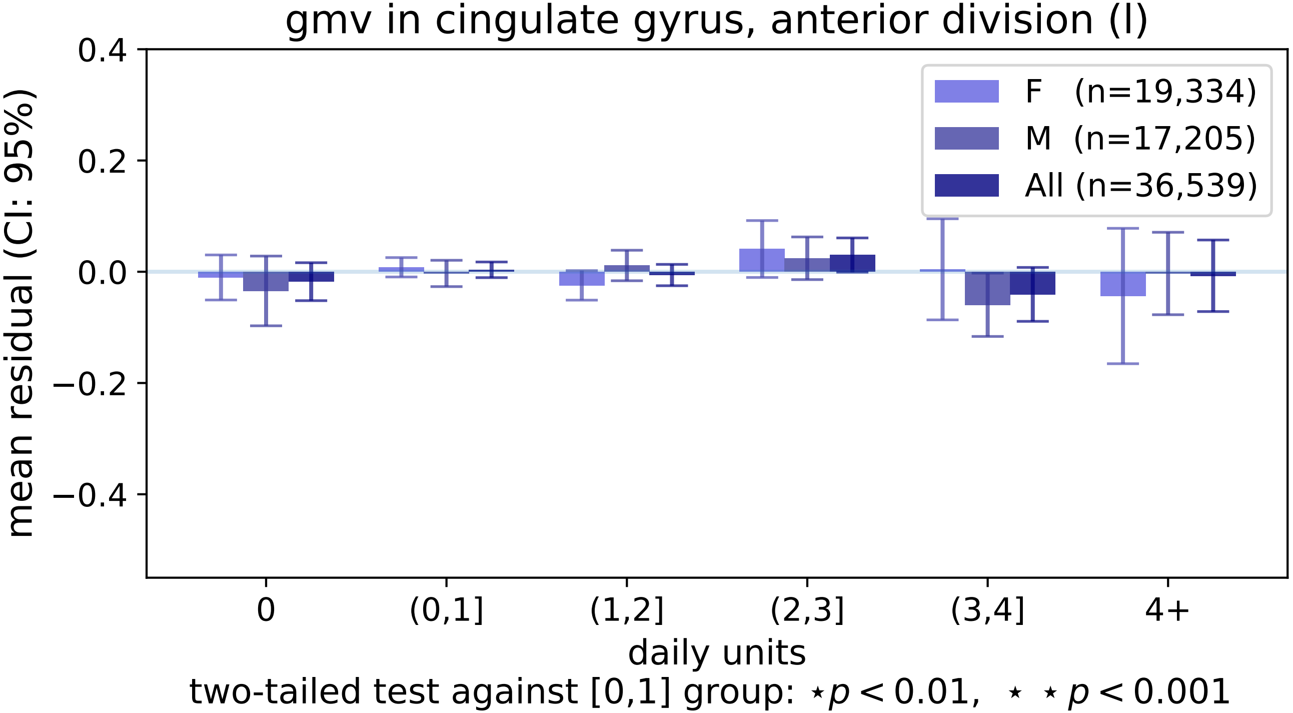
gmv in cingulate gyrus, anterior division (l)
mean residual (CI: 95%)
0.4
0.2
0.0
−0.2
−0.4
F (n=19,334)
M (n=17,205)
All (n=36,539)
0
(0,1]
(1,2]
(2,3]
(3,4]
4+
daily units
two-tailed test against [0,1] group: ⋆p < 0.01, ⋆ ⋆ p < 0.001

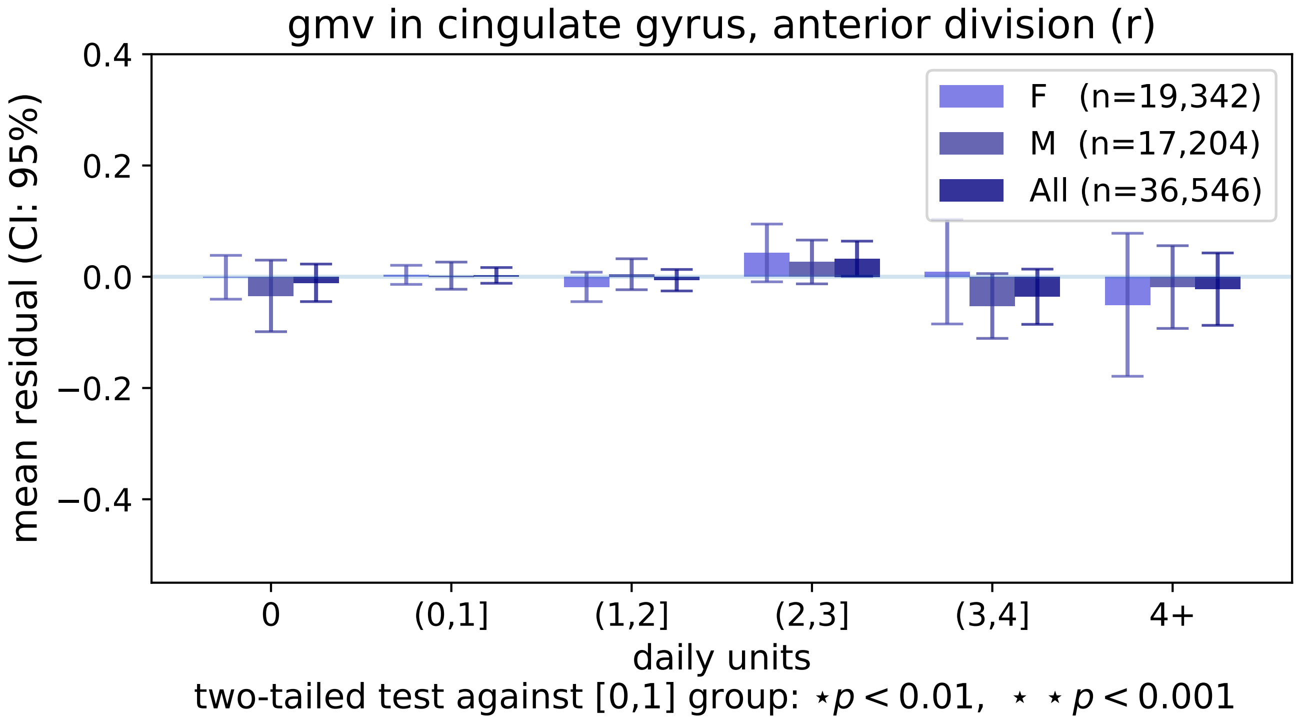

gmv in cingulate gyrus, anterior division (r)
0.4
0.2
0.0
−0.2
−0.4
mean residual (CI: 95%)
F (n=19,342)
M (n=17,204)
All (n=36,546)
0
(0,1]
(1,2]
(2,3]
(3,4]
4+
daily units
two-tailed test against [0,1] group: ⋆p < 0.01, ⋆ ⋆ p < 0.001

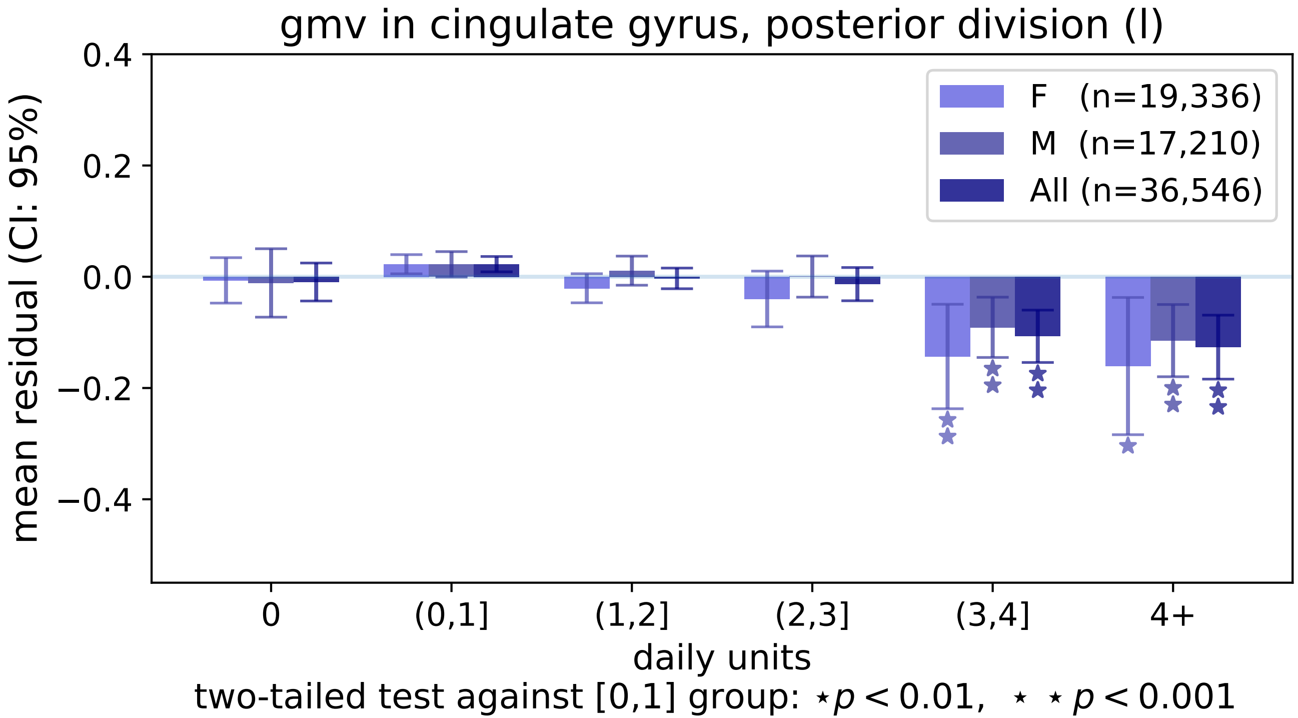

gmv in cingulate gyrus, posterior division (l)
0.4
0.2
0.0
−0.2
−0.4
mean residual (CI: 95%)
F (n=19,336)
M (n=17,210)
All (n=36,546)
0
(0,1]
(1,2]
(2,3]
(3,4]
4+
daily units
two-tailed test against [0,1] group: ⋆ p < 0.01, ⋆ ⋆ p < 0.001

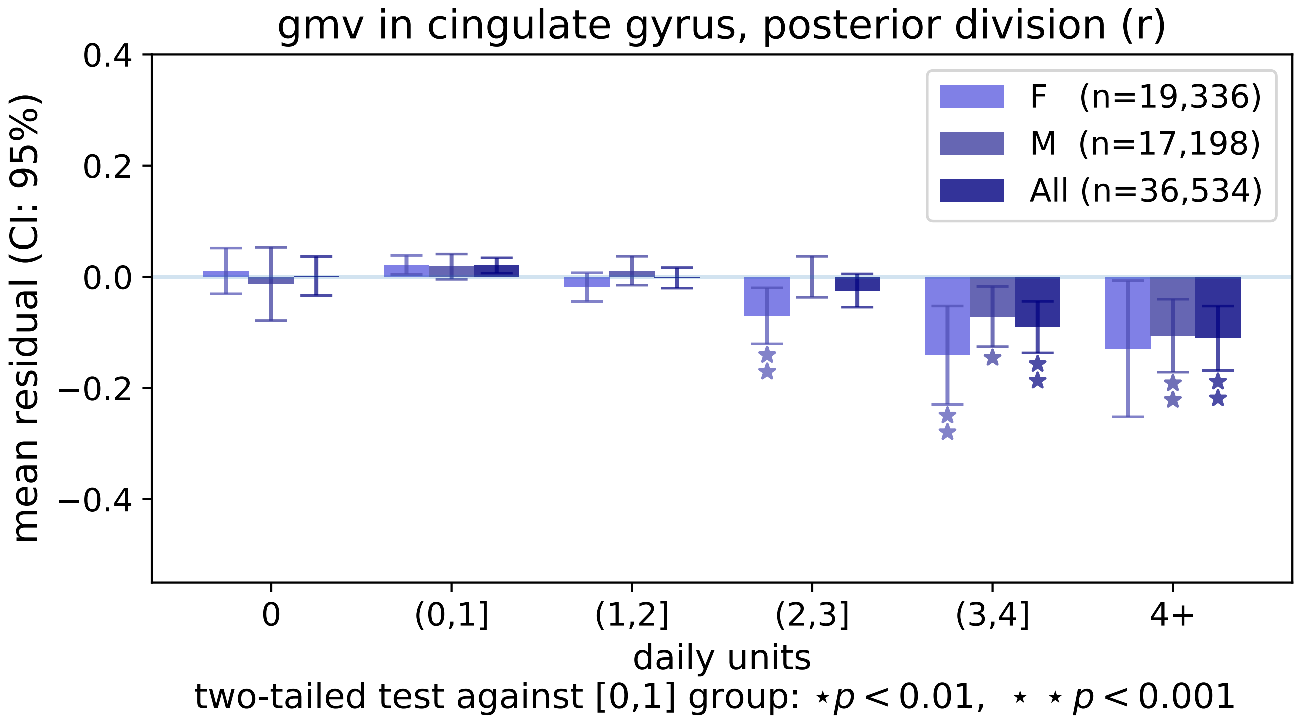
gmv in cingulate gyrus, posterior division (r)
mean residual (CI: 95%)
0.4
0.2
0.0
−0.2
−0.4
F (n=19,336)
M (n=17,198)
All (n=36,534)
0
(0,1]
(1,2]
(2,3]
(3,4]
4+
daily units
two-tailed test against [0,1] group: ⋆ $p < 0.01$, ⋆ ⋆ $p < 0.001$

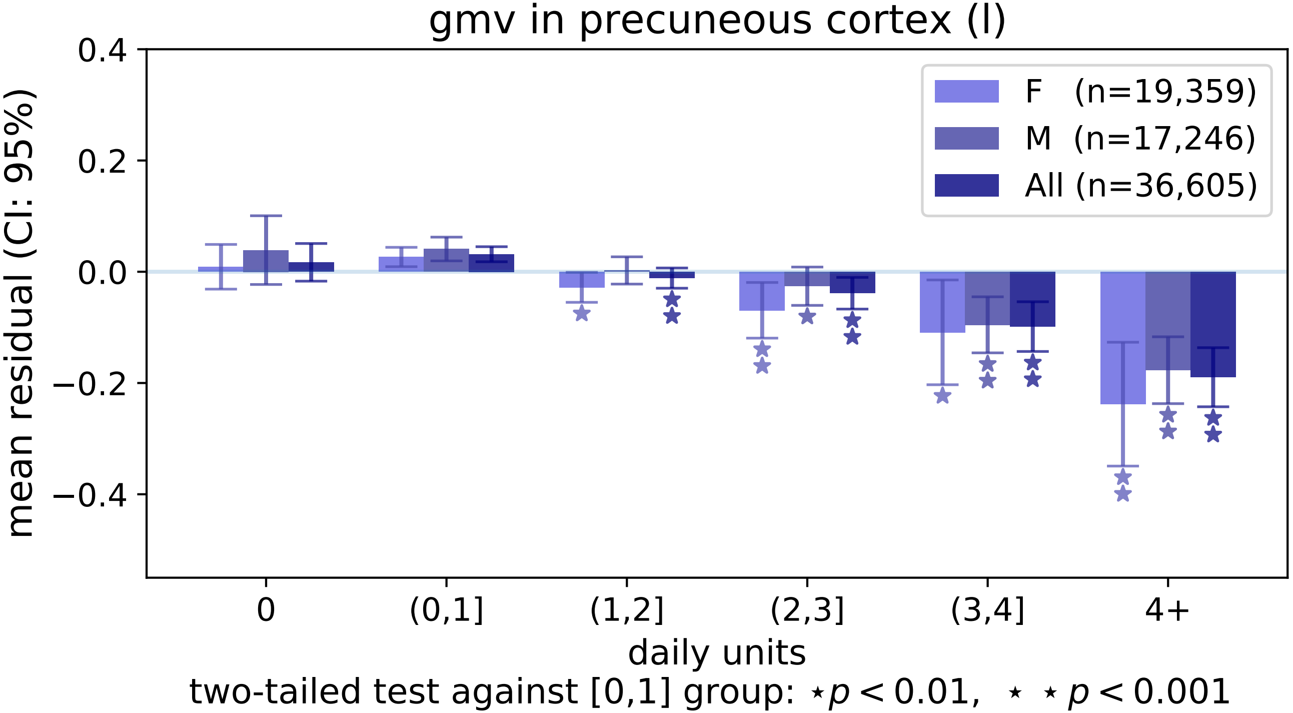

gmv in precuneous cortex (l)
0.4
0.2
0.0
−0.2
−0.4
mean residual (CI: 95%)
F (n=19,359)
M (n=17,246)
All (n=36,605)
0
(0,1]
(1,2]
(2,3]
(3,4]
4+
daily units
two-tailed test against [0,1] group: ⋆ $p < 0.01$, ⋆ ⋆ $p < 0.001$

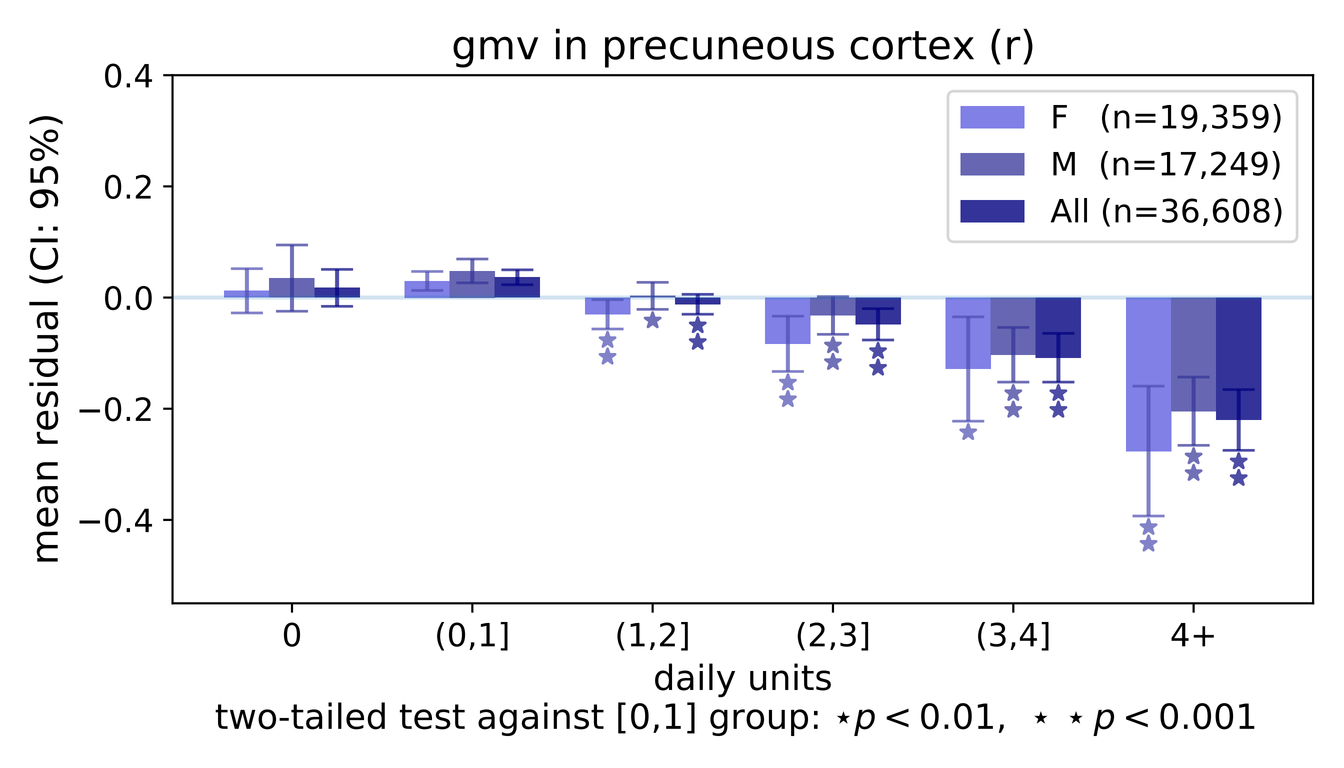

gmv in precuneous cortex (r)
mean residual (CI: 95%)
0.4
0.2
0.0
−0.2
−0.4
F (n=19,359)
M (n=17,249)
All (n=36,608)
0
(0,1]
(1,2]
(2,3]
(3,4]
4+
daily units
two-tailed test against [0,1] group: ⋆p < 0.01, ⋆ ⋆ p < 0.001

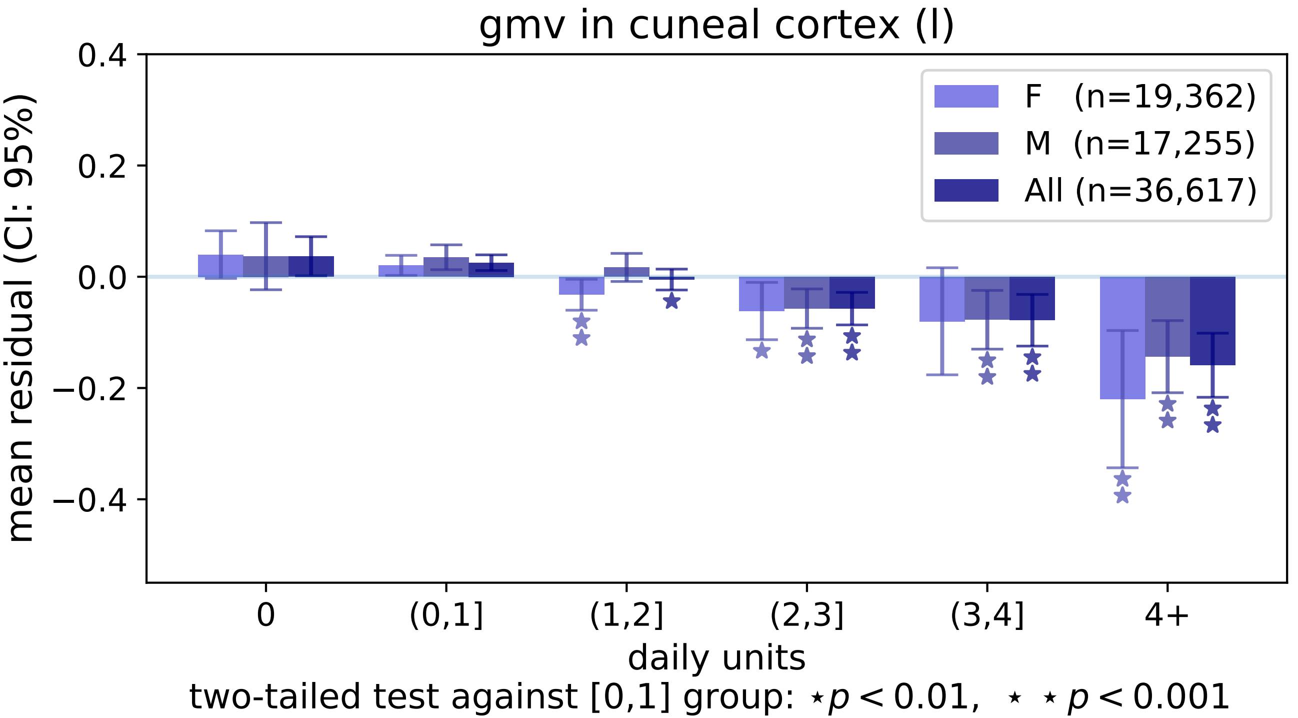

gmv in cuneal cortex (l)
mean residual (CI: 95%)
0.4
0.2
0.0
−0.2
−0.4
F (n=19,362)
M (n=17,255)
All (n=36,617)
0
(0,1]
(1,2]
(2,3]
(3,4]
4+
daily units
two-tailed test against [0,1] group: ⋆ p < 0.01, ⋆ ⋆ p < 0.001

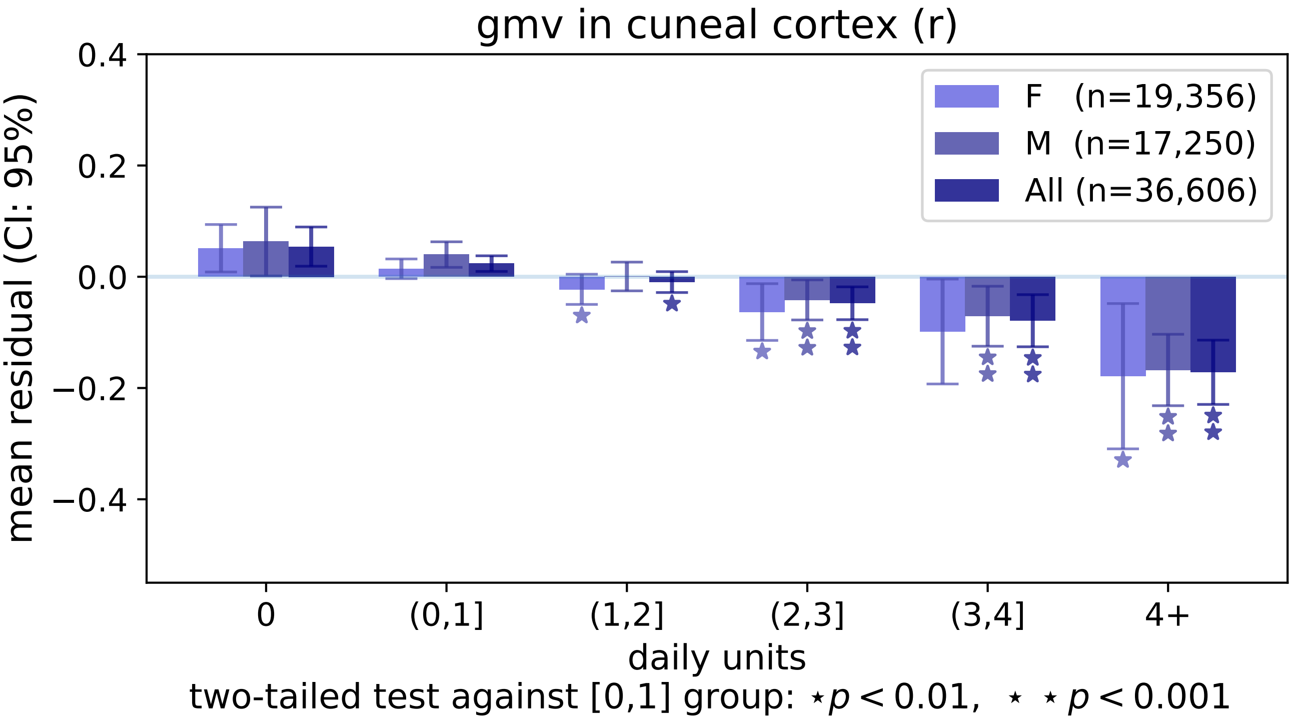
gmv in cuneal cortex (r)
0.4
0.2
0.0
−0.2
−0.4
mean residual (CI: 95%)
F (n=19,356)
M (n=17,250)
All (n=36,606)
0
(0,1]
(1,2]
(2,3]
(3,4]
4+
daily units
two-tailed test against [0,1] group: ⋆p < 0.01, ⋆ ⋆ p < 0.001

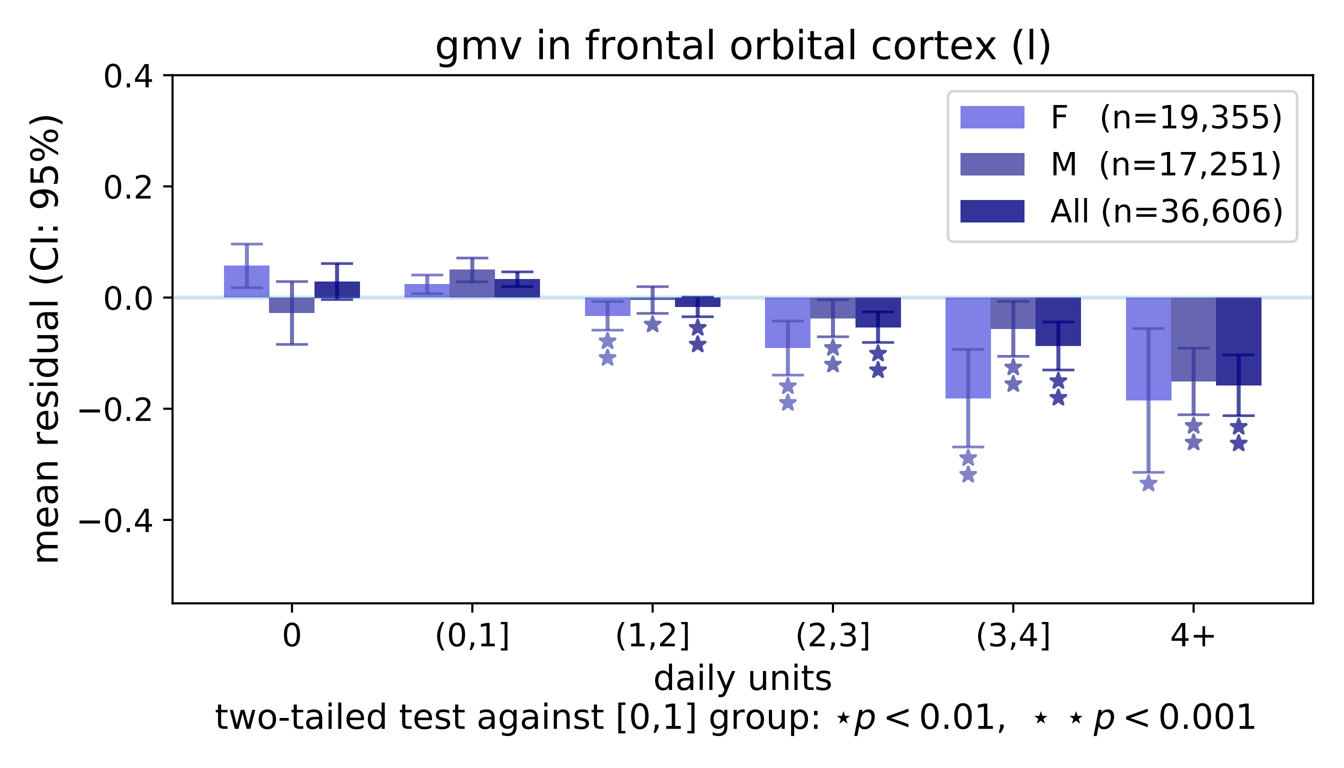
gmv in frontal orbital cortex (l)
mean residual (CI: 95%)
0.4
0.2
0.0
−0.2
−0.4
F (n=19,355)
M (n=17,251)
All (n=36,606)
0
(0,1]
(1,2]
(2,3]
(3,4]
4+
daily units
two-tailed test against [0,1] group: ⋆p < 0.01, ⋆ ⋆p < 0.001

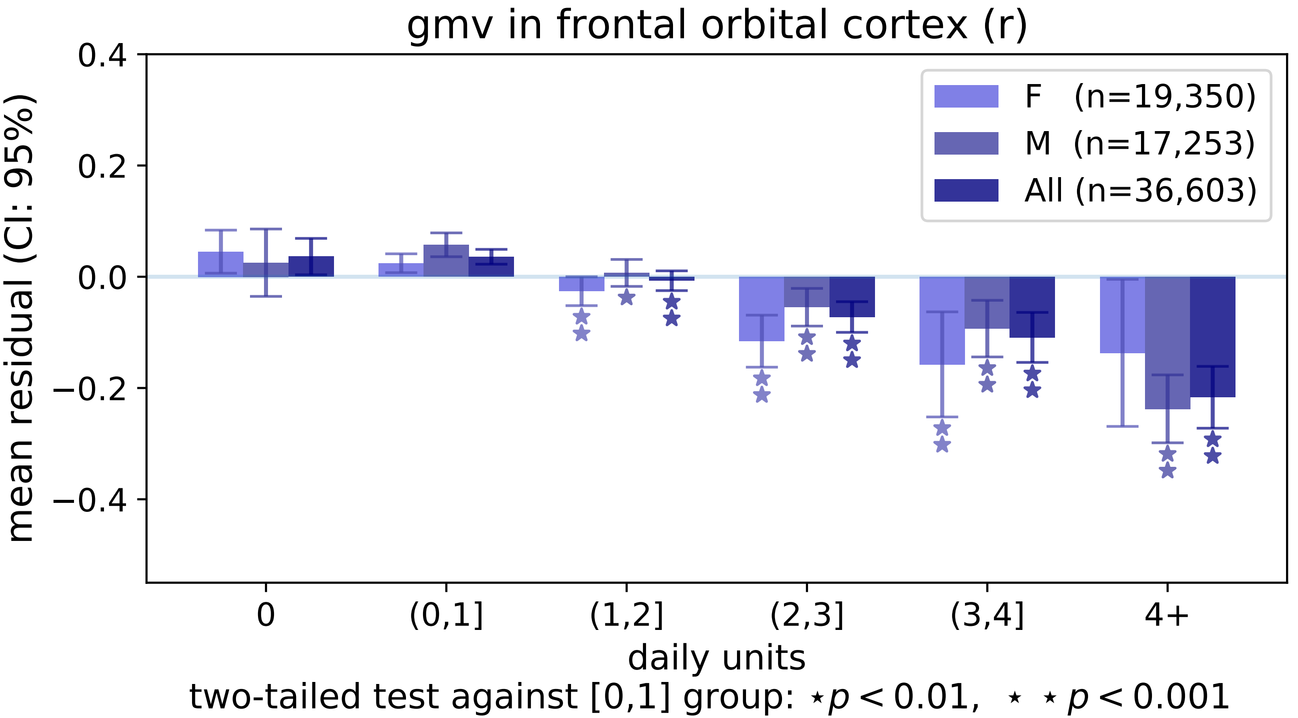

gmv in frontal orbital cortex (r)
F (n=19,350)
M (n=17,253)
All (n=36,603)
mean residual (CI: 95%)
0.4
0.2
0.0
−0.2
−0.4
0
(0,1]
(1,2]
(2,3]
(3,4]
4+
daily units
two-tailed test against [0,1] group: ⋆p < 0.01, ⋆ ⋆ p < 0.001

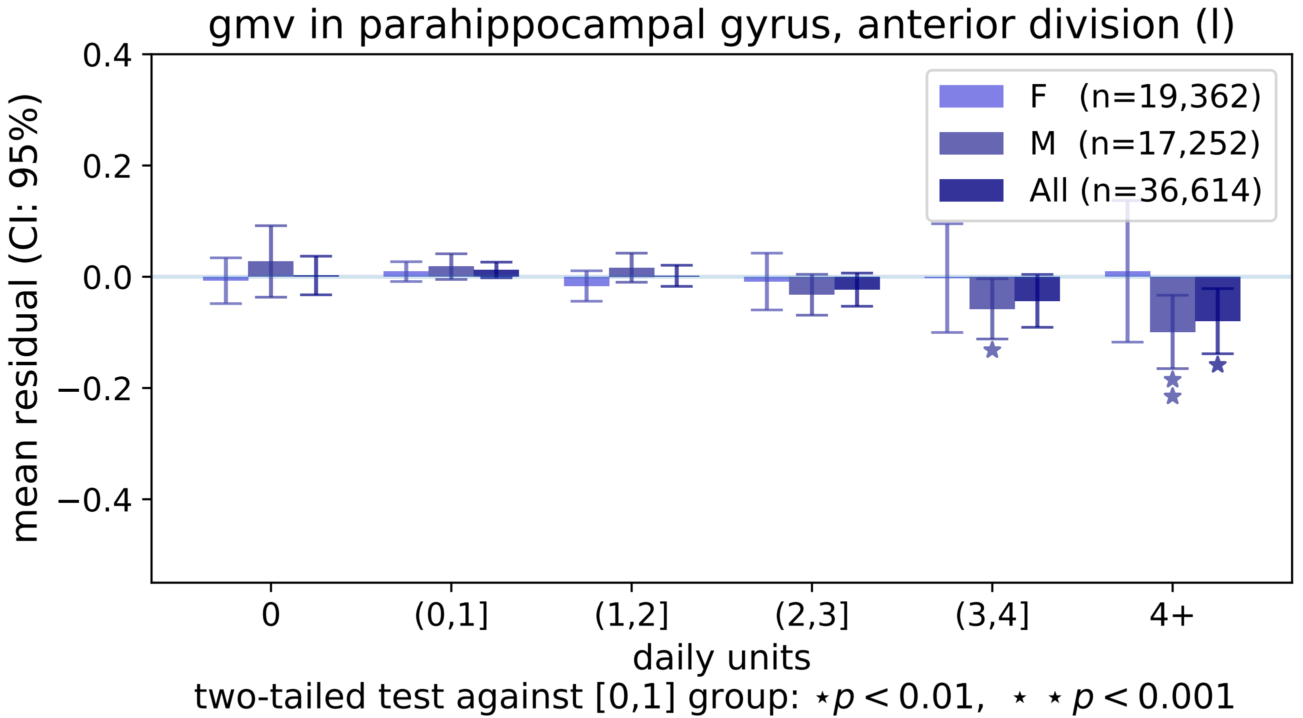
gmv in parahippocampal gyrus, anterior division (l)
mean residual (CI: 95%)
0.4
0.2
0.0
−0.2
−0.4
F (n=19,362)
M (n=17,252)
All (n=36,614)
0
(0,1]
(1,2]
(2,3]
(3,4]
4+
daily units
two-tailed test against [0,1] group: ⋆p < 0.01, ⋆ ⋆ p < 0.001

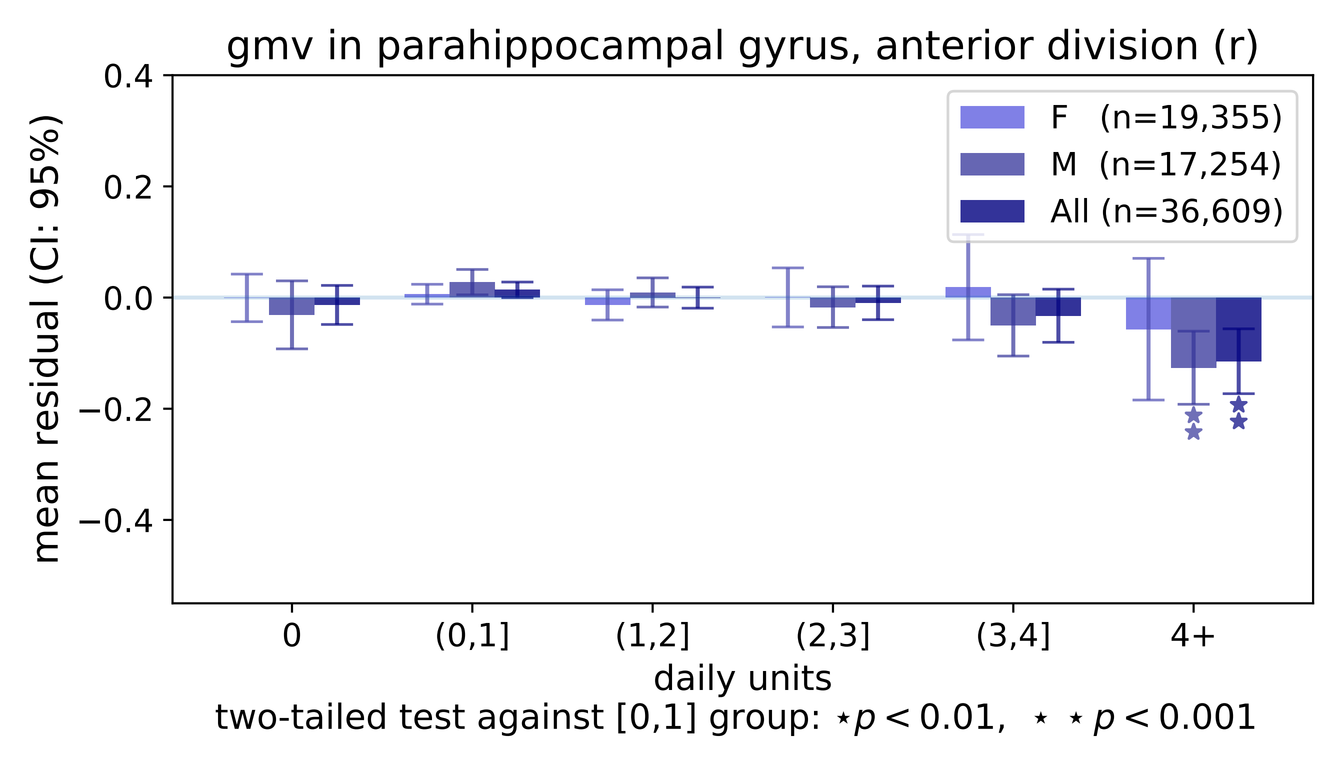
gmv in parahippocampal gyrus, anterior division (r)
F (n=19,355)
M (n=17,254)
All (n=36,609)
mean residual (CI: 95%)
0.4
0.2
0.0
−0.2
−0.4
0
(0,1]
(1,2]
(2,3]
(3,4]
4+
daily units
two-tailed test against [0,1] group: ⋆ p < 0.01, ⋆ ⋆ p < 0.001

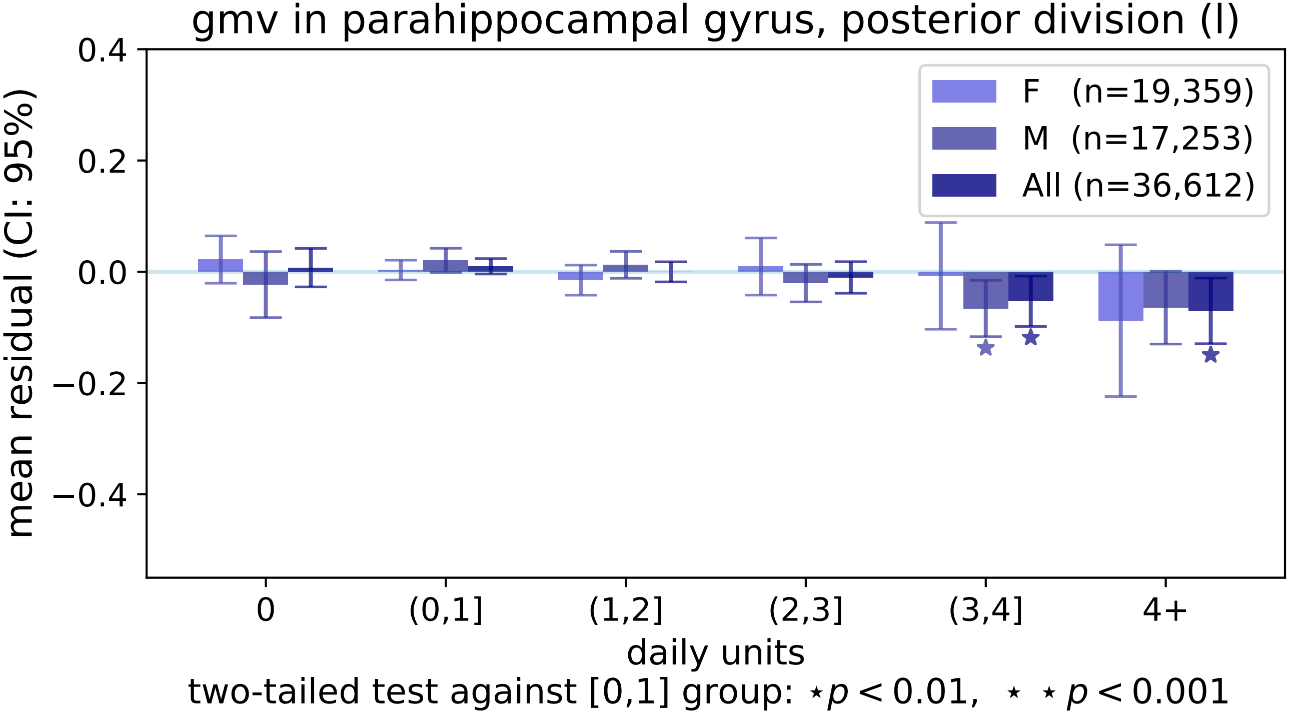
gmv in parahippocampal gyrus, posterior division (l)
mean residual (CI: 95%)
0.4
0.2
0.0
−0.2
−0.4
F (n=19,359)
M (n=17,253)
All (n=36,612)
0
(0,1]
(1,2]
(2,3]
(3,4]
4+
daily units
two-tailed test against [0,1] group: ⋆p < 0.01, ⋆ ⋆ p < 0.001

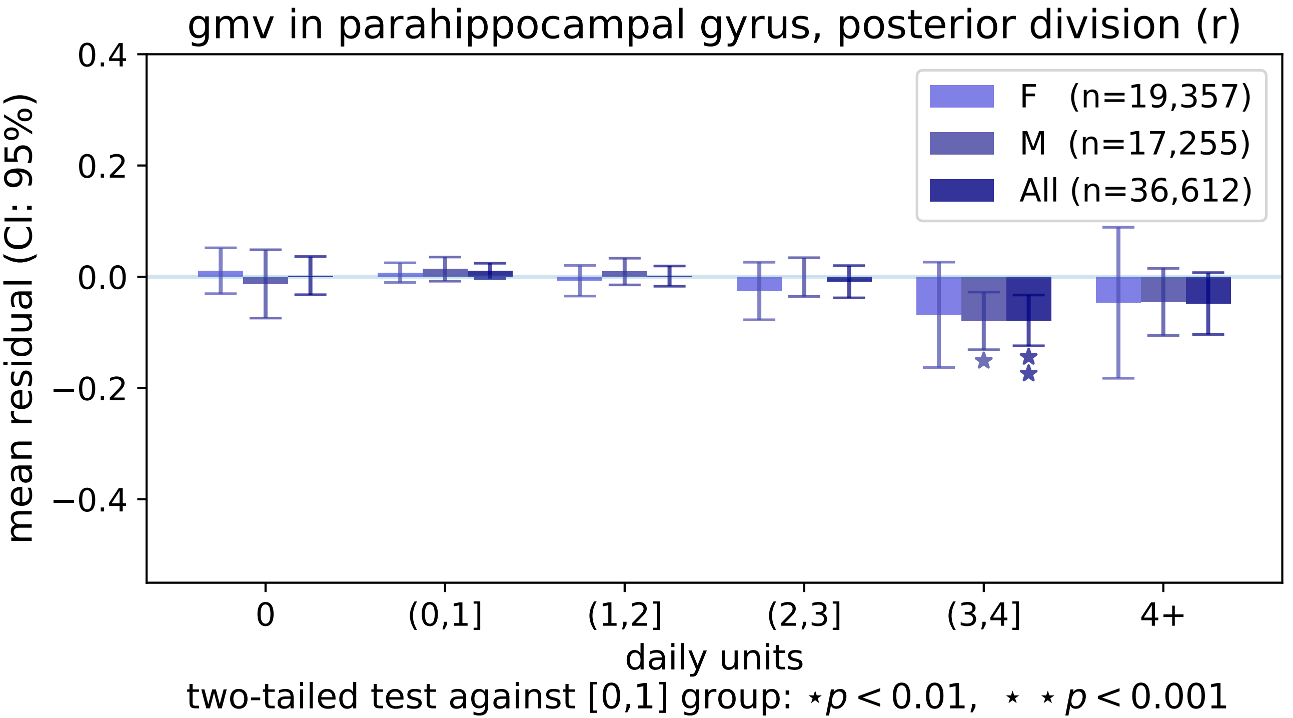

gmv in parahippocampal gyrus, posterior division (r)
0.4
0.2
0.0
−0.2
−0.4
mean residual (CI: 95%)
F (n=19,357)
M (n=17,255)
All (n=36,612)
0
(0,1]
(1,2]
(2,3]
(3,4]
4+
daily units
two-tailed test against [0,1] group: ⋆$p < 0.01$, ⋆ ⋆$p < 0.001$

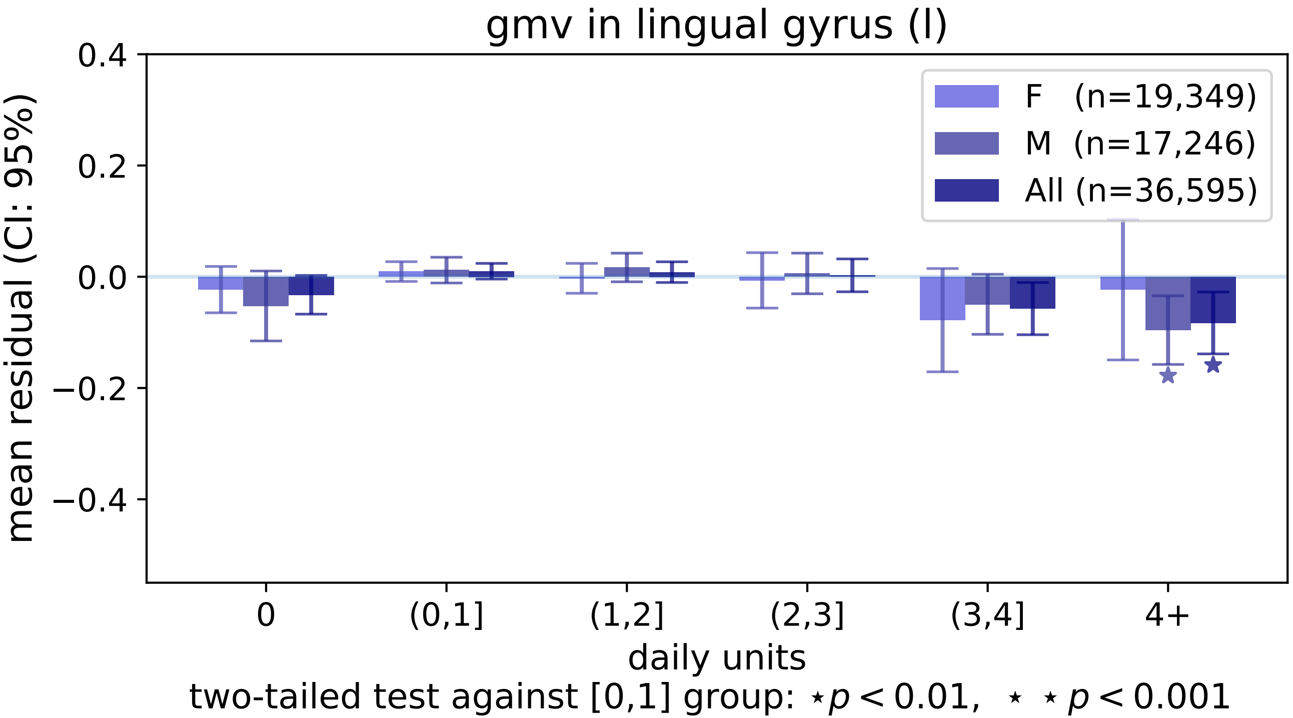
gmv in lingual gyrus (l)
mean residual (CI: 95%)
0.4
0.2
0.0
−0.2
−0.4
F (n=19,349)
M (n=17,246)
All (n=36,595)
0
(0,1]
(1,2]
(2,3]
(3,4]
4+
daily units
two-tailed test against [0,1] group: ⋆p < 0.01, ⋆ ⋆ p < 0.001

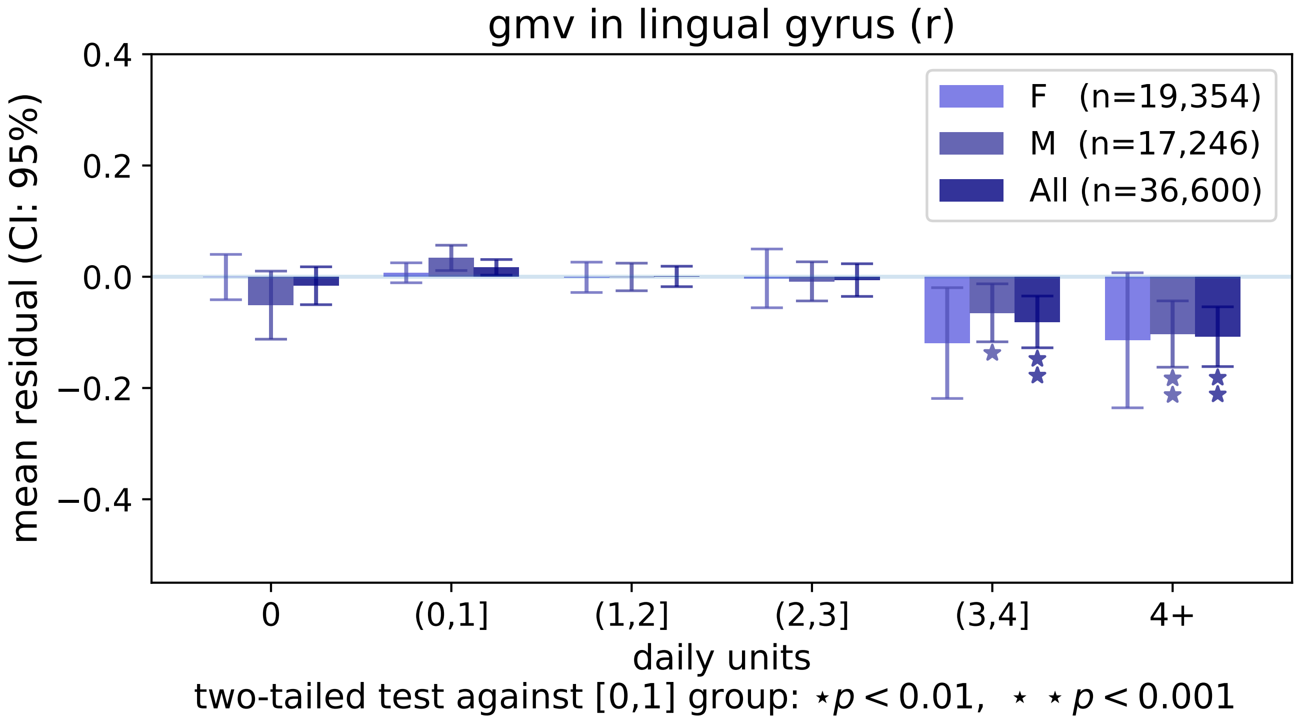

gmv in lingual gyrus (r)
0.4
0.2
0.0
−0.2
−0.4
mean residual (CI: 95%)
F (n=19,354)
M (n=17,246)
All (n=36,600)
0
(0,1]
(1,2]
(2,3]
(3,4]
4+
daily units
two-tailed test against [0,1] group: ⋆p < 0.01, ⋆ ⋆ p < 0.001

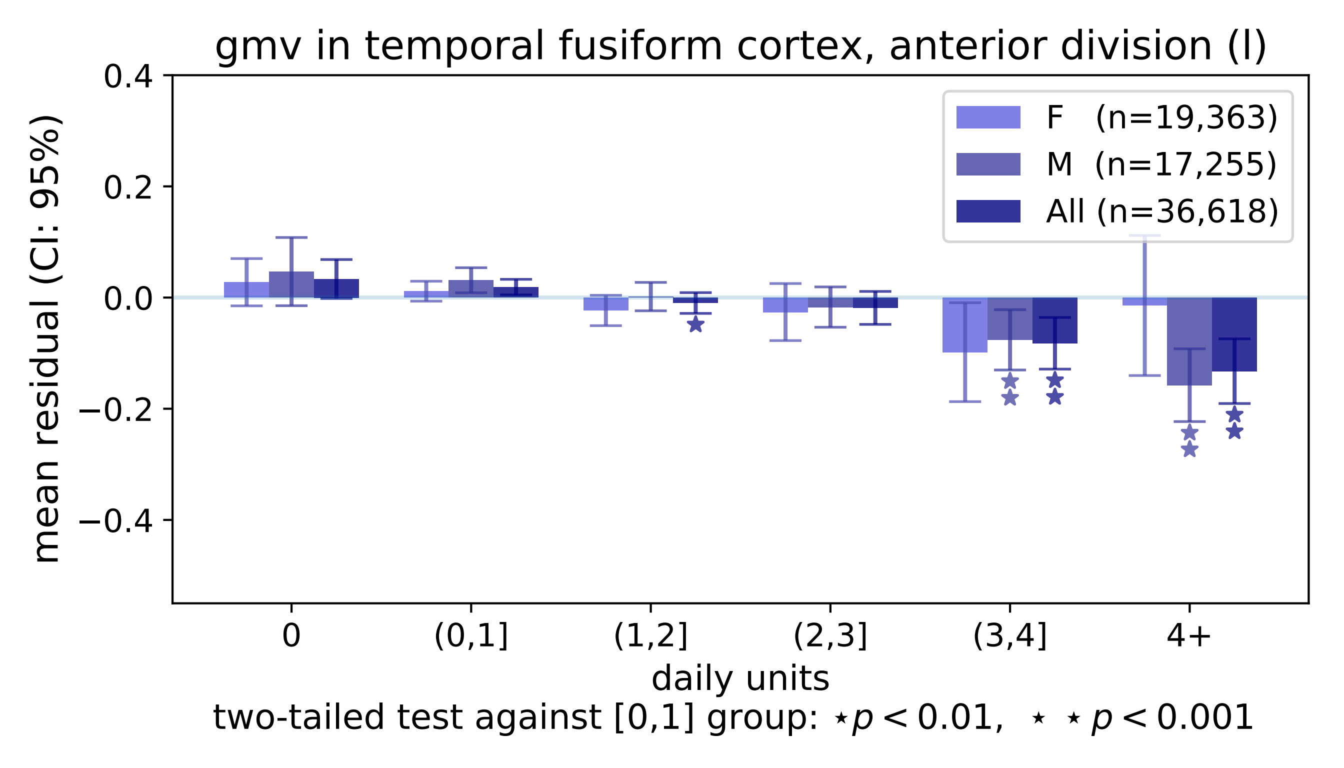
gmv in temporal fusiform cortex, anterior division (l)
0.4
0.2
0.0
−0.2
−0.4
mean residual (CI: 95%)
F (n=19,363)
M (n=17,255)
All (n=36,618)
0
(0,1]
(1,2]
(2,3]
(3,4]
4+
daily units
two-tailed test against [0,1] group: ⋆ p < 0.01, ⋆ ⋆ p < 0.001

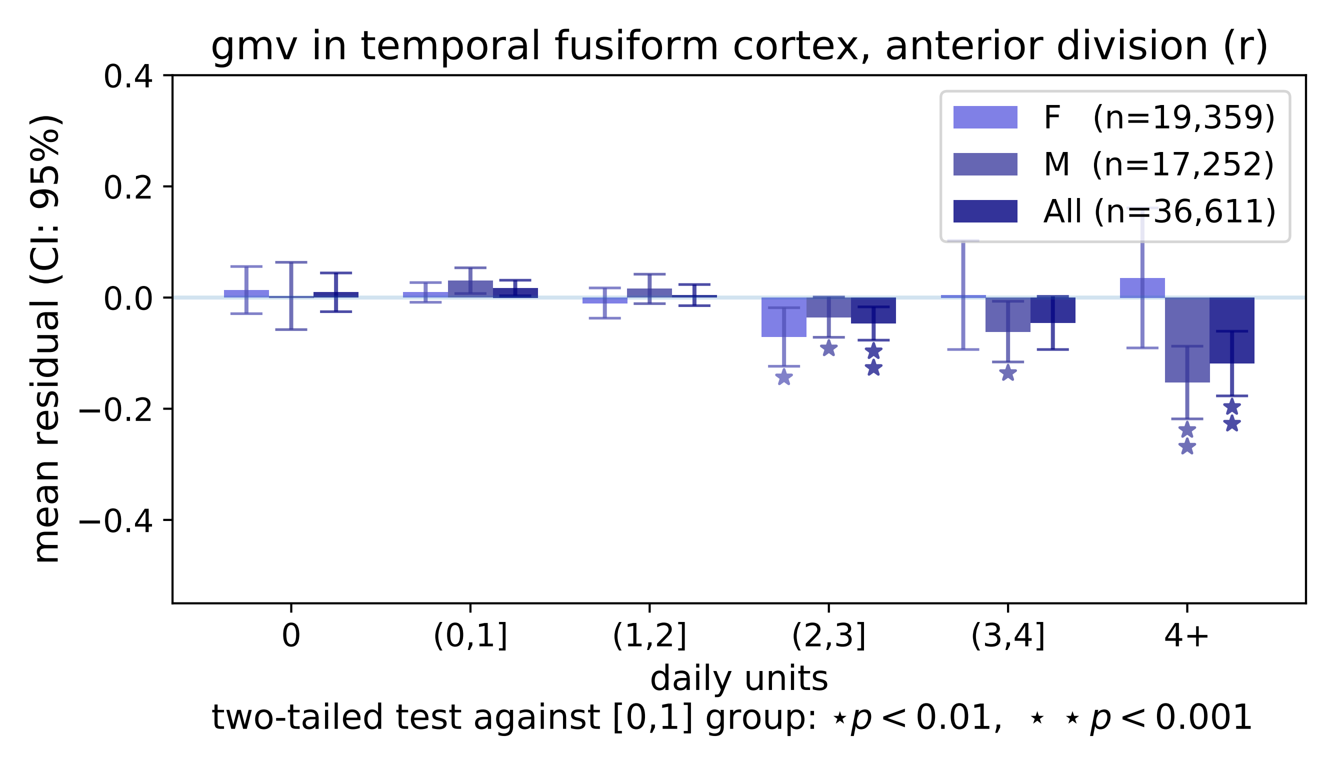

gmv in temporal fusiform cortex, anterior division (r)
mean residual (CI: 95%)
0.4
0.2
0.0
−0.2
−0.4
F (n=19,359)
M (n=17,252)
All (n=36,611)
0
(0,1]
(1,2]
(2,3]
(3,4]
4+
daily units
two-tailed test against [0,1] group: ⋆p < 0.01, ⋆ ⋆ p < 0.001

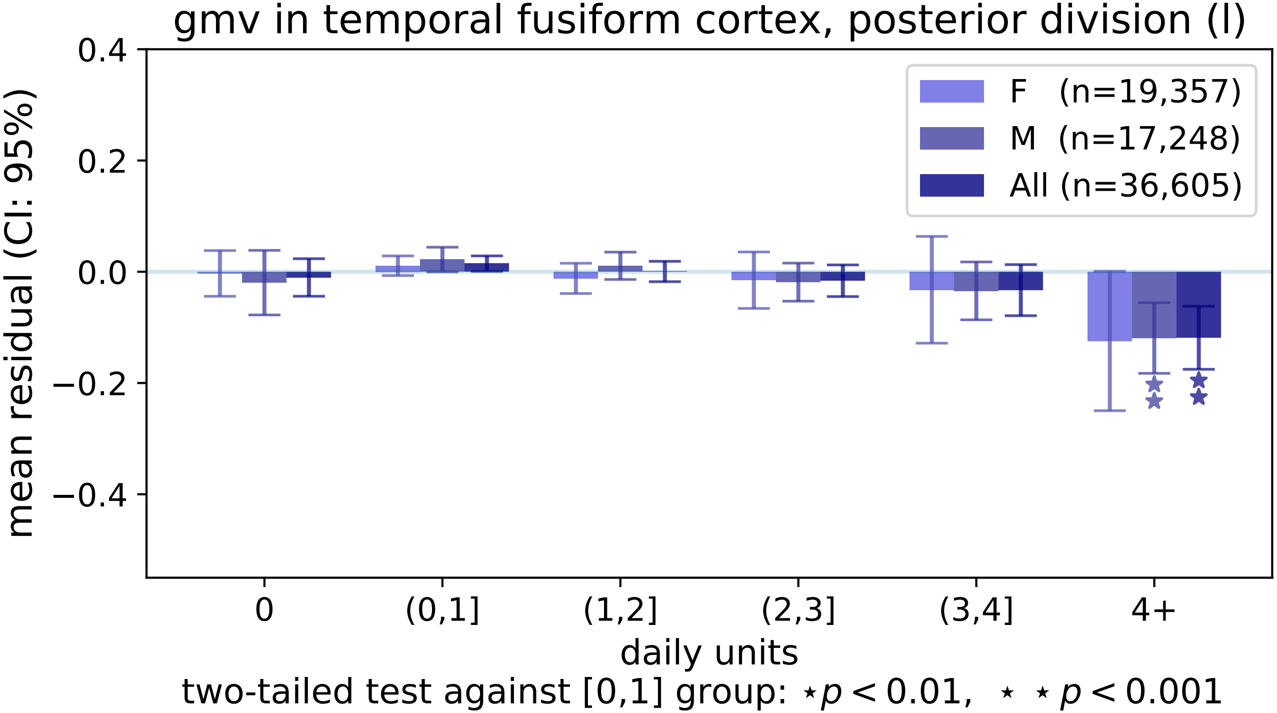

gmv in temporal fusiform cortex, posterior division (l)
mean residual (CI: 95%)
0.4
0.2
0.0
−0.2
−0.4
F (n=19,357)
M (n=17,248)
All (n=36,605)
0
(0,1]
(1,2]
(2,3]
(3,4]
4+
daily units
two-tailed test against [0,1] group: ⋆p < 0.01, ⋆ ⋆ p < 0.001

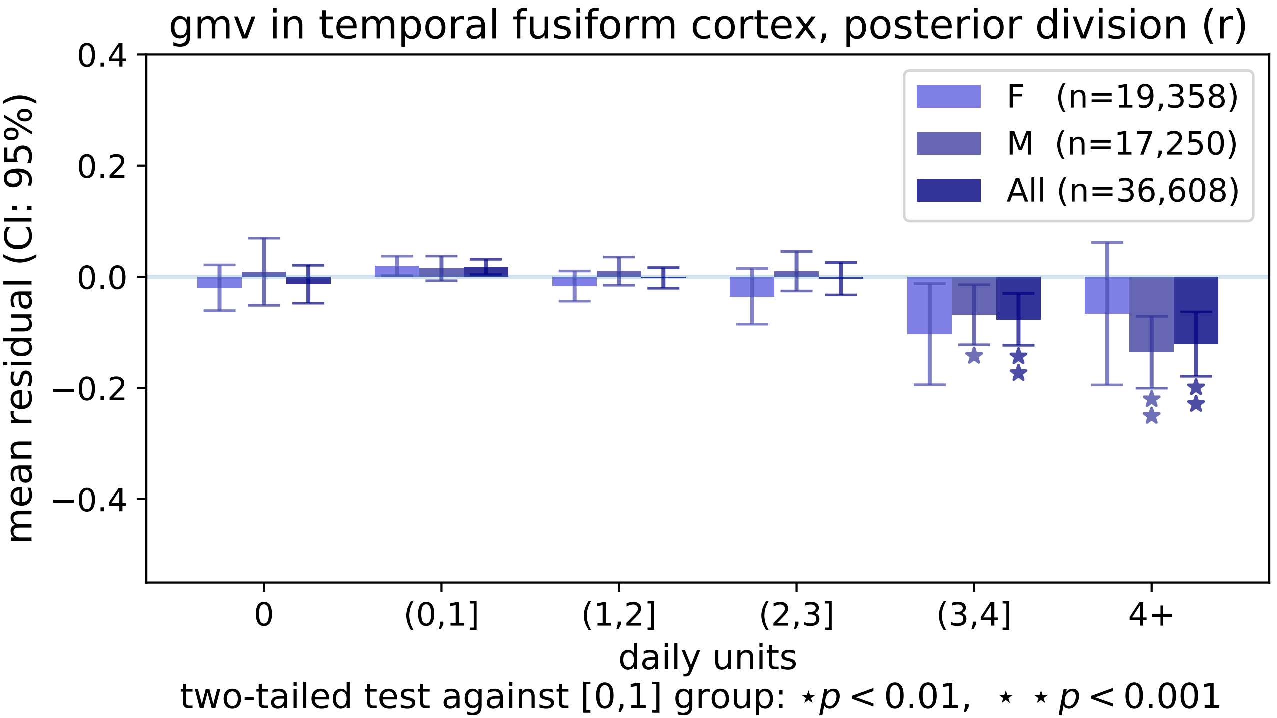

gmv in temporal fusiform cortex, posterior division (r)
mean residual (CI: 95%)
0.4
0.2
0.0
−0.2
−0.4
F (n=19,358)
M (n=17,250)
All (n=36,608)
0
(0,1]
(1,2]
(2,3]
(3,4]
4+
daily units
two-tailed test against [0,1] group: ⋆ p < 0.01, ⋆ ⋆ p < 0.001

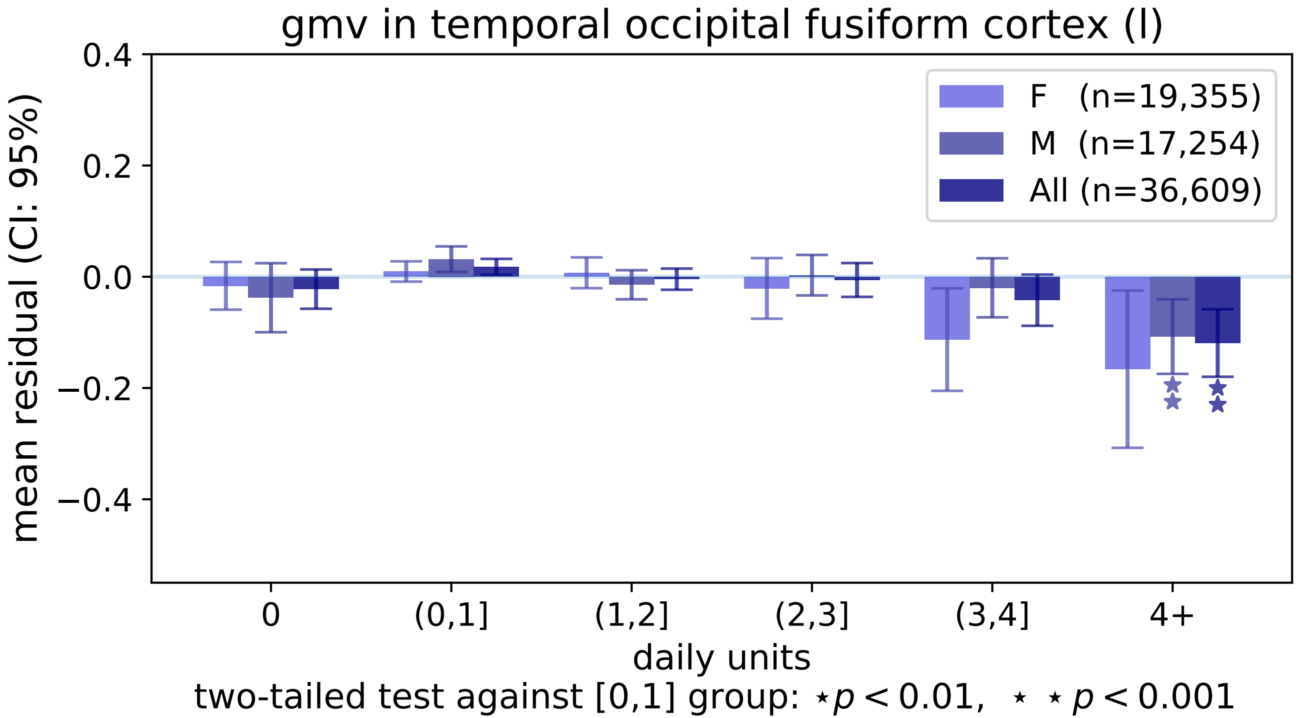
gmv in temporal occipital fusiform cortex (l)
0.4
0.2
0.0
−0.2
−0.4
mean residual (CI: 95%)
F (n=19,355)
M (n=17,254)
All (n=36,609)
0
(0,1]
(1,2]
(2,3]
(3,4]
4+
daily units
two-tailed test against [0,1] group: ⋆p < 0.01, ⋆ ⋆ p < 0.001

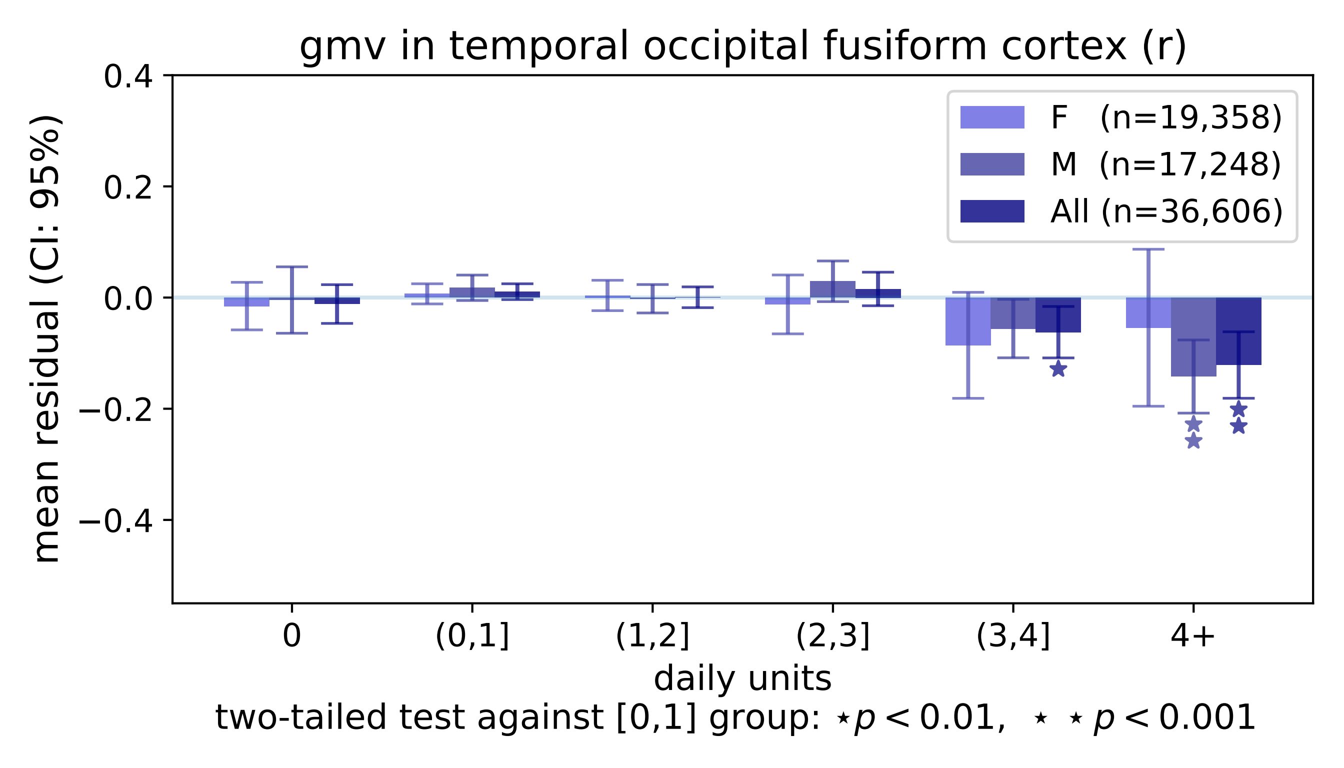
gmv in temporal occipital fusiform cortex (r)
mean residual (CI: 95%)
0.4
0.2
0.0
−0.2
−0.4
F (n=19,358)
M (n=17,248)
All (n=36,606)
0
(0,1]
(1,2]
(2,3]
(3,4]
4+
daily units
two-tailed test against [0,1] group: ⋆p < 0.01, ⋆ ⋆p < 0.001

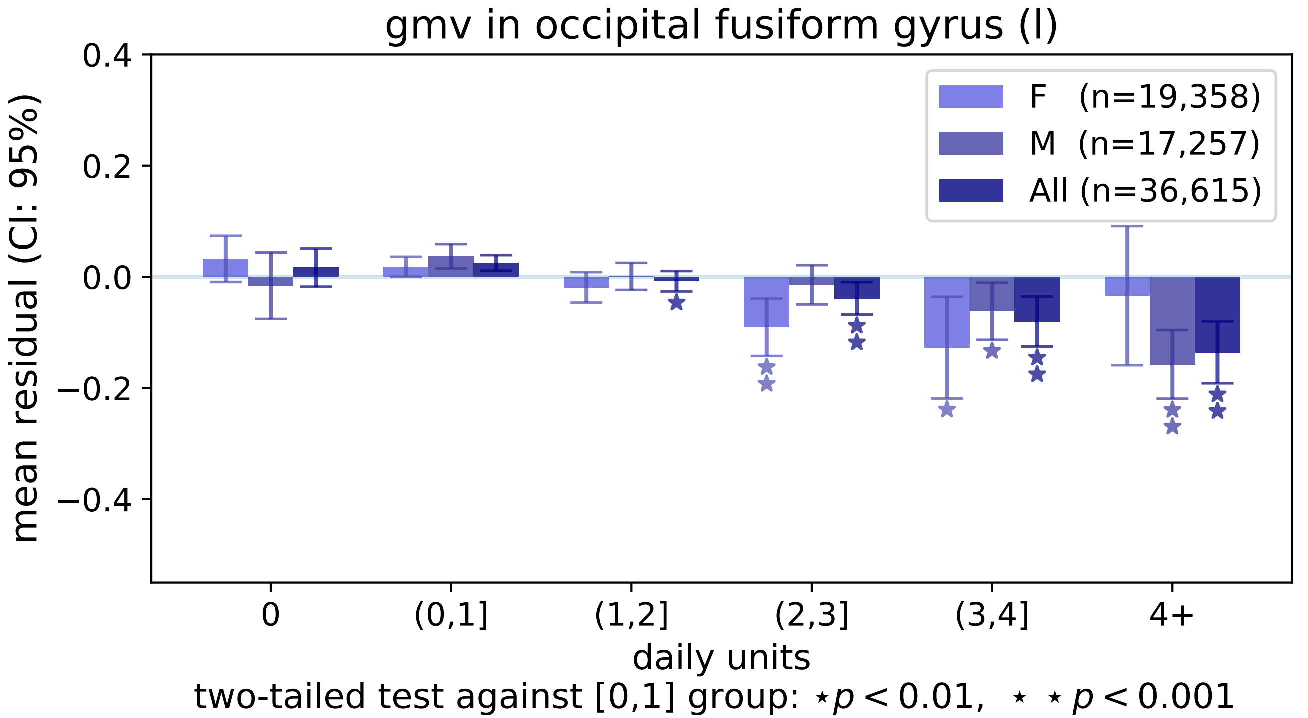
gmv in occipital fusiform gyrus (l)
0.4
0.2
0.0
−0.2
−0.4
mean residual (CI: 95%)
F (n=19,358)
M (n=17,257)
All (n=36,615)
0
(0,1]
(1,2]
(2,3]
(3,4]
4+
daily units
two-tailed test against [0,1] group: ⋆$p < 0.01$, ⋆ ⋆ $p < 0.001$

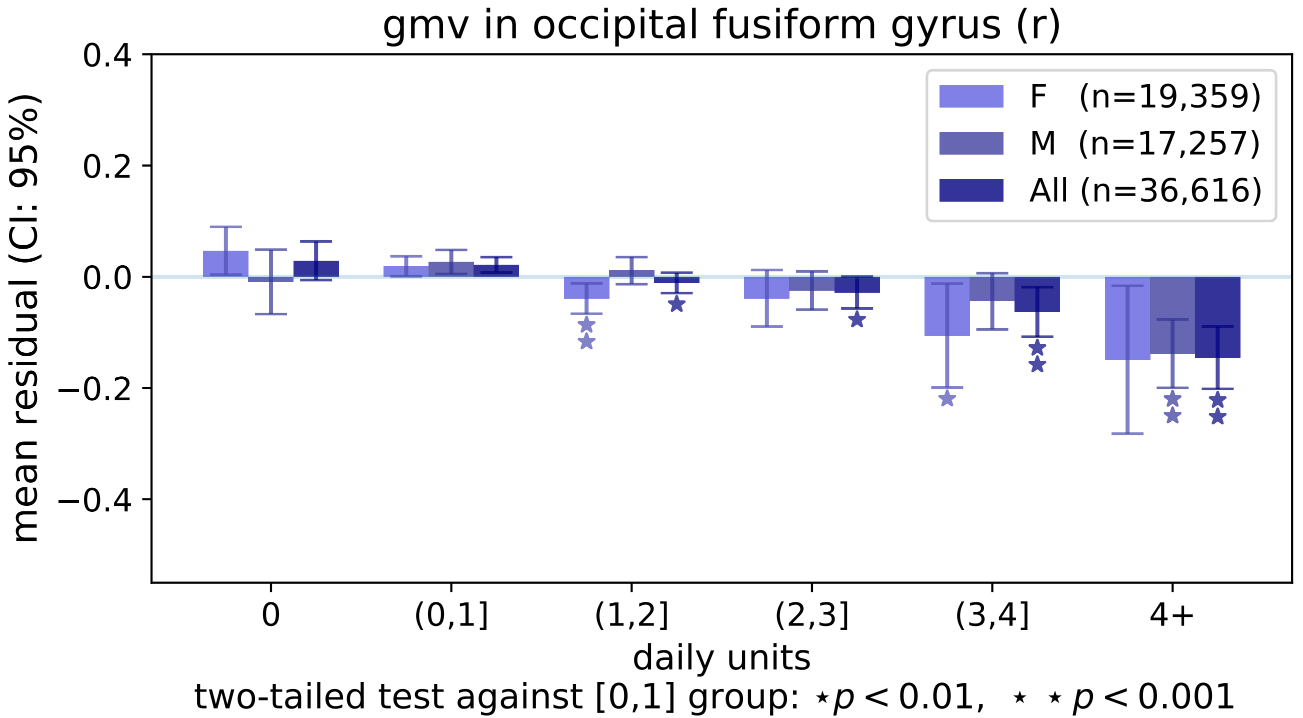
gmv in occipital fusiform gyrus (r)
0.4
0.2
0.0
−0.2
−0.4
mean residual (CI: 95%)
F (n=19,359)
M (n=17,257)
All (n=36,616)
0
(0,1]
(1,2]
(2,3]
(3,4]
4+
daily units
two-tailed test against [0,1] group: ⋆p < 0.01, ⋆ ⋆ p < 0.001

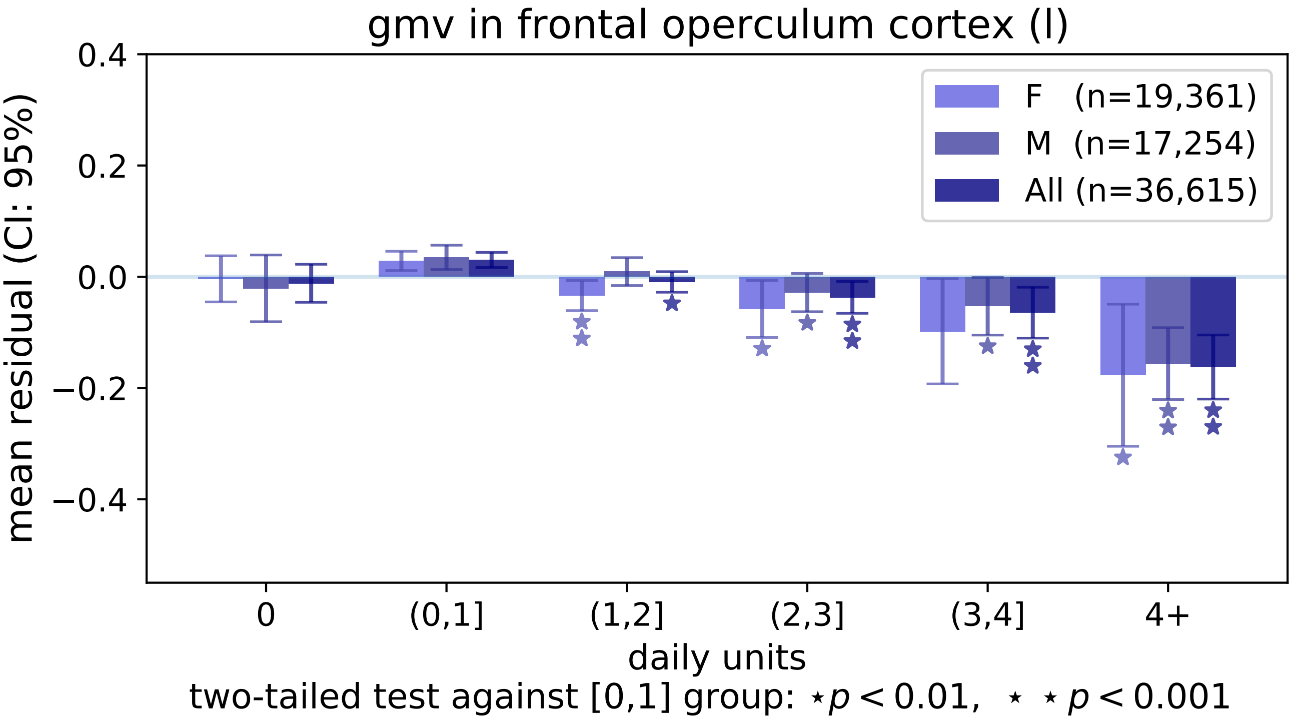
gmv in frontal operculum cortex (l)
mean residual (CI: 95%)
0.4
0.2
0.0
−0.2
−0.4
F (n=19,361)
M (n=17,254)
All (n=36,615)
0
(0,1]
(1,2]
(2,3]
(3,4]
4+
daily units
two-tailed test against [0,1] group: ⋆p < 0.01, ⋆ ⋆ p < 0.001

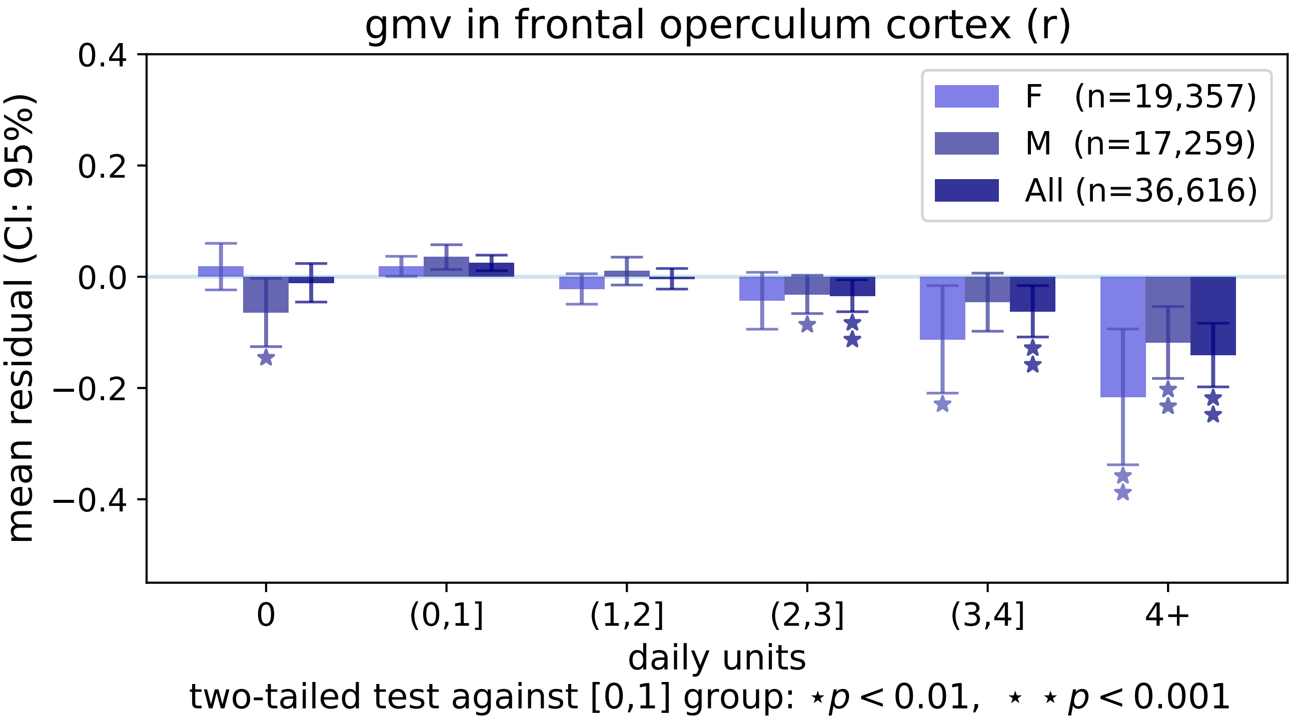

gmv in frontal operculum cortex (r)
mean residual (CI: 95%)
0.4
0.2
0.0
−0.2
−0.4
F (n=19,357)
M (n=17,259)
All (n=36,616)
0
(0,1]
(1,2]
(2,3]
(3,4]
4+
daily units
two-tailed test against [0,1] group: ⋆p < 0.01, ⋆ ⋆ p < 0.001

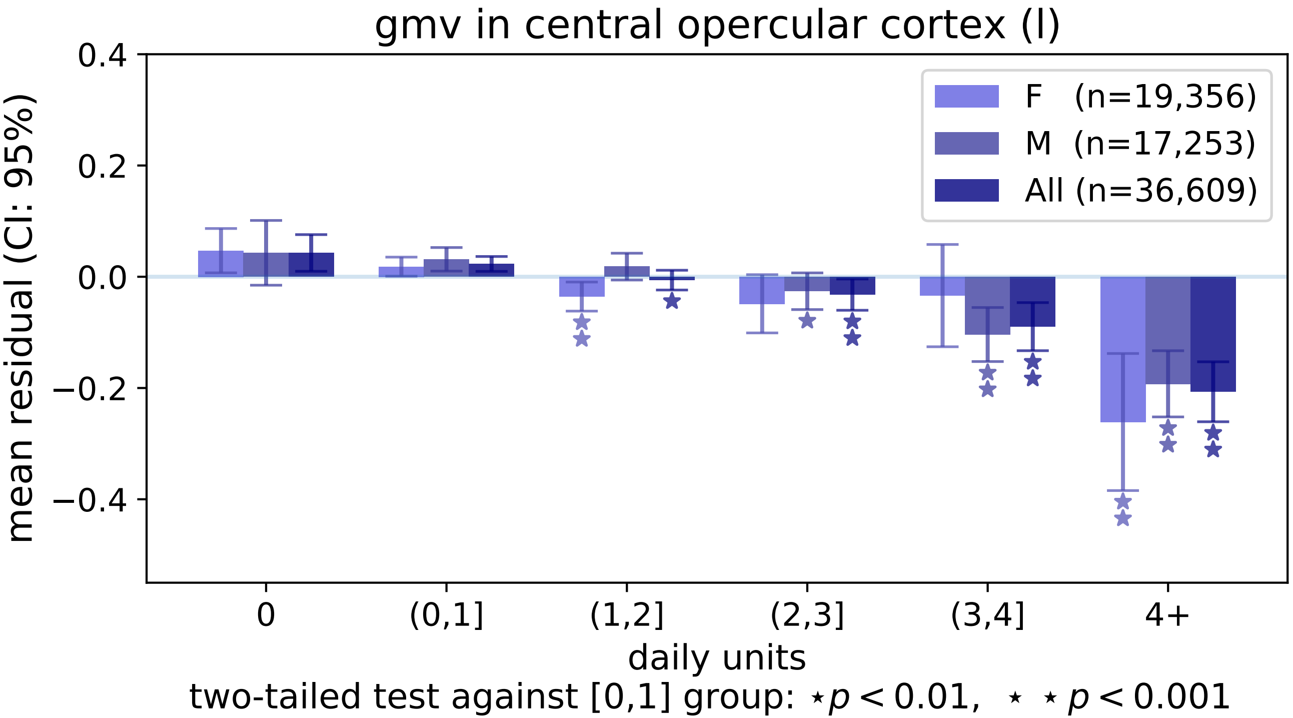
gmv in central opercular cortex (l)
mean residual (CI: 95%)
0.4
0.2
0.0
−0.2
−0.4
F (n=19,356)
M (n=17,253)
All (n=36,609)
0
(0,1]
(1,2]
(2,3]
(3,4]
4+
daily units
two-tailed test against [0,1] group: ⋆p < 0.01, ⋆ ⋆ p < 0.001

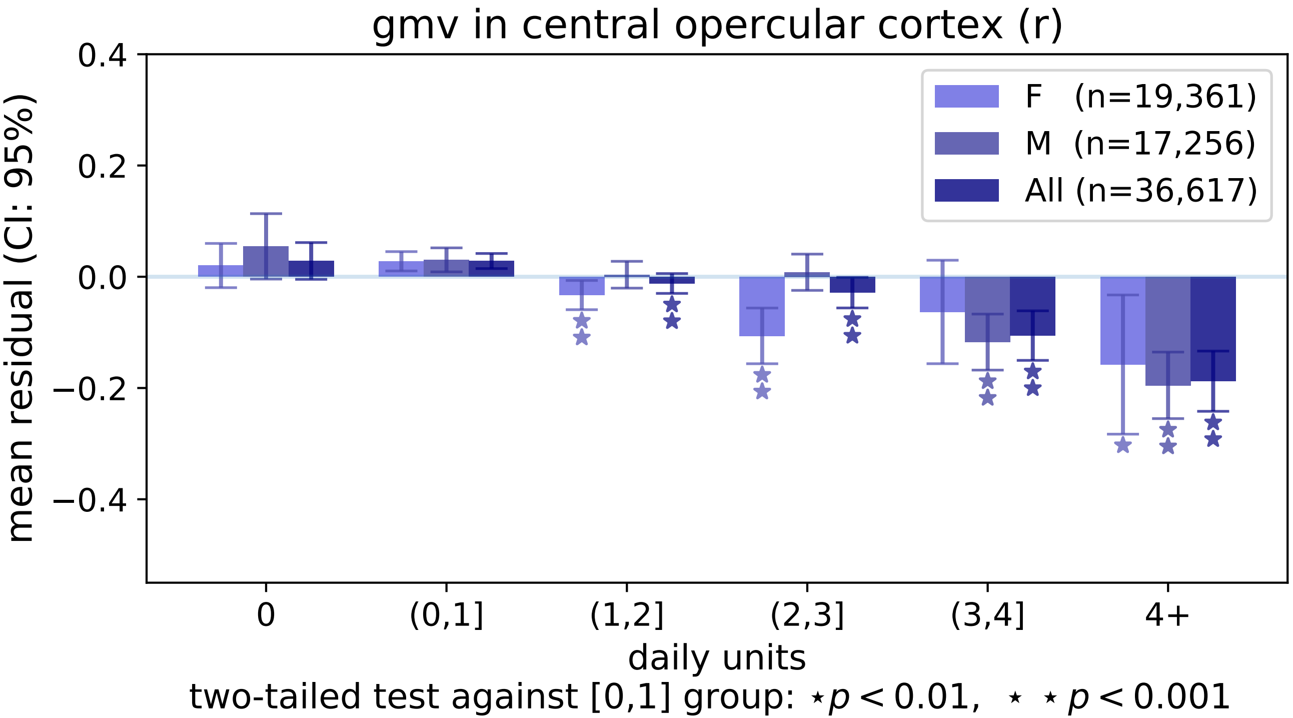
gmv in central opercular cortex (r)
mean residual (CI: 95%)
0.4
0.2
0.0
−0.2
−0.4
F (n=19,361)
M (n=17,256)
All (n=36,617)
0
(0,1]
(1,2]
(2,3]
(3,4]
4+
daily units
two-tailed test against [0,1] group: ⋆ p < 0.01, ⋆ ⋆ p < 0.001

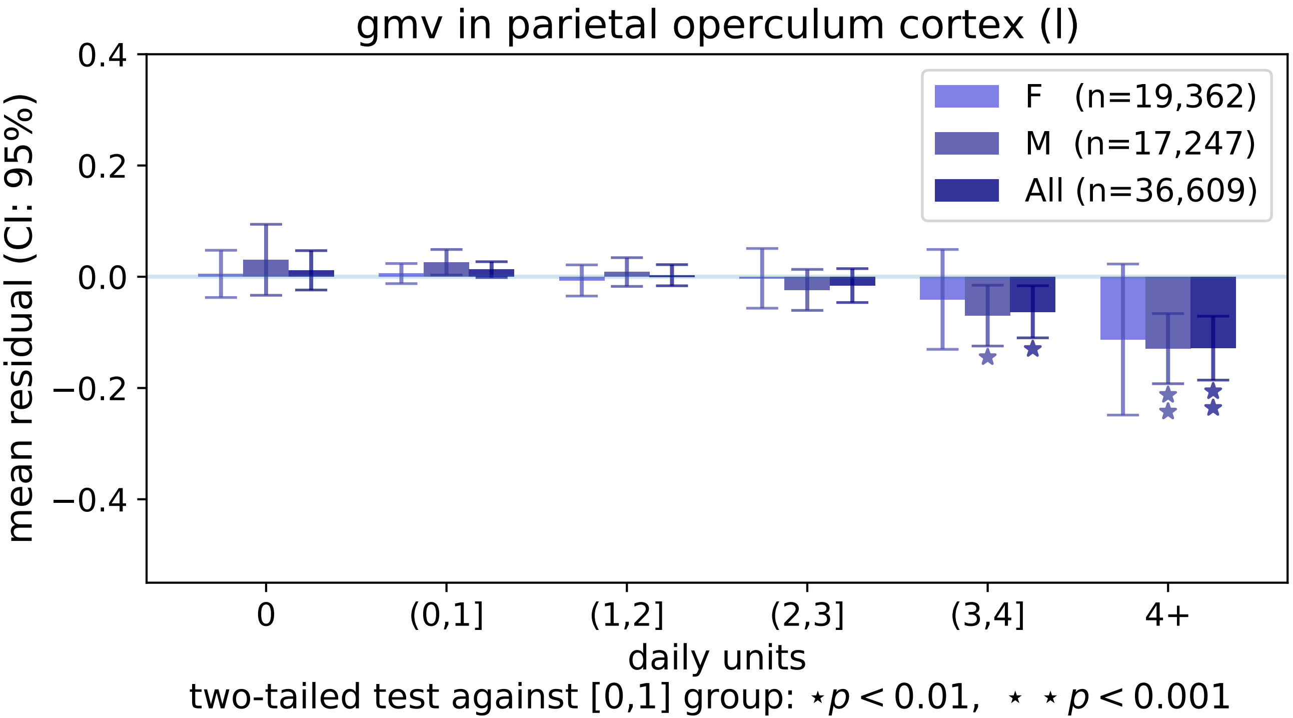
gmv in parietal operculum cortex (l)
mean residual (CI: 95%)
0.4
0.2
0.0
−0.2
−0.4
F (n=19,362)
M (n=17,247)
All (n=36,609)
0
(0,1]
(1,2]
(2,3]
(3,4]
4+
daily units
two-tailed test against [0,1] group: ⋆p < 0.01, ⋆ ⋆ p < 0.001

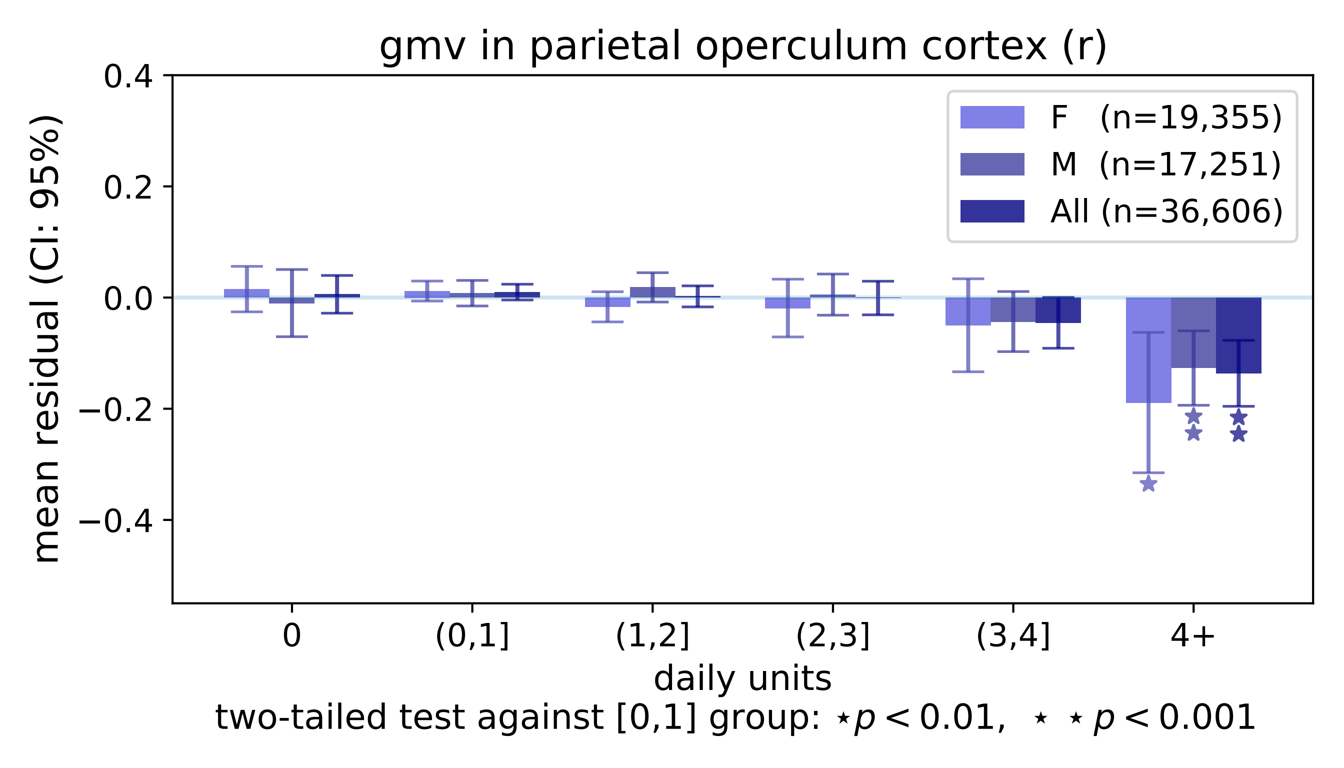
gmv in parietal operculum cortex (r)
mean residual (CI: 95%)
0.4
0.2
0.0
−0.2
−0.4
F (n=19,355)
M (n=17,251)
All (n=36,606)
0
(0,1]
(1,2]
(2,3]
(3,4]
4+
daily units
two-tailed test against [0,1] group: ⋆$p < 0.01$, ⋆ ⋆ $p < 0.001$

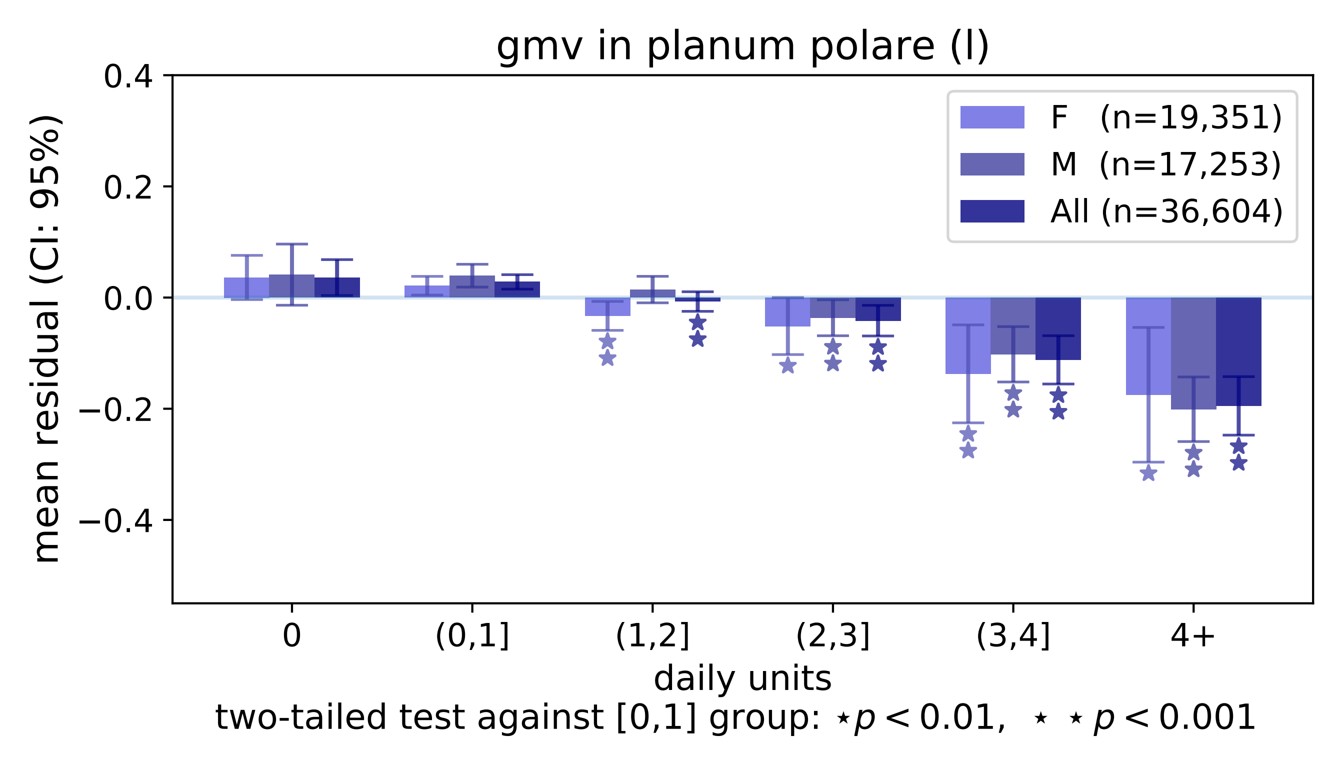
gmv in planum polare (l)
mean residual (CI: 95%)
0.4
0.2
0.0
−0.2
−0.4
F (n=19,351)
M (n=17,253)
All (n=36,604)
0
(0,1]
(1,2]
(2,3]
(3,4]
4+
daily units
two-tailed test against [0,1] group: ⋆ $p < 0.01$, ⋆ ⋆ $p < 0.001$

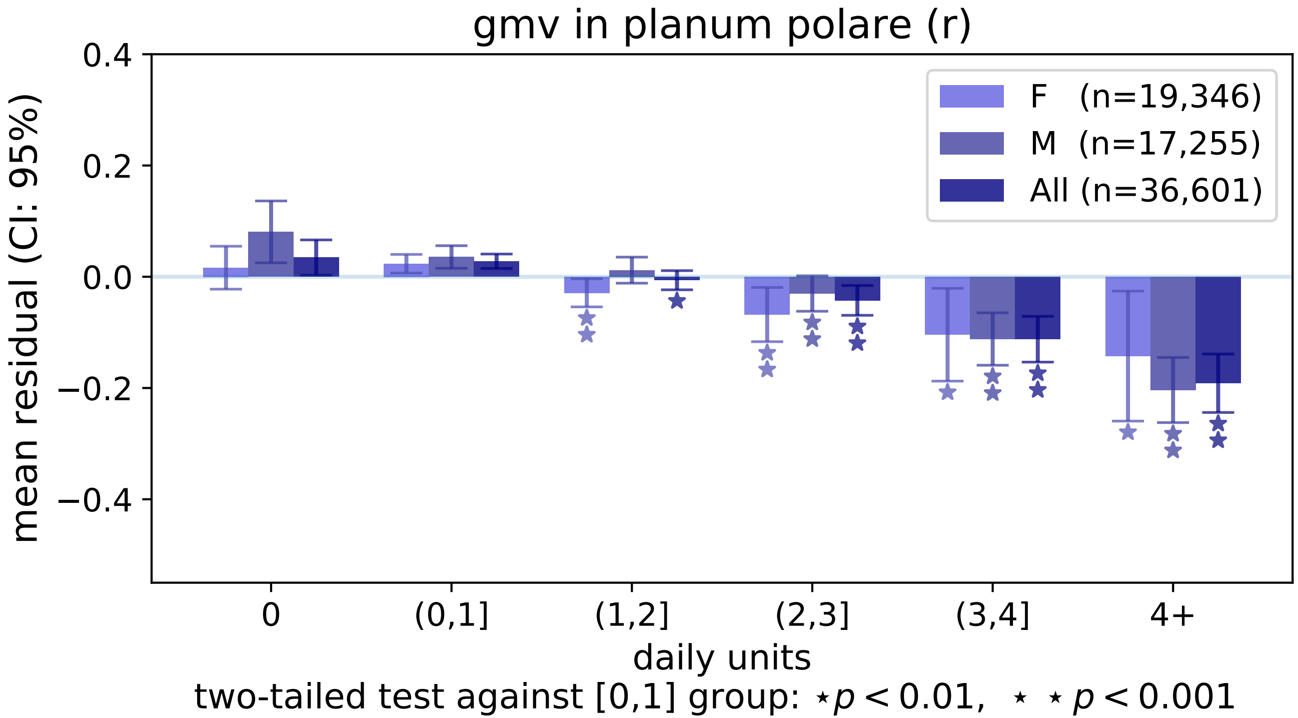

gmv in planum polare (r)
mean residual (CI: 95%)
0.4
0.2
0.0
−0.2
−0.4
F (n=19,346)
M (n=17,255)
All (n=36,601)
0
(0,1]
(1,2]
(2,3]
(3,4]
4+
daily units
two-tailed test against [0,1] group: ⋆p < 0.01, ⋆ ⋆ p < 0.001

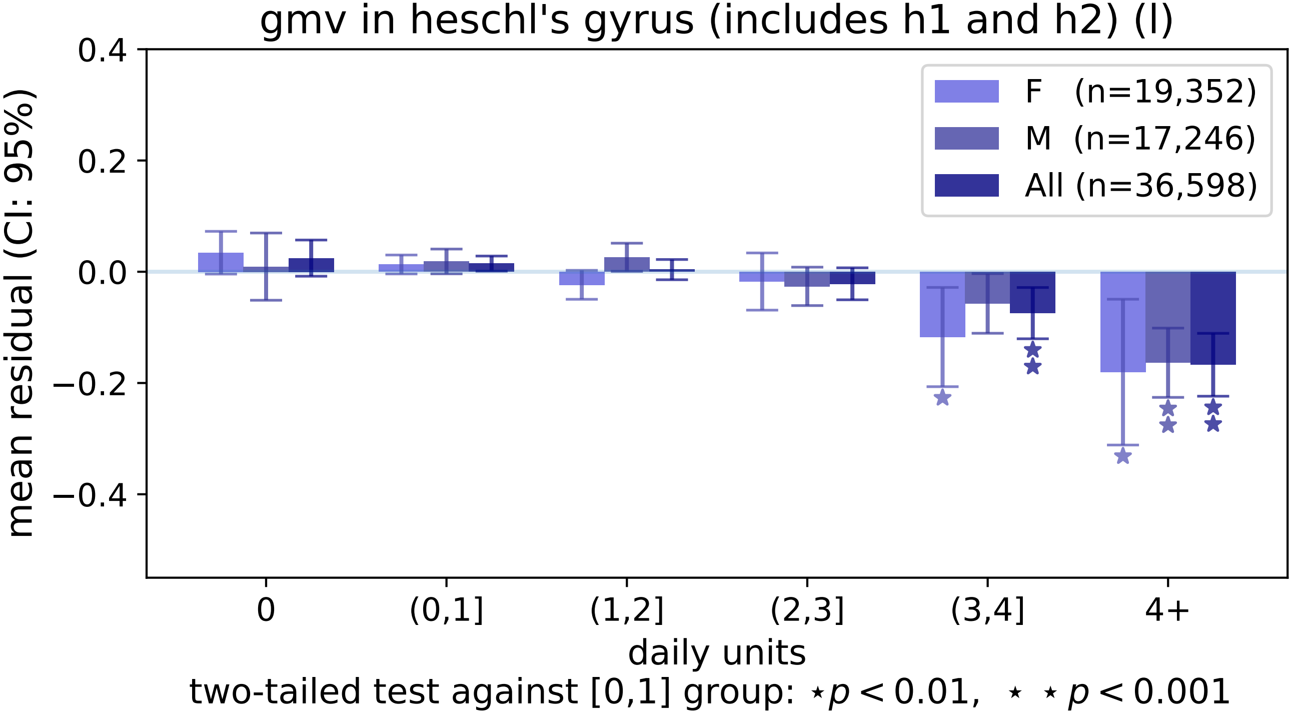
gmv in heschl's gyrus (includes h1 and h2) (l)
mean residual (CI: 95%)
0.4
0.2
0.0
−0.2
−0.4
F (n=19,352)
M (n=17,246)
All (n=36,598)
0
(0,1]
(1,2]
(2,3]
(3,4]
4+
daily units
two-tailed test against [0,1] group: ⋆p < 0.01, ⋆ ⋆ p < 0.001

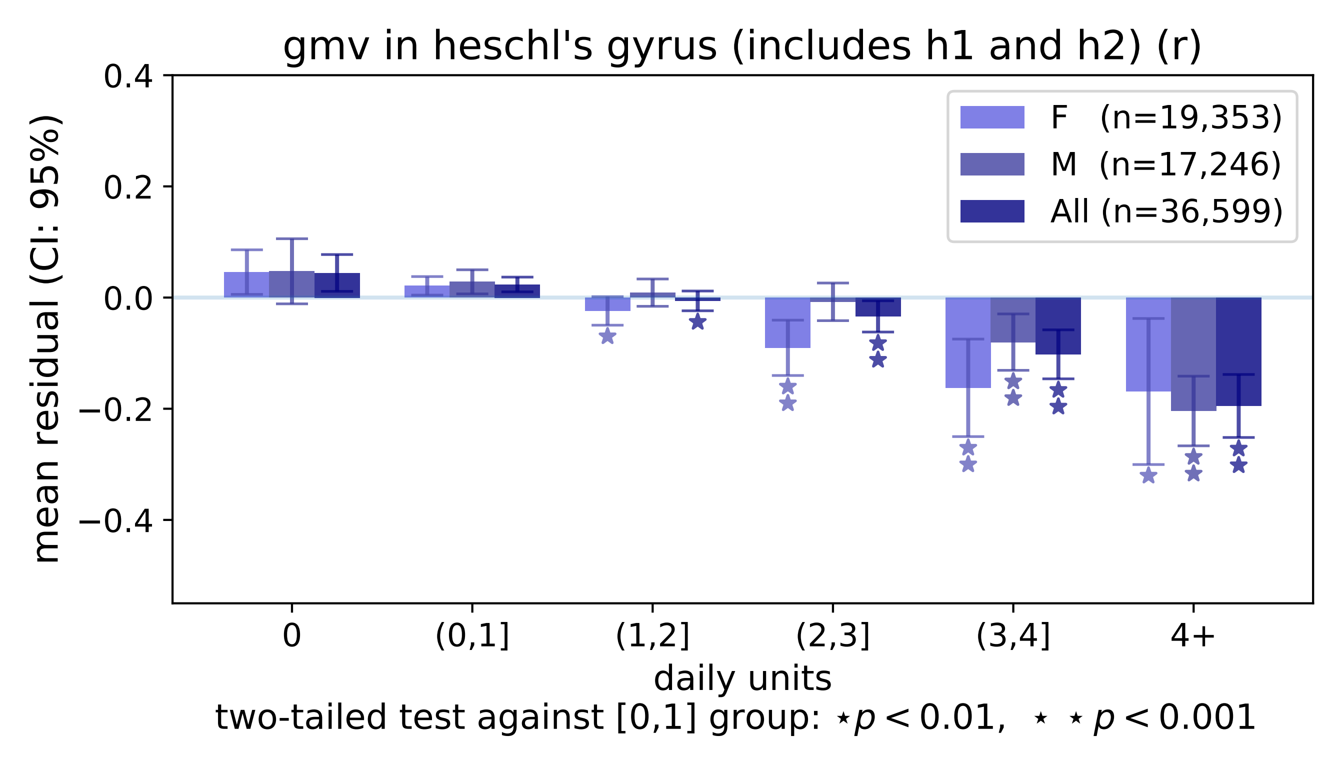
gmv in heschl's gyrus (includes h1 and h2) (r)
mean residual (CI: 95%)
0.4
0.2
0.0
−0.2
−0.4
F (n=19,353)
M (n=17,246)
All (n=36,599)
0
(0,1]
(1,2]
(2,3]
(3,4]
4+
daily units
two-tailed test against [0,1] group: ⋆p < 0.01, ⋆ ⋆ p < 0.001

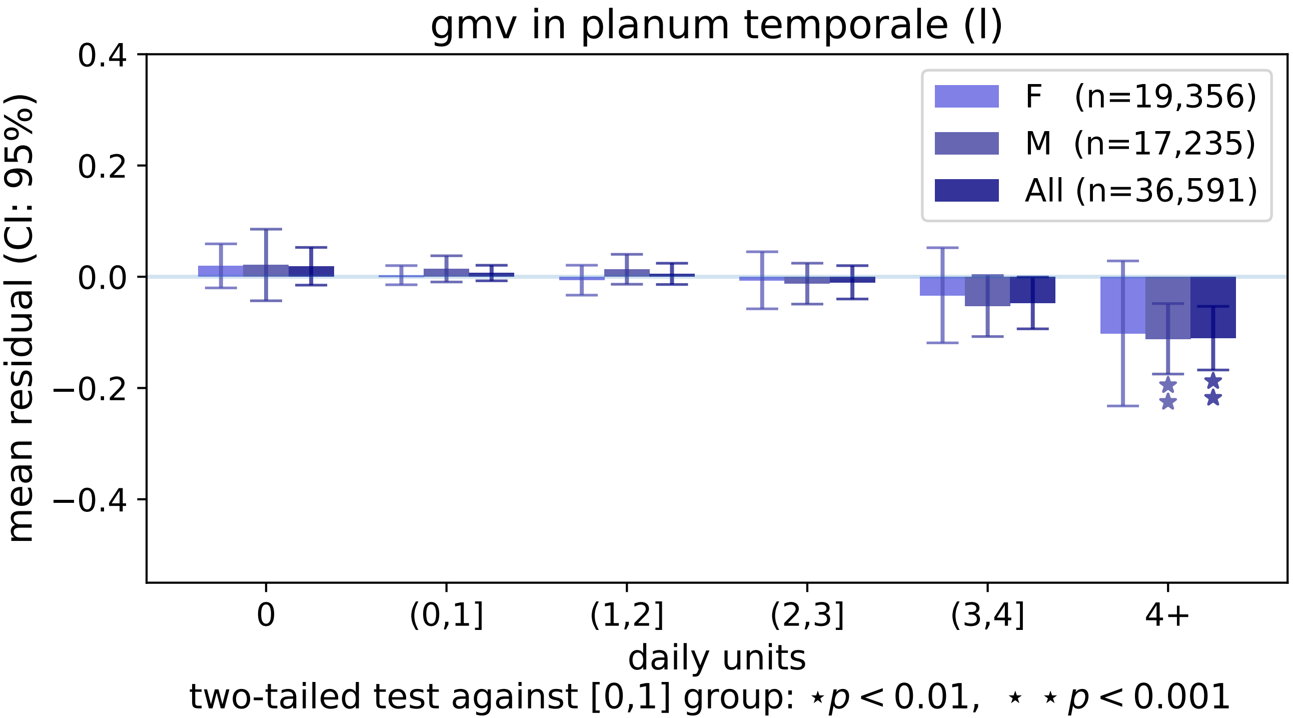
gmv in planum temporale (l)
F (n=19,356)
M (n=17,235)
All (n=36,591)
mean residual (CI: 95%)
0.4
0.2
0.0
−0.2
−0.4
0
(0,1]
(1,2]
(2,3]
(3,4]
4+
daily units
two-tailed test against [0,1] group: ⋆p < 0.01, ⋆ ⋆ p < 0.001

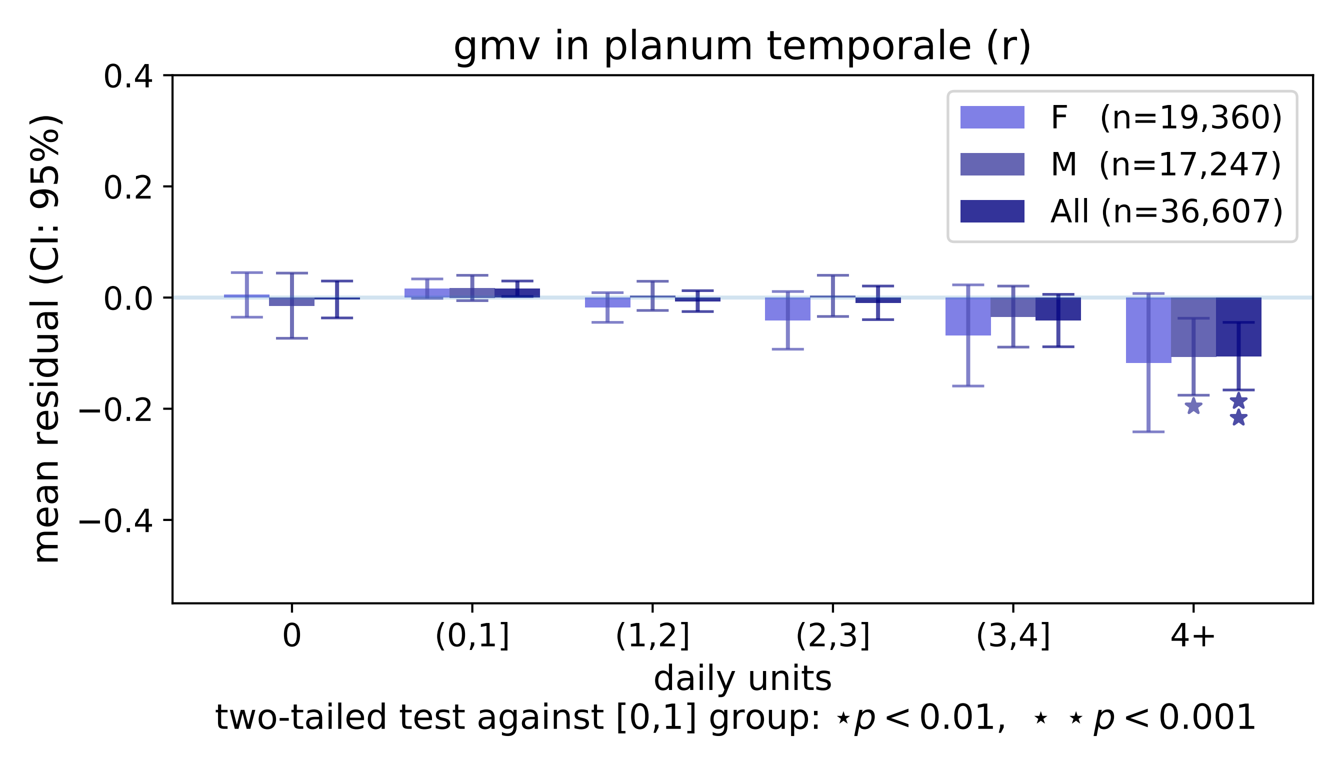
gmv in planum temporale (r)
0.4
0.2
0.0
−0.2
−0.4
mean residual (CI: 95%)
F (n=19,360)
M (n=17,247)
All (n=36,607)
0
(0,1]
(1,2]
(2,3]
(3,4]
4+
daily units
two-tailed test against [0,1] group: ⋆p < 0.01, ⋆ ⋆ p < 0.001

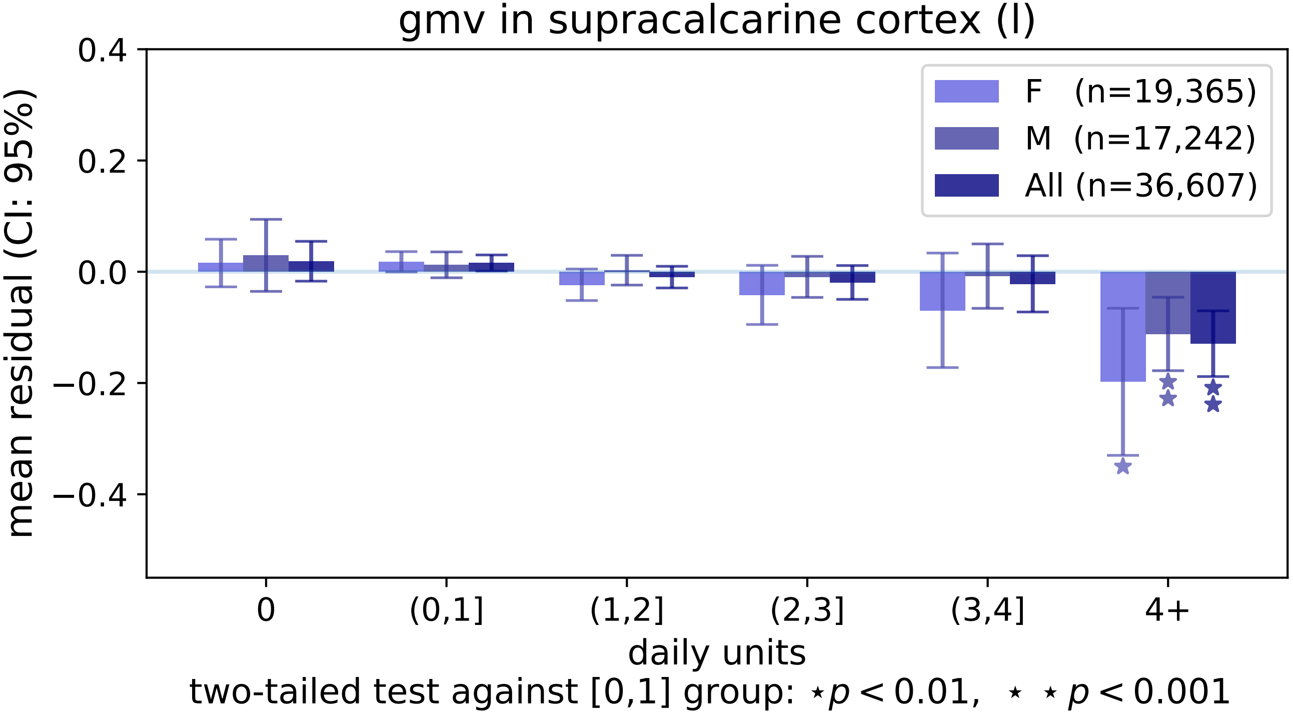

gmv in supracalcarine cortex (l)
mean residual (CI: 95%)
0.4
0.2
0.0
−0.2
−0.4
F (n=19,365)
M (n=17,242)
All (n=36,607)
0
(0,1]
(1,2]
(2,3]
(3,4]
4+
daily units
two-tailed test against [0,1] group: ⋆ p < 0.01, ⋆ ⋆ p < 0.001

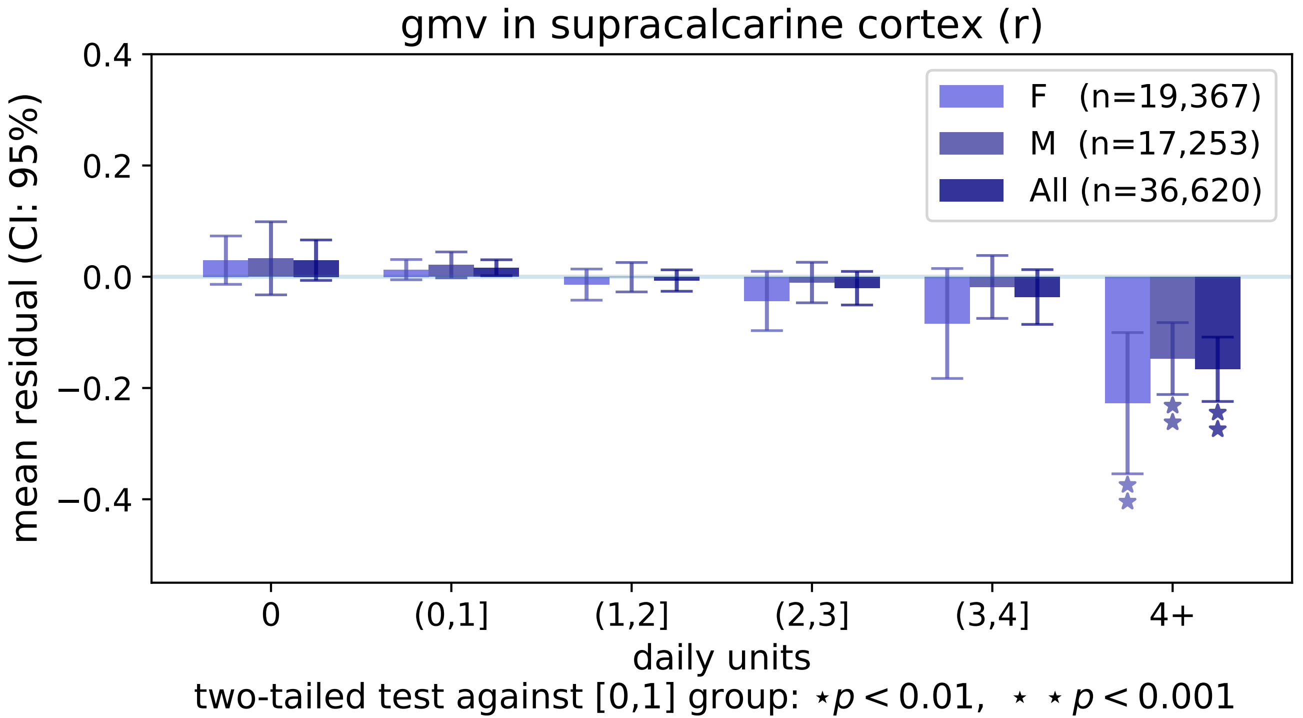
gmv in supracalcarine cortex (r)
mean residual (CI: 95%)
0.4
0.2
0.0
−0.2
−0.4
F (n=19,367)
M (n=17,253)
All (n=36,620)
0
(0,1]
(1,2]
(2,3]
(3,4]
4+
daily units
two-tailed test against [0,1] group: ⋆ p < 0.01, ⋆ ⋆ p < 0.001

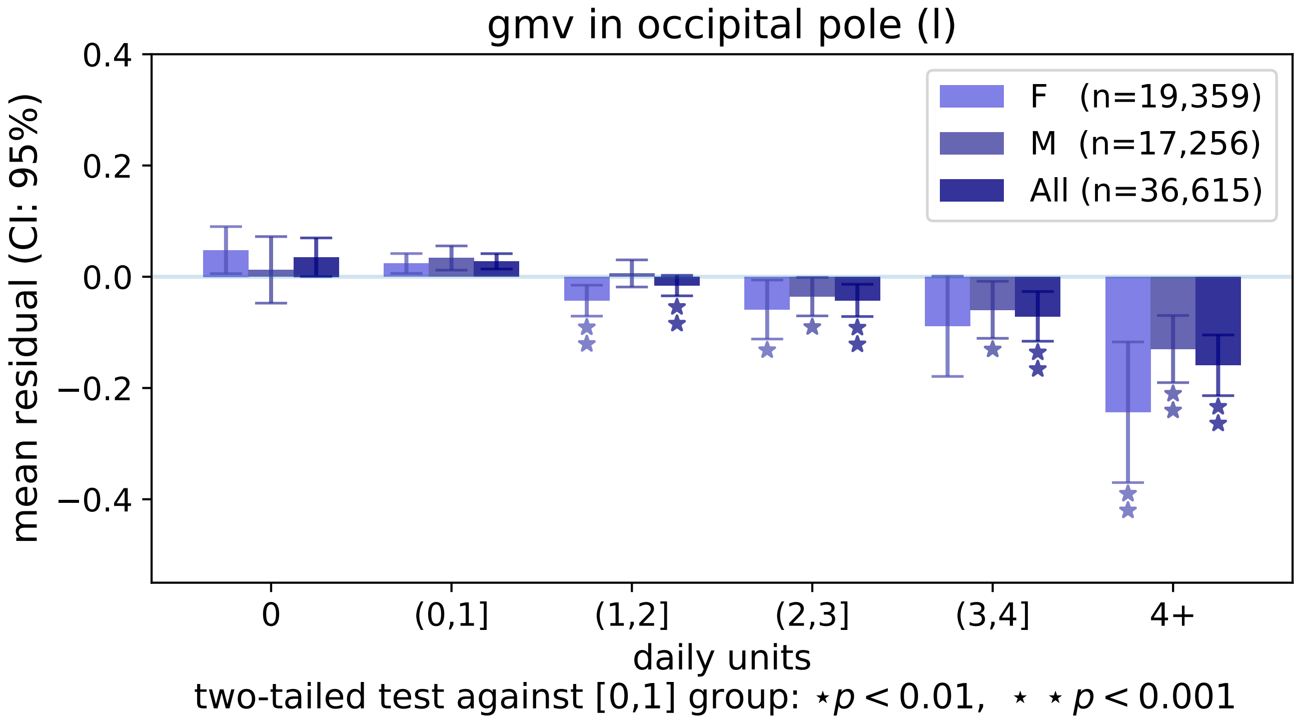
gmv in occipital pole (l)
0.4
0.2
0.0
−0.2
−0.4
mean residual (CI: 95%)
F (n=19,359)
M (n=17,256)
All (n=36,615)
0
(0,1]
(1,2]
(2,3]
(3,4]
4+
daily units
two-tailed test against [0,1] group: ⋆ p < 0.01, ⋆ ⋆ p < 0.001

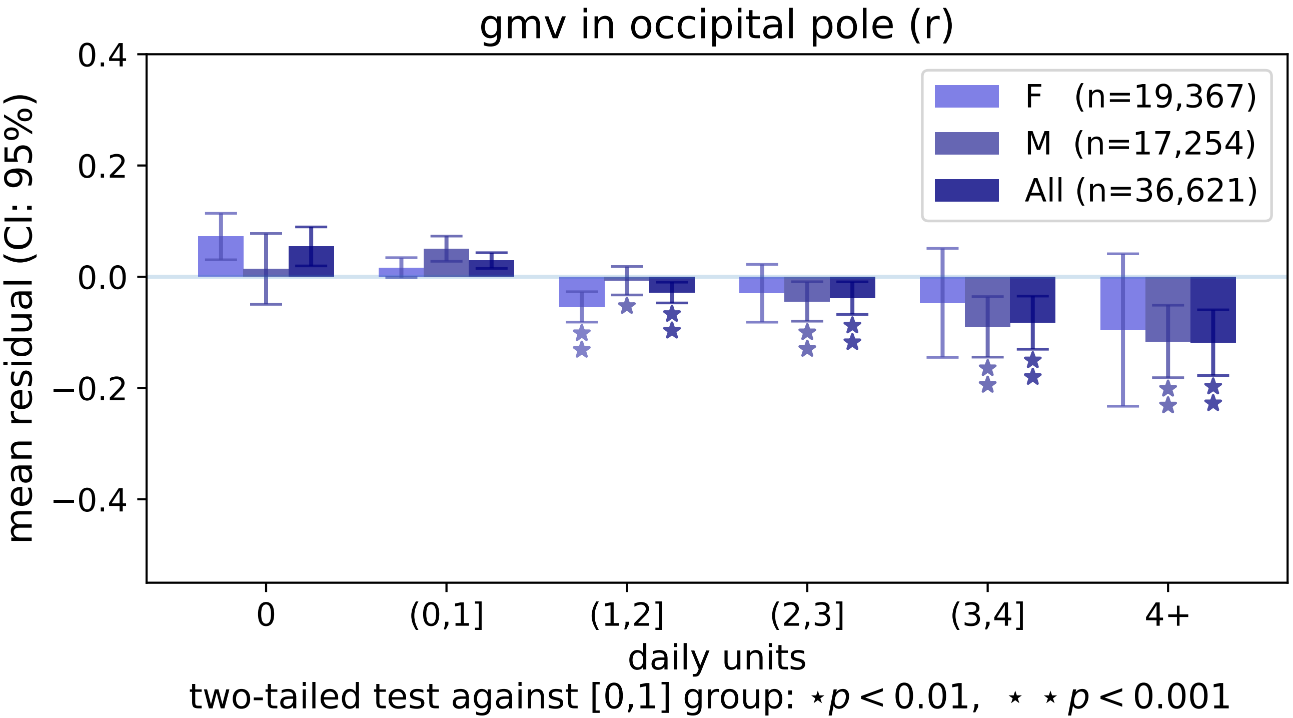
gmv in occipital pole (r)
0.4
0.2
0.0
−0.2
−0.4
mean residual (CI: 95%)
F (n=19,367)
M (n=17,254)
All (n=36,621)
0
(0,1]
(1,2]
(2,3]
(3,4]
4+
daily units
two-tailed test against [0,1] group: ⋆p < 0.01, ⋆ ⋆ p < 0.001

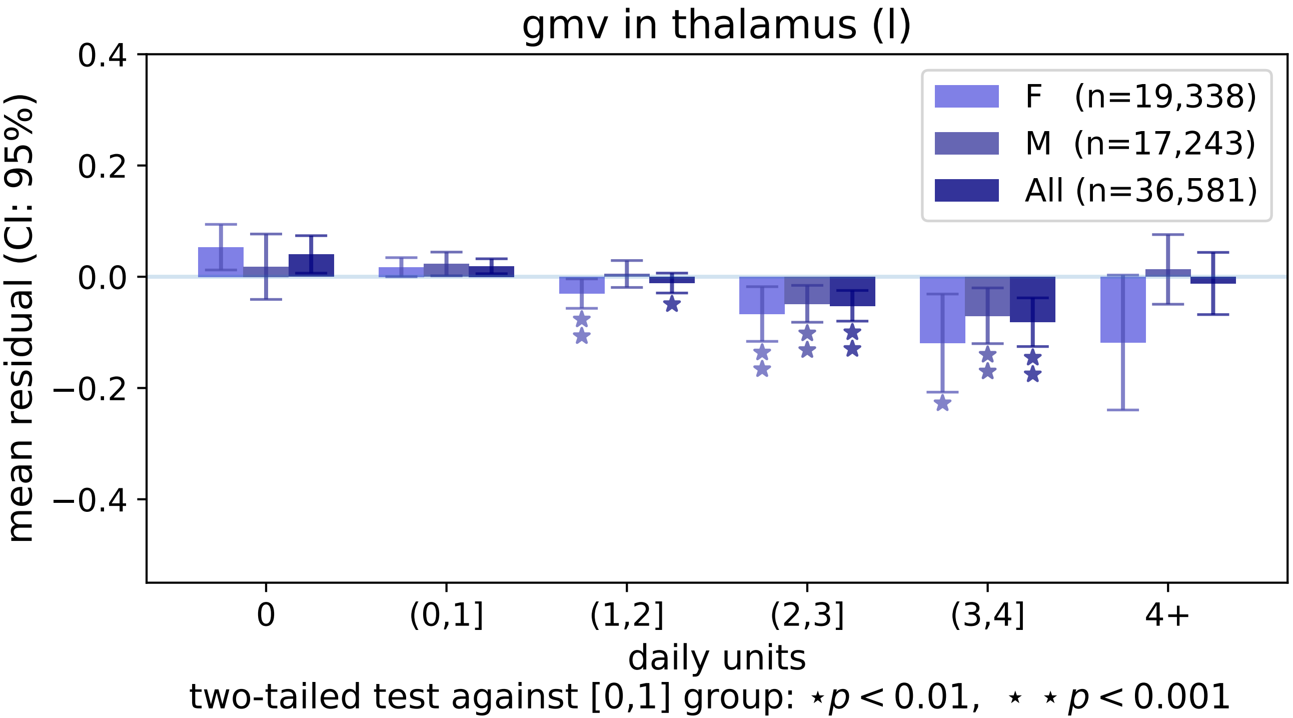

gmv in thalamus (l)
0.4
0.2
0.0
−0.2
−0.4
mean residual (CI: 95%)
F (n=19,338)
M (n=17,243)
All (n=36,581)
0
(0,1]
(1,2]
(2,3]
(3,4]
4+
daily units
two-tailed test against [0,1] group: ⋆p < 0.01, ⋆ ⋆ p < 0.001

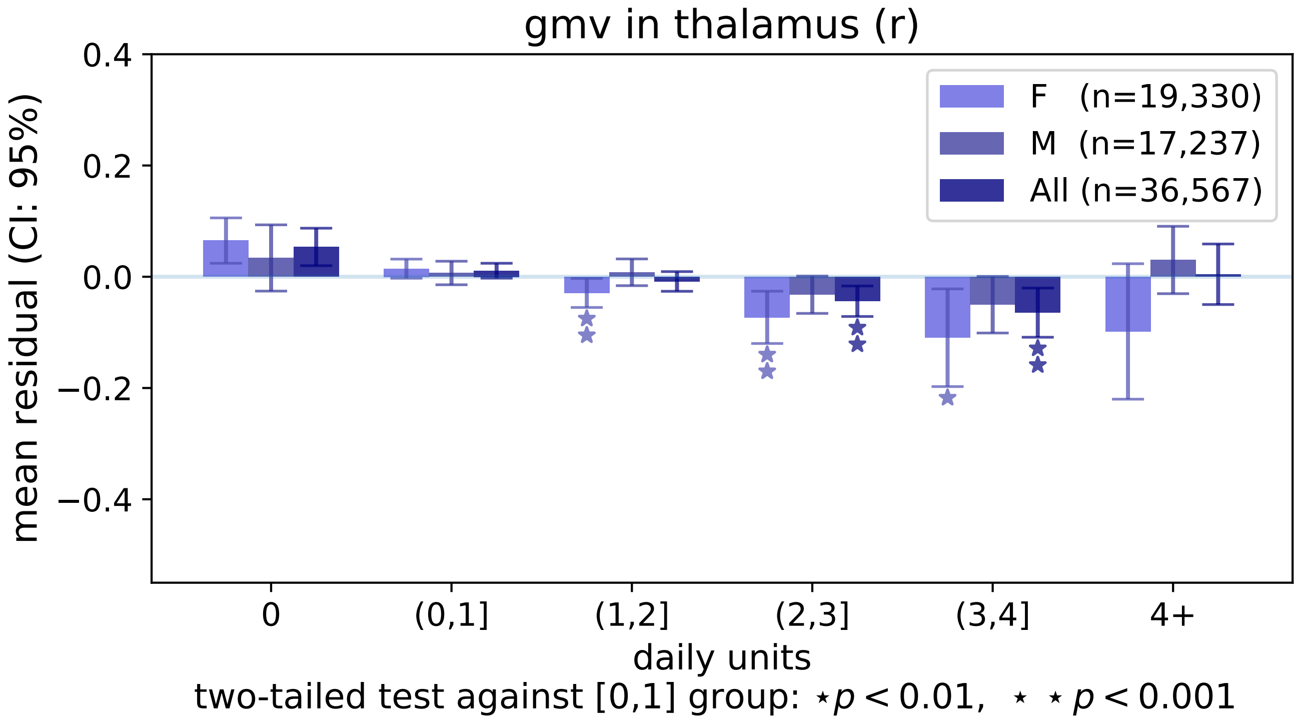
gmv in thalamus (r)
mean residual (CI: 95%)
0.4
0.2
0.0
−0.2
−0.4
F (n=19,330)
M (n=17,237)
All (n=36,567)
0
(0,1]
(1,2]
(2,3]
(3,4]
4+
daily units
two-tailed test against [0,1] group: ⋆p < 0.01, ⋆ ⋆ p < 0.001

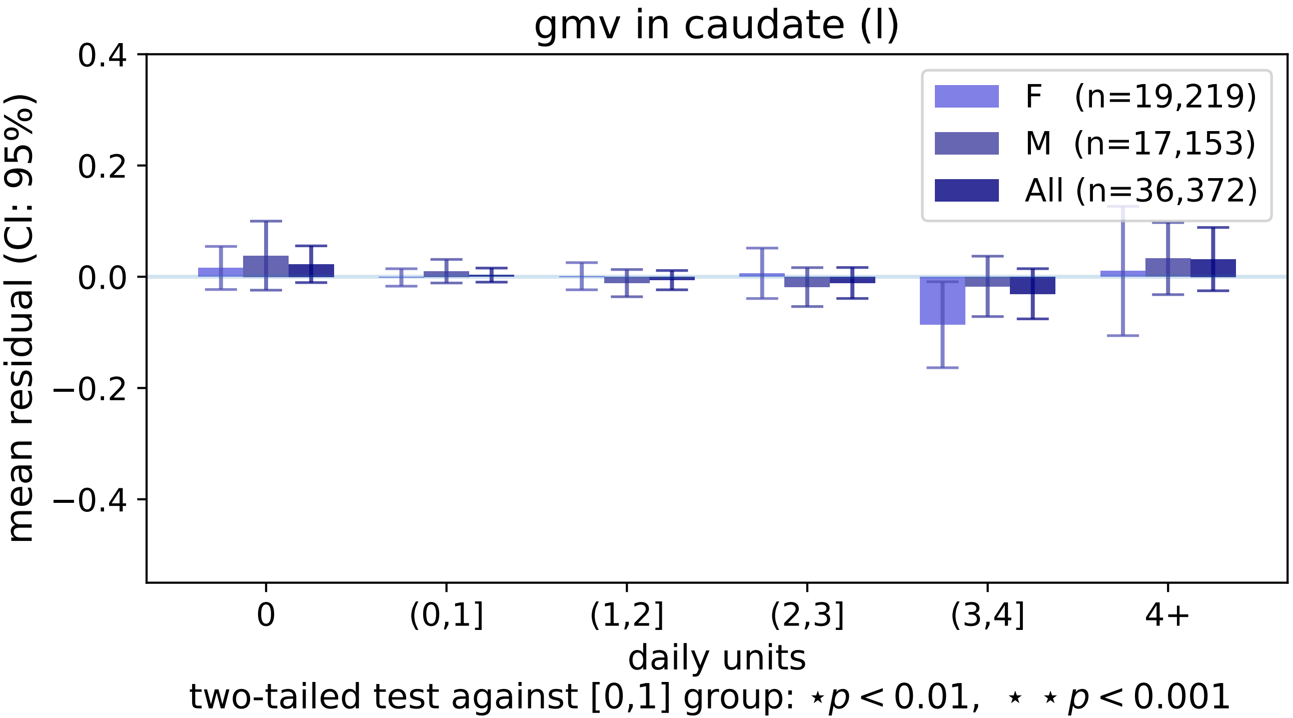
gmv in caudate (l)
mean residual (CI: 95%)
0.4
0.2
0.0
−0.2
−0.4
F (n=19,219)
M (n=17,153)
All (n=36,372)
0
(0,1]
(1,2]
(2,3]
(3,4]
4+
daily units
two-tailed test against [0,1] group: ⋆p < 0.01, ⋆ ⋆ p < 0.001

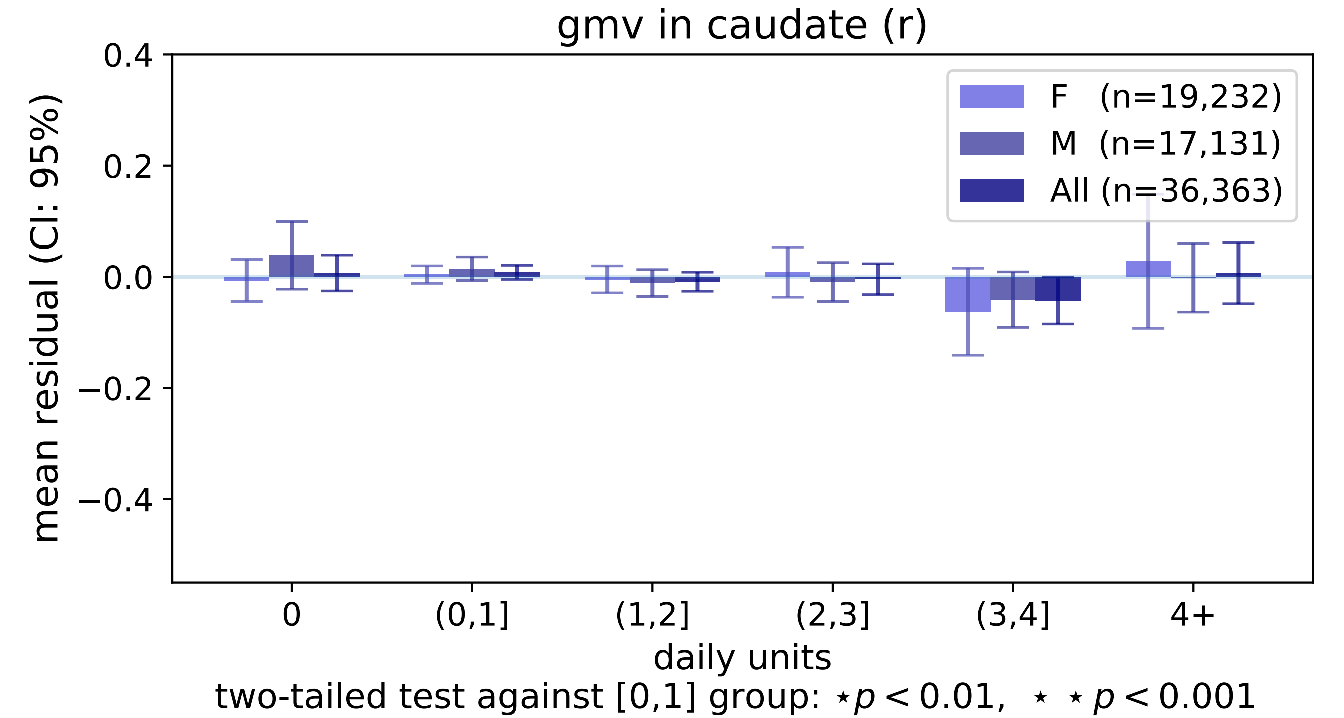

gmv in caudate (r)
mean residual (CI: 95%)
0.4
0.2
0.0
−0.2
−0.4
F (n=19,232)
M (n=17,131)
All (n=36,363)
0
(0,1]
(1,2]
(2,3]
(3,4]
4+
daily units
two-tailed test against [0,1] group: ⋆$p < 0.01$, ⋆ ⋆ $p < 0.001$

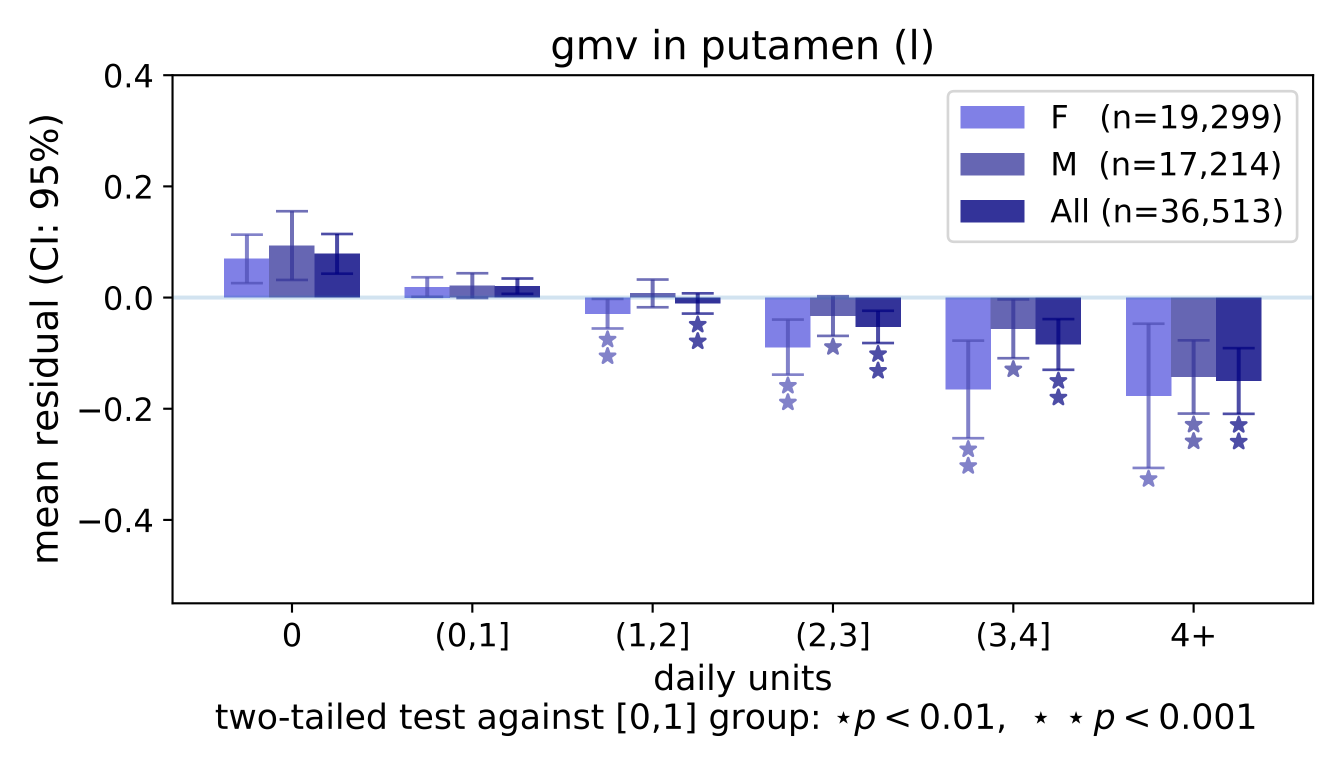
gmv in putamen (l)
0.4
0.2
0.0
−0.2
−0.4
mean residual (CI: 95%)
F (n=19,299)
M (n=17,214)
All (n=36,513)
0
(0,1]
(1,2]
(2,3]
(3,4]
4+
daily units
two-tailed test against [0,1] group: ⋆$p < 0.01$, ⋆ ⋆ $p < 0.001$

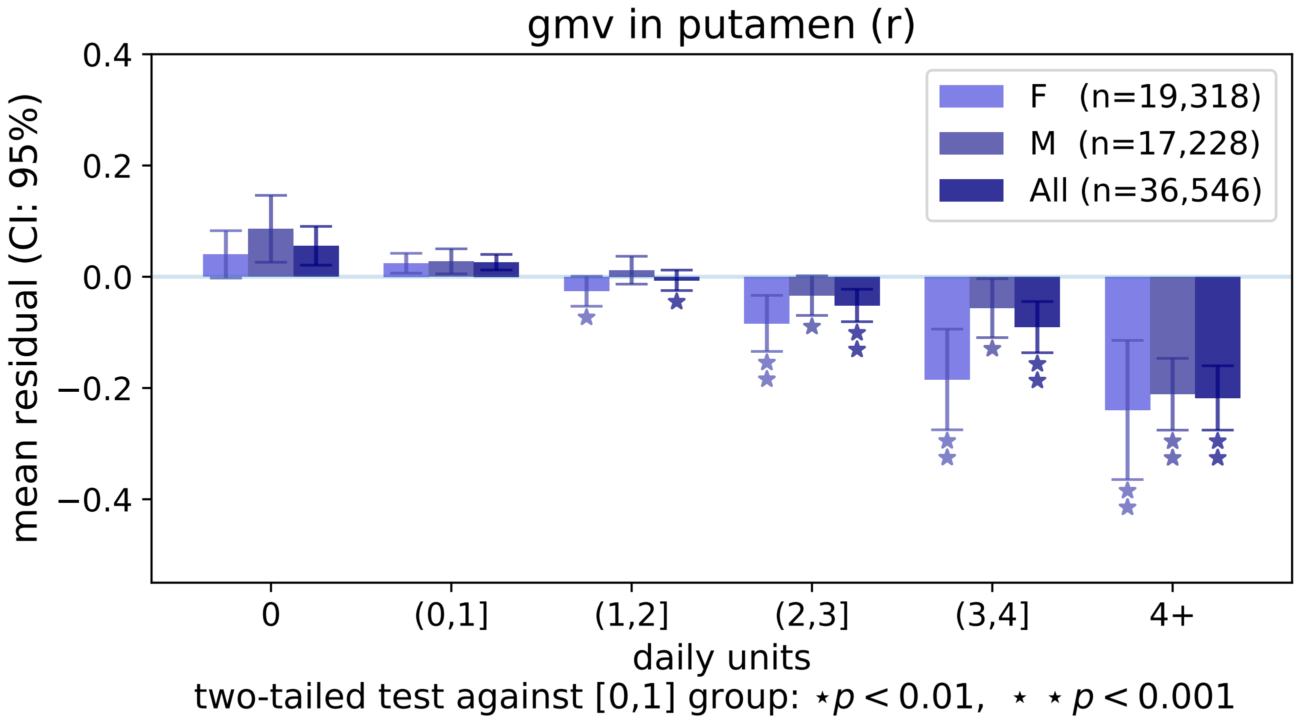
gmv in putamen (r)
mean residual (CI: 95%)
0.4
0.2
0.0
−0.2
−0.4
F (n=19,318)
M (n=17,228)
All (n=36,546)
0
(0,1]
(1,2]
(2,3]
(3,4]
4+
daily units
two-tailed test against [0,1] group: ⋆p < 0.01, ⋆ ⋆ p < 0.001

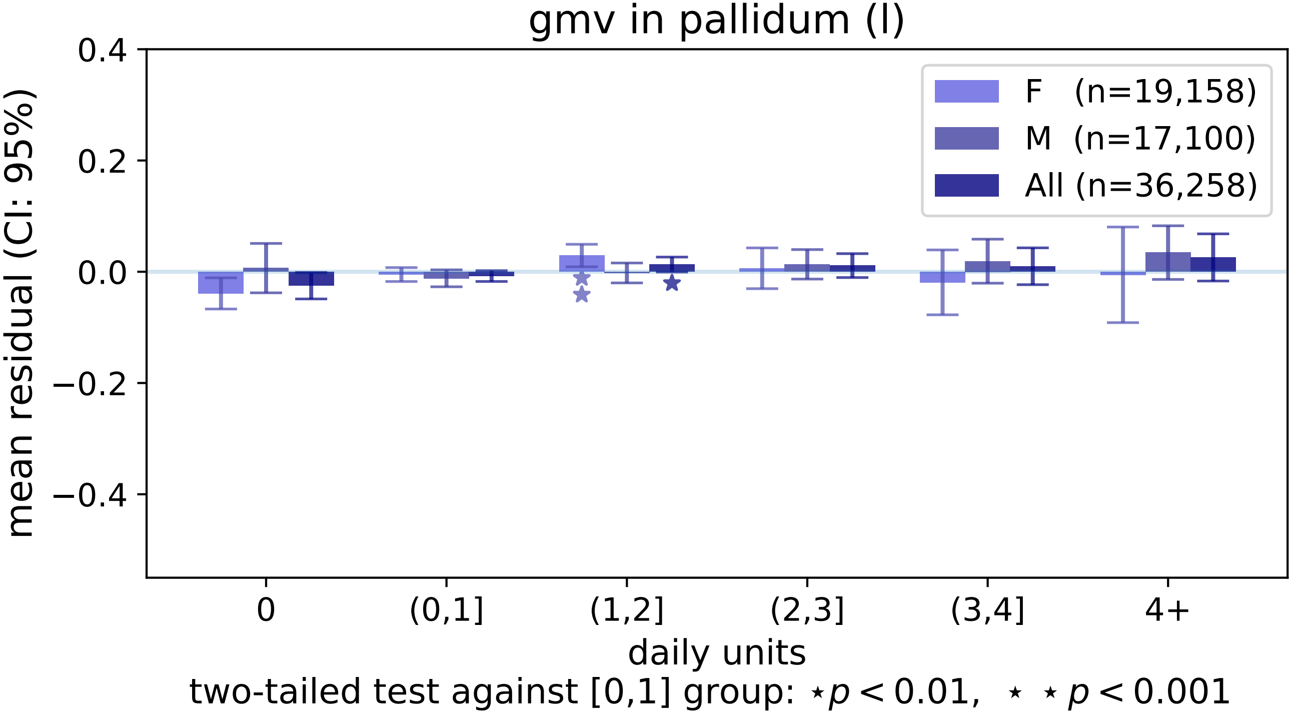

gmv in pallidum (l)
0.4
0.2
0.0
−0.2
−0.4
mean residual (CI: 95%)
F (n=19,158)
M (n=17,100)
All (n=36,258)
0
(0,1]
(1,2]
(2,3]
(3,4]
4+
daily units
two-tailed test against [0,1] group: ⋆p < 0.01, ⋆ ⋆ p < 0.001

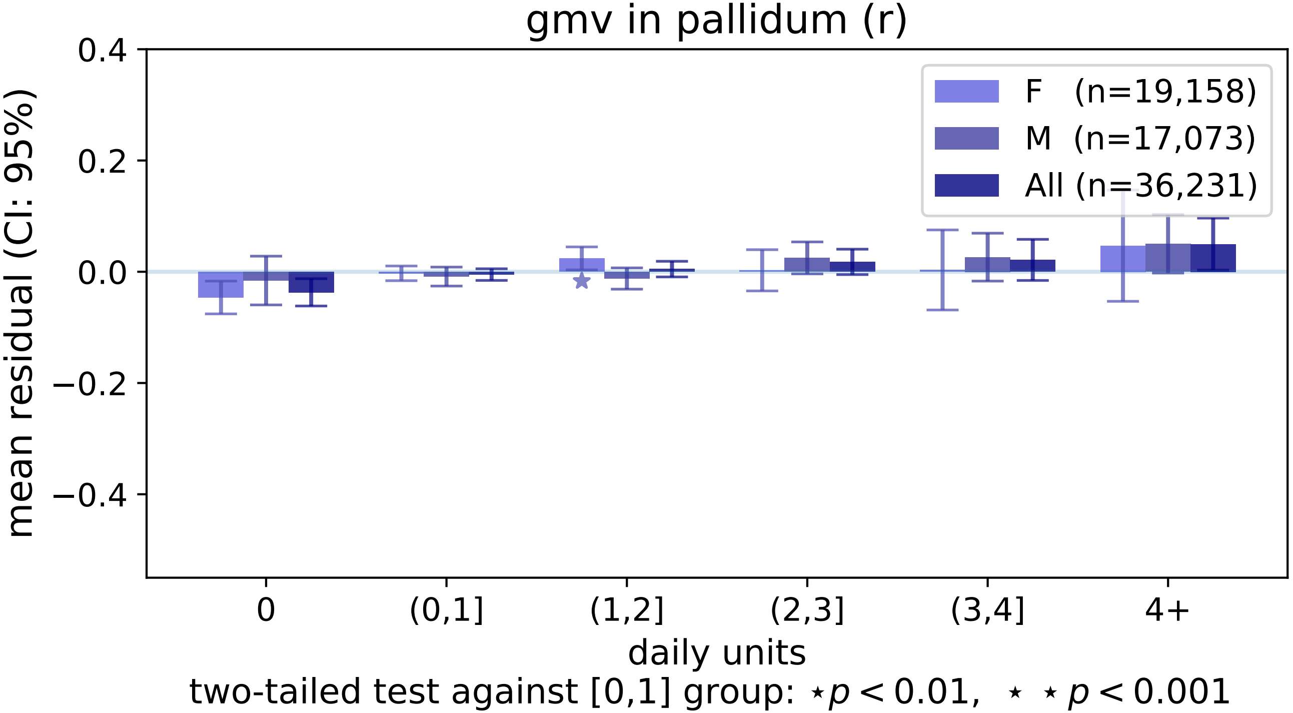
gmv in pallidum (r)
mean residual (CI: 95%)
0.4
0.2
0.0
−0.2
−0.4
F (n=19,158)
M (n=17,073)
All (n=36,231)
0
(0,1]
(1,2]
(2,3]
(3,4]
4+
daily units
two-tailed test against [0,1] group: ⋆p < 0.01, ⋆ ⋆ p < 0.001

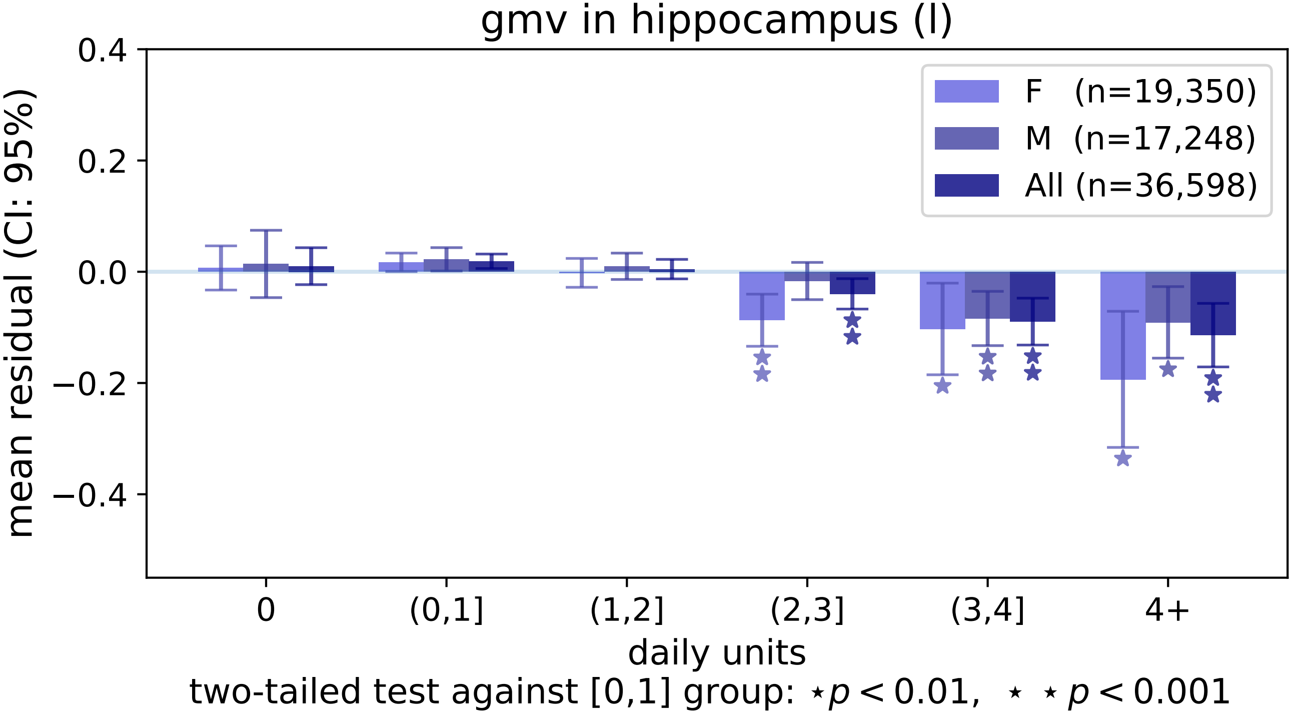
gmv in hippocampus (l)
mean residual (CI: 95%)
0.4
0.2
0.0
−0.2
−0.4
F (n=19,350)
M (n=17,248)
All (n=36,598)
0
(0,1]
(1,2]
(2,3]
(3,4]
4+
daily units
two-tailed test against [0,1] group: ⋆p < 0.01, ⋆ ⋆ p < 0.001

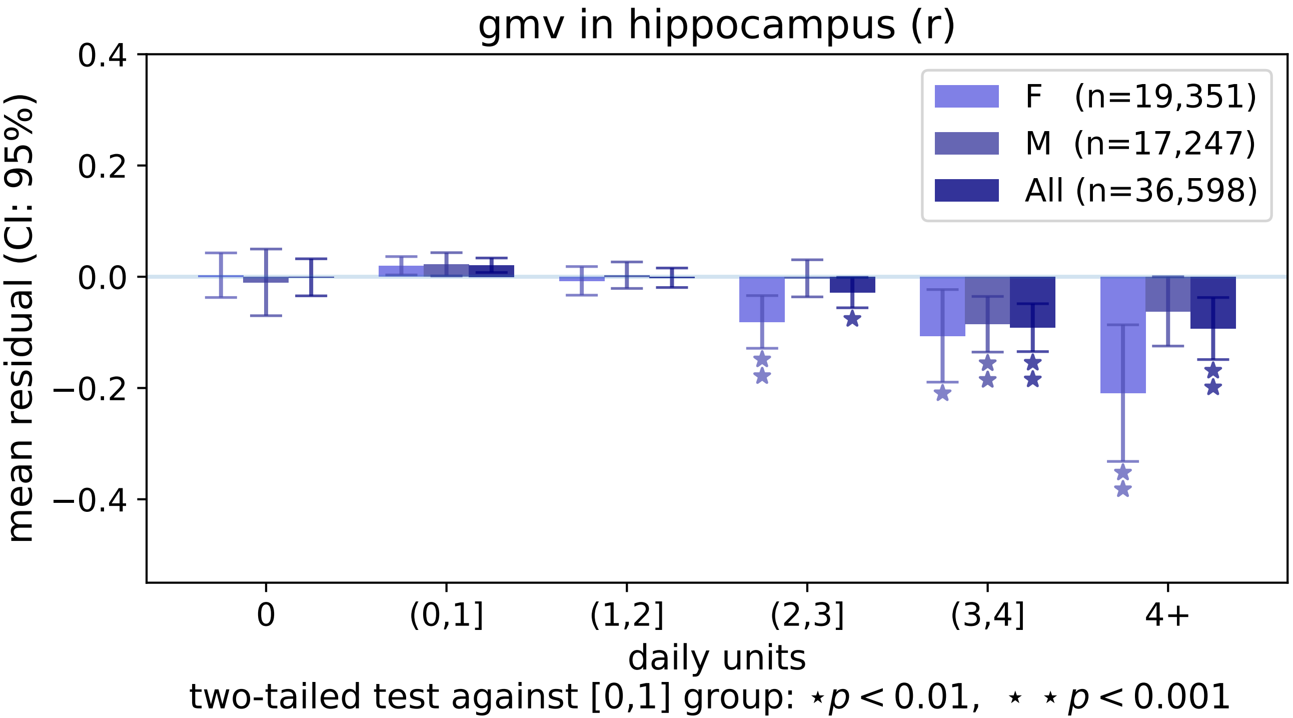
gmv in hippocampus (r)
F (n=19,351)
M (n=17,247)
All (n=36,598)
mean residual (CI: 95%)
0.4
0.2
0.0
−0.2
−0.4
0
(0,1]
(1,2]
(2,3]
(3,4]
4+
daily units
two-tailed test against [0,1] group: ⋆p < 0.01, ⋆ ⋆ p < 0.001

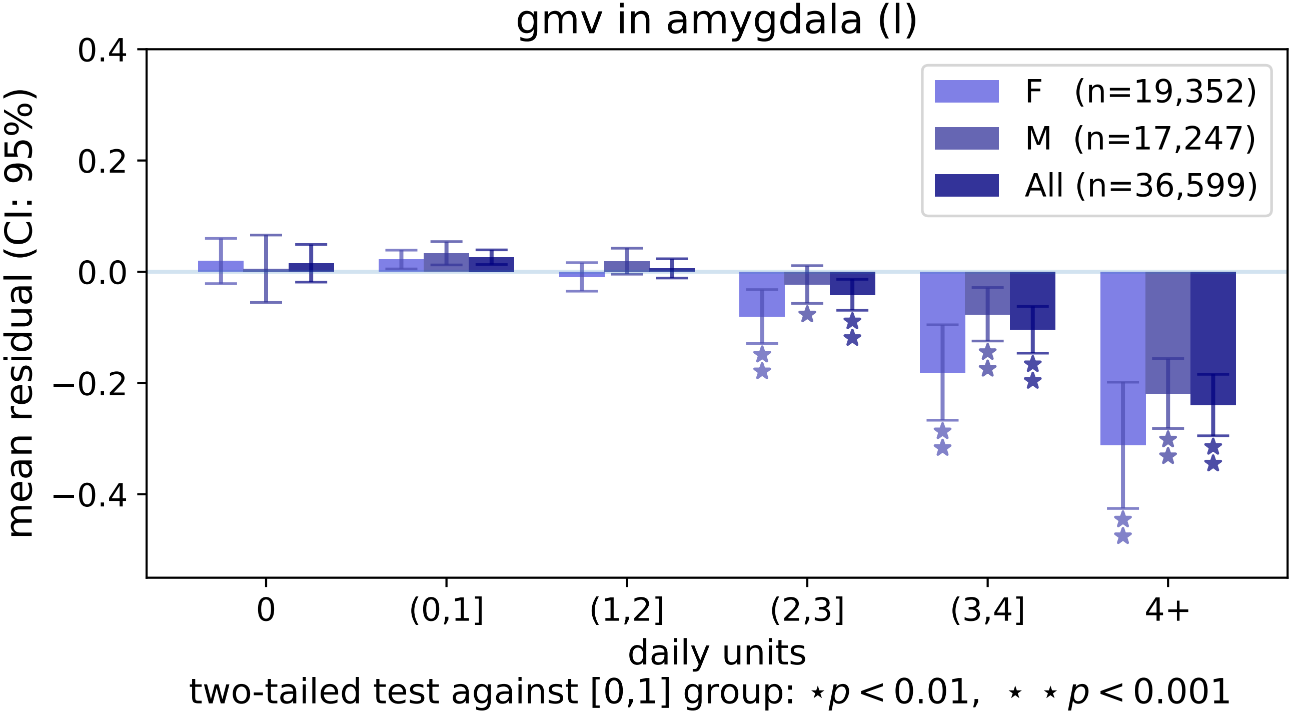
gmv in amygdala (l)
mean residual (CI: 95%)
0.4
0.2
0.0
−0.2
−0.4
F (n=19,352)
M (n=17,247)
All (n=36,599)
0
(0,1]
(1,2]
(2,3]
(3,4]
4+
daily units
two-tailed test against [0,1] group: ⋆ p < 0.01, ⋆ ⋆ p < 0.001

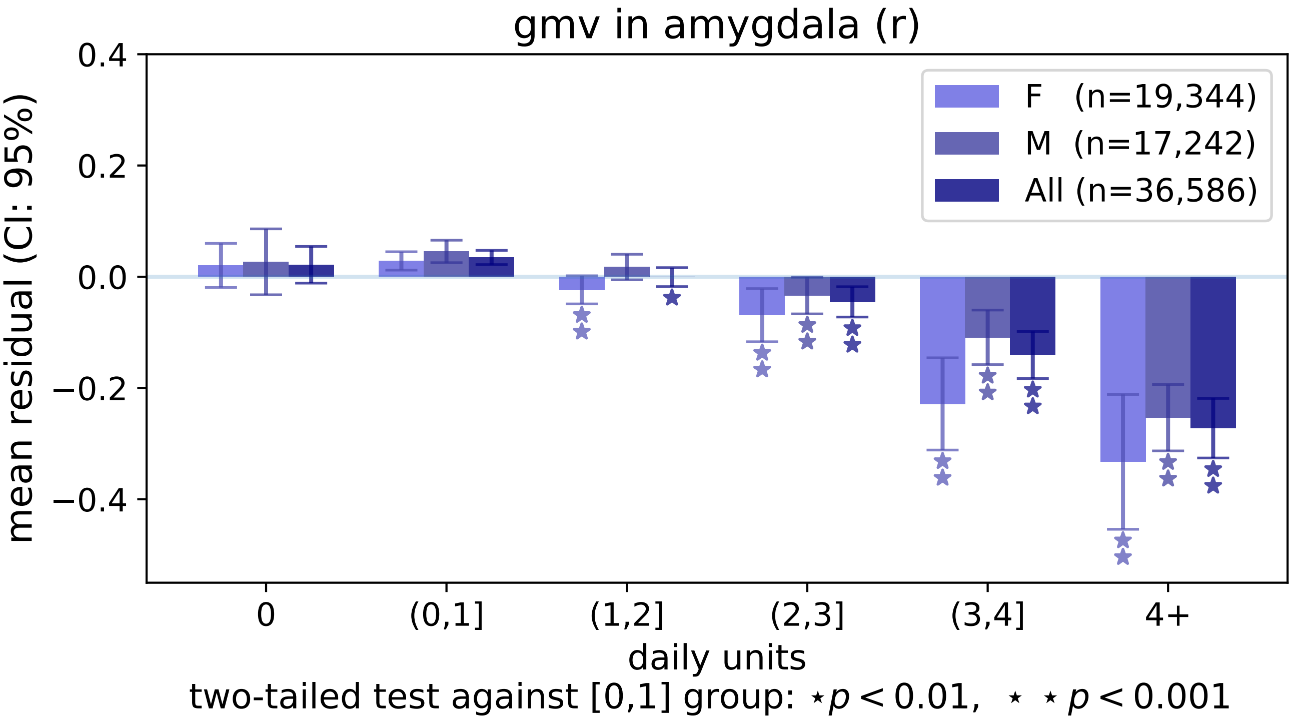

gmv in amygdala (r)
0.4
0.2
0.0
−0.2
−0.4
mean residual (CI: 95%)
F (n=19,344)
M (n=17,242)
All (n=36,586)
0
(0,1]
(1,2]
(2,3]
(3,4]
4+
daily units
two-tailed test against [0,1] group: ⋆p < 0.01, ⋆ ⋆ p < 0.001

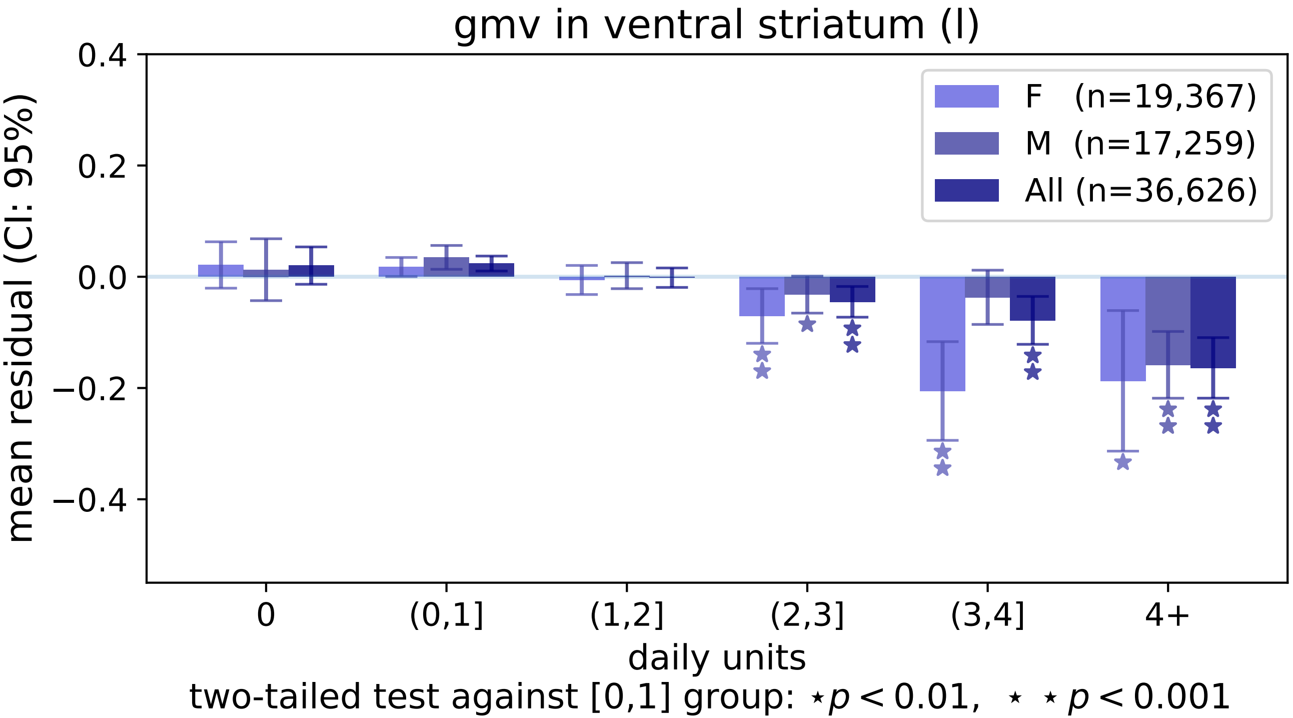
gmv in ventral striatum (l)
mean residual (CI: 95%)
0.4
0.2
0.0
−0.2
−0.4
F (n=19,367)
M (n=17,259)
All (n=36,626)
0
(0,1]
(1,2]
(2,3]
(3,4]
4+
daily units
two-tailed test against [0,1] group: ⋆ $p < 0.01$, ⋆ ⋆ $p < 0.001$

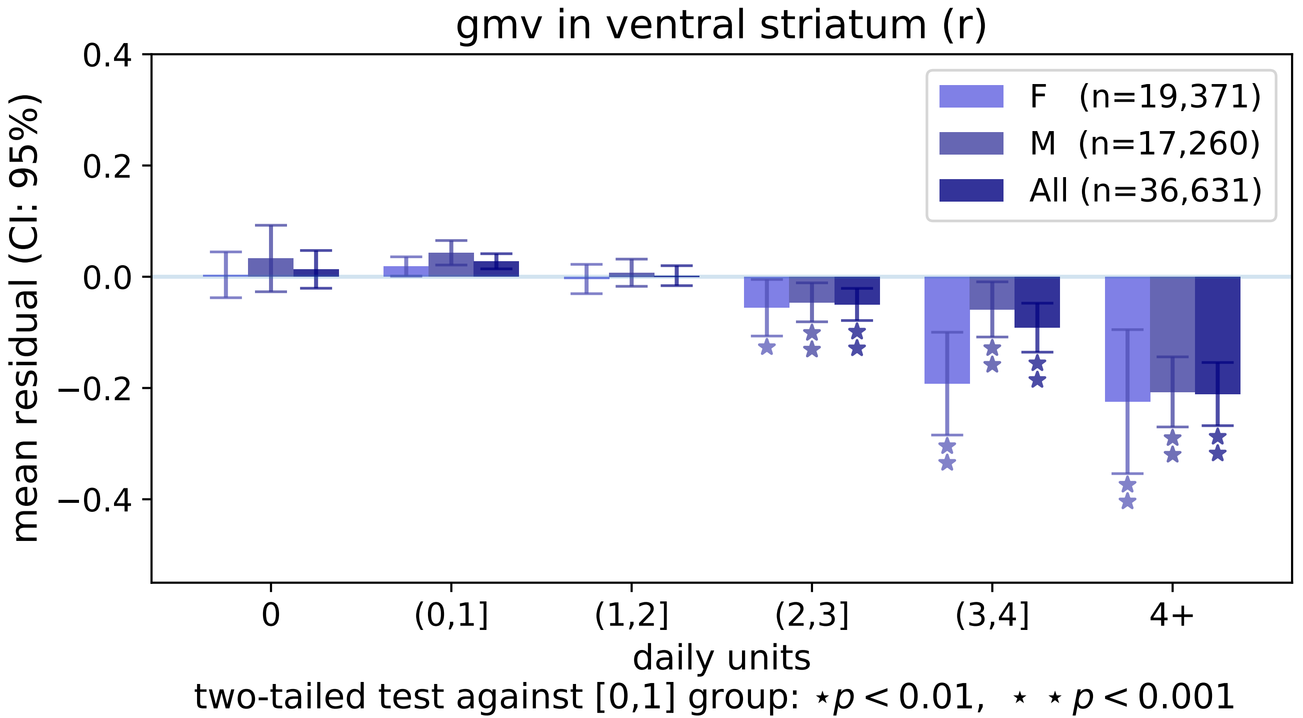
gmv in ventral striatum (r)
0.4
0.2
0.0
−0.2
−0.4
mean residual (CI: 95%)
F (n=19,371)
M (n=17,260)
All (n=36,631)
0
(0,1]
(1,2]
(2,3]
(3,4]
4+
daily units
two-tailed test against [0,1] group: ⋆ $p < 0.01$, ⋆ ⋆ $p < 0.001$

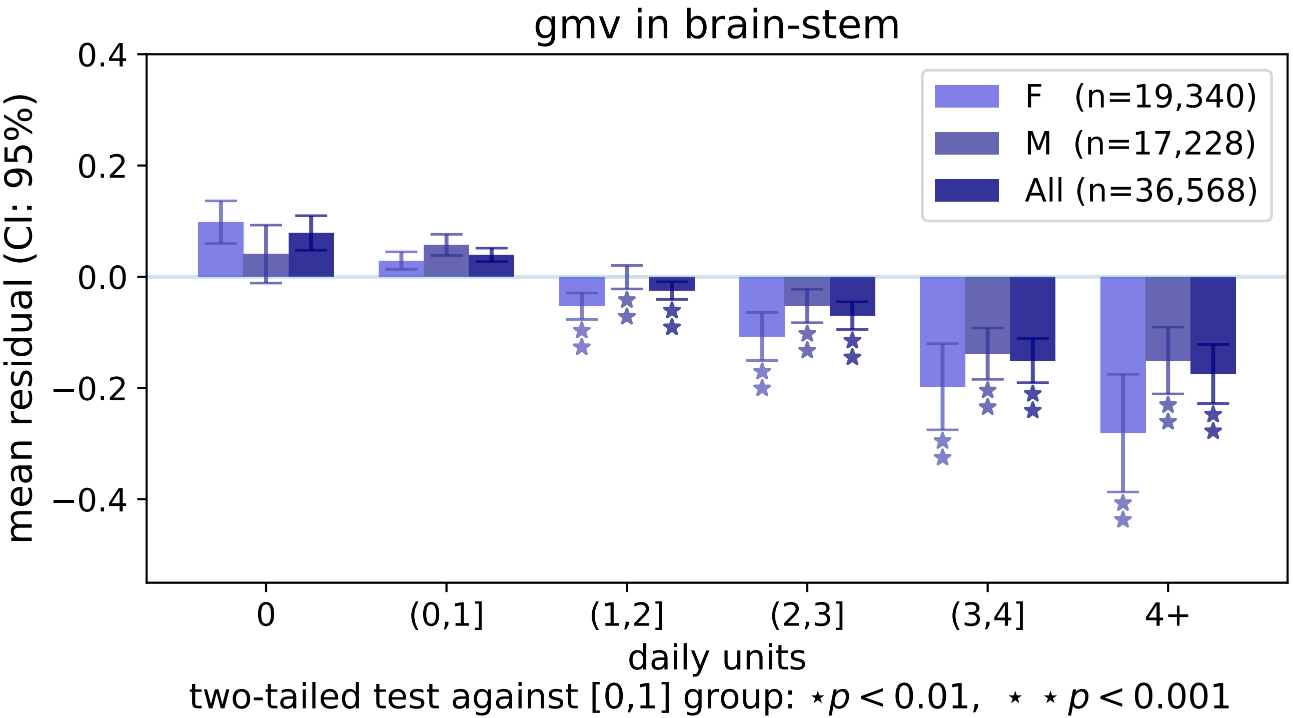
gmv in brain-stem
mean residual (CI: 95%)
0.4
0.2
0.0
−0.2
−0.4
F (n=19,340)
M (n=17,228)
All (n=36,568)
0
(0,1]
(1,2]
(2,3]
(3,4]
4+
daily units
two-tailed test against [0,1] group: ⋆p < 0.01, ⋆ ⋆ p < 0.001

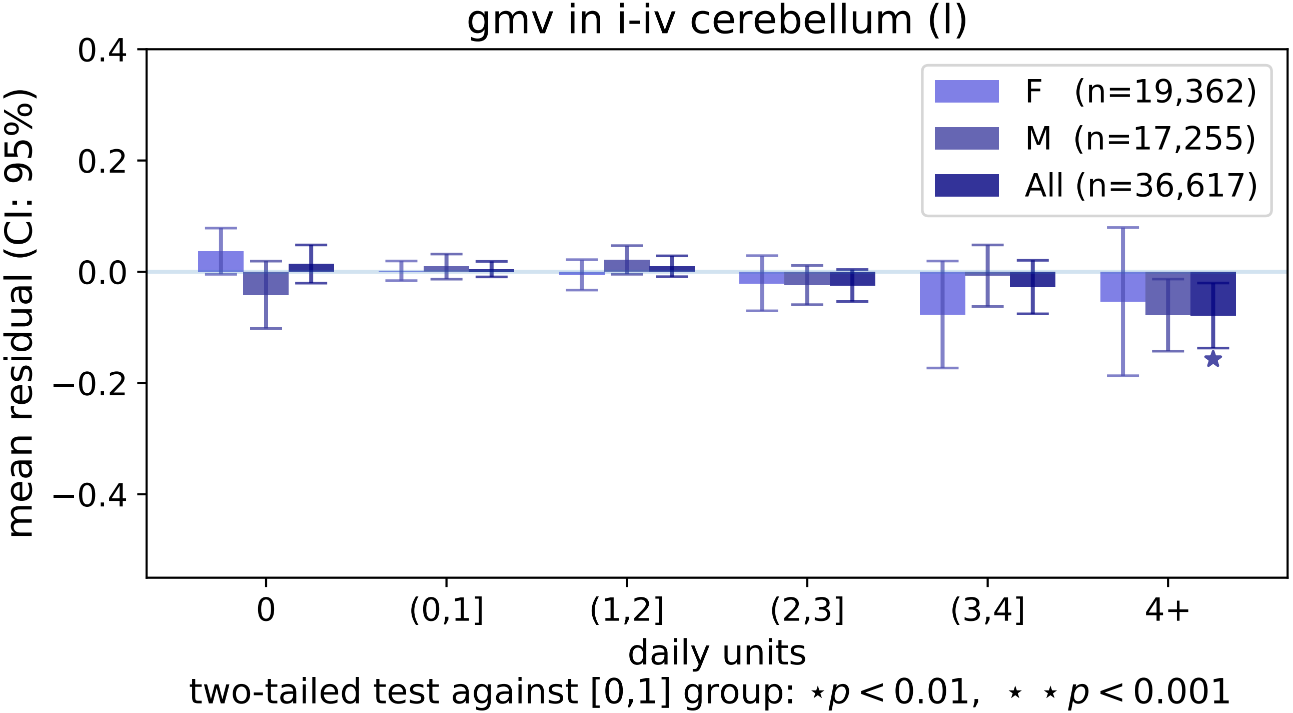
gmv in i-iv cerebellum (l)
mean residual (CI: 95%)
0.4
0.2
0.0
−0.2
−0.4
F (n=19,362)
M (n=17,255)
All (n=36,617)
0
(0,1]
(1,2]
(2,3]
(3,4]
4+
daily units
two-tailed test against [0,1] group: ⋆p < 0.01, ⋆ ⋆ p < 0.001

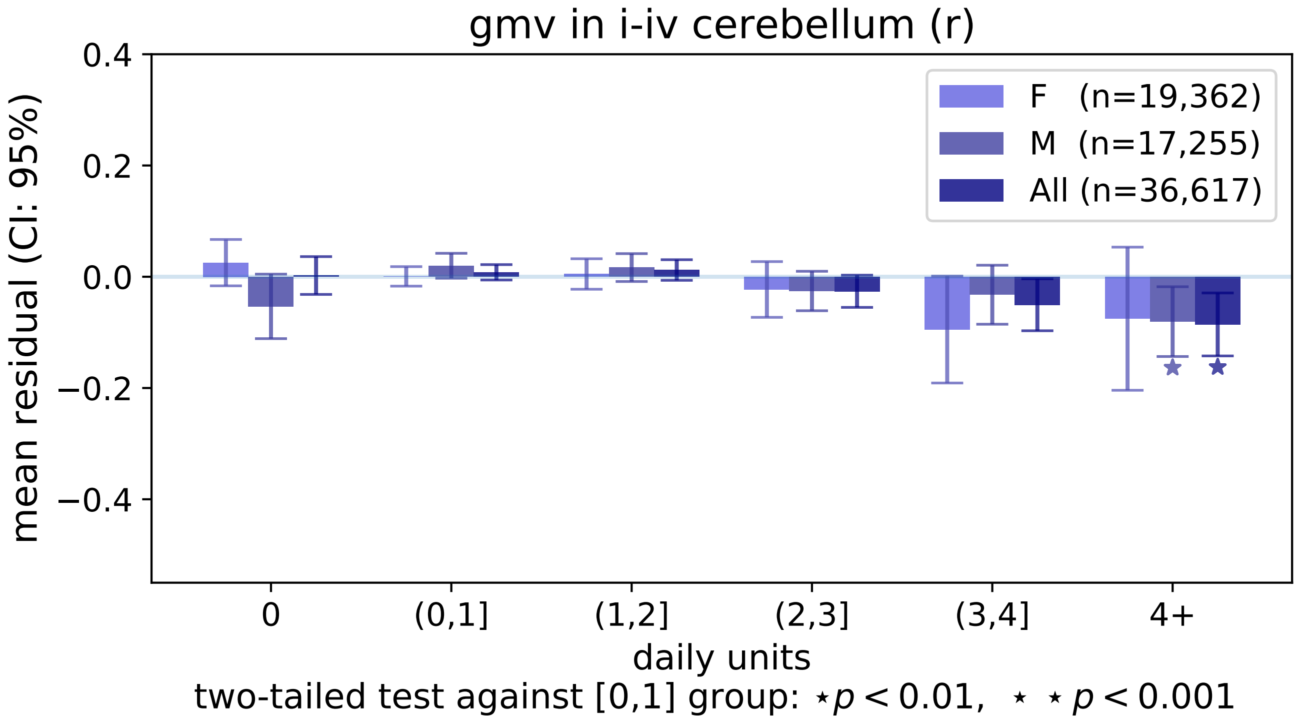
gmv in i-iv cerebellum (r)
0.4
0.2
0.0
−0.2
−0.4
mean residual (CI: 95%)
F (n=19,362)
M (n=17,255)
All (n=36,617)
0
(0,1]
(1,2]
(2,3]
(3,4]
4+
daily units
two-tailed test against [0,1] group: ⋆p < 0.01, ⋆ ⋆ p < 0.001

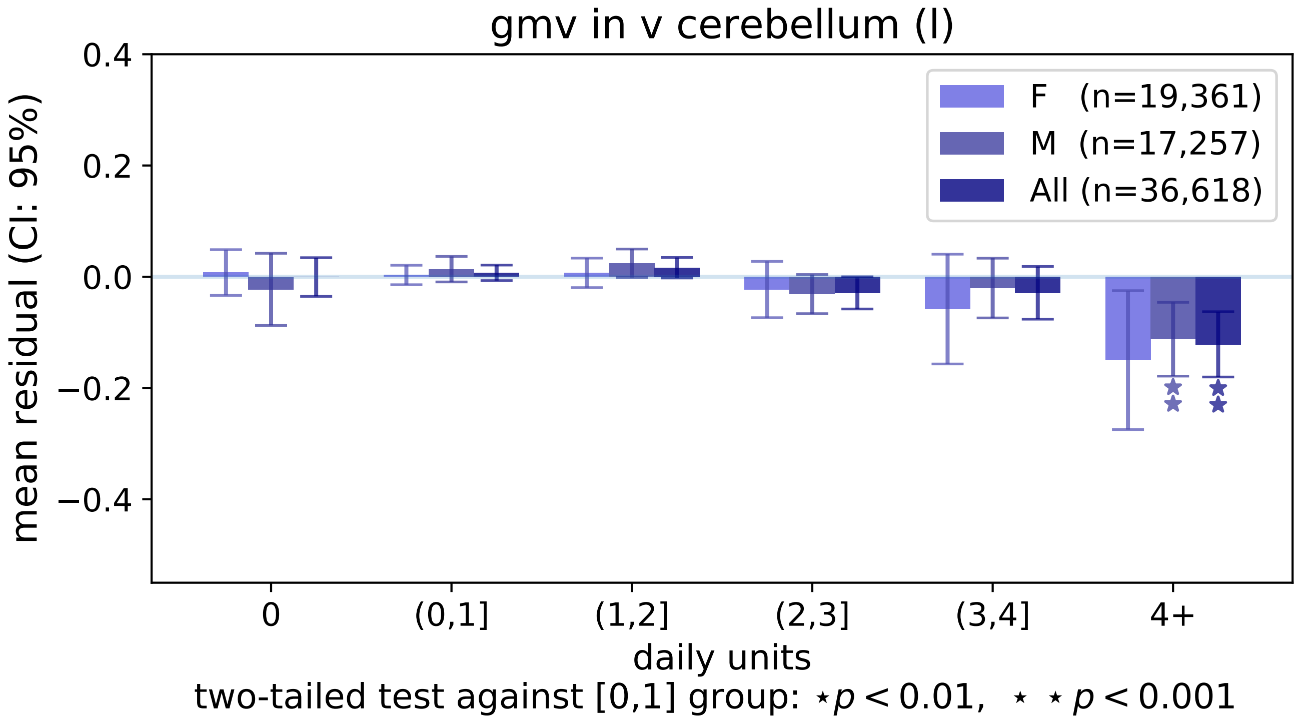
gmv in v cerebellum (l)
mean residual (CI: 95%)
0.4
0.2
0.0
−0.2
−0.4
F (n=19,361)
M (n=17,257)
All (n=36,618)
0
(0,1]
(1,2]
(2,3]
(3,4]
4+
daily units
two-tailed test against [0,1] group: ⋆p < 0.01, ⋆ ⋆ p < 0.001

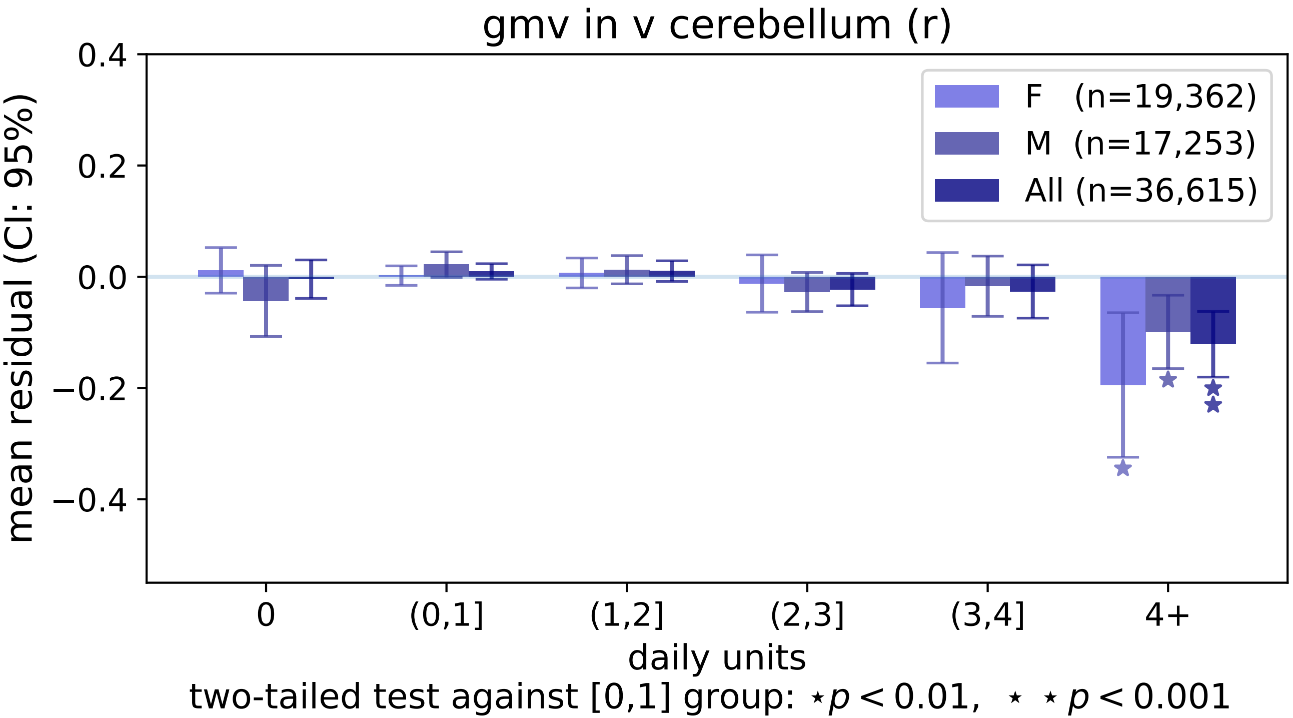
gmv in v cerebellum (r)
mean residual (CI: 95%)
0.4
0.2
0.0
−0.2
−0.4
F (n=19,362)
M (n=17,253)
All (n=36,615)
0
(0,1]
(1,2]
(2,3]
(3,4]
4+
daily units
two-tailed test against [0,1] group: ⋆ p < 0.01, ⋆ ⋆ p < 0.001

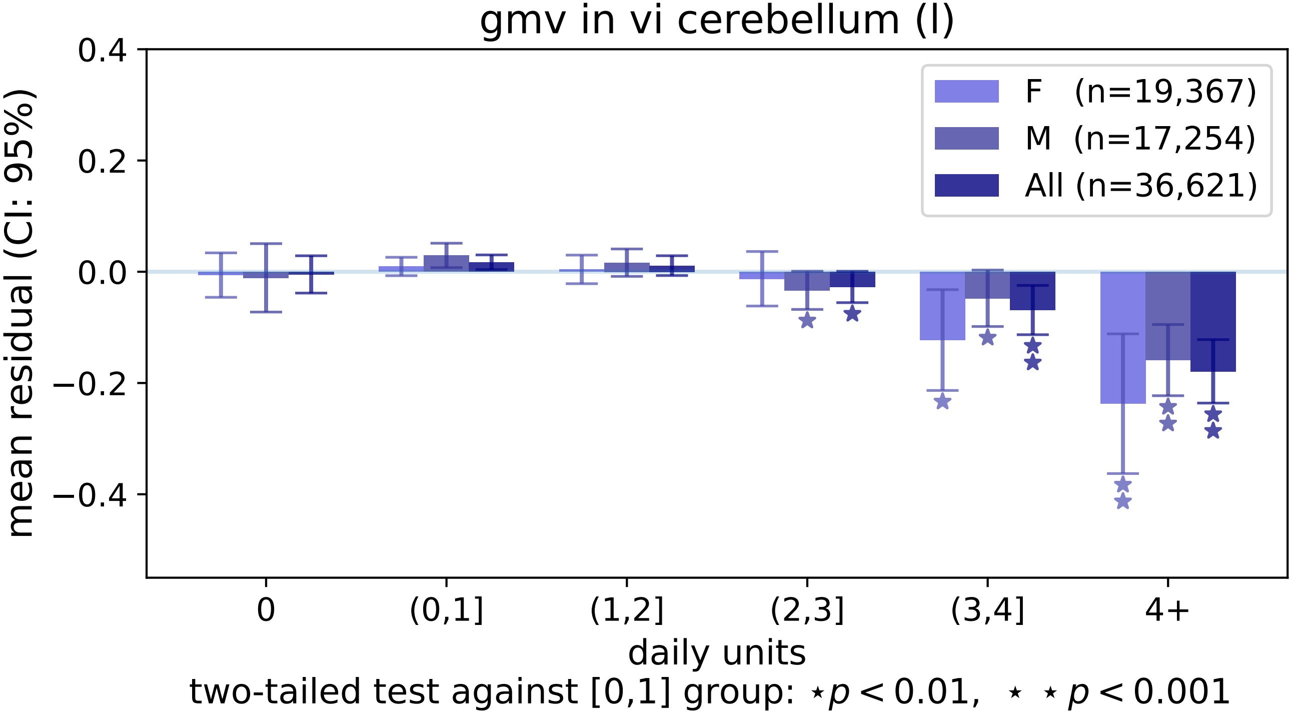

gmv in vi cerebellum (l)
0.4
0.2
0.0
−0.2
−0.4
mean residual (CI: 95%)
F (n=19,367)
M (n=17,254)
All (n=36,621)
0
(0,1]
(1,2]
(2,3]
(3,4]
4+
daily units
two-tailed test against [0,1] group: ⋆ $p < 0.01$, ⋆ ⋆ $p < 0.001$

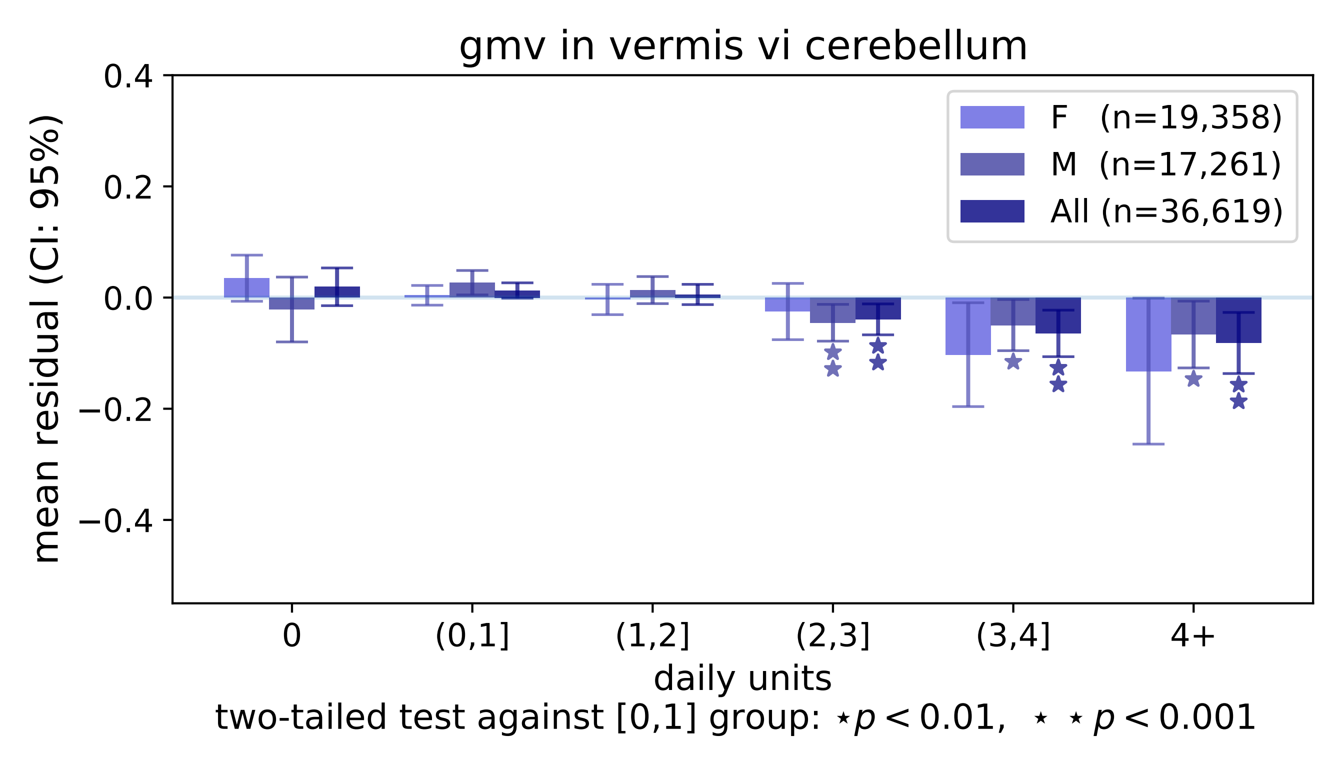
gmv in vermis vi cerebellum
mean residual (CI: 95%)
0.4
0.2
0.0
−0.2
−0.4
F (n=19,358)
M (n=17,261)
All (n=36,619)
0
(0,1]
(1,2]
(2,3]
(3,4]
4+
daily units
two-tailed test against [0,1] group: ⋆ p < 0.01, ⋆ ⋆ p < 0.001

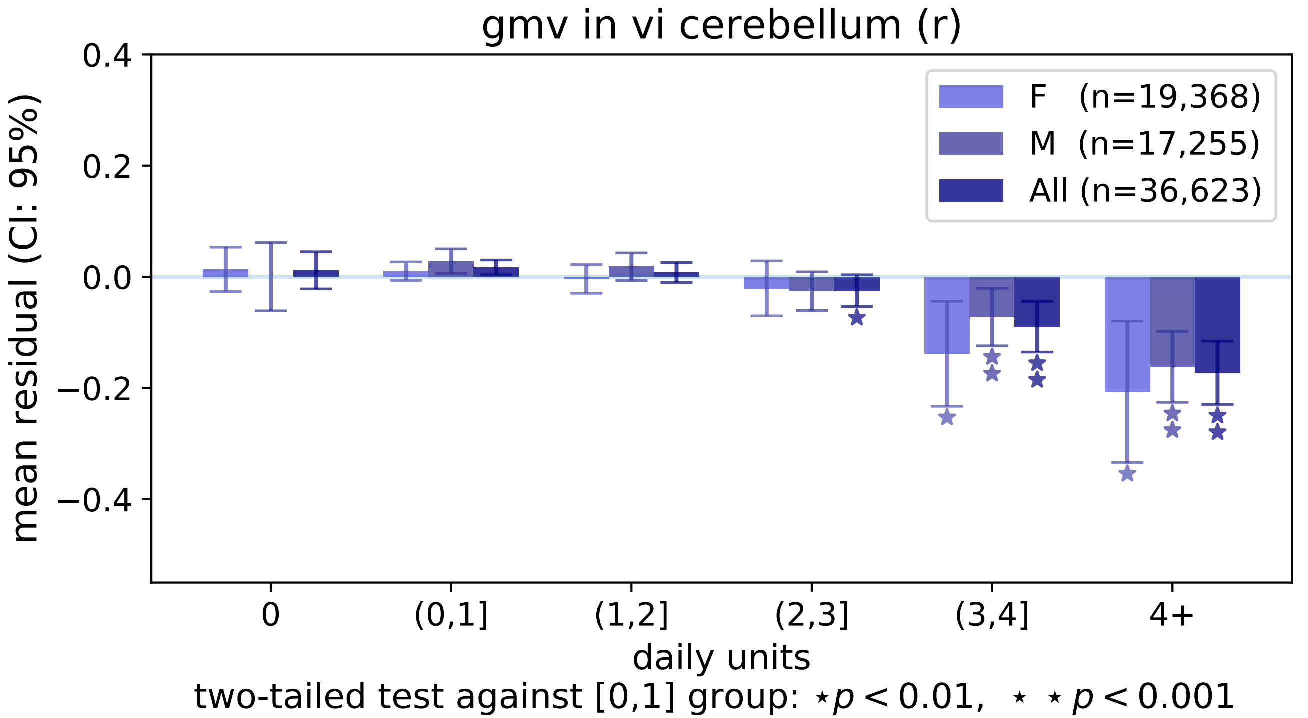

gmv in vi cerebellum (r)
mean residual (CI: 95%)
0.4
0.2
0.0
−0.2
−0.4
F (n=19,368)
M (n=17,255)
All (n=36,623)
0
(0,1]
(1,2]
(2,3]
(3,4]
4+
daily units
two-tailed test against [0,1] group: ⋆ p < 0.01, ⋆ ⋆ p < 0.001

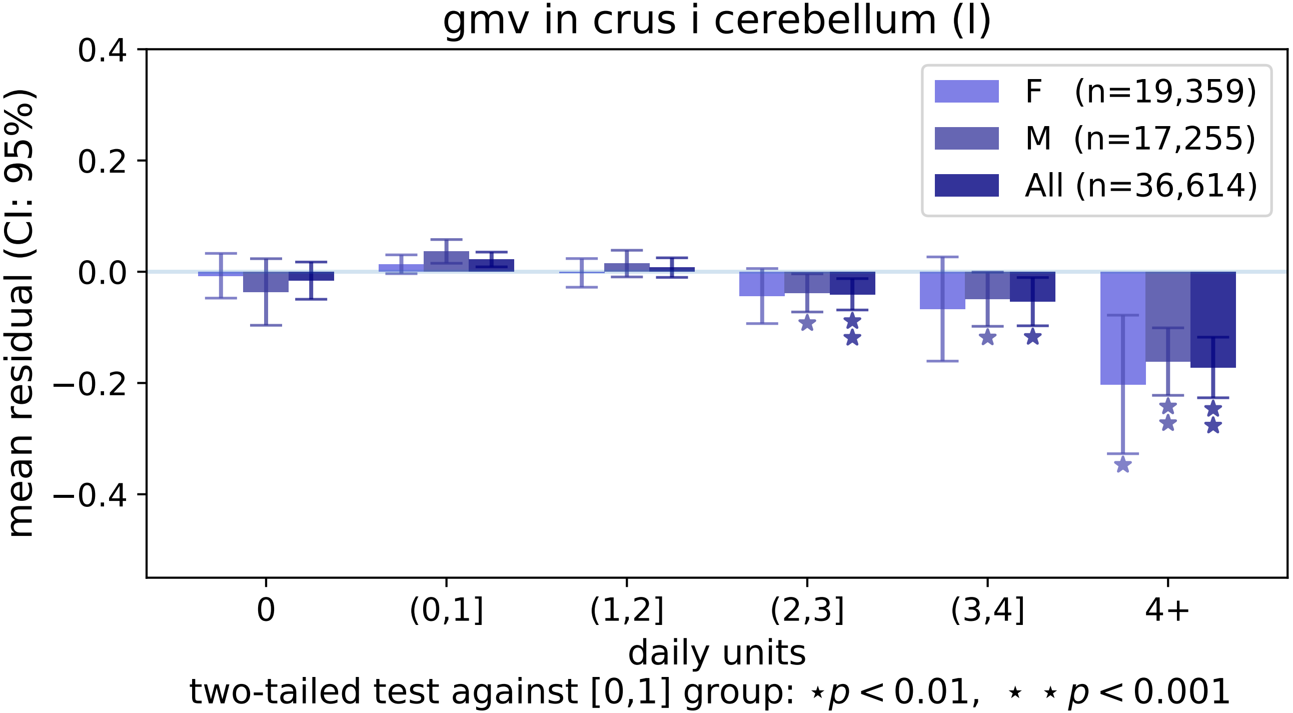
gmv in crus i cerebellum (l)
mean residual (CI: 95%)
0.4
0.2
0.0
−0.2
−0.4
F (n=19,359)
M (n=17,255)
All (n=36,614)
0
(0,1]
(1,2]
(2,3]
(3,4]
4+
daily units
two-tailed test against [0,1] group: ⋆p < 0.01, ⋆ ⋆ p < 0.001

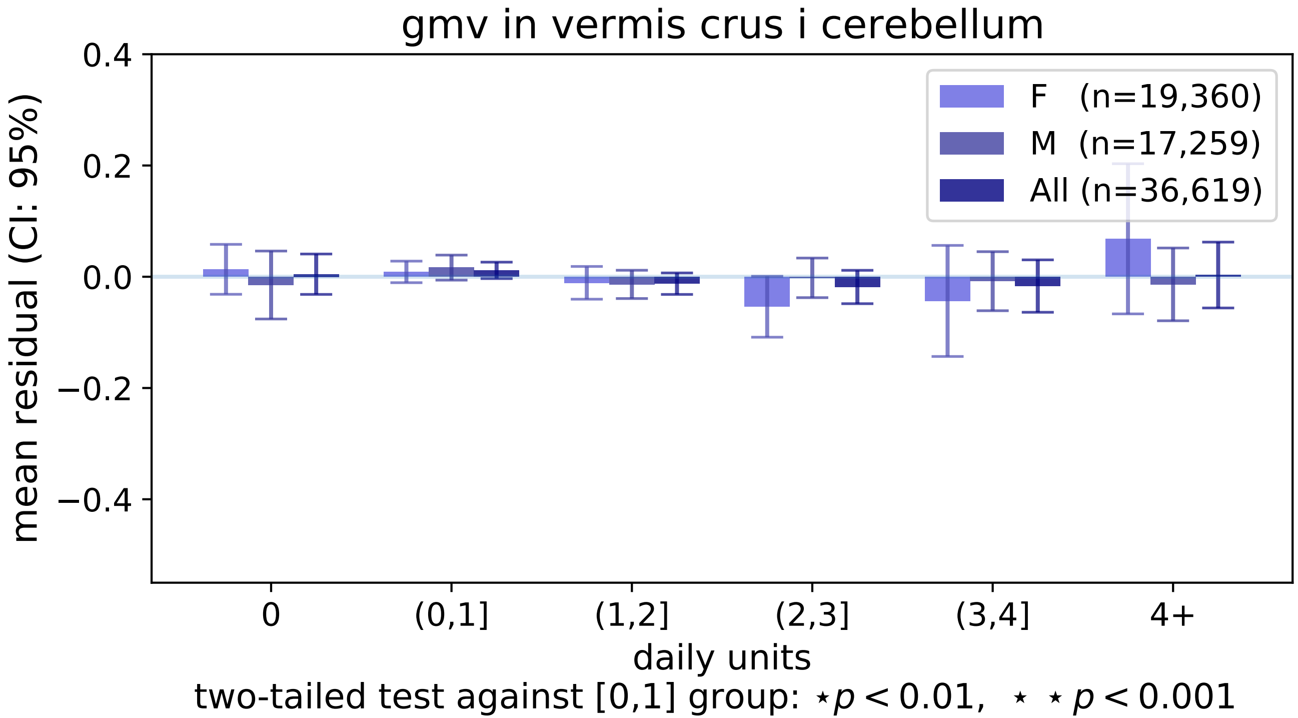
gmv in vermis crus i cerebellum
mean residual (CI: 95%)
0.4
0.2
0.0
−0.2
−0.4
F (n=19,360)
M (n=17,259)
All (n=36,619)
0
(0,1]
(1,2]
(2,3]
(3,4]
4+
daily units
two-tailed test against [0,1] group: ⋆p < 0.01, ⋆ ⋆ p < 0.001

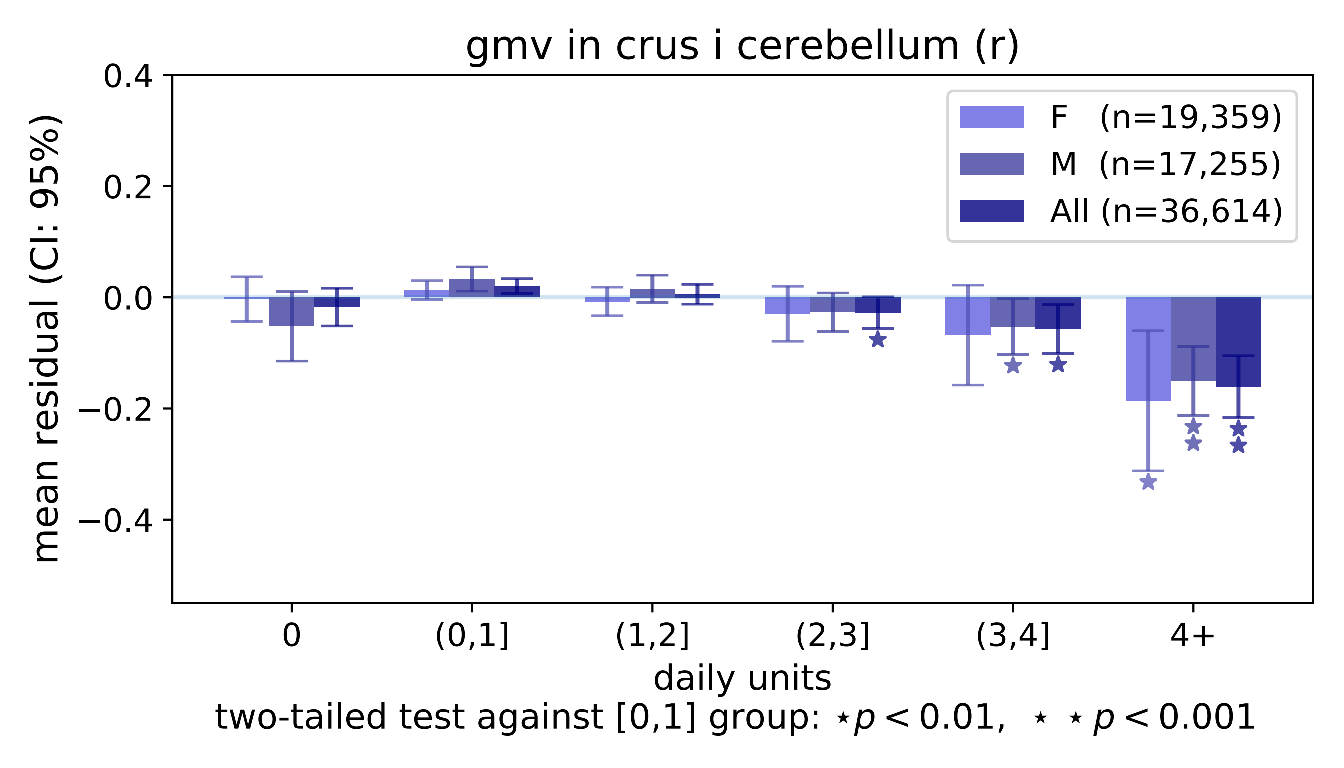

gmv in crus i cerebellum (r)
0.4
0.2
0.0
−0.2
−0.4
mean residual (CI: 95%)
F (n=19,359)
M (n=17,255)
All (n=36,614)
0
(0,1]
(1,2]
(2,3]
(3,4]
4+
daily units
two-tailed test against [0,1] group: ⋆ p < 0.01, ⋆ ⋆ p < 0.001

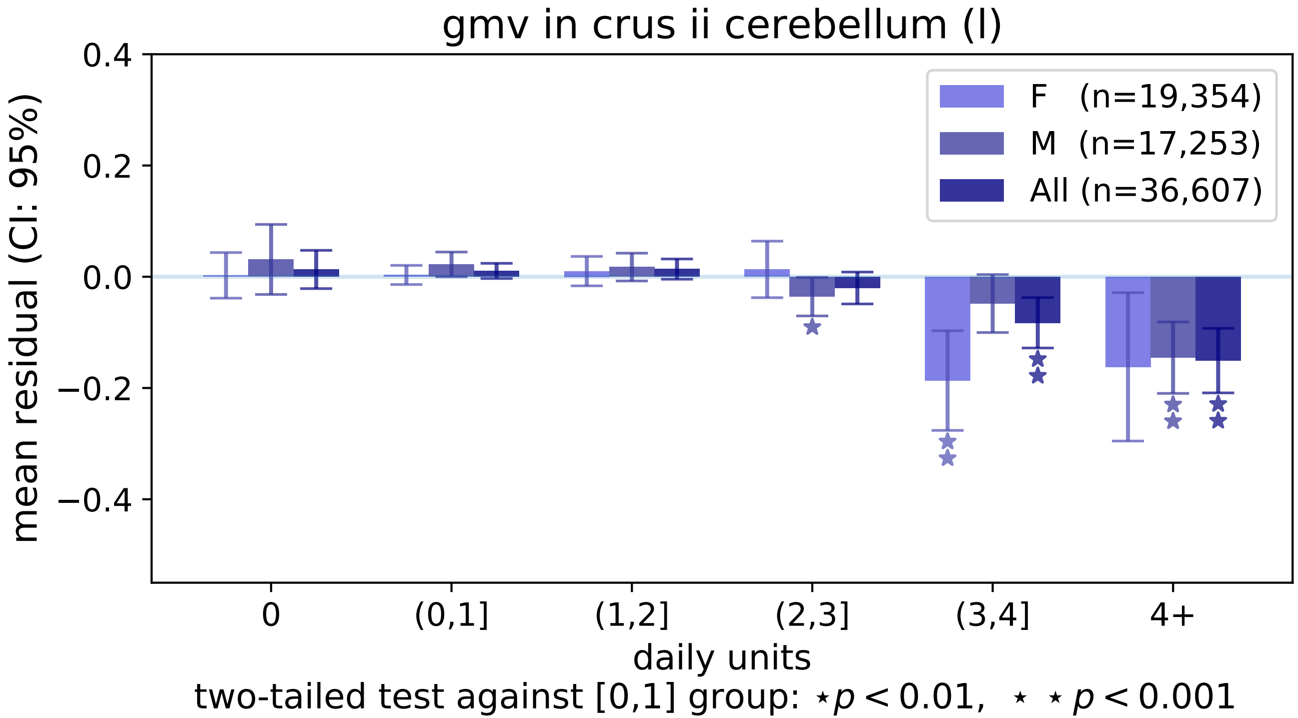
gmv in crus ii cerebellum (l)
mean residual (CI: 95%)
0.4
0.2
0.0
−0.2
−0.4
F (n=19,354)
M (n=17,253)
All (n=36,607)
0
(0,1]
(1,2]
(2,3]
(3,4]
4+
daily units
two-tailed test against [0,1] group: ⋆ p < 0.01, ⋆ ⋆ p < 0.001

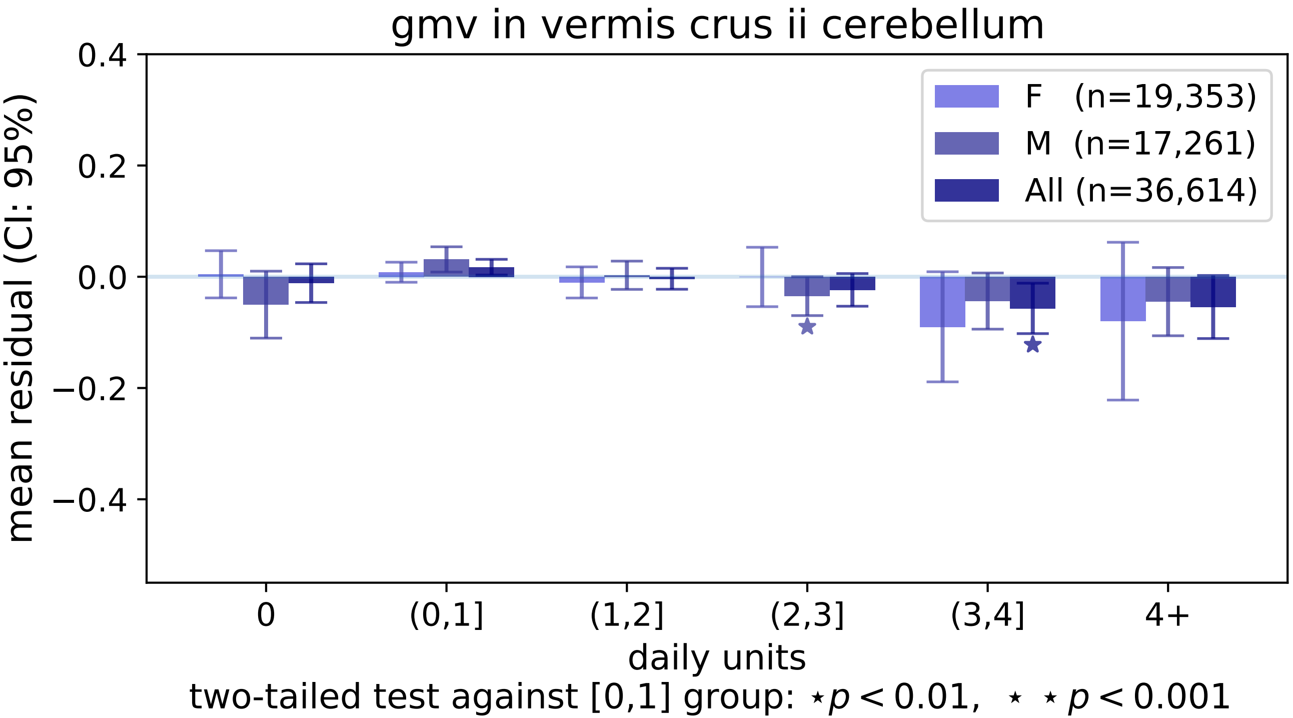

gmv in vermis crus ii cerebellum
mean residual (CI: 95%)
0.4
0.2
0.0
−0.2
−0.4
F (n=19,353)
M (n=17,261)
All (n=36,614)
0
(0,1]
(1,2]
(2,3]
(3,4]
4+
daily units
two-tailed test against [0,1] group: ⋆p < 0.01, ⋆ ⋆ p < 0.001

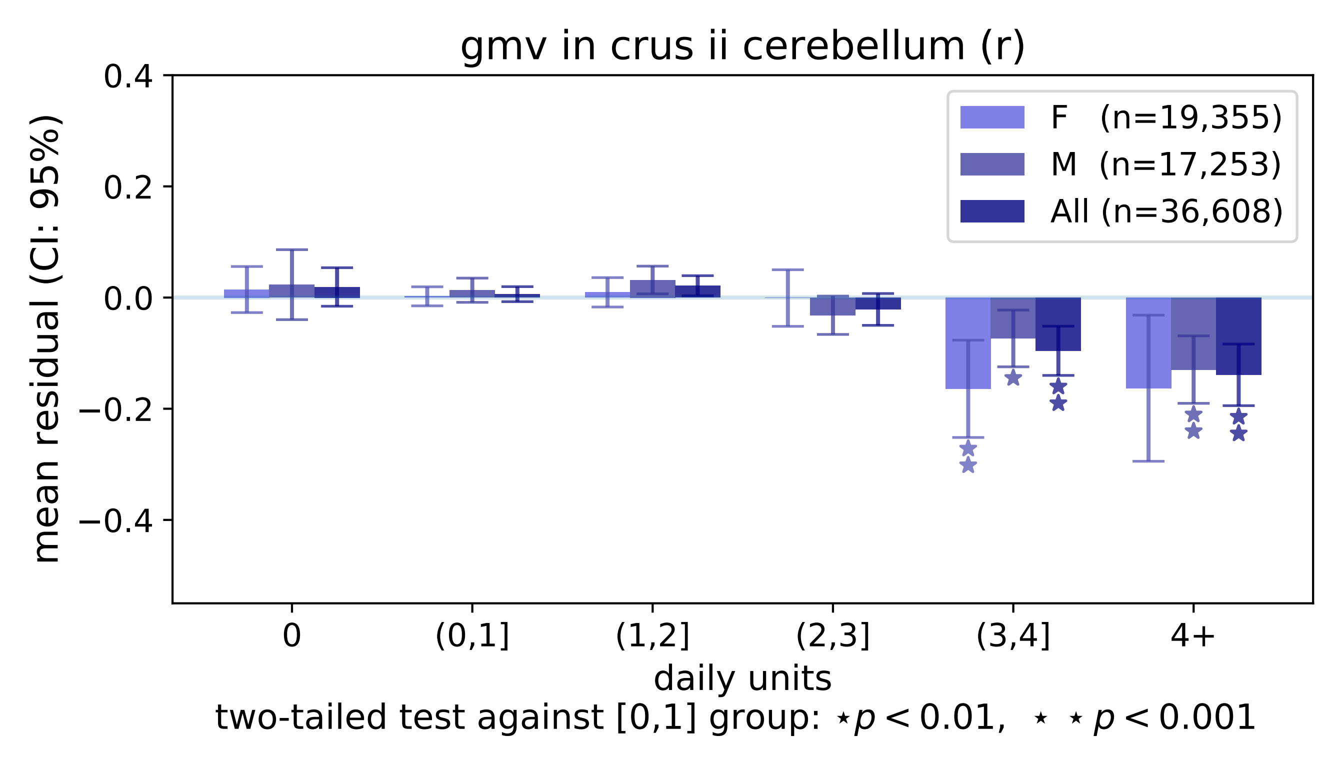
gmv in crus ii cerebellum (r)
0.4
0.2
0.0
−0.2
−0.4
mean residual (CI: 95%)
F (n=19,355)
M (n=17,253)
All (n=36,608)
0
(0,1]
(1,2]
(2,3]
(3,4]
4+
daily units
two-tailed test against [0,1] group: ⋆p < 0.01, ⋆ ⋆p < 0.001

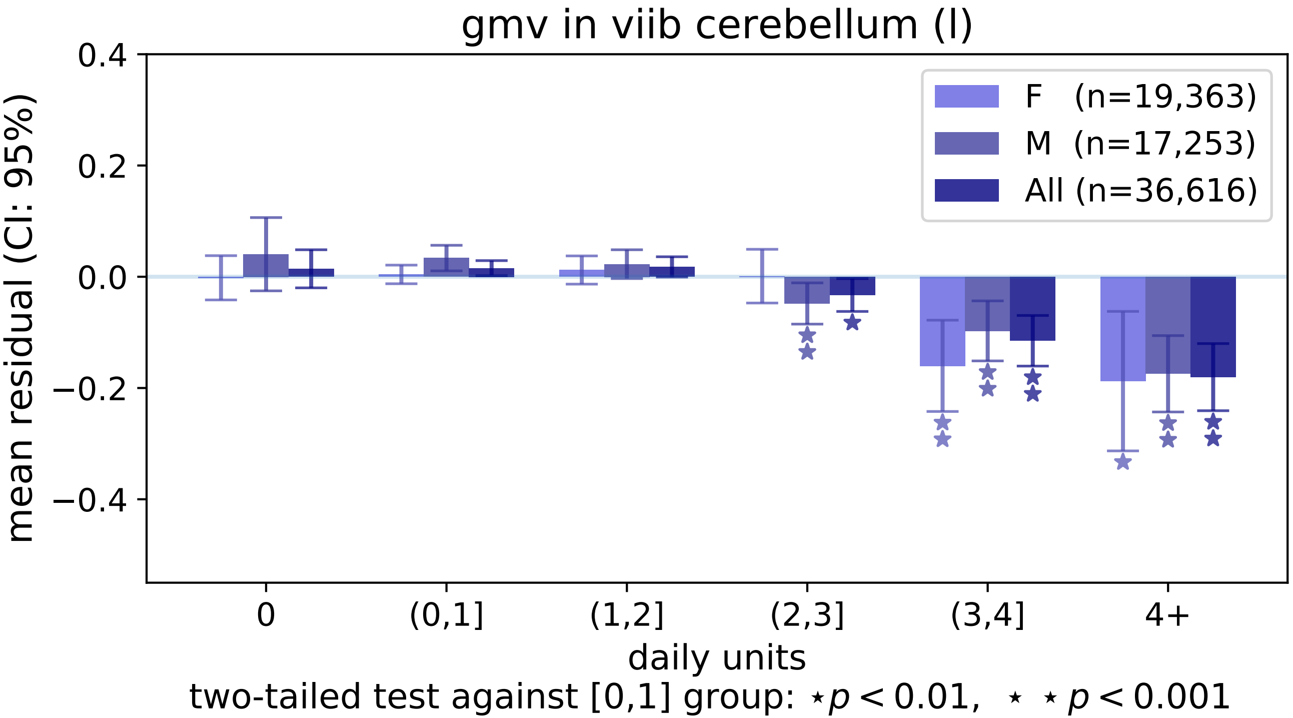
gmv in viib cerebellum (l)
mean residual (CI: 95%)
0.4
0.2
0.0
−0.2
−0.4
F (n=19,363)
M (n=17,253)
All (n=36,616)
0
(0,1]
(1,2]
(2,3]
(3,4]
4+
daily units
two-tailed test against [0,1] group: ⋆p < 0.01, ⋆ ⋆ p < 0.001

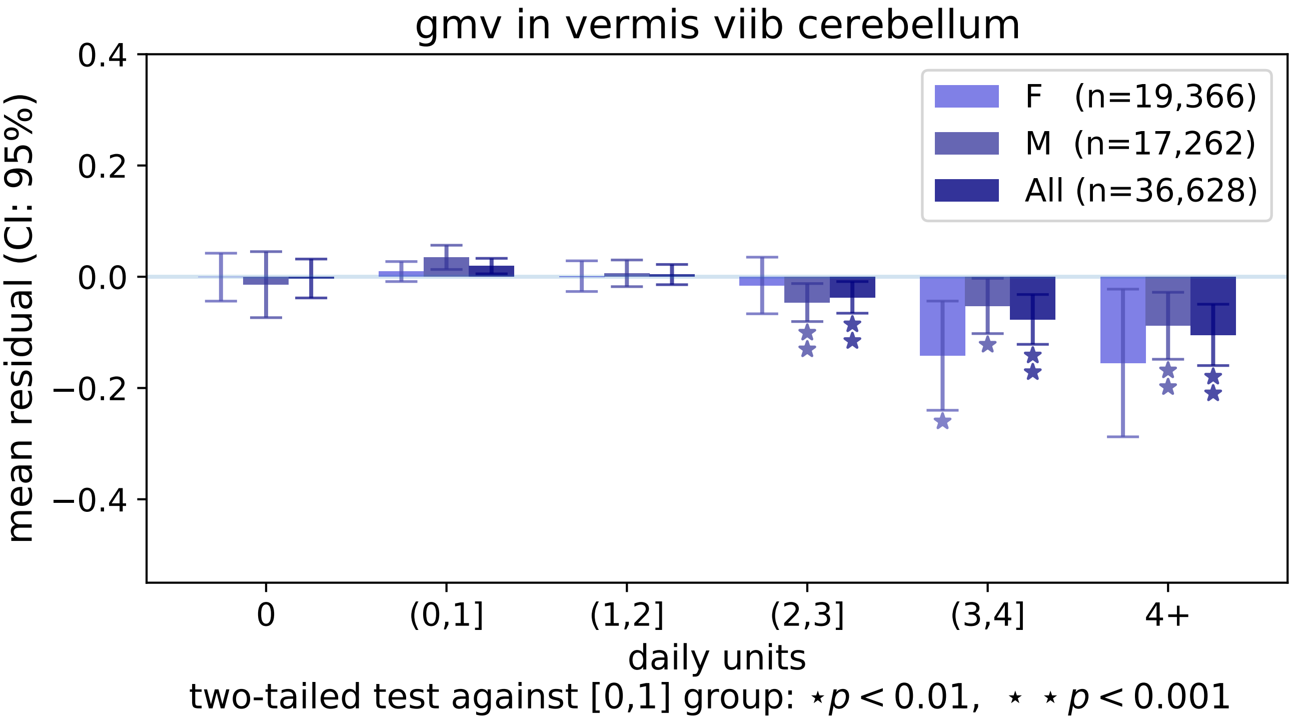
gmv in vermis viib cerebellum
mean residual (CI: 95%)
0.4
0.2
0.0
−0.2
−0.4
F (n=19,366)
M (n=17,262)
All (n=36,628)
0
(0,1]
(1,2]
(2,3]
(3,4]
4+
daily units
two-tailed test against [0,1] group: ⋆ p < 0.01, ⋆ ⋆ p < 0.001

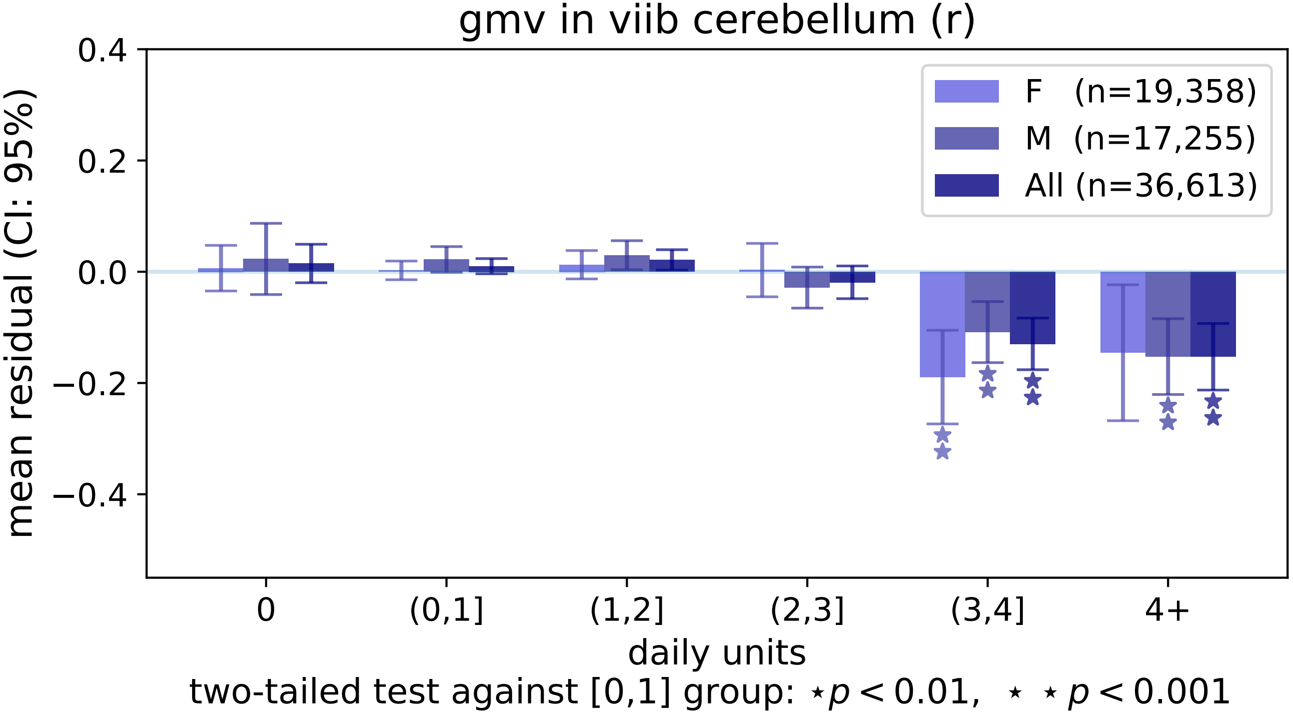
gmv in viib cerebellum (r)
mean residual (CI: 95%)
0.4
0.2
0.0
−0.2
−0.4
F (n=19,358)
M (n=17,255)
All (n=36,613)
0
(0,1]
(1,2]
(2,3]
(3,4]
4+
daily units
two-tailed test against [0,1] group: ⋆ p < 0.01, ⋆ ⋆ p < 0.001

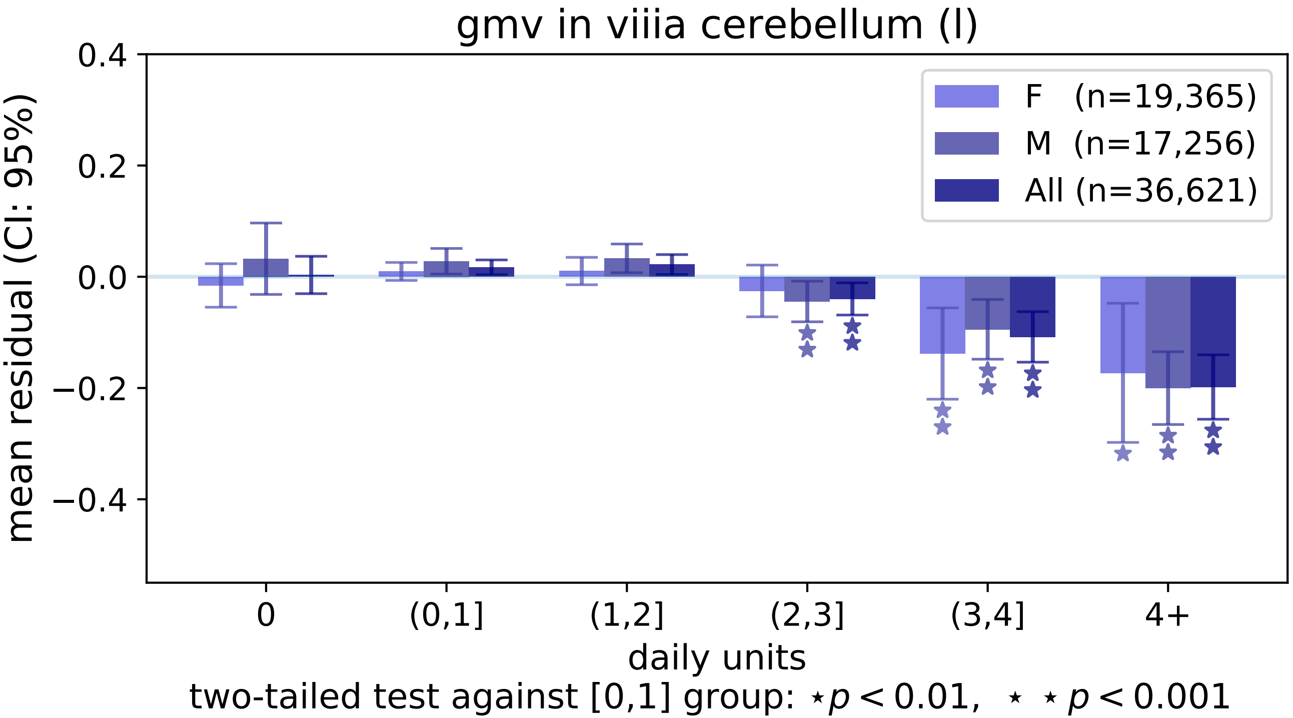
gmv in viiia cerebellum (l)
F (n=19,365)
M (n=17,256)
All (n=36,621)
mean residual (CI: 95%)
0.4
0.2
0.0
−0.2
−0.4
0
(0,1]
(1,2]
(2,3]
(3,4]
4+
daily units
two-tailed test against [0,1] group: ⋆p < 0.01, ⋆ ⋆ p < 0.001

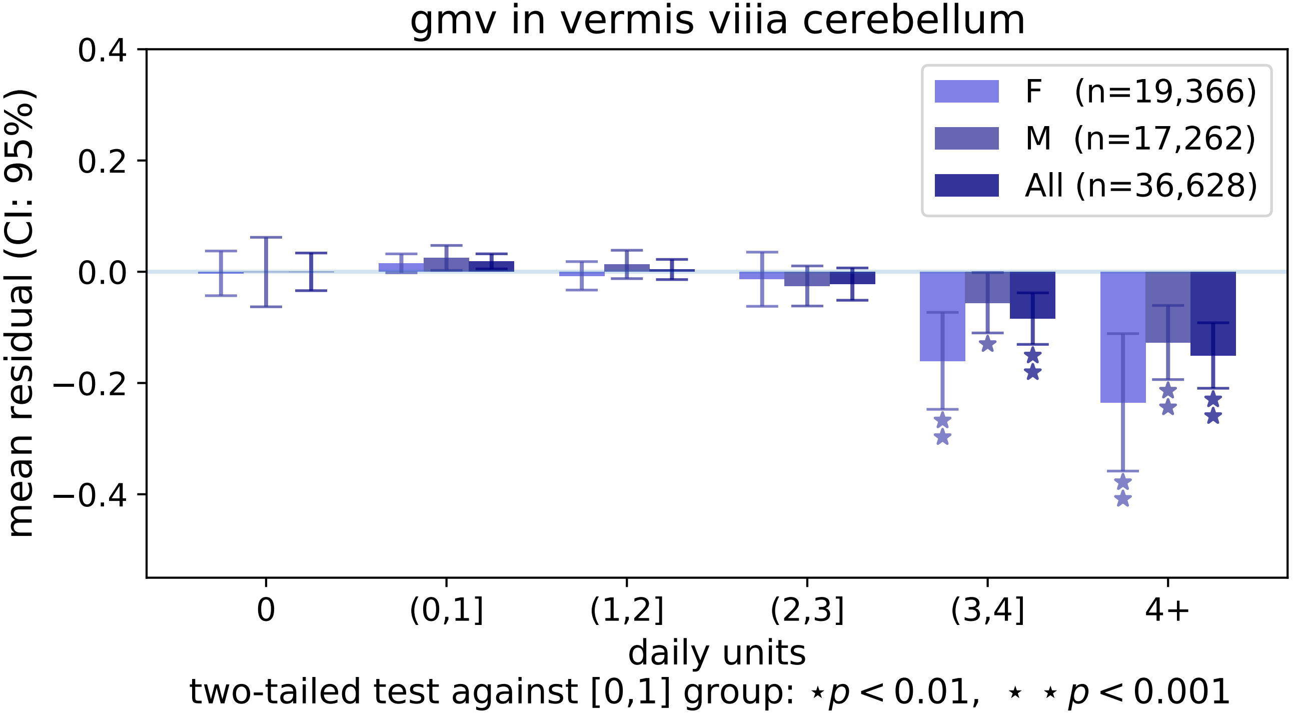
gmv in vermis viiia cerebellum
mean residual (CI: 95%)
0.4
0.2
0.0
−0.2
−0.4
F (n=19,366)
M (n=17,262)
All (n=36,628)
0
(0,1]
(1,2]
(2,3]
(3,4]
4+
daily units
two-tailed test against [0,1] group: ⋆ $p < 0.01$, ⋆ ⋆ $p < 0.001$

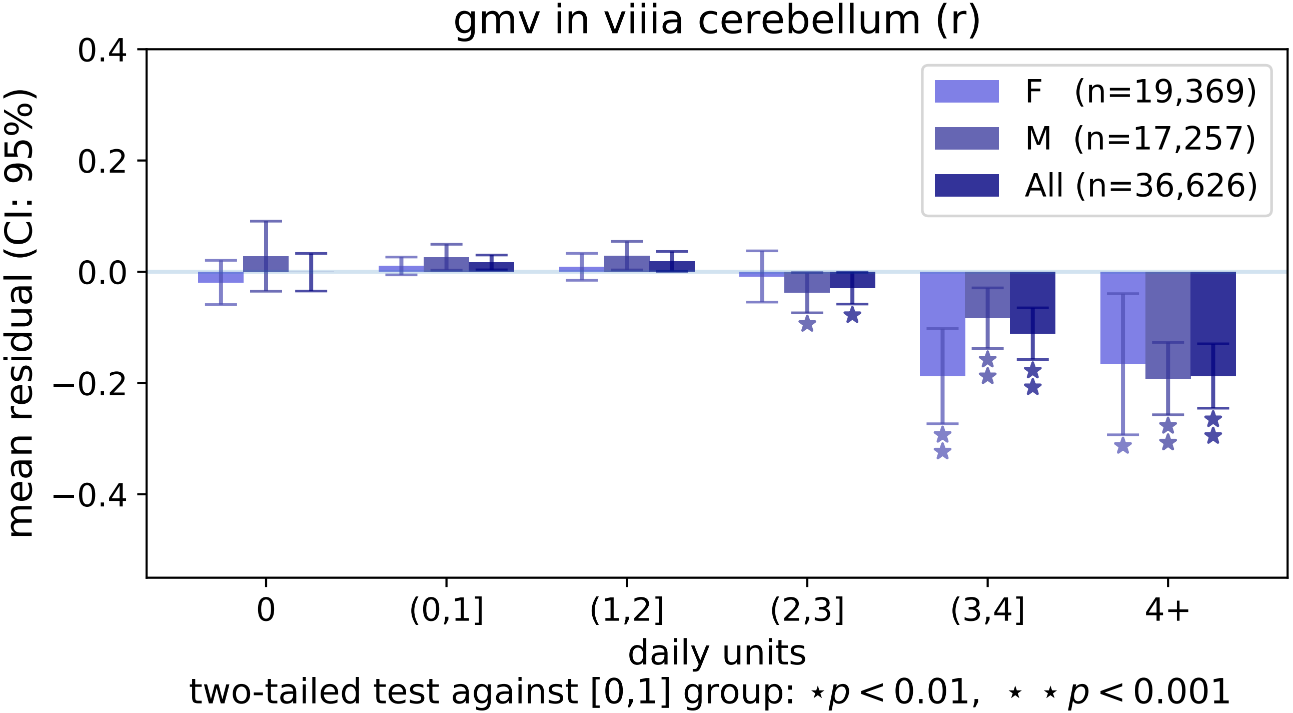
gmv in viiia cerebellum (r)
F (n=19,369)
M (n=17,257)
All (n=36,626)
mean residual (CI: 95%)
0.4
0.2
0.0
−0.2
−0.4
0
(0,1]
(1,2]
(2,3]
(3,4]
4+
daily units
two-tailed test against [0,1] group: ⋆p < 0.01, ⋆ ⋆ p < 0.001

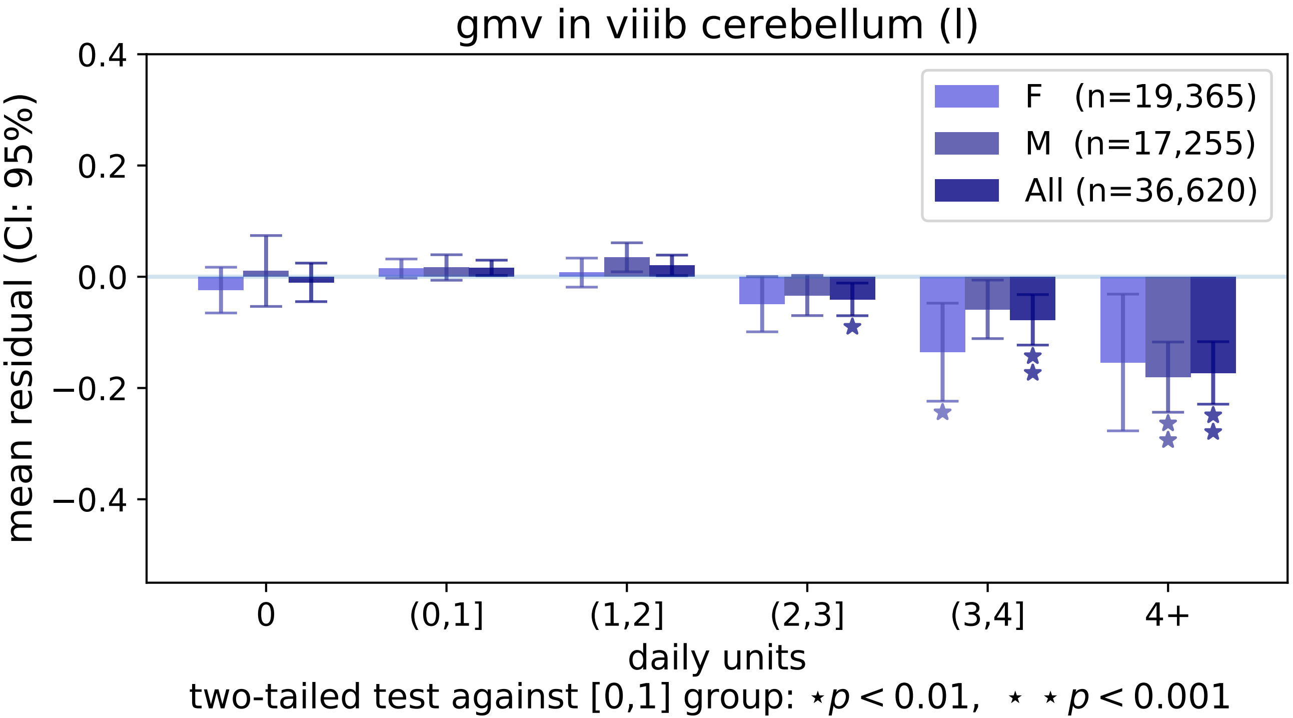

gmv in viiib cerebellum (l)
0.4
0.2
0.0
−0.2
−0.4
mean residual (CI: 95%)
F (n=19,365)
M (n=17,255)
All (n=36,620)
0
(0,1]
(1,2]
(2,3]
(3,4]
4+
daily units
two-tailed test against [0,1] group: ⋆p < 0.01, ⋆ ⋆ p < 0.001

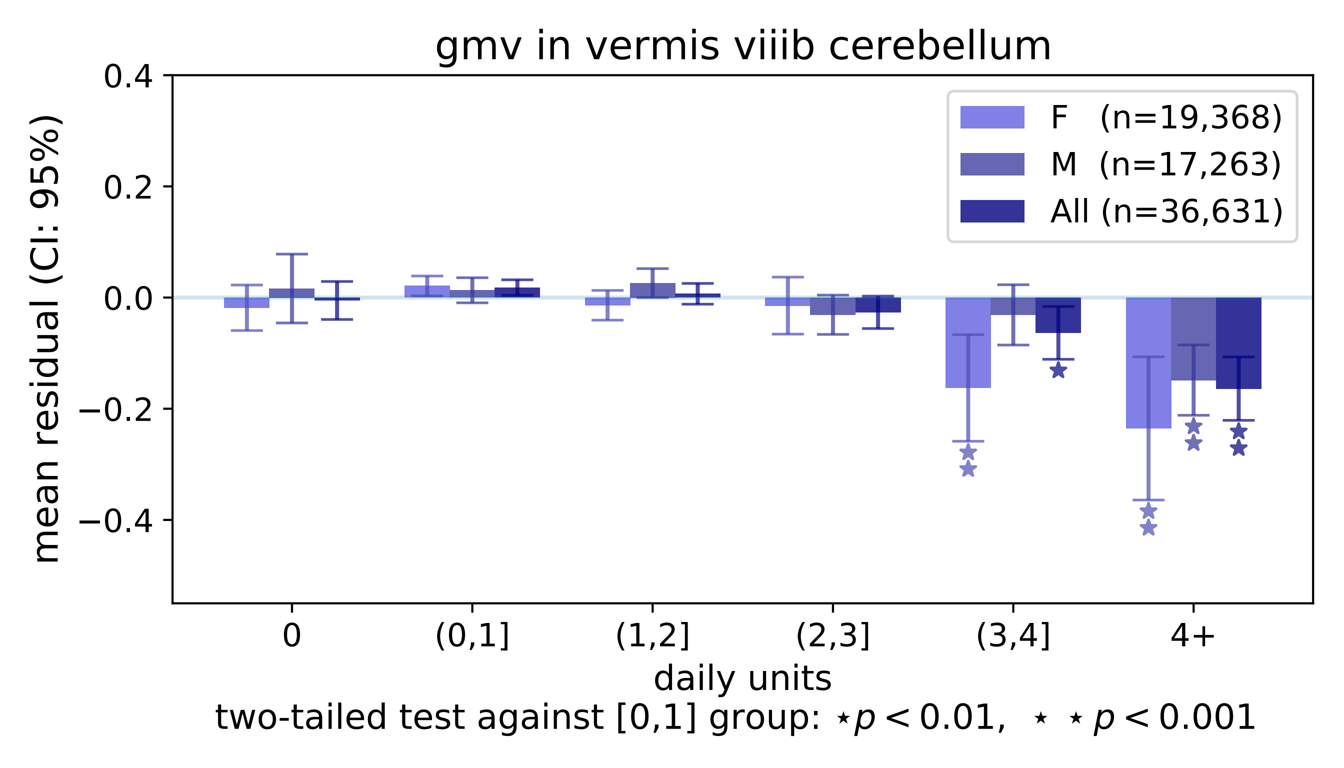
gmv in vermis viiib cerebellum
0.4
0.2
0.0
−0.2
−0.4
mean residual (CI: 95%)
F (n=19,368)
M (n=17,263)
All (n=36,631)
0
(0,1]
(1,2]
(2,3]
(3,4]
4+
daily units
two-tailed test against [0,1] group: ⋆p < 0.01, ⋆ ⋆p < 0.001

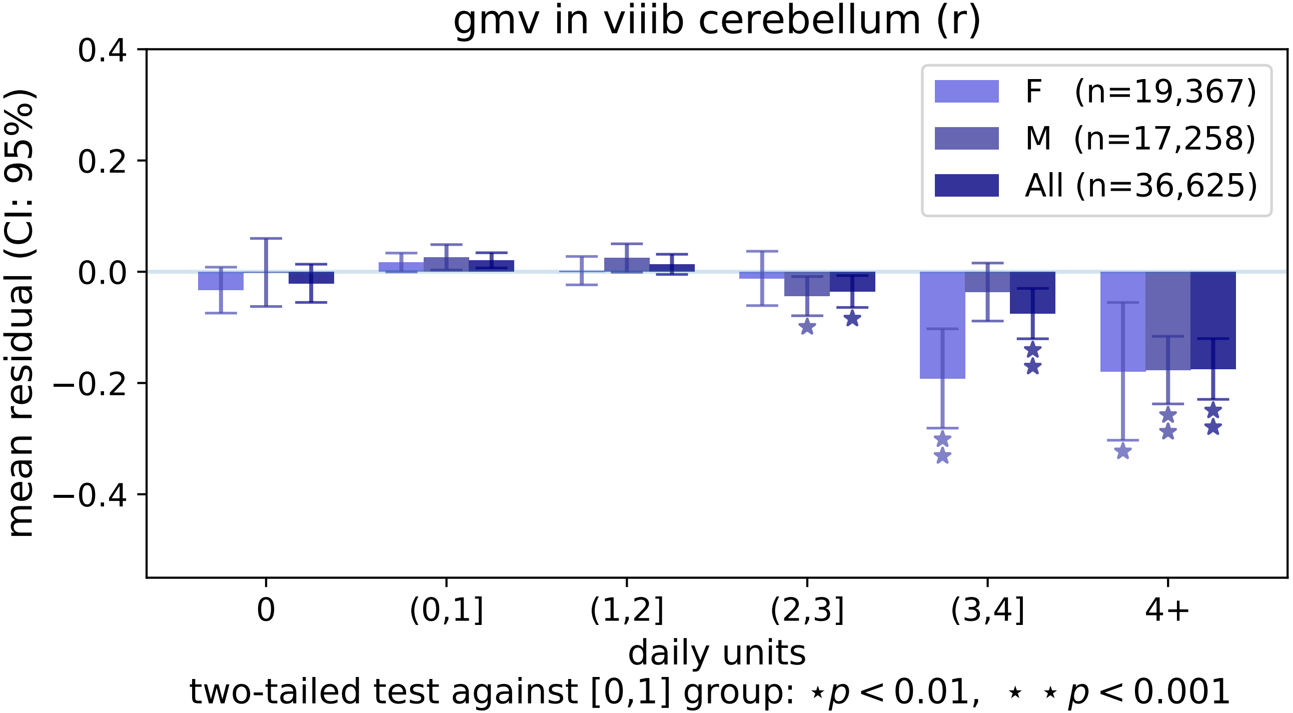
gmv in viiib cerebellum (r)
mean residual (CI: 95%)
0.4
0.2
0.0
−0.2
−0.4
F (n=19,367)
M (n=17,258)
All (n=36,625)
0
(0,1]
(1,2]
(2,3]
(3,4]
4+
daily units
two-tailed test against [0,1] group: ⋆$p<0.01$, ⋆ ⋆$p<0.001$

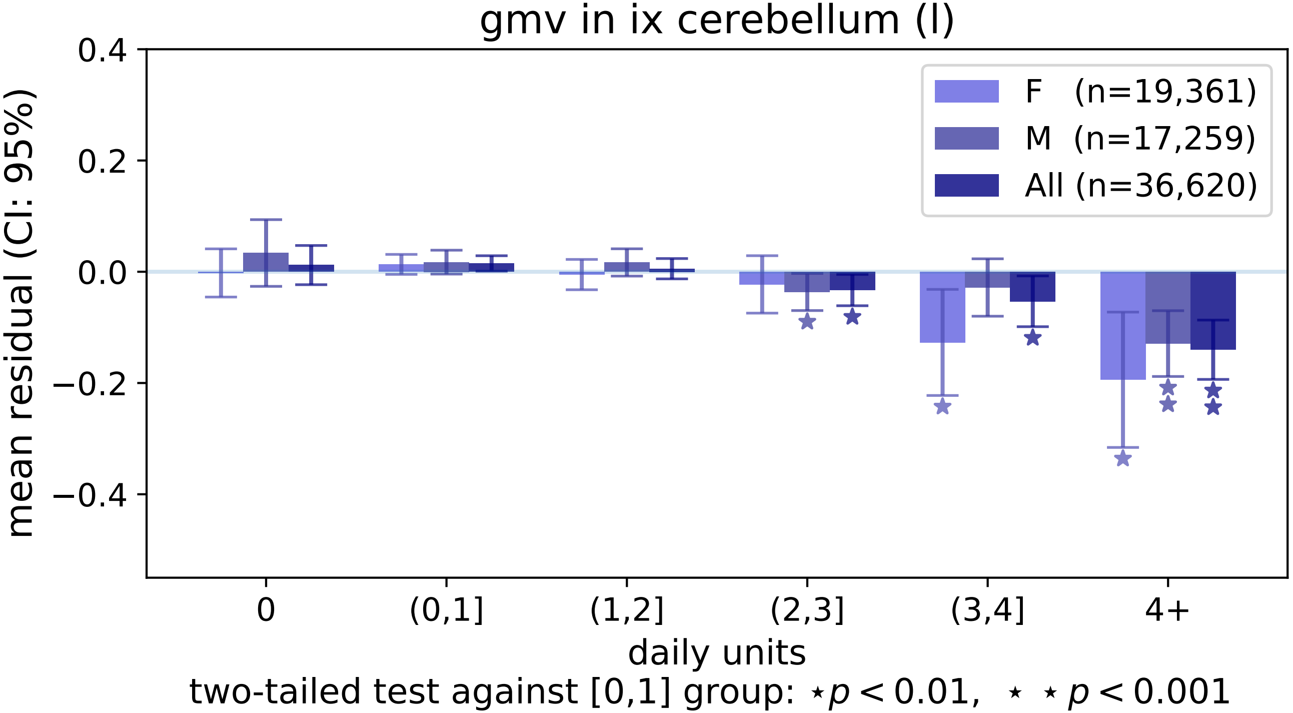
gmv in ix cerebellum (l)
0.4
0.2
0.0
−0.2
−0.4
mean residual (CI: 95%)
F (n=19,361)
M (n=17,259)
All (n=36,620)
0
(0,1]
(1,2]
(2,3]
(3,4]
4+
daily units
two-tailed test against [0,1] group: ⋆p < 0.01, ⋆ ⋆ p < 0.001

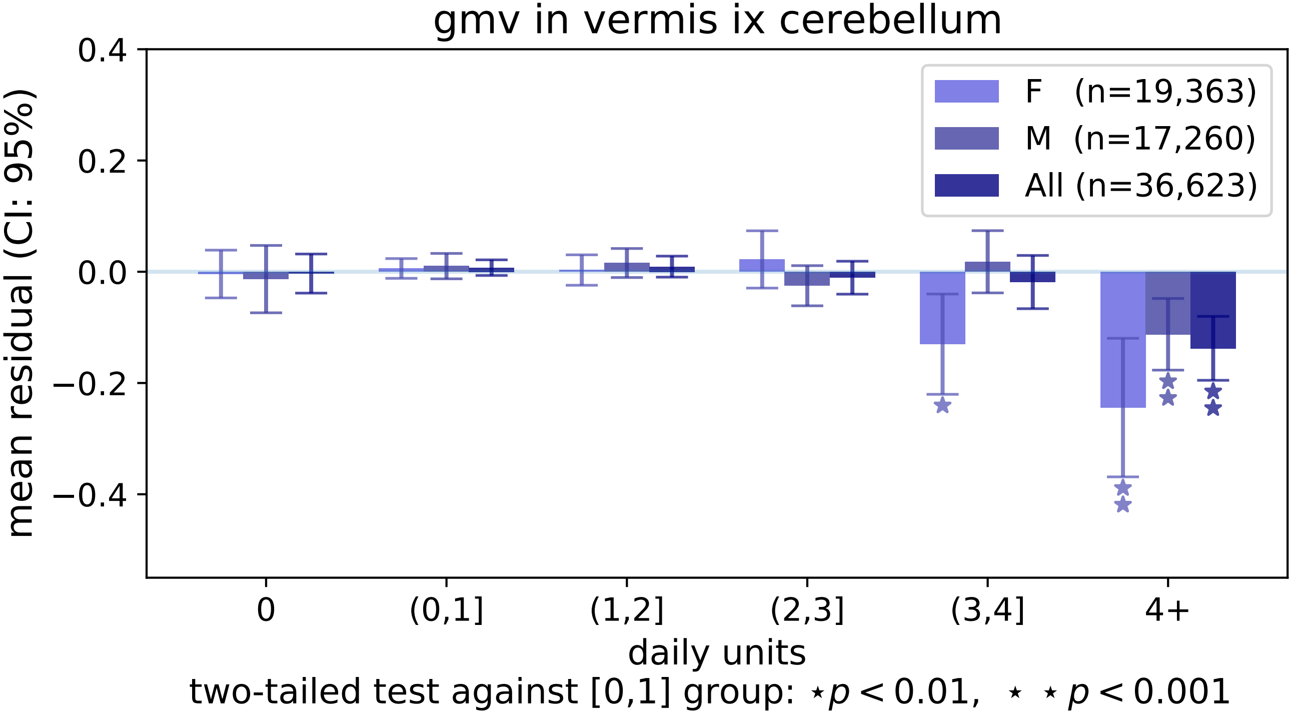

gmv in vermis ix cerebellum
mean residual (CI: 95%)
0.4
0.2
0.0
−0.2
−0.4
F (n=19,363)
M (n=17,260)
All (n=36,623)
0
(0,1]
(1,2]
(2,3]
(3,4]
4+
daily units
two-tailed test against [0,1] group: ⋆ p < 0.01, ⋆ ⋆ p < 0.001

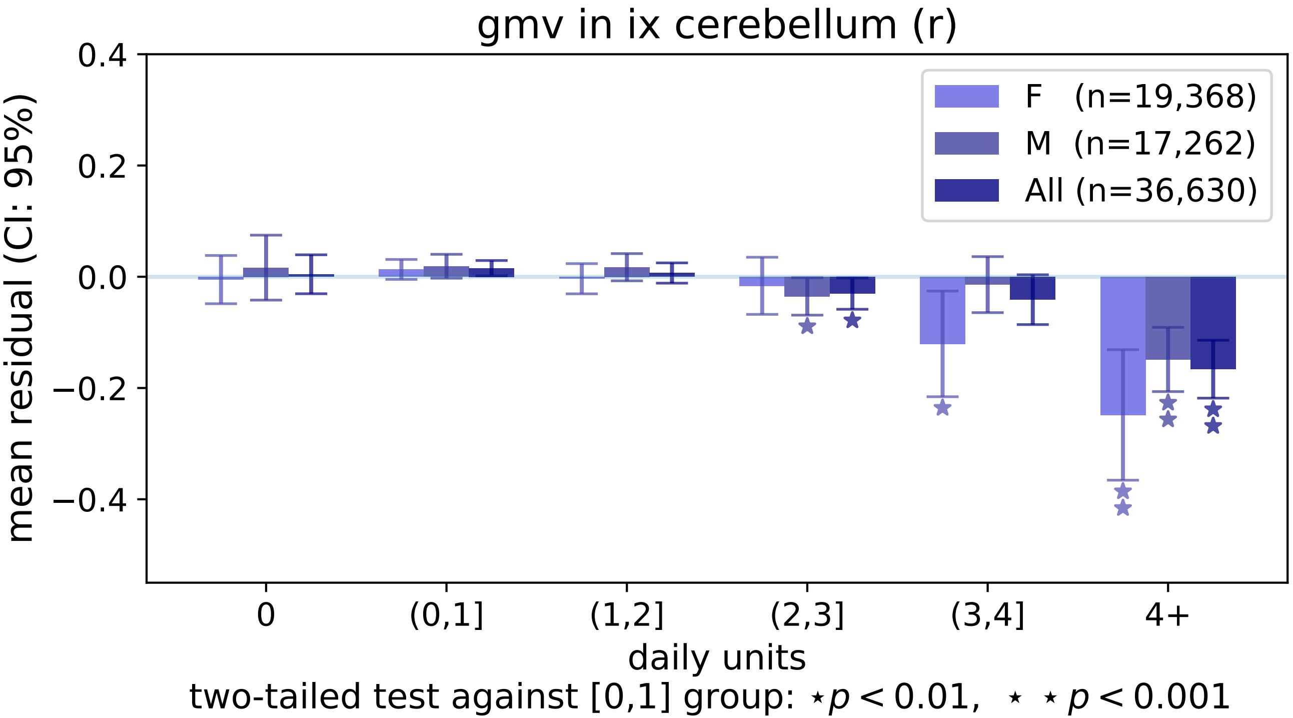

gmv in ix cerebellum (r)
mean residual (CI: 95%)
0.4
0.2
0.0
−0.2
−0.4
F (n=19,368)
M (n=17,262)
All (n=36,630)
0
(0,1]
(1,2]
(2,3]
(3,4]
4+
daily units
two-tailed test against [0,1] group: ⋆ p < 0.01, ⋆ ⋆ p < 0.001

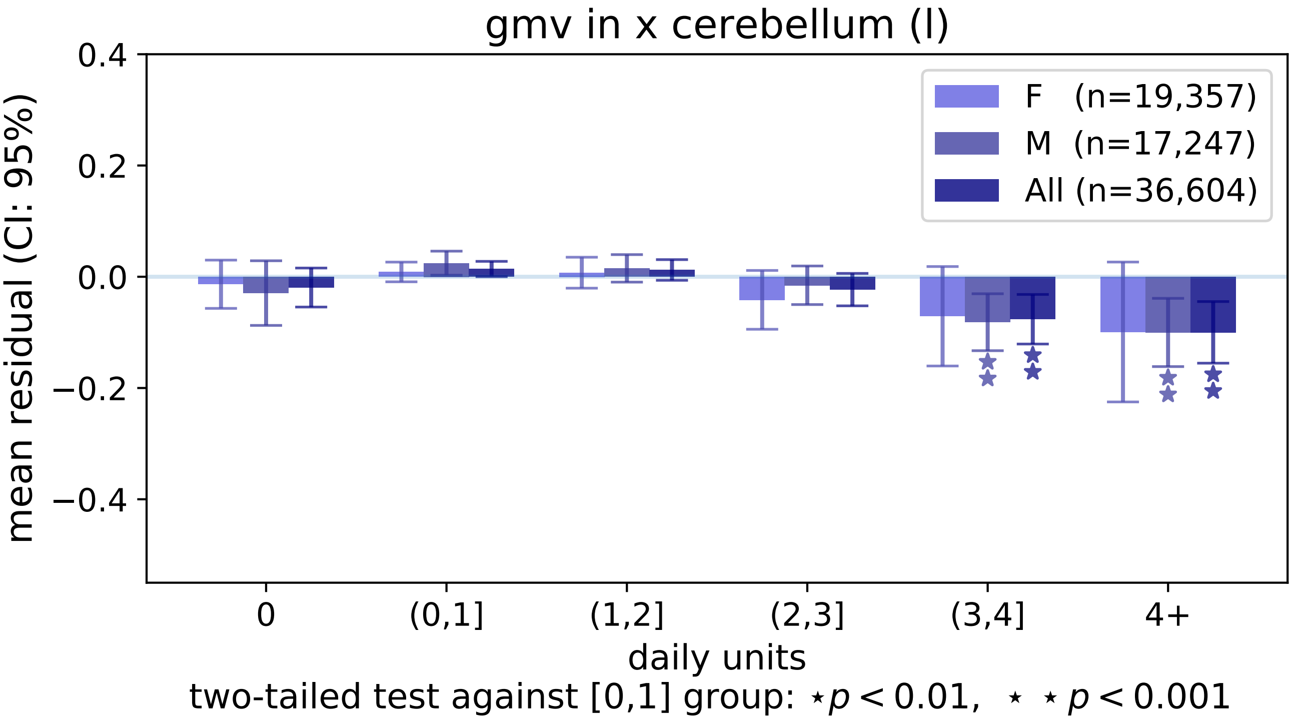

gmv in x cerebellum (l)
mean residual (CI: 95%)
0.4
0.2
0.0
−0.2
−0.4
F (n=19,357)
M (n=17,247)
All (n=36,604)
0
(0,1]
(1,2]
(2,3]
(3,4]
4+
daily units
two-tailed test against [0,1] group: ⋆$p < 0.01$, ⋆ ⋆ $p < 0.001$

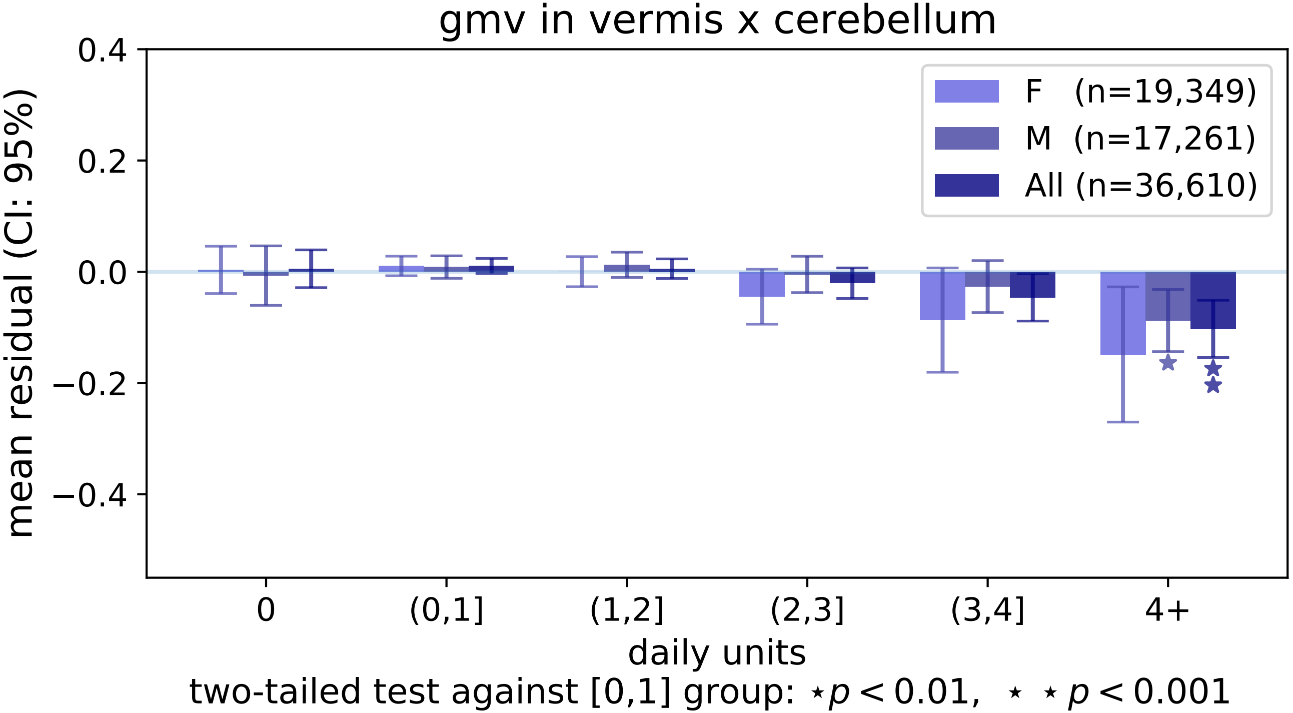

gmv in vermis x cerebellum
mean residual (CI: 95%)
0.4
0.2
0.0
−0.2
−0.4
F (n=19,349)
M (n=17,261)
All (n=36,610)
0
(0,1]
(1,2]
(2,3]
(3,4]
4+
daily units
two-tailed test against [0,1] group: ⋆p < 0.01, ⋆ ⋆ p < 0.001

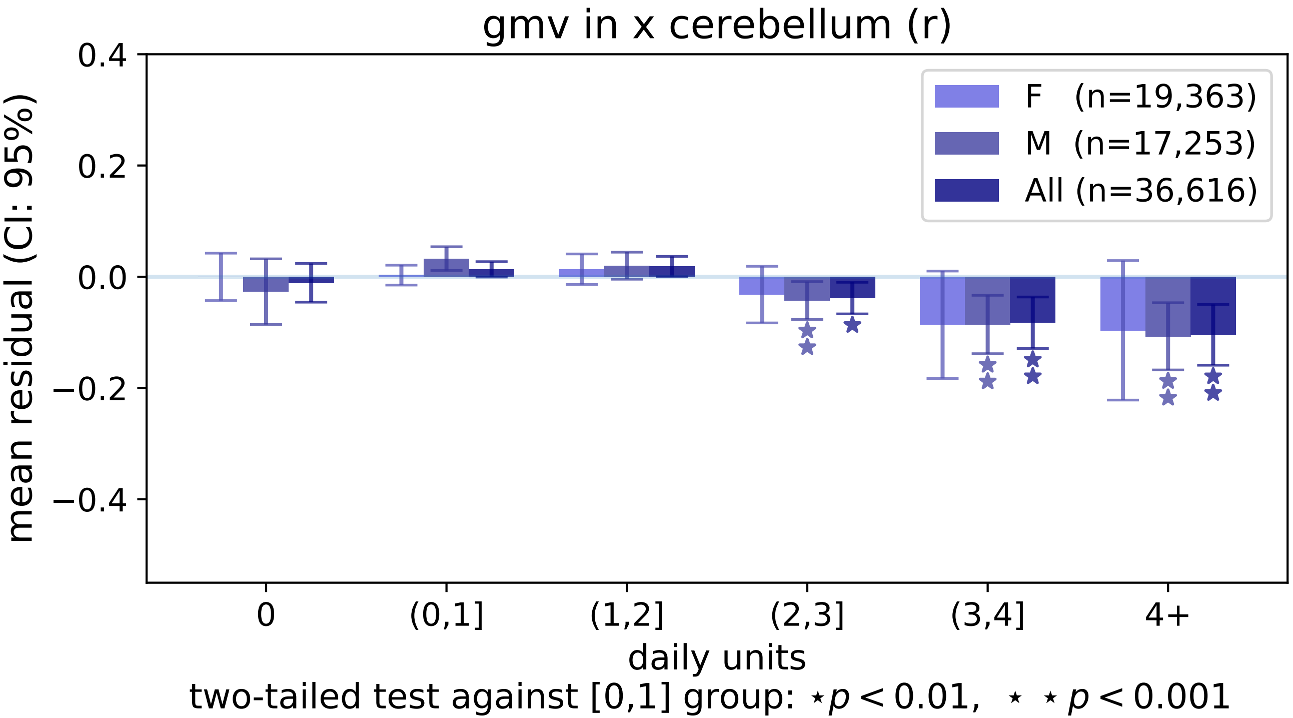
gmv in x cerebellum (r)
mean residual (CI: 95%)
0.4
0.2
0.0
−0.2
−0.4
F (n=19,363)
M (n=17,253)
All (n=36,616)
0
(0,1]
(1,2]
(2,3]
(3,4]
4+
daily units
two-tailed test against [0,1] group: ⋆p < 0.01, ⋆ ⋆ p < 0.001

Supplementary Figure 4. Associations between daily alcohol units and white matter microstructure indices of interest across white matter tract regions. Asterisks denote statistically significant effects in F-test, corrected for multiple hypothesis testing (Holm method), $p < 1.64 \times 10^{-4}$. Colors represent the expected change in each imaging derived phenotype resulting from the increase in daily consumption from 2 to 3 units, based on the regression model. FA = fractional anisotropy, ICVF = intracellular volume fraction, ISOVF = isotropic volume fraction, MD = mean diffusivity, OD = orientation dispersion, r = right, l = left

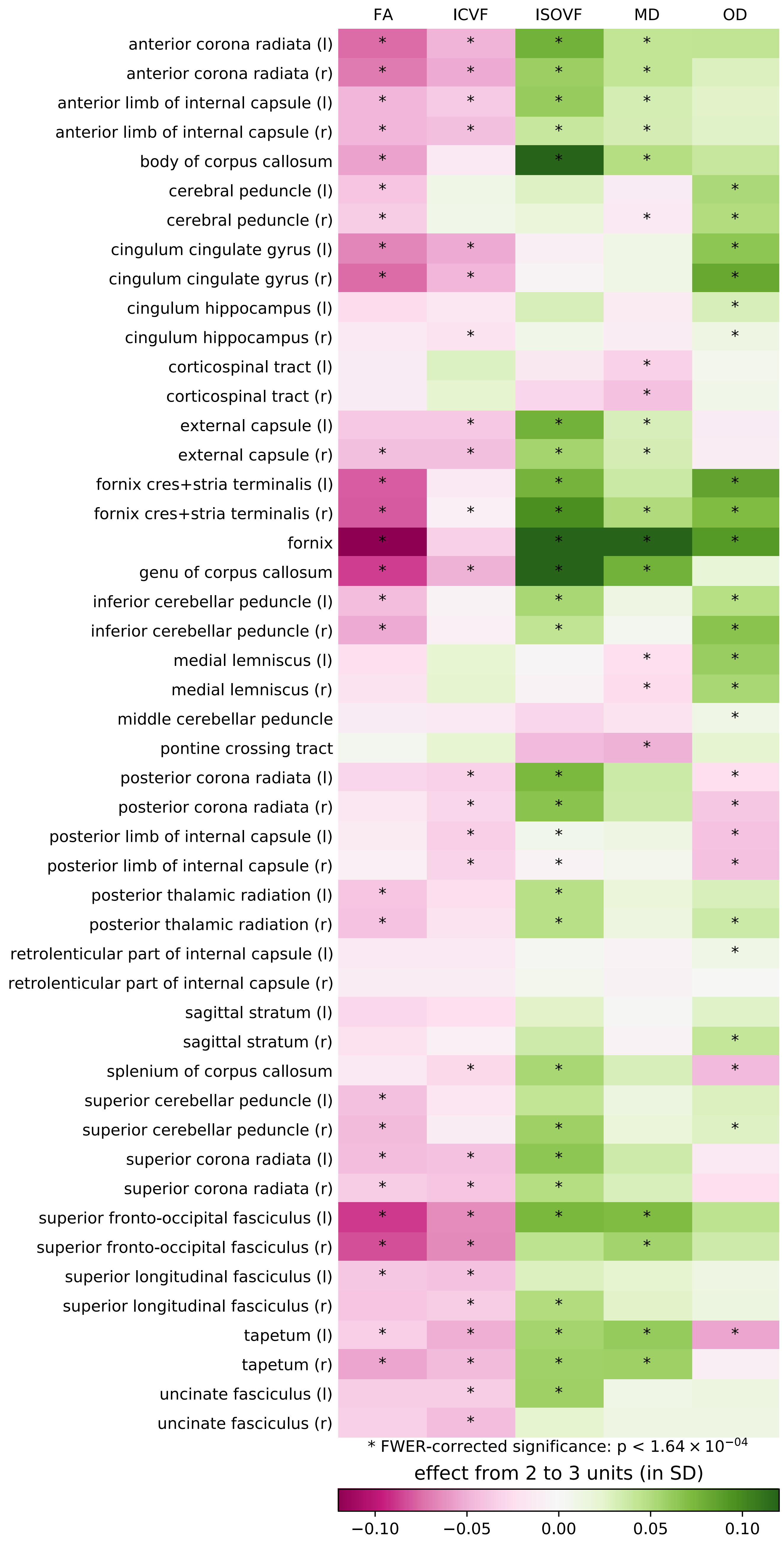
FA ICVF ISOVF MD OD
anterior corona radiata (l)
anterior corona radiata (r)
anterior limb of internal capsule (l)
anterior limb of internal capsule (r)
body of corpus callosum
cerebral peduncle (l)
cerebral peduncle (r)
cingulum cingulate gyrus (l)
cingulum cingulate gyrus (r)
cingulum hippocampus (l)
cingulum hippocampus (r)
corticospinal tract (l)
corticospinal tract (r)
external capsule (l)
external capsule (r)
fornix cres+stria terminalis (l)
fornix cres+stria terminalis (r)
fornix
genu of corpus callosum
inferior cerebellar peduncle (l)
inferior cerebellar peduncle (r)
medial lemniscus (l)
medial lemniscus (r)
middle cerebellar peduncle
pontine crossing tract
posterior corona radiata (l)
posterior corona radiata (r)
posterior limb of internal capsule (l)
posterior limb of internal capsule (r)
posterior thalamic radiation (l)
posterior thalamic radiation (r)
retrolenticular part of internal capsule (l)
retrolenticular part of internal capsule (r)
sagittal stratum (l)
sagittal stratum (r)
splenium of corpus callosum
superior cerebellar peduncle (l)
superior cerebellar peduncle (r)
superior corona radiata (l)
superior corona radiata (r)
superior fronto-occipital fasciculus (l)
superior fronto-occipital fasciculus (r)
superior longitudinal fasciculus (l)
superior longitudinal fasciculus (r)
tapetum (l)
tapetum (r)
uncinate fasciculus (l)
uncinate fasciculus (r)
* FWER-corrected significance: p < 1.64 × 10^−04
effect from 2 to 3 units (in SD)
−0.10 −0.05 0.00 0.05 0.10

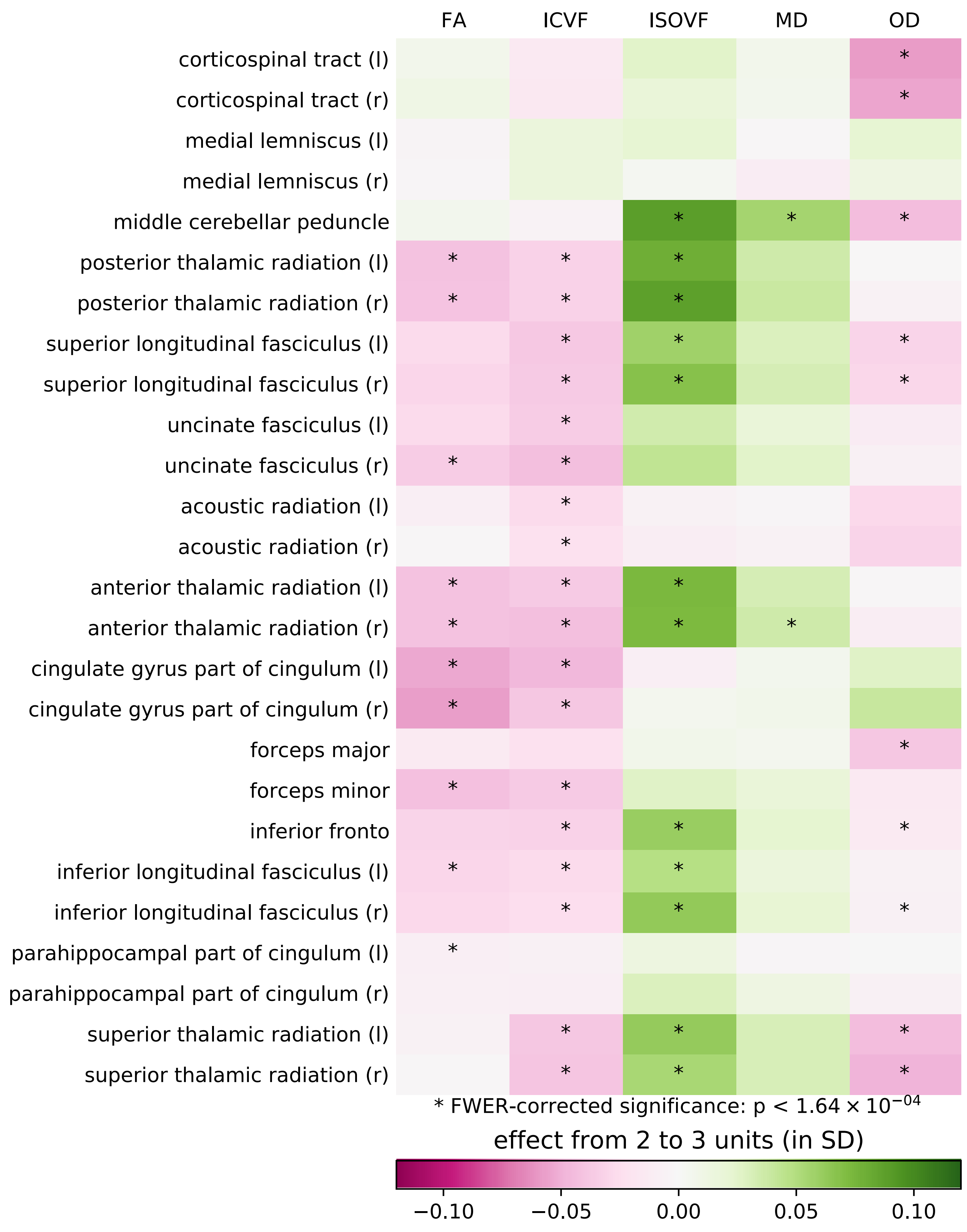
FA ICVF ISOVF MD OD
corticospinal tract (l)
corticospinal tract (r)
medial lemniscus (l)
medial lemniscus (r)
middle cerebellar peduncle
posterior thalamic radiation (l)
posterior thalamic radiation (r)
superior longitudinal fasciculus (l)
superior longitudinal fasciculus (r)
uncinate fasciculus (l)
uncinate fasciculus (r)
acoustic radiation (l)
acoustic radiation (r)
anterior thalamic radiation (l)
anterior thalamic radiation (r)
cingulate gyrus part of cingulum (l)
cingulate gyrus part of cingulum (r)
forceps major
forceps minor
inferior fronto
inferior longitudinal fasciculus (l)
inferior longitudinal fasciculus (r)
parahippocampal part of cingulum (l)
parahippocampal part of cingulum (r)
superior thalamic radiation (l)
superior thalamic radiation (r)
* FWER-corrected significance: p < 1.64 × 10^−04
effect from 2 to 3 units (in SD)
−0.10 −0.05 0.00 0.05 0.10
