# Supplementary material for: Associations between alcohol consumption and gray and white matter volumes in the UK Biobank
Source: Nat Commun. 2022 Mar 4;13:1175. doi: 10.1038/s41467-022-28735-5 (PMC8897479; doi:10.1038/s41467-022-28735-5)
Supplement: Supplementary file 3 — Reporting summary [file 41467_2022_28735_MOESM3_ESM.pdf]

nature research

Corresponding author(s): Wetherill, Reagan
Nave, Gideon
Daviet, Remi

Last updated by author(s): Jan 19, 2022

Reporting Summary

Nature Research wishes to improve the reproducibility of the work that we publish. This form provides structure for consistency and transparency in reporting. For further information on Nature Research policies, see Authors & Referees and the Editorial Policy Checklist .

Statistics

For all statistical analyses, confirm that the following items are present in the figure legend, table legend, main text, or Methods section.

n/a	Confirmed	
☐	☒	The exact sample size (*n*) for each experimental group/condition, given as a discrete number and unit of measurement
☐	☒	A statement on whether measurements were taken from distinct samples or whether the same sample was measured repeatedly
☐	☒	The statistical test(s) used AND whether they are one- or two-sided<br>*Only common tests should be described solely by name; describe more complex techniques in the Methods section.*
☐	☒	A description of all covariates tested
☐	☒	A description of any assumptions or corrections, such as tests of normality and adjustment for multiple comparisons
☐	☒	A full description of the statistical parameters including central tendency (e.g. means) or other basic estimates (e.g. regression coefficient) AND variation (e.g. standard deviation) or associated estimates of uncertainty (e.g. confidence intervals)
☐	☒	For null hypothesis testing, the test statistic (e.g. *F*, *t*, *r*) with confidence intervals, effect sizes, degrees of freedom and *P* value noted<br>*Give P values as exact values whenever suitable.*
☒	☐	For Bayesian analysis, information on the choice of priors and Markov chain Monte Carlo settings
☒	☐	For hierarchical and complex designs, identification of the appropriate level for tests and full reporting of outcomes
☒	☐	Estimates of effect sizes (e.g. Cohen's *d*, Pearson's *r*), indicating how they were calculated

Our web collection on statistics for biologists contains articles on many of the points above.

Software and code

Policy information about availability of computer code

Data collection	N/A
Data analysis	Analyses code is available on the project's page on OSF: https://osf.io/qkp4g/

For manuscripts utilizing custom algorithms or software that are central to the research but not yet described in published literature, software must be made available to editors/reviewers. We strongly encourage code deposition in a community repository (e.g. GitHub). See the Nature Research guidelines for submitting code & software for further information.

Data

Policy information about availability of data

All manuscripts must include a data availability statement. This statement should provide the following information, where applicable:
- Accession codes, unique identifiers, or web links for publicly available datasets
- A list of figures that have associated raw data
- A description of any restrictions on data availability

The data is publicly available to researchers upon application to the UK Biobank: https://www.ukbiobank.ac.uk/

Field-specific reporting

Please select the one below that is the best fit for your research. If you are not sure, read the appropriate sections before making your selection.

☒ Life sciences ☐ Behavioural & social sciences ☐ Ecological, evolutionary & environmental sciences

For a reference copy of the document with all sections, see nature.com/documents/nr-reporting-summary-flat.pdf

Life sciences study design

All studies must disclose on these points even when the disclosure is negative.

Sample size	Our final sample consisted of N = 36,678 individuals with brain scans and genotype data, all of the imaged UKB participants as of 18 Oct 2018.
Data exclusions	From the text:<br>Our sample comprised 36,678 individuals of European ancestry from the UKB, all study participants whose data were available as of September 1, 2020. The number of participants included in each model decreased when phenotype data were missing. All of the structural T1 MRI images that we used passed the automated quality control of the UKB brain imaging processing pipeline.29 We ran additional quality checks using the Computational Anatomy Toolbox (CAT; www.neuro.uni-jena.de/cat/) for SPM (www.fil.ion.ucl.ac.uk/spm/software/spm12/), which resulted in 747 individuals who exhibited substantial image inhomogeneity (i.e., overall volume correlation below two standard deviations from the mean) being removed from the analysis.
Replication	The analysis was pre-registerd, the code is available for replication. Replication will be possible upon the release of new brain images from the UK Biobank. Replication was successful.
Randomization	Given that this is a non-experimental study, randomization was not used.
Blinding	Given that this is a non-experimental study, blinding was not used or necessary.

Reporting for specific materials, systems and methods

We require information from authors about some types of materials, experimental systems and methods used in many studies. Here, indicate whether each material, system or method listed is relevant to your study. If you are not sure if a list item applies to your research, read the appropriate section before selecting a response.

Materials & experimental systems

n/a	Involved in the study	
☒	☐	Antibodies
☒	☐	Eukaryotic cell lines
☒	☐	Palaeontology
☒	☐	Animals and other organisms
☐	☒	Human research participants
☒	☐	Clinical data

Methods

n/a	Involved in the study	
☒	☐	ChIP-seq
☒	☐	Flow cytometry
☐	☒	MRI-based neuroimaging

Human research participants

Policy information about studies involving human research participants

Population characteristics	We used publicly available data from the UK Biobank (UKB), which recruited 502,617 people aged 40 to 69 years from the general population across the United Kingdom. The data used in analyses consists of 36,678 participants (52.8% female)
Recruitment	Recruitment was done by the UK Biobank
Ethics oversight	All UKB participants provided written informed consent and the study was granted ethical approval by the North West Multi-Centre Ethics committee.

Note that full information on the approval of the study protocol must also be provided in the manuscript.

Magnetic resonance imaging

Experimental design

Design type	N/A (T1 anatomical scans)
Design specifications	N/A
Behavioral performance measures	N/A

Acquisition

Imaging type(s)	T1
Field strength	The images were acquired using a 3-T Siemens Skyra scanner, with a 32-channel head coil (Siemens, Erlangen, Germany)
Sequence & imaging parameters	T1-weighted images were obtained using an MPRAGE sequence with the following parameters: TR=2000ms; TE=2.01ms; 208 sagittal slices; flip angle, 8°; FOV=256 mm; matrix=256×256; slice thickness=1.0mm (voxel size 1×1×1mm); total scan time=4min 54s. 3D FLAIR images were obtained with the following parameters: TR=1800ms; TE=395.0ms; 192 sagittal slices; FOV=256mm; 256×256; slice thickness=1.05mm (voxel size 1.05×1×1mm); total scan time=5min 52s.
Area of acquisition	Whole brain

Diffusion MRI [x] Used [ ] Not used

Parameters: Diffusion acquisition comprised a spin-echo echo-planar sequence with 10T2-weighted (b≈0 s mm−2) baseline volumes, 50b=1000s mm−2 and 50b=2000s mm−2 diffusion weighted volumes, with 100 distinct diffusion-encoding directions and 2mm isotropic voxels; total scan time=6min 32s.

Preprocessing

Preprocessing software	Structural imaging and diffusion data were processed by the UK Biobank team and made available to approved researchers as imaging-derived phenotypes (IDPs); the full details of the image processing and QC pipeline are available in an open-access article. IDPs used in analyses included whole-brain GMV, whole-brain WMV, 139 regional GMV IDPs derived using parcellations from the Harvard-Oxford cortical and subcortical atlases and Diedrichsen cerebellar atlas (UKB fields 25782 to 25920), and 375 tract-averaged measures of fractional anisotropy (FA), mean diffusivity (MD), intra-cellular volume fraction (ICVF), isotropic volume fraction (ISOVF), and orientation diffusion (OD) extracted by averaging parameters over 74 different white-matter tract regions based on subject-specific tractography and from population-average white matter masks. Volumetric IDPs were normalized for head size by multiplying the raw IDP by the T1-based "head size scaling factor".
Normalization	See above
Normalization template	See above
Noise and artifact removal	See above
Volume censoring	N/A

Statistical modeling & inference

Model type and settings	multivariate linear regression (fixed effects model)
Effect(s) tested	Regression beta coefficients

Specify type of analysis: [ ] Whole brain [ ] ROI-based [x] Both

Anatomical location(s): We relied on the imaging-derived phenotypes (IDPs) provided by the UKB brain imaging processing pipeline, which used parcellations from the Harvard-Oxford cortical and subcortical atlases and the Diedrichsen cerebellar atlas.

Statistic type for inference (See Eklund et al. 2016)	Voxel-wise
Correction	Permutation test

Models & analysis

n/a	Involved in the study
[x]	[ ] Functional and/or effective connectivity
[x]	[ ] Graph analysis
[x]	[ ] Multivariate modeling or predictive analysis
